# Loneliness as an Interface Between Alzheimer's Disease and Suicidal Behaviour: A Systematic Review, Meta‐Analysis and Meta‐Analytic Factor Analysis

**DOI:** 10.1111/psyg.70165

**Published:** 2026-04-07

**Authors:** Juliano Flávio Rubatino Rodrigues, Lívia Peregrino Rodrigues, Taiki Teshima, Shiyun Yang, Yunfan Wu, Xujing Hu, Giovanka Isabel Figueroa Abarca, Keila Cristianne Trindade da Cruz, María Fernanda Serna Rodriguez, Fernando Victor Martins Rubatino, Miyae Yamakawa, Moacir Fernandes de Godoy, Gerardo Maria de Araújo Filho

**Affiliations:** ^1^ Faculdade de Medicina de São José do Rio Preto (FAMERP) São José do Rio Preto São Paulo Brazil; ^2^ International Psychogeriatric Association (IPA) Milwaukee Wisconsin USA; ^3^ Unimed Bauru Bauru São Paulo Brazil; ^4^ Faculdade de Medicina da Universidade de Marília (UNIMAR) Marília São Paulo Brazil; ^5^ The University of Osaka Osaka Japan; ^6^ Kansai Medical University Hirakata Osaka Japan; ^7^ National Institute of Geriatrics (INGER) Santiago Chile; ^8^ Universidade de Brasília Brasília Federal District Brazil; ^9^ Universidad Autónoma de Nuevo León San Nicolás de los Garza Nuevo León Mexico; ^10^ Universidade Presidente Antônio Carlos (UNIPAC) Conselheiro Lafaite Minas Gerais Brazil

**Keywords:** Alzheimer's disease, meta‐analysis, meta‐analytic factor analysis, suicidal behaviour, suicidal ideation, suicidal planning, suicide, suicide attempt, systematic review

## Abstract

Loneliness is an epidemic affecting mental health across all demographics. It is linked to mental disorders, such as anxiety and depression, and despair, highlighting a significant public health issue as persons feel more disconnected in a connected world. This study aims to investigate the relationship between loneliness, Alzheimer's disease and suicidal behaviour. This review was systematised in a dichotomous manner. Therefore, two systematic reviews were initially carried out following the PRISMA statement. The loneliness was understood as feeling lonely. One group searched for associations between loneliness and Alzheimer's disease and the other between loneliness and suicidal behaviour, with a consecutive meta‐analysis. After that, it was searched for between the two groups to seek loneliness, such as an interface in meta‐analytic factor analysis. Depression is the most studied and cited factor associated with loneliness as a link between Alzheimer's disease and suicide. Loneliness demonstrated association with Alzheimer's disease (OR = 1.89, 95% CI 1.57–2.28; *p* < 0.001); suicidal ideation (OR = 2.17, 95% CI 1.88–2.51; *p* < 0.001); suicidal planning (OR = 2.36, 95% CI 1.91–2.92; *p* < 0.001); suicide attempt (OR = 2.54, 95% CI 2.13–3.04; *p* < 0.001); and suicide (OR = 4.9, 95% CI 4.4–5.5; *p* < 0.001). Entrapment, hopelessness, insomnia and stress demonstrated significative correlation (*r* > 0.40; *p* < 0.001) with loneliness in the interface between AD and suicidal behaviour. Loneliness has been identified as a comorbid factor between AD and suicide. To prevent both AD and suicide, it is essential to monitor levels of stress, insomnia, feelings of entrapment and hopelessness. The triad of loneliness, hopelessness and insomnia seems to represent the greatest risk profile.

## 1 Introduction

In our rapidly evolving and interconnected world, the pervasive issue of loneliness has emerged as a significant concern, profoundly affecting the well‐being of countless individuals. This growing epidemic is staggering; research indicates that one in every six people across the globe grapples with feelings of isolation. The ramifications of loneliness extend far beyond mere emotional distress, as it has been linked to alarming health consequences. Astonishingly, it is estimated that loneliness is associated with approximately 100 deaths every hour, culminating in more than 871 000 fatalities each year. This silent epidemic highlights the urgent need to address the causes and effects of loneliness, fostering connections and support for those who are affected [1].

This profound sense of isolation often leads to feelings of despair and is closely linked to various mental health disorders, resulting in a complex web of emotional distress [2]. The stress resulting from loneliness became increasingly apparent during the COVID‐19 pandemic. This period highlighted a troubling connection between social isolation and the rise of depression and anxiety disorders, as many individuals faced prolonged periods of solitude and disconnection from their communities [3].

Mental disorders that are provoked by feelings of loneliness have been increasingly linked to a heightened risk of suicidal behaviour. This alarming rise in suicide rates serves as a stark reminder of the deep and pervasive effects that isolation can have on mental health. It underscores the urgent necessity for empathetic and effective support systems that address these emotional struggles and provide a lifeline to those in need [4].

Beyond the emotional distress that loneliness can inflict, research indicates that it may also lead to significant cognitive impairments. This connection becomes even more concerning considering the troubling increase in neurodegenerative conditions like Alzheimer's disease (AD). Such a rise adds layers of complexity to an already alarming issue, underscoring the intricate bond that exists between our emotional health and cognitive functioning. This interplay emphasises the pressing need for a comprehensive and holistic strategy to effectively tackle the challenges posed by loneliness and its repercussions on mental acuity [5]. Furthermore, the connections between cognitive decline and emotional dysfunction are intense focuses of study in the manifestations of Mild behaviour impairment (MBI) [6].

The links between neurodegenerative disorders and suicidal behaviour have been the focus of intense research by the scientific community [7]. Having observed the theory of the association between Alzheimer's disease and suicide (ASA theory), we propose that loneliness could serve as a significant link between Alzheimer's disease and the risk of suicide. This hypothesis suggests that the profound sense of isolation often experienced by individuals suffering from Alzheimer's may contribute to an increased vulnerability to suicidal thoughts and behaviours. There is important to investigate loneliness such as an important interface between Alzheimer's disease and suicide, due to the explanation of commonalities in ASA Theory [8].

To effectively address this complex and multifaceted issue, it is imperative to thoroughly examine the intricate relationship between loneliness and various mental health challenges. Such a comprehensive investigation will facilitate the development of targeted interventions and holistic support systems that are thoughtfully designed to address the diverse needs of individuals facing these difficulties. By identifying the underlying factors and interconnections, we can create environments that promote authentic relationships, empathy and support, ultimately guiding individuals toward healing and resilience in the face of modern life's challenges [9]. This current study seeks to explore the complex relationship between loneliness and its role as a potential interface connecting AD and suicidal behaviour. By examining how feelings of isolation may influence individuals living with Alzheimer's, we aim to gain a deeper understanding of the psychological challenges they face and the implications for their mental health and well‐being.

In this study, we use the term interface to denote a shared psycho‐biological pathway through which loneliness may simultaneously intensify Alzheimer's disease–related neurocognitive vulnerability and elevate suicidal risk. This concept extends beyond existing work on shared risk factors such as depression or social isolation, proposing a unifying mechanism that has not been systematically synthesised in prior reviews. According to ASA Theory, suicidal behaviour and Alzheimer's disease can be studied together. However, to facilitate understanding, we decided to start with distinct fields and gradually explore their intersection. As anticipated, we found no primary studies that examined the relationship between loneliness, Alzheimer's disease and suicide simultaneously. This distinction can be explained by ASA Theory, as these are opposing phenomena that manifest differently, despite sharing common risk factors. Finally, in relation to the ultimate goal of this review—which is to study the intersection of these topics—we adopt the premise of ASA Theory and consider Alzheimer's disease and suicide as a single morbidity. Based on these theoretical and philosophical foundations, we employed rigorous scientific and statistical methods for our measurements. Consequently, the method used, factor analysis, aims to identify common risk factors at this intersection. To achieve our objective, we proceed with some steps to answer: (i) first, we conduct an extensive comprehensive review of the association between loneliness and suicidal behaviour or AD and describe the principal findings; (ii) we seek to identify the associated factor with loneliness and AD; (iii) we seek to identify the associated factor with loneliness and suicidal behaviour; (iv) we seek to understand what factors are common between Alzheimer's disease and suicide in the occurrence of loneliness; (v) to conduct a meta‐analysis of chance of AD in previous loneliness and vice‐versa; (vi) to conduct a meta‐analysis of chance of suicidal behaviour in previous loneliness and vice‐versa; (vii) to conduct a factor analysis of commonalities between AD and suicidal behaviour in associated with loneliness.

## 2 Methods

### 2.1 Study Design

According to ASA Theory, suicidal behaviour and Alzheimer's disease could be studied together, but to facilitate understanding, we chose to start from distinct fields and arrive at the interface between the two. So, we adopted a two‐stage analytic pipeline: (i) two parallel systematic reviews examining loneliness in relation to Alzheimer's disease and suicidal behaviour, followed by meta‐analyses in each domain; and (ii) a meta‐analytic factor analysis integrating correlational structures from eligible studies to identify shared correlates across both conditions. This overview is provided to clarify the flow of evidence synthesis. This study was registered in protocols.io with the doi: https://doi.org/10.17504/protocols.io.3byl4ze22vo5/v1 and followed the PRISMA statement methodology [10].

### 2.2 Search Strategy

The defined papers' titles and abstracts are eligible and non‐restricted languages without time limits. Furthermore, it was systematically identified by searching electronic databases Embase [Emtree—Major Focus Exp.], Pubmed [Mesh Terms], Lilacs—Complete collection of the Virtual Health Library [Title/abstracts], 医中誌 (Ichushi) Web, 知网 (CNKI), 万方 (Wanfang Data) and 维普 (VIP) in November 2024.

This review was systematised in a dichotomous manner. Therefore, two systematic reviews were initially carried out. In one, the search terms to Embase, Lilacs and Pubmed included “suicide” OR “suicidal behaviour” OR “suicidal ideation” OR “suicide attempt” AND “loneliness.” In Ichushi Web the following descriptors were used: “自殺” OR “自殺未遂” OR “自殺念慮” OR “自殺既遂” OR “自殺行動” AND “孤独” OR “社会的孤立.” For Chinese databases the following descriptors were used: “自杀” OR “自杀行为” OR “自杀意念” OR “自杀未遂” AND “孤独” OR “孤独感.” In the CNKI database, logical operators such as “”, “+,” and “−” are used in place of “AND,” “OR,” and “NOT.” Consequently, the search query was modified to: (自杀 + 自杀行为 + 自杀意念 + 自杀未遂) * (孤独 + 孤独感). For Wanfang Data and VIP databases, the query used was “M = (自杀 OR 自杀行为 OR 自杀意念 OR 自杀未遂) AND M = (孤独 OR 孤独感)” where “M” specifies a search within titles and keywords. The PECOS strategy was specified with the following terms: Population (P) = individuals; exposure (E) = loneliness; comparison (C) = without loneliness; outcome (O) = suicide; and study design (S) = all kinds of studies with an association between loneliness and suicide.

In the second group to review, the search terms to Embase, Lilacs and Pubmed included “alzheimer” OR “alzheimer's” OR “alzheimer's disease” AND “loneliness” (Embase Emtree—Major Focus Exp. “alzheimer” AND “loneliness”). In Ichushi Web the following descriptor were uses: “Alzheimer病” OR “アルツハイマー” OR “認知症” AND “孤独” OR “社会的孤立.” For Chinese databases the following descriptors were used: In the CNKI database, the query used was (阿尔兹海默症 + 阿尔兹海默病 + 阿尔茨海默氏症 + Alzheimer + 老年痴呆症 + 痴呆症) * (孤独 + 孤独感). For Wanfang Data and VIP databases, the query used was “M = (阿尔兹海默症 OR 阿尔兹海默病 OR 阿尔茨海默氏症 OR Alzheimer OR 老年痴呆症 OR 痴呆症) AND M = (孤独 OR 孤独感).” The PECOS strategy was specified with the following terms: Population (P) = individuals; exposure (E) = loneliness; comparison (C) = without loneliness; outcome (O) = Alzheimer's disease; and study design (S) = all kinds of studies with an association between loneliness and suicide. It was research studies in grey literature, too.

To be included, the article had to address loneliness and something about suicide or Alzheimer's disease. To be eligible, studies met the following criteria: They had to include a paper that reported loneliness and suicidal behaviour and loneliness and Alzheimer's disease. Loneliness in caregivers of Alzheimer's patients and loneliness triggered by someone's death by suicide were not included in this systematic review. The terms for inclusion regarding loneliness were ‘loneliness’, ‘being alone’, ‘living alone’, ‘aloneness’, ‘solitude’ and ‘lonely’. The terms for inclusion regarding suicide were ‘suicide’, ‘suicidal behaviour’, ‘suicidal ideation’, ‘suicide attempt’, ‘suicide programming’ and ‘suicidality’. The terms for inclusion regarding ‘Alzheimer's Disease’ were ‘Alzheimer’, ‘Alzheimer's’ and ‘Alzheimer's Disease’. We excluded every study that did not describe a possible association between loneliness and suicide or loneliness and Alzheimer's disease.

After that, it searched for similarities between the two groups to find a solution to loneliness, such as an interface. Researchers examined whether the concept of loneliness was approached differently across the studies. Any identified differences were categorised into separate groups.

### 2.3 Definition of Loneliness

The concept of loneliness has been debated for a long time. Many types of understanding have been proposed to define loneliness. Sometimes, it is used as social isolation synonymous. The search for a better‐known definition of loneliness continues [11]. Here, loneliness was understood as feeling lonely.

### 2.4 Definition of Suicidal Behaviour

This study understood suicidal behaviour, such as a continuum between suicidal ideation to planning suicide and attempting suicide with or without death.

### 2.5 Definition of Alzheimer's Disease

Diagnostic criteria for Alzheimer's disease have undergone constant modifications in the last decade [12]. We used the parameters established by the International Working Group (IWG) and the US National Institute on Aging and the Alzheimer's Association (NIA‐AA) as references [13]. Since we did not limit the search in time, there will be differences in the diagnostic understanding of many studies. However, understanding Alzheimer's disease as a syndrome, we chose to include all articles that included the term ‘Alzheimer's disease’ in their nomenclature and carefully reviewed those included for statistical evaluation to ascertain their quality. Those that did not receive case definitions had reduced quality scores. This review included studies with clinical diagnostics, biomarkers, or autopsy definitions of Alzheimer's disease.

### 2.6 Screening

Six authors (J.F.R.R., L.P.R., T.T., S.Y., Y.W. and W.H.) systematically searched the papers. A total of 3.576 studies published from 1946 to 2025 were first screened, and their titles and abstracts were reviewed.

After the first screening, the eligible 787 papers were read in full. All papers were printed for handling. Two authors (J.F.R.R. and L.P.R.) manually assessed the studies included in the review to select them and perform all statistical calculations. In cases of doubt regarding the inclusion of any article, a third author was consulted for the decision (G.M.A.F.).

**FIGURE 1:**
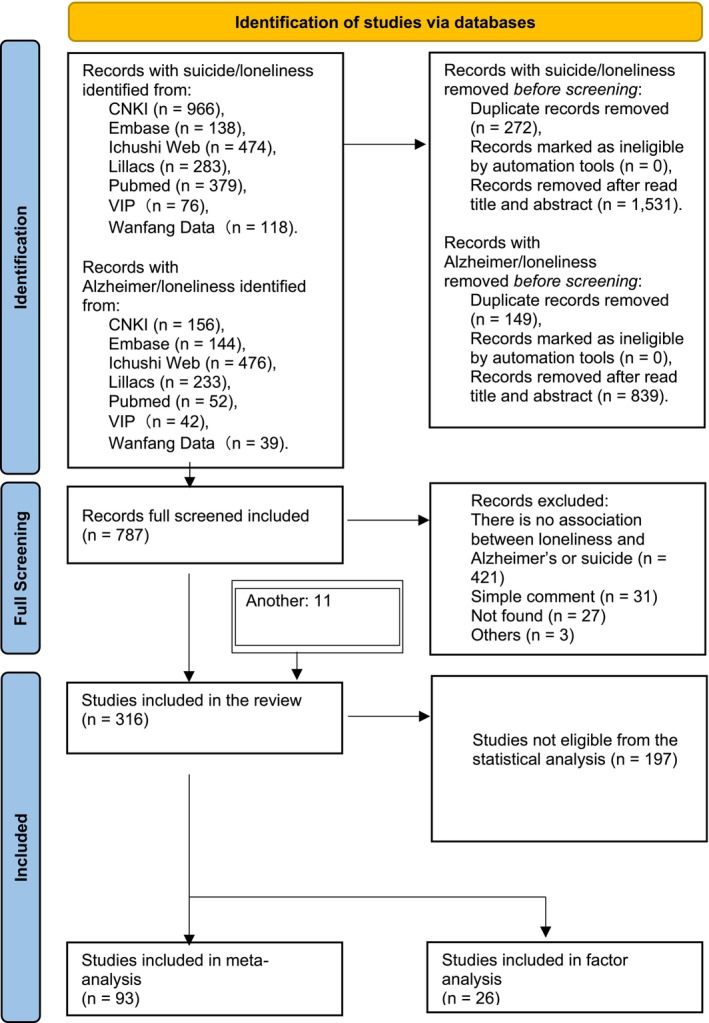
Flowchart.

Studies that evaluated loneliness and dementia in a general context without specifically distinguishing Alzheimer's disease were excluded from consideration. Additionally, comments that did not present a thorough literature review were also excluded (Figure 1).

The screening of studies for the meta‐analysis, from those included in the review, was carried out by two authors (J.F.R.R. and F.C.P.R.). A third author was consulted in the event of any doubts (L.P.R).

### 2.7 Data Extraction Strategies

The English language was chosen to record all data. Factors associated with loneliness were described, as well as sample size, study type, main results and conclusion. All studies were manually analysed by two authors (J.F.R.R. and L.P.R.). Data were registered in Tables S1 and S2. All Supporting Information can be found in the following link: https://zenodo.org/records/18302143 [DOI: 10.5281/zenodo.18302143].

### 2.8 Quality Assessment

The quality assessment was measured by Newcastle–Ottawa Quality Assessment Scale (NOQAS) [14], AXIX [15], R‐AMSTAR [16], STROBE‐MR [17], PEDro [18], ARRIVE [19, 20] and COREQ [21] scales. More details may be found in the Appendix A.

### 2.9 Data Analysis

After a systematic review of the two groups, the meta‐analysis and a factor analysis were conducted to understand better how loneliness could be understood with the interface between suicidal behaviour and Alzheimer's Disease.

#### 2.9.1 Meta‐Analysis

The data were entered into Excel for distribution and similarity assessment. Comparable were selected for meta‐analysis, with statistical calculations using IBM Corp. Released 2023, Version 29. Effect sizes were presented as odds ratios (OR), risk ratios (RR), or hazard ratios (HR). Heterogeneity was measured with *I* ^2^: high (> 80%), moderate (50%–80%) and low (< 50%). Statistical significance was assessed with z‐scores (> |1.96|). Homogeneity was evaluated using the *Q* test (*p* < 0.05 indicating high heterogeneity). Publication bias was assessed with a funnel plot and Egger's test, where a 95% confidence interval including zero indicated no significant bias. If not, it suggested bias, leading to a Trim‐and‐Fill analysis to gauge impact on effect size. A bubble plot and Galbraith plot were employed to further evaluate studies if asymmetry was detected.

#### 2.9.2 Mata‐Analytic Factor Analysis

Statistical dates in meta‐analytic factor analysis (MAFA) were pooled in the webMASEM system for one‐stage MASEM [22]. The MAFA was used to investigate correlation factors with loneliness in the interface between AD and suicidal behaviour. All studies that performed factor analysis and described correlation matrices with loneliness were included for MAFA. AD and suicidal behaviour were counted as morbusus factors. The aim of this study was to identify simultaneous correlates with the morbosus factor and loneliness. For this study, the quality of the MAFA was assessed using the *Q* coefficient and a *p*‐value less than or equal to 0.05 was considered adequate. Factors that showed a correlation greater than 0.40 were considered significant.

## 3 Results

A total of 3578 articles were found, 2434 in the suicide group and 1142 in the Alzheimer's group. After identifying the seven databases, 787 articles were selected for full reading. Studies were written in Chinese, English, French, German, Japanese, Norwegian, Portuguese, Spanish and Turkish.

It included 316 in this review study. No original research was found that investigated loneliness, AD and suicide at the same time. Two reviews described loneliness associated with AD or suicide [23, 24]. Forty‐four described an association between AD and loneliness. The other 261 described an association between suicide and loneliness. The included reviews were essential for identifying studies that were not initially included in our research.

Depression was the most associated factor described in the interface between loneliness and AD or suicide. Depression was associated with cognitive decline and interpersonal interaction [25]. The deficiency in social interaction has been considered a risk factor for the development of AD [26]. Depression, low social interaction and cognitive decline appear to act as an interface between AD and loneliness [27]. Depression is one of the 5 Ds in the model developed by Van Orden to explain the risk of suicide in older adults who feel lonely [28]. Loneliness has been shown to increase the risk of suicide in older adults with depression [29]. In a qualitative study, the interface between depression and loneliness was described as gross behaviour and a numb relationship [30]. Loss of mobility is another factor at the interface between depression, loneliness and suicide [31]. The loss of family support is another factor in this context [32].

Depression is linked to loneliness in a vicious cycle, where feelings of isolation worsen depression, and depression leads to a desire to isolate oneself. The fewer interpersonal interactions a person establishes, the greater the chance of cognitive impairment occurring [33]. Hinton and Levkoff conducted narrative interviews with caregivers and found that progressive memory loss is associated with feelings of loneliness [34]. It seems a vicious cycle occurs, where the loneliness caused by the cognitive impairment of AD worsens the course of the disease [35].

Loneliness is associated with abnormal structure and activity in brain areas such as the prefrontal cortex, insula, amygdala, hippocampus and posterior superior temporal cortex. Functional magnetic resonance imaging studies show it impacts attentional, visual and default mode networks, along with biological markers related to AD, like amyloid and tau. Findings on the ventral striatum and cerebellum were mixed [36]. This found were associated with epigenetic alterations, including decreased BDNF [37]. In animal studies, another important finding was that Aβ peptide increases with social isolation [38]. Loneliness‐related switch genes, including BCAM, NECTIN2, NPAS3, RBM38, PELI1, DPP10 and ASGR2, have been identified as genetic risk factors for AD [39]. In a Mendelian Randomisation, the genes of loneliness were the most important risk factor for suicide attempt (OR = 7.07, *p* = 0.05) [40].

Judging by the pace of publications in recent years, it's clear that loneliness has been intensely studied worldwide in these times of globalisation. The COVID‐19 pandemic has increased evidence suggesting that loneliness is associated with suicide risk [41]. In an effort to understand the associations between loneliness and behaviour, a scoping review published in 2023 identified determining factors such as drug use, mental disorders and economic problems [42]. A case–control study found a significant association with loneliness and suicide risk with an OR of 4.9 (95% CI: 4.4–5.5) [43].

### 3.1 Quality Assessment

The quality assessment was conducted individually, and the results were qualified for a collective evaluation according to the level of scientific evidence. Although the studies were qualified using scales appropriate for each specific type, all qualifications were divided into the same risk of bias groups, namely minimal, low, medium and high.

#### 3.1.1 Alzheimer's Disease and Loneliness Association Studies

##### 3.1.1.1 Case–Control Studies

Three case–control studies were included that investigated the association between AD and loneliness. One of them demonstrated a high risk of bias [44]. The other two showed a minimal risk of bias [45, 46].

##### 3.1.1.2 Cross‐Sectional Studies

Eight cross‐sectional studies were included that investigated the association between AD and loneliness. One of them demonstrated a high risk of bias [47]. Three demonstrated low risk of bias [48, 49, 50]. The other four demonstrated minimal risk of bias [51, 52, 53, 54].

##### 3.1.1.3 Cohort Studies

Eighteen cohort studies were included that investigated the association between AD and loneliness. One of them demonstrated a high risk of bias [55]. Three demonstrated a medium risk of bias [56, 57, 58]. Seven demonstrated low risk of bias [59, 60, 61, 62, 63, 64, 65]. The other seven demonstrated minimal risk of bias [66, 67, 68, 69, 70, 71, 72].

##### 3.1.1.4 Animal Studies

Two animal studies were included that investigated the association between AD and loneliness. Both demonstrated a medium risk of bias [38, 73].

##### 3.1.1.5 Mendelian Studies

Two Mendelian studies were included that investigated the association between AD and loneliness. One of them demonstrated a medium risk of bias [39]. The other demonstrated low risk of bias [74].

##### 3.1.1.6 Qualitative Study

One qualitative study was included that investigated the association between AD and loneliness, and demonstrated a medium risk of bias [34].

##### 3.1.1.7 Review Studies

Twelve review studies were included that investigated the association between AD and loneliness. Seven demonstrated a high risk of bias [23, 24, 25, 27, 35, 37, 75]. The other five demonstrated a medium risk of bias [26, 33, 36, 76, 77].

#### 3.1.2 Suicide and Loneliness Association Studies

##### 3.1.2.1 Case–Control Studies

Nine case–control studies were included that investigated the association between suicide and loneliness. One of them demonstrated a medium risk of bias [78]. The other eight demonstrated a low risk of bias [43, 79, 80, 81, 82, 83, 84, 85].

##### 3.1.2.2 Cross‐Sectional Studies

One hundred and eighty‐seven cross‐sectional studies were included that investigated the association between suicide and loneliness. Two of them demonstrated a high risk of bias [86, 87]. Four demonstrated a medium risk of bias [88, 89, 90, 91]. One hundred and nine demonstrated a low risk of bias [92, 93, 94, 95, 96, 97, 98, 99, 100, 101, 102, 103, 104, 105, 106, 107, 108, 109, 110, 111, 112, 113, 114, 115, 116, 117, 118, 119, 120, 121, 122, 123, 124, 125, 126, 127, 128, 129, 130, 131, 132, 133, 134, 135, 136, 137, 138, 139, 140, 141, 142, 143, 144, 145, 146, 147, 148, 149, 150, 151, 152, 153, 154, 155, 156, 157, 158, 159, 160, 161, 162, 163, 164, 165, 166, 167, 168, 169, 170, 171, 172, 173, 174, 175, 176, 177, 178, 179, 180, 181, 182, 183, 184, 185, 186, 187, 188, 189, 190, 191, 192, 193, 194, 195, 196, 197, 198, 199, 200]. Seventy‐one demonstrated minimal risk of [201, 202, 203, 204, 205, 206, 207, 208, 209, 210, 211, 212, 213, 214, 215, 216, 217, 218, 219, 220, 221, 222, 223, 224, 225, 226, 227, 228, 229, 230, 231, 232, 233, 234, 235, 236, 237, 238, 239, 240, 241, 242, 243, 244, 245, 246, 247, 248, 249, 250, 251, 252, 253, 254, 255, 256, 257, 258, 259, 260, 261, 262, 263, 264, 265, 266, 267, 268, 269, 270, 271].

##### 3.1.2.3 Cohort Studies

Forty‐one cohort studies were included that investigated the association between suicide and loneliness. Two of them demonstrated a high risk of bias [272, 273]. Nine demonstrated a medium risk of bias [274, 275, 276, 277, 278, 279, 280, 281, 282]. Twenty‐four demonstrated a low risk of bias [283, 284, 285, 286, 287, 288, 289, 290, 291, 292, 293, 294, 295, 296, 297, 298, 299, 300, 301, 302, 303, 304, 305, 306]. Six demonstrated a minimal risk of bias [307, 308, 309, 310, 311, 312].

##### 3.1.2.4 Clinical Trials

Four clinical trials were included that investigated the association between suicide and loneliness. One of them demonstrated a high risk of bias [313]. Another demonstrated a medium risk of bias [28]. Two demonstrated a low risk of bias [314, 315]. One demonstrated a minimal risk of bias [316].

##### 3.1.2.5 Mendelian Studies

On Mendelian randomisation was included that investigated the association between suicide and loneliness, and demonstrated a minimal risk of bias [40].

##### 3.1.2.6 Qualitative Study

Nine qualitative studies were included that investigated the association between suicide and loneliness. One of them demonstrated a high risk of bias [317]. Three studies demonstrated a medium risk of bias [318, 319, 320]. Four studies demonstrated a low risk of bias [30, 321, 322, 323]. One study demonstrated minimal risk of bias [324].

##### 3.1.2.7 Review Studies

Twenty‐Two review studies were included that investigated the association between suicide and loneliness. Seven of them demonstrated a high risk of bias [23, 24, 325, 326, 327, 328, 329]. Seven studies demonstrated a medium risk of bias [31, 32, 42, 330, 331, 332, 333]. Six studies demonstrated low risk of bias [29, 41, 334, 335, 336, 337]. Two studies demonstrated a minimal risk of bias [338, 339].

### 3.2 Meta‐Analysis

**FIGURE 2:**
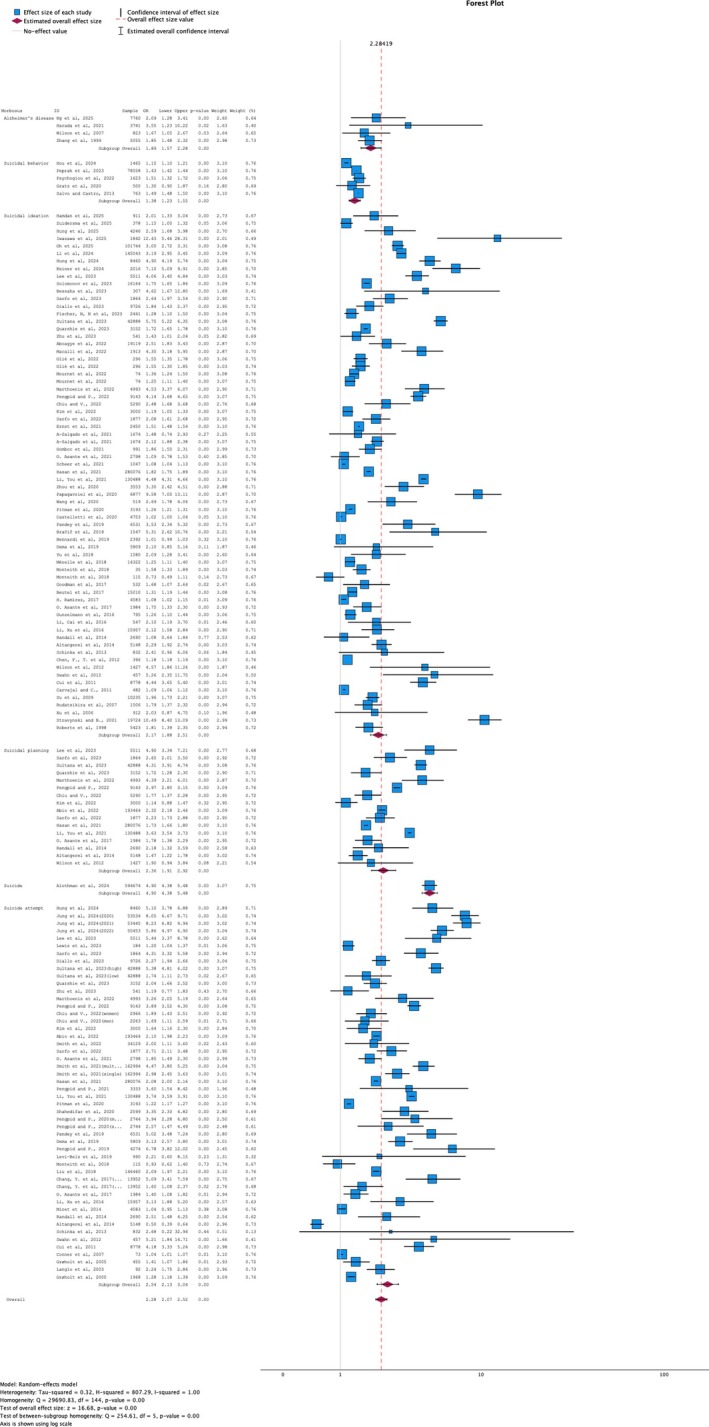
Association between loneliness and Alzheimer's disease or suicidal behaviour.

Given the diverse research fields, a random‐effects model was appropriate for the statistical analysis (Figure 2). After certifying the quality of studies, those with at least one pair of possible data points for comparison were included in the aggregate statistical calculations.

#### 3.2.1 Relation Between Alzheimer's Association and Loneliness

One cohort study was excluded due to a high risk of bias [55]. Another nine were excluded due to insufficient data for comparison [47, 56, 57, 58, 59, 60, 61, 63, 64, 68]. Four studies have measured the cumulative risk of developing AD in people who experience loneliness [62, 65, 66, 72]. The pooling data demonstrated low heterogeneity, with a RR of 1.64 [CI: 1.37–1.97; *p* < 0.01; *I* ^2^ = 28%; *z* = 5.44] (Supporting Information Graphic S1). Three studies have measured the immediate risk of developing AD in people who experience loneliness [67, 70, 71]. The pooling data demonstrated low heterogeneity, with an HR of 1.49 [CI: 1.27–1.74; *p* < 0.01; *I* ^2^ = 25%; *z* = 5.01] (Supporting Information Graphic S2). It was calculated the associated between AD and loneliness and pooling data demonstrated a high heterogeneity with an OR of 1.89 [CI: 1.57–2.28; *p* < 0.01; *I* ^2^ = 100%; *z* = 6.64] (Supporting Information Graphic S2a).

#### 3.2.2 Relation Between Suicidal Behaviour and Loneliness

It was possible to pool data from cross‐sectional and cohort studies to measure the odds ratio. Suicidal behaviour was distributed in three groups.

##### 3.2.2.1 Suicidal Ideation

Sixty‐six studies (cohort and cross‐sectional) were included in the meta‐analysis about the association between suicidal ideation and loneliness [90, 91, 92, 93, 104, 106, 107, 113, 114, 119, 121, 122, 134, 138, 139, 140, 142, 151, 153, 154, 157, 158, 162, 164, 169, 172, 173, 174, 176, 179, 181, 190, 194, 202, 204, 208, 209, 210, 227, 228, 229, 232, 233, 235, 236, 240, 242, 246, 247, 248, 249, 250, 251, 254, 255, 266, 270, 278, 288, 290, 291, 299, 303, 307, 309, 310]. The pooling data demonstrated high heterogeneity, with an OR of 2.17 [CI: 1.88–2.51; *p* < 0.01; *I* ^2^ = 100%; *z* = 10.58] (Supporting Information Graphic S3).

###### 3.2.2.1.1 Suicidal Ideation in Longitudinal Studies

The nine cohort studies demonstrated high heterogeneity, with an OR of 1.32 [CI: 1.12–2.56; *p* < 0.01; *I* ^2^ = 98%; *z* = 3.38] (Supporting Information Graphic S3b).

###### 3.2.2.1.2 Suicidal Ideation in Cross‐Sectional Studies

The 60 cross‐sectionals studies demonstrated high heterogeneity, with an OR of 2.34 [CI: 2.00–2.73; *p* < 0.01; *I* ^2^ = 100%; *z* = 10.66] (Supporting Information Graphic S3f).

##### 3.2.2.2 Suicidal Planning

Sixteen cross‐sectional studies were included in the meta‐analysis about the association between suicidal ideation and loneliness [104, 113, 121, 122, 124, 138, 153, 162, 164, 172, 227, 229, 233, 235, 236, 246]. The pooling data demonstrated high heterogeneity, with an OR of 2.36 [CI: 1.91–2.92; *p* < 0.01; *I* ^2^ = 99%; *z* = 7.99] (Supporting Information Graphic S4).

##### 3.2.2.3 Suicide Attempt

Forty‐three studies (cohort and cross‐sectional) were included in the meta‐analysis about the association between suicidal ideation and loneliness [104, 113, 114, 121, 122, 124, 126, 134, 135, 137, 138, 140, 142, 150, 153, 158, 162, 164, 173, 183, 188, 191, 199, 200, 209, 224, 227, 228, 229, 233, 235, 236, 246, 254, 262, 266, 268, 269, 270, 271, 276, 282, 303]. The pooling data demonstrated high heterogeneity, with an OR of 2.54 [CI: 2.13–3.04; *p* < 0.01; *I* ^2^ = 99%; *z* = 10.32] (Supporting Information Graphic S5). Egger's test demonstrates risk of bias [CI: 0.557–1.114].

###### 3.2.2.3.1 Suicide Attempt in Longitudinal Studies

The three cohort studies demonstrated high heterogeneity, with an OR of 1.47 [CI: 1.00–2.16; *p* < 0.01; *I* ^2^ = 82%; *z* = 1.94] (Supporting Information Graphic S5b).

###### 3.2.2.3.2 Suicide Attempt in Cross‐Sectional Studies

The 40 cross‐sectionals studies demonstrated high heterogeneity, with an OR of 2.64 [CI: 2.19–3.17; *p* < 0.01; *I* ^2^ = 99%; *z* = 10.33] (Supporting Information Graphic S5e).

#### 3.2.3 Meta‐Analytic Factor Analysis (MAFA)

Twenty‐six studies were included in MAFA [48, 60, 89, 99, 120, 131, 132, 141, 143, 146, 168, 171, 175, 178, 183, 184, 189, 195, 196, 202, 207, 211, 223, 257, 260, 303]. Seven factors had at least two correlations extracted: anxiety, depression, entrapment, hopelessness, insomnia, perceived burdensomeness and stress (Table 1). Four factors showed a correlation greater than 0.4 (*p* < 0.001) with the morbid factor for AD or suicidal behaviour—entrapment, hopelessness, insomnia and stress (Supporting Information Graphic S6).

**TABLE 1:** Meta‐analytic factor analysis of loneliness as an interface in morbosus (AD and suicidality).

	Morbosus	Anxiety	Depression	Entrapment	Hopelessness	Insomnia	Perceived burdensomeness	Stress
Morbosus	1.000							
Anxiety	0.354	1.000						
Depression	0.398	0.259	1.000					
Entrapment	0.466	0.354	0.316	1.000				
Hopelessness	0.628	0.397	0.474	0.466	1.000			
Insomnia	0.519	0.467	0.251	0.630	0.475	1.000		
Perceived burdensomeness	0.317	0.625	0.355	0.518	0.249	0.396	1.000	
Stress	0.474	0.523	0.398	0.316	0.353	0.466	0.626	1.000

*Note: Q* statistic of effect sizes: 3541.616. Degrees of freedom of the *Q* statistic: 197. *p* value of the *Q* statistic.

## 4 Discussion

### 4.1 Main Insights

This study aimed to investigate the factors associated with loneliness as an interface between Alzheimer's disease and suicide. Their results proved to be the largest review on loneliness as an association with Alzheimer's disease or suicidal behaviour. Moreover, it offers a promising research model for clarifying major questions regarding the relationship between neurodegenerative diseases and affective disorders.

It is well known that depression is strongly associated with loneliness, but this study clarified the importance of factors such as insomnia and hopelessness as key correlates between AD, suicide and loneliness. In these digital times, the association between loneliness and insomnia seems to have intensified [340].

### 4.2 Meta‐Analysis Heterogeneity Asymmetry

One study demonstrated risk of bias in meta‐analysis about AD and loneliness association [62]. This work was presented as a poster, and many characteristics of the participants were not described, making it difficult to know how the diagnoses of Alzheimer's disease and the characterisation of loneliness were made.

Two cohort studies demonstrated high asymmetry in meta‐analysis about the association between loneliness and suicidal ideation. One of them investigated loneliness during the pandemic, focusing more on the context of social isolation than on the actual feeling of loneliness [278]. The other study made a great approach to loneliness, but information on research into suicidal behaviour is scarce. Some patients were excluded due to cognitive difficulties, which leaves room for bias due to other neurodegenerative problems associated with suicidal behaviour, such as frontotemporal dementia [299].

Two cross‐sectional studies demonstrated high asymmetry in meta‐analysis about the association between loneliness and suicidal ideation. One of them investigated suicidal behaviour in adolescents, but they did not inform how loneliness was defined [138]. The other investigated suicidal ideation in severely obese bariatric surgery [169]. Although loneliness was investigated using appropriate instruments, the comparison group was not well characterised and may have included individuals with depression.

Two studies demonstrated high asymmetry in meta‐analysis about the association between loneliness and suicidal planning. One of them had the primary objective to investigate the association between bullying and suicidal behaviour, but the criteria for defining loneliness were unclear [246]. The other investigated suicidal behaviour in adolescents, but they did not inform how loneliness was defined [138].

Two studies demonstrated high asymmetry in meta‐analysis about the association between loneliness and suicide attempts. One of them investigated suicidal behaviour in adolescents, but they did not inform how loneliness was defined [138]. The other researched loneliness after sudden bereavement. The vast majority of the sample consisted of women (81%), and the control group, which may contain patients with depression, was not clearly described [140].

### 4.3 General Discussion

We observed that since the review by Qiao et al. [77] published in 2022, there have been few studies investigating the association between AD and loneliness. On the other hand, studies investigating the association between loneliness and suicidal behaviour are published all the time. It will be important for the scientific community to give the same importance to loneliness in the context of AD.

Most research on loneliness tends to be cross‐sectional, meaning that it often examines the associations between factors at a single point in time. While this approach allows for valuable insights into how different elements relate to loneliness, it does not provide a comprehensive understanding of true risk over time. More robust evidence can be derived from genetic or longitudinal cohort studies, which track participants over an extended period. An illustrative example of such research is the 9‐year study conducted by UK Biobank, which revealed a significant association between loneliness and suicide risk (HR = 1.43, 95% CI: 1.01–2.03) [295].

### 4.4 Strength and Limitations

The primary strength of this study is its comprehensive inclusion of a wide range of literature, which serves as an invaluable resource for researchers focused on understanding loneliness. This extensive compilation not only enriches the existing body of research but also provides a solid foundation for future investigations. However, one notable weakness is that the study did not explore the intricacies of the sex and age data in greater depth. This lack of detail may represent a missed opportunity, as a more thorough analysis could yield important insights and pave the way for future studies to further investigate these critical demographic factors. Moreover, suicidal behaviour and the subjective experience of loneliness vary substantially across sociocultural contexts; therefore, the pooled effect sizes in this review should be interpreted as cross‐cultural averages rather than universally generalisable estimates. The high heterogeneity of the results in the meta‐analysis reflects the advantage of a diverse group, but its data need to be viewed with caution in terms of generalisations.

### 4.5 Future Directions

The findings from this review emphasise a substantial gap in our comprehension of the intricate relationship between loneliness, insomnia and feelings of hopelessness. This gap underscores an urgent need for further exploration of how these elements interact with one another. By delving deeper into this relationship, researchers have the potential to uncover critical insights into the psychological and emotional factors that may lead to sleep disturbances and emotional upheaval. Understanding these connections could pave the way for more effective interventions aimed at addressing not only sleep issues but also the broader emotional distress that often accompanies them.

## 5 Conclusion

Depression emerges as a fundamental factor that significantly impacts an individual's experience of loneliness, especially in its intricate relationship with AD and the heightened risk of suicide that accompanies it. This pervasive loneliness is not merely a feeling; it is a complex interplay between depression, AD and the emotional turmoil that individuals endure. At the core of this experience are powerful contributors that intensify feelings of isolation and despair. Individuals often grapple with an overwhelming sense of entrapment, suffocating hopelessness and persistent disrupted sleep patterns marked by relentless insomnia. The stress levels prevalent in these situations can reach unbearable heights, further compounding the feelings of loneliness. Each of these factors intertwines to create a fragile emotional state, making those dealing with AD particularly vulnerable. As the isolation deepens, so does the risk of spiralling into suicidal thoughts and behaviours, underlining the urgent need for understanding and support for those caught in this profound struggle. The emotional landscape of these individuals is fraught with challenges that not only affect their mental well‐being but also highlight the critical need for compassionate intervention and care.

## Funding

The authors' resources were used.

## Conflicts of Interest

The authors declare no conflicts of interest.

## Supporting information


**Table S1:** Summary of studies included in the review of loneliness and Alzheimer's disease.

**Table S2:** Summary of studies included in the review of loneliness and suicidal behaviour.

**Data S1:** Supplementary statistics loneliness and AD‐SB.

## Data Availability

The data that support the findings of this study are available in the Supporting Information of this article.

## Appendix A

### Quality Assessment Details

The Newcastle–Ottawa Quality Assessment Scale (NOQAS) was used to measure the methodological quality of studies, controlling for publication bias in case–control and cohort studies [14]. Studies that scored zero to 2 were considered to be at high risk of bias. Those that scored 3–4 were supposed to be at medium risk of bias. Those that scored 5–6 were at low risk of bias. Those that scored 7 or more were at minimal risk of bias.

The AXIS evaluates the quality of cross‐sectional studies [15]. Each numbered item corresponds to one point, so the AXIS score ranges from 0 to 20. Based on the total AXIS score, studies will be categorised into risk of bias levels: Minimal risk of bias: Score of 15 or higher; Low risk of bias: Score of 10–14; Medium risk of bias: Score of 5–9; High risk of bias: Score of 0–4.

It will utilise the Revised Assessment of Multiple Systematic Reviews (R‐AMSTAR) to evaluate the methodological quality of studies and control for publication bias in systematic reviews [16]. The quality score R‐AMSTAR ranges from 11 to 44. Based on the total R‐AMSTAR score, studies will be categorised into risk of bias levels: Minimal risk of bias: Score of 36 or higher; Low risk of bias: Score of 26–35; Medium risk of bias: Score of 17–25; High risk of bias: Score of 11–16.

It used the Strengthening the Reporting of Observational Studies in Epidemiology Using Mendelian Randomisation (STROBE‐MR) checklist to measure the methodological quality of Mendelian randomisation studies [17]. Each item correctly corresponds to one point. The quality score STROBE‐MR ranges from 0 to 20. Based on the total STROBE‐MR score, studies will be categorised into risk of bias levels: Minimal risk of bias: Score of 15 or higher; Low risk of bias: Score of 10–14; Medium risk of bias: Score of 5–9; High risk of bias: Score of 0–4.

It used the PEDro scale to measure the methodological quality of clinical trials [18]. The quality score PEDro ranges from 0 to 11. Based on the total PEDro score, studies will be categorised into risk of bias levels: Minimal risk of bias: Score of 9 or more; Low risk of bias: Score of 6–8; Medium risk of bias: Score of 3–5; High risk of bias: Score of 0–2.

It used the ARRIVE guidelines 2.0 to measure the methodological quality of animal studies, controlling for publication bias in animal laboratory studies [19, 20]. Each item correctly corresponds to one point. The quality score ARRIVE ranges from 0 to 21. Based on the total ARRIVE score, studies will be categorised into risk of bias levels: Minimal risk of bias: Score of 15 or more; Low risk of bias: Score of 9–14; Medium risk of bias: Score of 4–8; High risk of bias: Score of 0–3.

It used the COREQ score to measure the methodological quality of qualitative research [21]. Each item correctly corresponds to one point. The quality score COREQ ranges from 0 to 32. Based on the total COREQ score, studies will be categorised into risk of bias levels: Minimal risk of bias: Score of 22 or more; Low risk of bias: Score of 15–21; Medium risk of bias: Score of 8–14; High risk of bias: Score of 0–7.

Quality assessment aims to contextualise the findings. It may guide sensitivity analyses, such as excluding studies with a high risk of bias, to evaluate the robustness of the overall results.
