# Supplementary material for: Loneliness as an Interface Between Alzheimer's Disease and Suicidal Behaviour: A Systematic Review, Meta‐Analysis and Meta‐Analytic Factor Analysis
Source: Psychogeriatrics. 2026 Apr 7;26(3):e70165. doi: 10.1111/psyg.70165 (PMC13055150; doi:10.1111/psyg.70165)
Supplement: Supplementary file 1 — Table S1: Summary of studies included in the review of loneliness and Alzheimer's disease. [file PSYG-26-0-s002.docx]

**Table 1. Summary of studies included in the review of loneliness and Alzheimer’s Disease**

| **Citation** | **Sample size** | **Quality** | **Design** | **Associated factor** | **Study aim** | **Relevant Result** |
| --- | --- | --- | --- | --- | --- | --- |
| Ng et al, 2025^66^ | 7,760 | 7 | Cohort | Cognitive decline | To investigate the association between loneliness and cognitive decline/incident AD. | They found association between loneliness and incident AD, OR: 2·117 (1.227–3·655,0·006). |
| Kehrer-Dunlap et al, 2024^56^ | 150 | 3 | Cohort | Anxiety;  Depression. | To investigate the relationship between amyloid accumulation and subjective experiences of loneliness among cognitively normal older adults during the COVID-19 pandemic. | They found a significant positive association was detected between loneliness and amyloid accumulation (β = 0·064, SE = 0·027, 95% CI = [0·011, 0·118], p = 0·018). |
| Suehiro et al, 2024^51^ | 43 | 16 | Cross-sectional | Depression,  Regional cerebral blood flow. | To investigate the relationship between loneliness and regional cerebral blood flow (rCBF) in AD. | They found even though the loneliness group was more depressed and had more impaired proximal memory, the non-loneliness group had lower rCBF in the precuneus and wedge. The precuneus is said to be involved in self-reflection, suggesting that AD patients, who normally suffer from reduced blood flow in the precuneus, may be less likely to feel lonely unless blood flow in the precuneus is maintained to some extent. |
| Lao et al, 2024^59^ | 680 | 6 | Cohort | Depression;  Black Race;  Cerebral vascular disease. | To investigate if greater loneliness would be associated with greater Alzheimer’s Disease (AD) and cerebrovascular pathology; greater cognitive impairment across all domains independently of pathology; and a stronger negative association between pathology and cognition, reflecting lower cognitive reserve. | They found in weighted models, greater loneliness was associated with microinfarcts, lower episodic and working memory in the absence of AD pathology, lower working memory in the absence of infarcts, a stronger association of infarcts with lower episodic memory, and a stronger association of microinfarcts with lower working and semantic memory. |
| Danish et al, 2024^52^ | 39 | 15 | Cross-sectional | Depression;  Obstrutive sleep apnea. | To investigate whether loneliness is associated with poor sleep quality and sleep apnea in older women at risk of developing AD. | They found that higher loneliness was significantly associated to lower habitual sleep efficiency and sleep duration and was also influenced by use of sleep medication. |
| Zhao et al, 2024^53^ | 381 | 15 | Cross-sectional | Depression;  APOE4 carrier  status. | To investigate whether loneliness was associated with advanced neuroimaging markers of AD using neuroimaging data from Framingham Heart Study participants without dementia. | They found no associations were observed between loneliness and either Aβ or tau deposition in primary regions of interest. |

AD: Alzheimer’s disease; APOE: apolipoprotein E; APOE ε4: apolipoprotein allele ε4; CVD: cardiovascular disease; FTD: frontotemporal dementia; VD: Vascular dementia

Continue on the next page

**Continuing Table 1. Summary of studies included in the review of loneliness and Alzheimer’s Disease**

| **Citation** | **Sample size** | **Quality** | **Design** | **Associated factor** | **Study aim** | **Relevant Result** |
| --- | --- | --- | --- | --- | --- | --- |
| Kotwal et al, 2024^54^ | 72 | 15 | Cross-sectional | Decrease social network. | To investigate the perspectives of persons with  dementia and care partners on changes to their social lives, focusing on experiences with loneliness and social isolation. | They found that persons with dementia and care partners experienced a gradual loss of social connections as the disease progressed which affected overall well-being and the ability to mobilize supportive resources. |
| Oken et al, 2024^25^ | - | 14 | Review | Depression. | To review the impact of chronic loneliness on cognitive impairment and dementia among older adults. | They found that loneliness may decrease resilience, or produce greater cognitive change associated with the same level of AD neuropathology. |
| Joshi et al, 2024^26^ | - | 20 | Review | Have poorer social connections. | To review associations of social connections with cognitive decline and trials of technology-based and other social interventions to enhance social connections in AD. | They found that social support reduces the risk of AD was weak. |
| Santiago et al, 2023^39^ | 409 | 9 | Mendelian | Depression,  Schizophrenia. | To investigate the genetic association between loneliness, neurodegenerative and neuropsychiatric diseases. | They found molecular determinants of loneliness and dysregulated pathways in the brain of non-demented adults. The association of switch genes with known risk factors for neuropsychiatric and neurodegenerative diseases provides a molecular explanation for the observed prevalence of these diseases among lonely individuals. |
| Ren et al, 2023^35^ | - | 12 | Review | Have poorer social connections. | To investigate the effect of loneliness and social isolation on AD development and the onset of AD symptoms in humans and animal models, respectively. | They found that social isolation in animal models of AD has been associated with increased amyloid plaque deposition, neurofibrillary tangles, neuroinflammation and altered hormonal stress responses. |
| Sutin et al, 2023^67^ | 492,322 | 8 | Cohort | APOE ε4;  Depression. | To investigate the association between loneliness and risk of incident all-cause dementia and whether the association extends to specific causes of dementia. | They found that loneliness was associated with a nearly 60% increased risk of all-cause dementia (HR=1·59, 95% CI=1·51–1·65; n=7,475 incident all-cause). In cause-specific analyses, loneliness was a stronger predictor of VD (HR=1·82, 95% CI=1·62–2·03; n=1,691 incident VD) than AD (HR=1·40, 95% CI=1·28–1·53; n=3135 incident AD), and was, surprisingly, a strong predictor of FTD (HR=1·64, 95% CI=1·22–2·20; n=252 incident FTD). |

Continue on the next page

**Continuing Table 1. Summary of studies included in the review of loneliness and Alzheimer’s Disease**

| **Citation** | **Sample size** | **Quality** | **Design** | **Associated factor** | **Study aim** | **Relevant Result** |
| --- | --- | --- | --- | --- | --- | --- |
| Shafighi et al, 2023^60^ | 532,603 | 6 | Cohort | Alcohol abuse;  CVD;  Diabetes;  Physical exercise;  Sleep disturbance;  Smoking. | To investigate the risk factors for developing dementia in the context of loneliness and lacking social support. | They found that of social isolation on ADRD remains in its infancy relative to the current evidence on other classical risk factors. |
| Valencia-Contrera et al, 2023^76^ | - | 18 | Review | CVD;  Depression  Frailty. | To review pathologies in the elderly associated with loneliness. | They found that association between loneliness and cognitive impairment, decreased physical fitness, Alzheimer’s disease and cardiovascular events is highlighted. |
| Haj et al, 2022^44^ | 62 | 2 | Case-control | Anxiety;  Depression. | To investigate the effect of COVID lockdown in patients with AD. | They found higher levels of depression [t(61)=4·68, P<0·001, Cohen’s d=0·50], anxiety [t(61)=3·23, P=0·002, Cohen’s d=0·45], and loneliness [t(61)=2·41, P=0·019, Cohen’s d=0·35]. |
| Saris et al, 2022^45^ | 163 | 7 | Case-control | Anxiety;  Depression. | To investigate social disfunction in patients with schizophrenia or AD. | They found that more AD symptomatology was associated with fewer prosocial activities (β = -0·400, p = 0·014). More negative SZ symptomatology was associated with less interpersonal functioning (β = -0·330, p = 0·010), and more positive SZ symptoms related to more loneliness (β = 0·278, p = 0·049). |
| Lao et al, 2022^55^ | 960 | 2 | Cohort | Fewer years of schooling. | To investigate the interaction between loneliness and AD or cerebrovascular pathology on cognitive performance. | They found negative association between loneliness and cognition. Loneliness does not appear to be related to AD or cerebrovascular pathology directly. |
| Salinas et al, 2022^61^ | 2,308 | 6 | Cohort | APOE ε4. | To investigate the association of loneliness with 10-year all-cause dementia risk and early cognitive and neuro-anatomic imaging markers. | They found that lonely participants <80 years of age without APOE ε4 alleles had a 3-fold greater risk (adjusted hazard ratio 3·03, 95% CI, 1·63–5·62). |
| Qiao et al, 2022^77^ | 62,345 | 25 | Review | Alcohol abuse;  Hypertension;  Smoking. | To review all available cohort studies to assess the association between loneliness and dementia among individuals of the general population. | They found that loneliness was associated with an increased risk of AD (RR: 1·72, 95% CI: 1·32–2·23; P < 0·001) and dementia (RR: 1·23, 95% CI: 1·16–1·31; P < 0·00001). |

Continue on the next page

**Continuing Table 1. Summary of studies included in the review of loneliness and Alzheimer’s Disease**

| **Citation** | **Sample size** | **Quality** | **Design** | **Associated factor** | **Study aim** | **Relevant Result** |
| --- | --- | --- | --- | --- | --- | --- |
| Morese et al, 2022^75^ | - | 14 | Review | Depression;  Hypertension;  Smoking. | To review association between loneliness and AD. | They found that social cognition is linked to social inclusion, while impairments in social cognition due to social brain dysfunction are connected to feelings of loneliness. |
| Elovainio et al, 2022^68^ | 155,033 | 8 | Cohort | CVD;  Depression. | To investigate whether genetic risk may intensify and attenuate the associations of social isolation and loneliness with the risk of dementia. | They found that a higher polygenic risk score (PRS) for AD was associated with an increased risk of dementia. Using continuous PRS, the HR per 1 SD increase in the score was 1·27 (95% CI 1·21 to 1·34) in an analysis adjusted for age, sex and 10 principal components. |
| Kotwal et al, 2022^47^ | 40 | 3 | Cross-sectional | Progressive cognitive  deficits. | To investigate association between loneliness and AD. | They found that loneliness was associated with cognition decline. |
| Zhang et al, 2022^69^ | 176 | 7 | Cohort | Alcohol abuse;  Smoking. | To investigate the influence of perceived social isolation or loneliness on brain structure and future cognitive trajectories in patients who are living with or are at risk for AD. | They found that loneliness was a significant predictor (P=0.05) of annual ADAS-Jcog change in the SCD-MCI group and not the AD group (P=0·40). |
| Saris et al, 2022^48^ | 150 | 14 | Cross-sectional | Schizophrenia. | To investigate association of loneliness with AD and schizophrenia. | They found social dysfunction transdiagnostically associates with social connection disintegrated across schizophrenia and AD. |
| Lam et al, 2021^36^ | 16,771 | 21 | Review | APOE ε4. | To review published brain-based findings linked to loneliness. | They found a significant relationship between loneliness  and higher amyloid burden, especially in APOE ε4 carriers. |
| Akhter-Khan et al, 2021^70^ | 2,880 | 7 | Cohort | APOE ε4. | To investigate the relationship between individual changes of loneliness in mid-life and risk of dementia and AD 18 years later. | They found that social network, physical health, and Apolipoprotein ε4, persistent loneliness was associated with higher (HR, 1·91; 95%CI 1·25–2·90; p<0.01), and transient loneliness with lower (HR, 0·34; 95%CI 0·14–0·84; p<0.05), risk of dementia onset, compared with no loneliness. Results were similar for AD risk. Those with persistent loneliness had higher risk, of developing both dementia (13·4% vs. 7·5%, p < 0.01) and AD (10·6% vs.  6·0%, p < 0·01) |

Continue on the next page

**Continuing Table 1. Summary of studies included in the review of loneliness and Alzheimer’s Disease**

| **Citation** | **Sample size** | **Quality** | **Design** | **Associated factor** | **Study aim** | **Relevant Result** |
| --- | --- | --- | --- | --- | --- | --- |
| Shen el al, 2021^74^ | 1,245,472 | 11 | Mendelian randomization | Poor religiosity. | To investigate examine bidirectional relationships between social isolation, social interaction, and Alzheimer’s disease using Mendelian randomization method for assessing potential causal inference. | They found that AD was associated with reducing attendance at a religious group (0·017 in attendance at a religious group per AD versus control status; 95% CI: 1·005–1·030; p = 0·004). |
| Harada et al, 2021^62^ | 2,651 | 6 | Cohort | Depression. | To investigate both depression and loneliness on incident dementia. | They found increases in incident AD were seen across the 4 groups (depression only RR=1·80, 95% CI=0·87-3·73, p=0·111; loneliness only RR=2·51, 95% CI=1·43-4·41, p=0·001; and both RR=2·51, 95%CI=1·33-4·76, p=0·005; p for trend <0·001). |
| Tragantzopoulou et al, 2021^23^ | - | 11 | Review | Low social networks. | To review social isolation and loneliness while highlighting the serious repercussions on health and behavior. | They found that older adults who felt lonely were more than twice as likely to develop an AD-like dementia syndrome, than those who were not lonely. |
| Sundström et al, 2020^71^ | 1,905 | 8 | Cohort | CVD;  Depression;  Fewer years of schooling. | To investigate the effect of perceived loneliness on the development of dementia (all-cause), AD and VD. | They found that report of perceived loneliness, showed increased risk of all-cause dementia (hazard ratio [HR] = 1·46, 95% CI 1·14–1·89), and AD (HR = 1·69, 95% CI 1·20–2·37), but not VD (HR = 1·34, 95% CI 0·87–2·08). |
| Gimenez-Llort et al, 2020^73^ | 37 | 5 | **Animals (Disease Modeling)** | Anxiety. | To investigate the observations of derangement of digging patterns in the subgroup of male 3xTg-AD mice that, after 10 months of social life, lost their partners and lived alone during the last 2–3 months. | They found that combined behavioral paradigm unveiled distinct features of digging signatures that can be useful to study these perseverative behaviors and their interplay with anxiety states already present in the AD scenario and their worsening by naturalistic/forced isolation. |
| Muntsant et al, 2020^38^ | 29 | 6 | **Animals (Disease Modeling)** | Anxiety. | To investigate the impact of long-term isolation in male 3xTg-AD mice modeling advanced stages of AD and as compared to age-matched counterparts with normal aging. | They found an exacerbated (two-fold increase) hyperactivity and emergence of bizarre behaviors in isolated 3xTg-AD mice, worrisome results since agitation is a challenge in the clinical management of dementia and an important cause of caregiver burden. |
| Victor et al, 2020^63^ | 1,547 | 5 | Cohort | Depression. | To investigate the prevalence and determinants of loneliness among people living with dementia. | They found nonsignificant statistics prevalence of loneliness in AD (p=0·465). |

Continue on the next page

**Continuing Table 1. Summary of studies included in the review of loneliness and Alzheimer’s Disease**

| **Citation** | **Sample size** | **Quality** | **Design** | **Associated factor** | **Study aim** | **Relevant Result** |
| --- | --- | --- | --- | --- | --- | --- |
| Balouch et al, 2019^57^ | 93 | 4 | Cohort | Depression. | To investigate the relationship between loneliness and social network and cognitive and psychopathology decline in people with AD. | They found that social networks was significantly related to cognition (independent of age, gender, depression, loneliness, and family subset of social network): B = 0·284, P = 0·01. |
| Hsiao et al, 2018^37^ | - | 12 | Review | Increased of the  Amiloid-β. | To review the impact of social behaviors on the progression of cognitive impairment in the animal model of AD. | They found that both humans and mice have a higher risk of developing AD if they are lonely or living isolated. |
| Cui, 2018^49^ | 76 | 13 | Cross-sectional | Alcohol abuse;  Physical exercise; Smoking. | To investigate the impact of behavior habits and emotions on Alzheimer’s disease patients. | They found that main causes of AD onset are alcohol drinking, smoking, lack of exercise, and loneliness. Among these four factors, smoking and anger are the most serious contributing factors. |
| Poey et al, 2017^64^ | 779 | 5 | Cohort | APOE ε4;  Depression. | To investigate whether the social environment moderates the relationship between the APOE ε4 allele and cognitive functioning. | They found that living alone (relative risk ratio [RRR] = 5·814; p < 0·001) and self-reported loneliness (RRR = 1.928, p = 0·049) were associated with a greater risk of cognitive difficulty. Living arrangements, perceived social support, and loneliness were found to moderate the relationship between the APOE ε4 allele and cognitive function. |
| Zhang and Tian, 2017^50^ | 906 | 12 | Cross-sectional | Alcohol abuse;  Anger;  Diet;  Physical exercise; Smoking. | To investigate the prevalence of Alzheimer′s disease (AD) of elderly people in Chongqing, study the effect of behavior and emotion on the incidence of AD of elderly people, and provide reference for the prevention of AD. | They found that smoking, drinking, anger and loneliness are AD risk factors, among which smoking and angry are the independent risk factors. Drinking-induced AD may be associated with hypertension. Exercise and healthy diet are the protective factors of AD. |
| Haj et al, 2016^46^ | 46 | 8 | Case-control | Hallucinations. | To investigate the relationship between social isolation, loneliness, and hallucinations in AD. | They found that AD participants demonstrated higher levels of hallucinations [t(44) = 5.42, p < 0·001)], loneliness [t(44) = 3·76, p < 0·001)], and social isolation (Z = −3·46, p < 0·001) than healthy controls. |
| Boss et al, 2015^33^ | 26,079 | 19 | Review | Depression. | To review the current findings on the association between loneliness and cognitive function in older adults. | They found that loneliness significantly predicted the increased risk for dementia and Alzheimer’s disease at follow-up (OR = 2·56, 95% CI = 1·82–3·61; RR = 1·51, 95% CI = 1·063–2·14). |

Continue on the next page

**Continuing Table 1. Summary of studies included in the review of loneliness and Alzheimer’s Disease**

| **Citation** | **Sample size** | **Quality** | **Design** | **Associated factor** | **Study aim** | **Relevant Result** |
| --- | --- | --- | --- | --- | --- | --- |
| Mushtaq et al, 2014^24^ | - | 11 | Review | Cognitive decline. | To review associated factors with loneliness. | They found that those in the top deciles of loneliness scores were 2.1 times more likely to develop AD than those in the bottom deciles of loneliness scores. |
| Luanaigh et al, 2008^27^ | - | 14 | Review | Cognitive decline. | To review the association between physicians and psychiatrists of the medical impact and biological effects of loneliness as well as making the argument that loneliness should be a legitimate therapeutic target. | They found that loneliness may increase the risk of clinical expression of dementia for the same degree of Alzheimer’s neuropathology. |
| Wilson et al, 2007^72^ | 823 | 8 | Cohort | Depression. | To investigate if loneliness increases the risk to development AD. | They found that risk of AD was more than doubled in lonely persons (score 3·2, 90^th^ percentilie) compared with persons who were not lonely (score 1·4, 10^th^ percentile). |
| Hinton and Levkoff, 1999^34^ | - | 14 | Qualitative | Cognitive decline. | To investigate how family caregivers draw on their cultural and personal resources to create stories about the nature and meaning of illness and to ask how ethnic identity may influence the kinds of stories family caregivers tell. | They found that AD patients could feel lonely because their social cognition functions are decreasing. |
| Zhang et al, 1999^65^ | 1,203 | 5 | Cohort | Bad psychological feeling;  Blue collar occupation;  Lower education;  No gardening in leisure,  No job;  No reading or writing;  No taking community activities. | To investigate the relationship between psychosocial factor and risk of AD. | They found that blue collar occupation, no job, no reading or writing, no taking community activities, no gardening in leisure, bad psychological feeling and lower education were associated with loneliness and AD. |
| Webber et al, 1994^58^ | 2,505 | 4 | Cohort | Poorer;  Women. | To investigate association of live alone and AD. | They found that AD patients who are poorer and women are more susceptible to feeling lonely. |
