# Supplementary material for: Loneliness as an Interface Between Alzheimer's Disease and Suicidal Behaviour: A Systematic Review, Meta‐Analysis and Meta‐Analytic Factor Analysis
Source: Psychogeriatrics. 2026 Apr 7;26(3):e70165. doi: 10.1111/psyg.70165 (PMC13055150; doi:10.1111/psyg.70165)
Supplement: Supplementary file 2 — Table S2: Summary of studies included in the review of loneliness and suicidal behaviour. [file PSYG-26-0-s001.docx]

**Table 2. Summary of studies included in the review of loneliness and suicidal behavior**

| **Citation** | **Sample size** | **Quality** | **Design** | **Associated factor** | **Study aim** | **Relevant Result** |
| --- | --- | --- | --- | --- | --- | --- |
| Hamdan et al, 2025^92^ | 911 | 12 | Cross-sectional | Alcohol addiction;  Depression;  Non-heterosexual;  Social media addiction;  Unmarried. | To investigate whether the increase in suicidal  ideation among college students (mostly young adults) during the pandemic was due to the known risk factors of loneliness, depression, alcohol use disorder, social media addiction, and other background variables. | They found that during the pandemic suicidal ideation was associated with loneliness (χ2 = 54·65, p < 0·001), depressive symptoms (χ2 = 110·82, p < 0·001), alcohol use disorder (χ2 = 10·02, P < 0·01) and social media addiction (χ2 = 13·73, P < 0·001). Being single [OR = 2·55; p < 0·01], and self-identifying as a non-heterosexual [OR = 2·55; p < 0·01] were found to constitute additional risk factors. |
| Wang et al, 2025^283^ | 2,190 | 6 | Cohort | Externalizing and internalizing problems. | To investigate the pathways through which loneliness, internalizing problems, and externalizing problems contribute to suicidal ideation in Chinese adolescents. | They found that loneliness predicted worse subsequent internalizing problems (β = 0·279, p < 0·001) and externalizing problems (β = 0·159, p < .001), which in turn predicted more severe suicidal ideation (β = 0·019, p < 0·001; β = 0·018, p < 0·001). |
| Zuidersma et al, 2025^307^ | 378 | 7 | Cohort | Depression. | To investigate of long-term course over time of suicidal thoughts and ideation in depressed older persons. | They found that suicidal ideation or thoughts of loss of meaning of life were still present in 22·7 % and 17·4 % of those who remitted after 2 and 6 years. |
| Van Orden et al, 2025^28^ | 291 | 5 | Clinical trial | Depression. | To investigate characteristics of suicide risk among older adults who report clinically significant loneliness grounded. | They found that older patients who present with significant loneliness their perceptions of what causes their feelings of loneliness as well as barriers to social engagement. |
| Gao and Fang, 2025^201^ | 618 | 15 | Cross-sectional | Machiavellianism. | To investigate the impact of Machiavellianism on adolescent prosocial behavior and suicide risk, with a specific emphasis on the mediating effects of hope and loneliness. | They found that hope and loneliness emerged as mediators in the relationship between Machiavellianism and both prosocial behavior and suicide risk, with hope functioning as a protective factor and loneliness serving as a risk factor. |
| Hung et al, 2025^202^ | 4,246 | 15 | Cross-sectional | Depression;  Drug abuse. | To investigate the prevalence of loneliness and to investigate the associations between loneliness and suicide. | They found ted that the three most significant factors linked to loneliness were “no one trustworthy to talk to” (OR = 3·69), substance abuse (OR = 3·43), and depression (OR = 3·07), whereas self-rated mental health (poor to good) (OR = 4·11), loneliness (OR = 2·59), and depression (OR = 2·09) were associated with current suicide ideation. |

PTSD: posttraumatic stress disorder; PTSS-SI: posttraumatic stress symptoms and suicide ideation; SA: suicide attempt; SI: suicidal ideation; WTD: wish to die.

**Continue to the next page**

**Continuing Table 2. Summary of studies included in the review of loneliness and suicidal behavior**

| **Citation** | **Sample size** | **Quality** | **Design** | **Associated factor** | **Study aim** | **Relevant Result** |
| --- | --- | --- | --- | --- | --- | --- |
| Iwasawa et al, 2025^93^ | 1,842 | 14 | Cross-sectional | Psychological distress. | To investigate the associations among loneliness, suicidal ideation, and psychological distress in a rural population. | They found that loneliness was strongly associated with suicidal ideation and psychological distress. |
| Lu et al, 2025^330^ | 409,557 | 24 | Review | Bipolar disorders;  Depression. | To review whether Loneliness and suicidality are interrelated phenomena. | They found that in healthy adults aged 18 to 64, loneliness mainly showed a moderate positive correlation with suicidality (r = 0·26 to 0·59), while correlations in older adults (aged 65+) (r = 0·498) and in adolescents aged 13-17 were weaker. |
| Wicher et al, 2025^284^ | 152 | 6 | Cohort | Borderline personality disorder | To investigate whether Loneliness has been linked with suicidal ideation in people with a borderline personality disorder. | They found that the predominance of social interactions was significantly negatively related to daily experiences of loneliness. |
| Koçak et al, 2025^203^ | 1,186 | 15 | Cross-sectional | Online gaming addiction;  Social network addiction. | To investigate the correlations between social network addiction, online gaming addiction, and suicidal ideation, with a focus on the mediating and moderating role of loneliness. | They found that loneliness impacted suicidal ideation and mediated the correlation between network addiction, online gaming addiction, and suicidal ideation. |
| Oh et al, 2025^204^ | 101,744 | 15 | Cross-sectional | Interpersonal abuse. | To investigate potential synergies between loneliness and interpersonal abuse among young adults in higher education. | They found that those who only reported being lonely had significantly greater odds of suicidal ideation (aOR: 3·01; 95% CI: 2·72–3·33), and those who only reported interpersonal abuse also had greater odds (aOR: 2·97; 95% CI: 2·52–3·50). |
| Schafer et al, 2025^94^ | 1,469 | 14 | Cross-sectional | Substance use. | To investigate the link between loneliness, substance use, and suicidality, paying particular attention to the mediational role of loneliness between substance use and suicidality. | They found that loneliness, substance use, and suicidality were significantly and positively related (rs = 0·33–0·42, ps < 0·01). |
| Hou et al, 2024^205^ | 1,465 | 15 | Cross-sectional | Being female; Being only child; Depression;  High high levels of psychotic-like experiences;  Poor sleep quality; PTSD. | To investigate the influencing factors of suicide risk among university students from single parent families. | They found that being female (OR = 1·94, 95% CI: 1·37–2·75), an only child (OR = 1·50, 95% CI: 1·05–2·16), having depression (OR = 2·20, 95% CI: 1·45–3·35), high psychotic-like experiences (OR = 2·78, 95% CI: 1·82–4·23), poor sleep quality (OR = 1·95, 95% CI: 1·32–2·89), and greater loneliness (OR = 1·15, 95% CI: 1·09–1·20) were associated with a higher likelihood of suicide risk, whereas being a graduate student (OR = 0·43, 95% CI: 0·29–0·65) served as a protective factor. |

**Continue to the next page**

**Continuing Table 2. Summary of studies included in the review of loneliness and suicidal behavior**

| **Citation** | **Sample size** | **Quality** | **Design** | **Associated factor** | **Study aim** | **Relevant Result** |
| --- | --- | --- | --- | --- | --- | --- |
| Shi et al,2024^206^ | 208 | 16 | Cross-sectional | Age;  Incidence;  Course of disease; Cumulative number of years of hospital stay. | To investigate the correlation between loneliness and passive suicidal ideation in patients with stable schizophrenia | They found that passive suicidal ideation was related to disease incidence (t = –2·434, P < 0·05), course (F = 18·821, P < 0·001), and cumulative hospital stay (F = 13·776, P < 0·001). Age (r = 0·322, P < 0·001) and loneliness (r = 0·565, P < 0·001) were positively correlated, and both cumulative hospital stay (β = 1·620, P < 0·05) and loneliness (β = 0·408, P < 0·001) independently predicted suicidal ideation. |
| Torres et al, 2024^285^ | 50,423 | 5 | Cohort | Depression;  Mobility limitations. | To investigate longitudinal evidence for the longitudinal relationship between social participation, loneliness, and mobility limitations and suicidal ideation in a representative sample of older adults. | They found highlight the importance of promoting mobility  programs and social activities to prevent suicidal ideation among older adults. |
| Heo et al, 2024^95^ | 1,770 | 14 | Cross-sectional | Unemployed;  Unmarried. | To investigate the impact of qualitative and quantitative loneliness on suicidal ideation in a working-age population by employed, self-employed, or unemployed. | They found that unemployed group had the highest loneliness score (mean=44·9) and the highest likelihood of having high levels of loneliness (31%). |
| Altin et al, 2024^207^ | 332 | 15 | Cross-sectional | Anxiety;  Depression;  Men;  Pornography. | To investigate the associations between suicidality and problematic pornography use in a sample of young adults, taking into account gender differences and the role of loneliness, emotional states, and well-being. | They found showed a positive link between pornography use and anxiety, depression, stress, loneliness, and suicidal ideation, and a negative correlation with well-being. |
| Ward et al, 2024^96^ | 8,175 | 14 | Cross-sectional | Depression;  Never attended church;  Unmarried. | To investigate the association between social disconnection and a WTD among a large nationally representative cohort of community-dwelling older adults. | They found that loneliness was the strongest risk factor to WTD while attendance of religious services was an important protective behaviour. |
| Meore et al, 2024^313^ | 83 | 2 | Clinical trial (Pilot study) | Depression. | To investigate the initial feasibility and acceptability of horticultural therapy when virtually administered. | They found that stress, pain, depression, and loneliness indices showed small to medium sized symptom reduction amongst Veterans with no history of suicidality (Cohen’s d=− 0·70, d=− 0·49, d=− 0·62, d=− 0·71), while those with elevated suicide risk at baseline also showed  reduction in these risk factors with small to medium effect sizes (d=− 0·58, d=− 0·018, d=− 0·46, d=− 0·41). |

**Continue to the next page**

**Continuing Table 2. Summary of studies included in the review of loneliness and suicidal behavior**

| **Citation** | **Sample size** | **Quality** | **Design** | **Associated factor** | **Study aim** | **Relevant Result** |
| --- | --- | --- | --- | --- | --- | --- |
| Smith et al, 2024^97^ | 8,163 | 14 | Cross-sectional | Anxiety;  Depression;  Lower levels of education;  Sedentary;  Sleep disorders;  Unemployed;  Unmarried;  Women. | To investigate association between sedentary behavior and WTD. | They found that persons with WTD (vs. no WTD) were significantly more likely to engage in ≥8 h/day of sedentary behavior, and be females, separated/divorced/  widowed, unemployed, and have lower levels of education and physical activity, greater number of chronic conditions, and disability, while they were also slightly younger. Furthermore, they were more likely to have depression, anxiety, as well as more sleep problems, loneliness, and perceived stress, while they also had fewer social networks. |
| Levi-Belz, et al 2024^274^ | 710 | 3 | Cohort | Depression;  PTSD. | To investigate the extent to which the experience of loneliness moderates the PTSS-SI association in the wake of the October 7th terrorist attack. | They found that clinicians treating individuals coping with high PTSS levels should attend to their patients’ sense of loneliness, as it comprises a significant risk factor for current SI and may be considered an important target in treatment. |
| Li et al, 2024^208^ | 145,043 | 16 | Cross-sectional | Childhood trauma. | To investigate the protect and risk factors of social health, self-injurious thoughts and behaviors between adults with or without childhood trauma. | They found risk effect of loneliness for suicide ideation and suicide attempt were weaker in participants experiencing childhood trauma (p<0·05). |
| Alacreu-Crespo et al, 2024 ^325^ | - | 12 | Review | Cardiovascular disease;  Mental disorders;  Sympathetic nervous system. | To review the relationship of autonomic nervous system activity with social environment and suicidal spectrum behaviors. | They found that Patients with suicidal ideation/suicide attempt have higher sympathetic nervous system and lower parasympathetic nervous system activity in resting conditions and during acute stress tasks compared with patients without suicidal ideation/suicide attempt. Death by suicide and violent suicide attempt are related to sympathetic nervous system hyperactivation. |
| Larson et al, 2024^88^ | 1,134 | 8 | Cross-sectional | Barriers to treatment; Depression;  Disability;  Financial insecurity;  Housing instability. | To investigate the social determinants of health associated with suicidality among female. | They found that most likely to report suicidality, are those experiencing disability (p<0·01), poor physical health (p<0·01), homelessness (p<0·001), barriers to mental health treatment (p<0·001), housing instability (p<0·001), financial insecurity (p<0·001), food insecurity (p<0·001), and feelings of loneliness or isolation (p<0·001). |
| Corcoran et al, 2024^334^ | - | 30 | Review | Terminal illness. | To review associated factor with euthanasia or suicide assisted request. | They found no evidence to support an association between different constructs of social connectedness and requested or actual euthanasia or suicide assisted. |

**Continue to the next page**

**Continuing Table 2. Summary of studies included in the review of loneliness and suicidal behavior**

| **Citation** | **Sample size** | **Quality** | **Design** | **Associated factor** | **Study aim** | **Relevant Result** |
| --- | --- | --- | --- | --- | --- | --- |
| Alothman et al, 2024^43^ | 594,674 | 5 | Case-control | Younger. | To investigate the association between loneliness and living alone and suicide mortality. | They found risk of suicide in individuals who had previously been reported to be either living alone or suffering loneliness was increased (OR 4·9; 95 % CI: 4·4 to 5·5). |
| Bentum et al, 2024^286^ | 1,164 | 5 | Cohort | Autism. | To investigate the occurrence of various potential risk factors for lifetime suicidal behavior and suicidal thoughts in the past month of autistic individuals. | They found that linear regression analyses identified the following predictors for suicidal behavior: psychiatric comorbidity, loneliness, and higher number of autistic traits, F(3, 240)=21·22, p<0·001, with R2 of 0·21. |
| Hung et al, 2024^209^ | 8,460 | 15 | Cross-sectional | COVID-19. | To investigate the impact of COVID-19 pandemic on the prevalence of loneliness and its associating suicide risks. | They found odds of loneliness for lifetime suicidal ideation, lifetime suicide attempt, and future suicide intent were 4·9, 5·1, and 9·2, respectively. During the COVID-19 period, loneliness and suicidality demonstrated a noteworthy decline trend, whereas “no one trustworthy to talk to” was the only item that showed significant increase under the pandemic and impacted on loneliness. |
| Zerach et al, 2024^275^ | 374 | 4 | Cohort | Depression. | To investigate the association between depression, loneliness and suicidal behavior. | They found association between loneliness, depression and suicidal ideation. |
| Canter et al, 2024^98^ | 88,986 | 13 | Cross-sectional | Distress. | To investigate whether psychological flourishing, a multi-dimensional construct of well-being, has the potential to play a preventative role in suicidal and nonsuicidal thoughts and actions. | They found that effect of distress on suicidal ideation was especially pronounced at high levels of loneliness. |
| Mejova and Hommadova, 2024^30^ | 147,734 | 18 | Qualitative | COVID-19;  Depresion;  LGBTQ+. | To investigate the quantity and quality of interaction the LGBTQ+ community has experienced on social media, specifically around the topic of loneliness. | They found that compared to the year before COVID-19, these users posted 67% more loneliness self-disclosures during the first year of COVID-19. Examining the emotional content of these tweets, they found frequent references to depression, dysphoria, and suicidal ideation. |
| Keiner et al, 2024^210^ | 2,016 | 16 | Cross-sectional | Burnout;  Depression;  Women. | To investigate the 1) how often medical students, residents, fellows, and faculty physicians who completed a suicide screening questionnaire at a large US academic medical center endorsed intense loneliness and 2) the  relationships of loneliness with other negative mental health states. | They found that greater odds of endorsing intense loneliness was seen in those under age 40 (OR=0·35,  p<0·001), women (OR=1·30, p=0·030), and non-Whites (OR=1·70, p<0·001); and in those with burnout (OR=3.14, p<0.001), depression (OR=12·34, p<0·001), other intense affective states (OR=4·34–8·34, p<0·05), and suicidal ideation (OR=3·47–13·00, p<0·001). |

**Continue on the next page**

**Continuing Table 2. Summary of studies included in the review of loneliness and suicidal behavior**

| **Citation** | **Sample size** | **Quality** | **Design** | **Associated factor** | **Study aim** | **Relevant Result** |
| --- | --- | --- | --- | --- | --- | --- |
| Pike et al, 2024^287^ | 146 | 5 | Cohort | Bipolar disorder. | To investigate how perceived social connectedness relates to suicidal thoughts and behaviors in Bipolar Disorder. | They found that Bipolar disorder participants reported significantly higher loneliness and lower social support than healthy controls. |
| Spoljar, 2024^326^ | - | 12 | Review | Schizophrenia. | To review the inhabitation of a place and the constitution of a territorial Self,’ in the context of  ‘country’ life and work. | They found that in schizophrenia, there is no place where a path can be traced, because tracing a path requires being both here and where one will be. |
| Ebulum et al, 2024^99^ | 500 | 14 | Cross-sectional | Low religiosity;  Stress. | To investigate whether loneliness, stress and religiosity would be associated with suicide ideation. | They found that whereas suicidal ideation could be increased by loneliness and stress, religious older adults  were less likely to report suicidal ideation. |
| Saulnier et al, 2024^314^ | 455 | 7 | Hybrid Trial | Social distress. | To investigate whether social support and social distress measures have general factors versus measure-specific factors that are associated with suicide risk. | They found that suicidal ideation was significantly associated with the general social support (B=−1·51),the general social distress (B=1·67), and the specific perceived burdensomeness (B=1·57) factors. Suicide attempts were significantly associated with the specific Perceived Rejection (OR=1·05) and Thwarted Belongingness (OR=0·91) factors. |
| Hamdan-Mansour et al, 2024^100^ | 403 | 14 | Cross-sectional | Academic procrastination;  Anxiety | To investigate the prediction power of loneliness, academic anxiety, and academic procrastination on suicidality among university students. | They found that being a working student, being on psychotropic medication, romantic and family relationships domains of loneliness, and academic anxiety were found to be predictors of suicidality (p<0·05). |
| Klugman et al, 2024^211^ | 337 | 15 | Cross-sectional | Burnout;  Depression;  Perfectionism. | To investigate the most significant risk factors contributing to suicidal risk and to propose a mediation model that explores the direct and indirect effects of being a medical student on suicidal risk. | They found that medical student status, depressive symptoms, and loneliness as significant predictors of suicide risk. Mediation analysis revealed loneliness and depressive symptoms mediating the relationship between medical student status and suicide risk. |
| Kwon and Jang, 2024^212^ | 1,393 | 17 | Cross-sectional | Sedentary lifestyle. | To investigate the association between the number of school sports teams that a student regularly participates in and psychological factors such as perceived stress, loneliness, and sleep satisfaction. | They found that that school sports are an advantageous tool for suicide prevention. |

**Continue on the next page**

**Continuing Table 2. Summary of studies included in the review of loneliness and suicidal behavior**

| **Citation** | **Sample size** | **Quality** | **Design** | **Associated factor** | **Study aim** | **Relevant Result** |
| --- | --- | --- | --- | --- | --- | --- |
| Wolf et al, 2024^272^ | 20 | 2 | Cohort | Depression. | To investigate the association between suicidal ideations and behaviors with loneliness in persistent depressive disorder. | They found that suicidal ideations and loneliness varied with one standard deviation over three to six hours. |
| Lin et al, 2024^101^ | 400 | 14 | Cross-sectional | Sexual stigma. | To investigate the mediating effect of loneliness on the associations of perceived sexual stigma and internalized sexual stigma with suicide in gay and bisexual men. | They found that both perceived sexual stigma and internalized sexual stigma were positively associated with suicide through the full  mediation of loneliness. |
| Yöyen and Keleş, 2024^213^ | 305 | 16 | Cross-sectional | Anger;  Impulsivity. | To investigate the psychological variables that discriminate between individuals who attempt suicide and those who only have suicidal ideation. | They found that anger/impulsivity (Wald = 4·827; p < 0·05), perceived burden on others (Wald = 8·613; p < 0·05), acquired suicide efficacy/death fearlessness (Wald = 13.377; p < 0v001), being female (Wald = 3·925; p < 0.05), presence of diagnosed psychiatric illness in the family (Wald = 5·705; p < 0·05), and receiving psychological support (Wald = 4·381. p < 0·05) variables are significant predictors of the transition from suicidal ideation to suicidal action. |
| Kumar et al, 2024^31^ | - | 25 | Review | Dependence. | To review the associated factors of suicidal ideation and suicide attempts in persons with  physical disabilities. | They found that associated factors of suicidal behavior were disability status, burdensomeness, felt stigma, depressive symptoms, loneliness, lack of social and emotional connectedness, long-term physical or mental disability, congenital disability, and aggression. |
| Wang et al, 2024^214^ | 2,343 | 15 | Cross-sectional | Depression. | To investigate the varying relative importance of different risk factors for suicidal ideation and suicide attempts to distinctively offer empirical support in clinical practice. | They found that the most significant risk factor in both groups was depression. However, factors such as academic stress, hopelessness, and age were more closely associated with suicidal ideation than suicide attempts. Factors related to the schooling status, total years of education, and loneliness were relatively more important in the suicide attempt stage compared to suicidal ideation. |

**Continue on the next page**

**Continuing Table 2. Summary of studies included in the review of loneliness and suicidal behavior**

| **Citation** | **Sample size** | **Quality** | **Design** | **Associated factor** | **Study aim** | **Relevant Result** |
| --- | --- | --- | --- | --- | --- | --- |
| Jung et al, 2024^276^ | 157,432 | 3 | Cohort | Financial stress. | To investigate explored the trend of suicide attempts and the association between loneliness, family financial stress, and suicide attempts during the COVID-19 pandemic among adolescents. | They found that risk of suicide attempt was higher among adolescents who experienced financial stress (in 2020: adjusted odds ratio [AOR], 1·53, 95% confidence interval [CI], 1·26–1·88; in 2021: AOR, 1·63, 95% CI, 1·03–1·54) and felt lonely (in 2020: AOR, 2·19, 95% CI, 1·78–2·70; in 2021: AOR, 2·65, 95% CI, 2·16–3·26; in 2022: AOR, 1·3, 95% CI, 1·04–1·55) than those who did not. |
| Cabrera-Mendoza et al, 2024^40^ | 1,062,722 | 16 | Mendelian randomization | Bipolar disorder;  Schizophrenia. | To investigate causal relationships between bipolar disorder, schizophrenia, and suicide attempt with behavioral traits, socioeconomic factors, and substance use disorders utilizing large-scale genome-wide association data. | They found that genetic liabilities to bipolar disorder and schizophrenia were associated with higher odds of suicide attempt (BD odds ratio (OR)=1·24, p=3·88x10-12; SZ OR=1·09, p=2·44x10-20). |
| Mizuno and Cho, 2023^327^ | 857 | 11 | Review | Poverty; Unemployment. | To review the issues of suicide and isolation and loneliness in the Covid-19 Disaster. | They found results of a questionnaire survey of 857 mental health care providers showed that the number one reason for suicidal ideation and attempts was isolation and loneliness (75%), followed by economic deprivation (70%) and unemployment (55%). Since multiple responses were given, no single factor is known. |
| Zhao et al, 2023^102^ | 1,411 | 14 | Cross-sectional | High level of connectedness to nature; Interpersonal sensitivity. | To investigate the influence and mechanism of interpersonal sensitivity on suicidal ideation in college students, and explore the mediating role of loneliness and the moderating role of connectedness to nature. | They found that interpersonal sensitivity predicted suicidal ideation (β = 0·43, P < 0·001) and loneliness (β = 0·49, P < 0·001). After adding loneliness, both interpersonal sensitivity (β = 0·29, P < 0v001) and loneliness (β = 0.28, P < 0·001) remained significant, indicating partial mediation. Connectedness to nature moderated the effect of loneliness on suicidal ideation (interaction β = −0·14, P < 0·01): the effect was stronger at low connectedness (β = 0·41, P < 0·001) than high connectedness (β = 0·14, P < 0·05). |

**Continue on the next page**

**Continuing Table 2. Summary of studies included in the review of loneliness and suicidal behavior**

| **Citation** | **Sample size** | **Quality** | **Design** | **Associated factor** | **Study aim** | **Relevant Result** |
| --- | --- | --- | --- | --- | --- | --- |
| Zhai et al,2023^215^ | 532 | 15 | Cross-sectional | Self-construal; Social support. | To investigate the effects of self-construal on suicidal ideation and its mechanism in the officers and soldiers of a naval unit. | They found that among 532 naval personnel, 1·69% reported suicidal ideation. Suicidal ideation was positively correlated with loneliness and negatively correlated with perceived social support, independent self-construal, and interdependent self-construal (all P < 0·01). Individuals with marginal self-construal (both self-construal types below average) showed the highest suicidal ideation. Mediation analysis showed that perceived social support and loneliness fully mediated the association between interdependent self-construal and suicidal ideation (indirect effect 95% CI: −0·303 to −0·208, P < 0·01), and loneliness partially mediated the association between perceived social support and suicidal ideation (indirect effect 95% CI: −0·231 to −0·168, P < 0·01). |
| Liu et al,2023^216^ | 1,324 | 15 | Cross-sectional | Low perceived campus climate;  Peer rejection. | To investigate the association between peer rejection and suicidal behavior among adolescents, and to test whether loneliness mediates this relationship and whether perceived campus climate moderates both the direct effect and the mediating effect. | They found peer rejection predicted suicidal behavior (B = 0.14, p < 0·001). With loneliness included, peer rejection predicted loneliness (B = 0·56, p < 0·001), and loneliness predicted suicidal behavior (B = 0·21, p < 0·001), with a significant indirect effect (0·12, 95% CI [0·09, 0·15]). The interaction between loneliness and perceived campus climate was significant (B = −0·10, p < 0·001). When campus climate was low (M −1SD), loneliness predicted suicidal behavior (B = 0·27, p < 0·001); when high (M +1SD), the effect was nonsignificant (B = 0·06, p = 0·146). The indirect effect via loneliness was significant only when campus climate was low (0·15, 95% CI [0.11, 0·19]) but not high (0·03, 95% CI [−0.01, 0.08]), with a significant difference (Δ = −0·12, 95% CI [−0·16, −0·07]). |
| Chen, Jian et al, 2023^103^ | 300 | 13 | Cross-sectional | Depression;  Low self-esteem;  Schizophrenia. | To investigate the moderating effects of self-esteem and perceived support from families and friends on the association of loneliness with suicide risk and depression in individuals with schizophrenia. | They found that self-esteem was significantly associated with a reduced magnitude of depression in participants with loneliness. |

Continue to the next page

**Continuing Table 2. Summary of studies included in the review of loneliness and suicidal behavior**

| **Citation** | **Sample size** | **Quality** | **Design** | **Associated factor** | **Study aim** | **Relevant Result** |
| --- | --- | --- | --- | --- | --- | --- |
| Peprah et al, 2023^217^ | 78,558 | 16 | Cross-sectional | Bullying. | To investigate the association between bullying and suicidal behavior among in-school adolescents. | They found that bullying was associated with a 44% increased risk of suicidal behavior after adjusting for potential confounders (relative risk = 1·44; 95% confidence interval = 1·39-1·48). Loneliness partially mediated the association between bullying and suicidal behavior, and parental involvement moderated the association. |
| McGillivray et al, 2023^316^ | 455 | 9 | Clinical Trial | Depression. | To investigate whether loneliness affects how young people experiencing suicidal ideation engage with and benefit from a therapeutic smartphone intervention. | They found that loneliness was positively associated with higher levels of overall suicidal ideation (B=0·75, 95% CI 0·08-1·42; P=0.03) and depression (B=0·88, 95% CI 0·45-1·32; P<0·001). |
| Chen, Wang et al, 2023^218^ | 14,726 | 16 | Cross-sectional | Emotional and behavioral problems. | To investigate the network structure and differences in emotional and behavioral problems, loneliness, and suicidal thoughts in adolescents during different pandemic periods. | They found that suicidal thoughts showed the closest connections with being unhappy and lonely. |
| Yang et al, 2023^219^ | 941 | 15 | Cross-sectional | Anxiety;  Depression. | To investigate the association between loneliness and symptoms of anxiety and depression among university students. | They found that “Suicide” was directly connected to five symptoms of depression and four items of loneliness, with the strongest connections being between it and “Feeling of worthlessness” and “Psychomotor agitation/retardation”. |
| Akram et al, 2023^89^ | 1,408 | 6 | Cross-sectional | Depression;  Insomnia. | To investigate of the prevalence of psychiatric symptoms by using well validated scales with robust psychometric properties. | They found association between loneliness, depression, insomnia and suicidal ideation. |
| Lee et al, 2023^104^ | 5,511 | 14 | Cross-sectional | Lower educational;  Older age;  Non-full-time employment;  Widowed, separated, or divorced status. | To investigate the association between loneliness and suicidality in the general population. | They found that individuals with loneliness were significantly associated with increased suicidal ideation (adjusted odd ratio [aOR], 4·05; 95% CI 3·36–4·88), suicidal plans (aOR, 4·91; 95% CI 3·34–7·21), and suicidal attempts (aOR, 4·82; 95% CI 3·03–7·66). |

Continue to the next page

**Continuing Table 2. Summary of studies included in the review of loneliness and suicidal behavior**

| **Citation** | **Sample size** | **Quality** | **Design** | **Associated factor** | **Study aim** | **Relevant Result** |
| --- | --- | --- | --- | --- | --- | --- |
| Matsushima et al, 2023^220^ | 768 | 16 | Cross-sectional | Delay childbearing. | To investigate how pregnancy decisions affect the well-being of women during the COVID-19 pandemic. | They found that loneliness and suicidal ideation that occurred after the beginning of the pandemic were significantly related to the decision to delay childbearing—1·55 [95% CI (1·03,2·34)] and 2·55 [95% CI (1·45–4·51)], respectively. |
| Wei et al, 2023^105^ | 1,338 | 14 | Cross-sectional | Anxiety;  Depression. | To investigate the relationship between loneliness and mental disorders among medical residents. | They found that loneliness increased the risk of major depression, major anxiety, and suicidal ideation (all p<0·001). |
| Shoib et al, 2023^42^ | 21,947 | 18 | Review | Economic hardship;  Mental disorder;  Substance use disorder. | To review the association between loneliness and suicidal behavior. | They found that association between loneliness and suicidal behavior is determined by individual, social and cultural factors. Co-existing mental illness, substance use disorder and economic hardship play an important role for the completion of suicide. |
| Tachikawa et al, 2023^221^ | 26,000 | 16 | Cross-sectional | Depression. | To investigate how loneliness relates to suicidal ideation following the onset of the COVID-19 pandemic. | They found that the Poisson regression suggested that those who were feeling lonely had higher PRs for suicidal ideation [4·83 for men (95%CI, 3·87 to 6·16) and 6·19 for women (95%CI, 4·77 to 8·45)]. |
| Solomonov et al, 2023^106^ | 16,164 | 14 | Cross-sectional | Lack of social support. | To investigate the prevalence of social disconnection and thoughts of suicide among older adults. | They found that households with 3 or more additional members (adjusted OR 1·73, 95% CI 1·28–2·33) and lack of social supports, particularly emotional supports (adjusted OR 2v60, 95% CI 2·09 – 3v23), were independently associated with greater likelihood of reporting such thoughts, as was greater reported loneliness (adjusted OR 1·75, 95% CI 1·64 – 1v87). |
| Bessaha et al, 2023^107^ | 307 | 14 | Cross-sectional | Higher levels of psychological distress. | To investigate whether loneliness and social support are associated with mental health services use and mental health symptoms (psychological distress and suicidal ideation) among emerging adults. | They found that participants with higher levels of loneliness had over four times the odds of past year suicidal ideation (OR = 4·61, 95% CI [2·46, 8·63]). |
| Ogrodniczuk et al, 2023^222^ | 434 | 16 | Cross-sectional | Thwarted belongingness | To investigate the impact of loneliness on suicidal ideation among men and whether thwarted belongingness mediated this relationship. | They found a significant association between loneliness and suicidal ideation that was mediated by thwarted belongingness. |

Continue to the next page

**Continuing Table 2. Summary of studies included in the review of loneliness and suicidal behavior**

| **Citation** | **Sample size** | **Quality** | **Design** | **Associated factor** | **Study aim** | **Relevant Result** |
| --- | --- | --- | --- | --- | --- | --- |
| Blázquez-Fernández et al, 2023^331^ | - | 22 | Review | Aggression;  Anxiety;  Bullying;  Drug/alcohol use;  Food insecurity;  Lack of parental understanding. | To review the association between ‘isolation’ and suicides. | They found that suicide and -social isolation and loneliness- have a positive and direct relationship although these findings varied slightly by areas. |
| McClelland et al, 2023^223^ | 582 | 16 | Cross-sectional | Depression. | To investigate how different forms of loneliness might be associated with self-injury, based on findings from existing theory-driven research. | They found that the quality and/or quantity of family, romantic and global relationships, should be explored when considering loneliness as a possible risk factor for  suicidal ideation and may have a significant impact on mental and physical health. |
| Aran et al, 2023^32^ | - | 21 | Review | Low familial support. | To review the relationship between social connection and suicide among newcomers, immigrants, and asylum seekers. | They found that social disconnection as an important determinant of mental health and suicide risk among immigrant populations. |
| Lewis et al, 2023^224^ | 184 | 15 | Cross-sectional | Anxiety;  COVID-19. | To investigate the role of anxious and avoidant attachment, loneliness, and frequency of social contact as prospective predictors of suicidal ideation during the first 6months of the COVID-19 pandemic using a relational diathesis-stress framework. | They found that both trait loneliness and anxious attachment predicted the prospective development of suicidal ideation during the first 6months of the COVID-19 pandemic. |
| Suárez-Relinque et al, 2023^225^ | 1,982 | 17 | Cross-sectional | Alexithymia;  Child-to-parent violence. | To investigate the relationship between involvement in child-to-parent violence and the development of emotional loneliness, suicidal ideation, and alexithymia based on sex. | They found that the adolescents with higher child-to-parent violence scored higher in emotional loneliness, suicidal ideation, and alexithymia. |
| Liang et al, 2023^308^ | 643 | 8 | Cohort | Anxiety;  Depression. | To investigate the joint trajectories of loneliness, depressive symptoms, and social anxiety from middle childhood to early adolescence and their associations with suicidal ideation. | They found the importance of individual differences considerations in understanding the joint patterns of loneliness, depressive symptoms, and social anxiety among youth during the transition into adolescence and the need for more sophisticated intervention programs tailored to the unique characteristics of the relevant trajectories to reduce the risk of suicidal ideation. |

Continue to the next page

**Continuing Table 2. Summary of studies included in the review of loneliness and suicidal behavior**

| **Citation** | **Sample size** | **Quality** | **Design** | **Associated factor** | **Study aim** | **Relevant Result** |
| --- | --- | --- | --- | --- | --- | --- |
| Shawon, Rouf et al, 2023^108^ | 222,401 | 13 | Cross-sectional | Anxiety. | To investigate the sex-specific prevalence of psychological distress and unhealthy eating habits among adolescents across countries and regions, and to explore their potential associations. | They found that the combined prevalence for loneliness, anxiety, suicide ideation, suicide planning, and suicide attempt was 12·1%, 10·7%, 15·3%, 15·0%, and 12·9%, respectively. |
| Vally and Helmy, 2023^109^ | 5,826 | 14 | Cross-sectional | Anxiety. | To investigate examine the prevalence of suicidal ideation, the formulation of a suicide plan, and suicide attempts amongst a sample of school-going adolescents. | They found that elevated risk was significantly associated with anxiety difficulties, the experience of loneliness, and amongst those who smoked tobacco. |
| Christensen et al, 2023^335^ | 177 | 26 | Review | Low family support. | To review qualitative studies examining older Asian people’s experiences of suicidal ideation. | They found that experiences of older Asian people varied from feelings of loneliness, despair, and isolation to wanting to live a fruitful life into old age. |
| Mahmoud et al, 2023^110^ | 800 | 11 | Cross-sectional | Psychological distress. | To investigate the influence of social media addiction, psychological distress, and loneliness on suicidal ideations and suicide attempts among healthcare students and professionals. | They found showed that lifetime suicidal thoughts were predicted by psychological distress, loneliness, and age. |
| Shin and Kim, 2023^226^ | 14,724 | 15 | Cross-sectional | Anxiety. | To investigate a data-based analysis method for predicting suicidal thoughts quickly and effectively and suggests countermeasures against the causes of suicidal thoughts. | They found showed that sadness and depression increased suicidal thoughts by more than 25 times, and anxiety, loneliness, and experience of abusive language increased suicidal thoughts by more than three times. |
| Sarfo et al, 2023^227^ | 1,864 | 15 | Cross-sectional | Bulling;  Tabaco. | To investigate the prevalence and associated factors of suicidal behavior among students. | They found that suicidal ideation was predicted by being physically attacked and bullied, parental guidance, tobacco use, loneliness, and worry. |
| Diallo et al, 2023^228^ | 9,726 | 15 | Cross-sectional | Bulling. | To investigate suicidality among West African adolescents. | They found that significant correlates of suicide attempt included older age (16+ years; odds ratio [OR]: 1·70, confidence interval [CI]: 1·09-2·63), difficulty sleeping due to worry (OR: 1·27, CI: 1·04-1·56), loneliness (OR: 1·65, CI: 1·39-1·96), truancy (OR: 1·38. CI: 1·05-1·82), being a target of bullying (OR: 1·53, CI: 1·26-1·85), getting physically attacked (OR: 1·73, CI: 1·42-2·11), physical fighting (OR: 1·47, CI: 1·21-1·79), current cigarette use (OR: 2·71, CI: 1·88-3·89), and initiation of drug use (OR: 2·19, CI: 1·71-2·81). |

Continue to the next page

**Continuing Table 2. Summary of studies included in the review of loneliness and suicidal behavior**

| **Citation** | **Sample size** | **Quality** | **Design** | **Associated factor** | **Study aim** | **Relevant Result** |
| --- | --- | --- | --- | --- | --- | --- |
| Fischer, Nichter, Aunon et al, 2023^111^ | 4,069 | 13 | Cross-sectional | Lack of meaning in life. | To investigate the prevalence and correlates associated with suicidal thoughts and behaviors. | They found that higher levels of loneliness and lower levels of purpose in life were most strongly associated with past year suicidal ideation; lifetime history of major depressive disorder with suicide plan and suicide attempt. |
| Kim et al, 2023^112^ | 60 | 14 | Cross-sectional | Feeling of rejection. | To investigate subtypes of nonsuicidal self-injury based on the forms of self-harm behavior. | They found that anger toward others, feeling of rejection, loneliness, and helplessness were significantly high in the substance abuse and suicide attempt subtype. |
| Fischer, Nichter, Na et al, 2023^288^ | 2,441 | 6 | Cohort | COVID-19 | To investigate longitudinal trends in suicidal thoughts and behaviors. | They found that factors strongly associated with new-onset suicidal ideation included higher education (odds ratio [OR], 3·27; 95% CI, 1·95-5·46), lifetime substance use disorder (OR, 2·07; 95% CI, 1·23-3·46), prepandemic loneliness (OR, 1·28; 95% CI, 1·09-1·49), and lower prepandemic purpose in life (OR, 0·92; 95% CI, 0·86-0·97). |
| Sultana et al, 2023^113^ | 42,888 | 13 | Cross-sectional | Bulling;  Low parental support. | To investigate estimate the prevalence of suicidal ideation, suicidal plan, and suicide attempt among adolescents with a focus on parental and peer support. | They found that feeling lonely was association with suicidal behavior [aOR: suicidal ideation-3·41(2·60,4·46), suicidal plan-1·92 (1·48,2·47), suicide attempt-2·25 (1·62,3·13)]. |
| Quarshie et al, 2023^229^ | 3,152 | 15 | Cross-sectional | Bulling. | To investigate the 12-month prevalence and describe some of the associated factors of suicide behavior among school-going adolescents aged 12–17 years old. | They found that physical attack victimization, bullying victimization, loneliness, and parental intrusion of privacy as key factors associated with increased likelihood of suicidal ideation, planning, one-time suicide attempt, and repeated attempted suicide. |
| Zhu et al, 2023^114^ | 541 | 14 | Cross-sectional | Bulling;  Depression. | To investigate the similarities and differences in risk and protective factors of children and adolescent suicidality. | They found that common correlates for suicidal ideation were depression, bullying, loneliness, self-compassion, and growth mindset, while those for suicide attempt were depression and bullying. |
| Motsa et al, 2023^115^ | 3,680 | 14 | Cross-sectional | Anxiety;  Bulling;  Food insecurity. | To investigate the potential mediating roles of anxiety and loneliness on the association of concurrent food insecurity and being bullied with suicidal behavior. | They found relationship between food insecurity and being bullied with suicidal behavior was partly (approximately 24%) mediated by anxiety and loneliness. |

Continue to the next page

**Continuing Table 2. Summary of studies included in the review of loneliness and suicidal behavior**

| **Citation** | **Sample size** | **Quality** | **Design** | **Associated factor** | **Study aim** | **Relevant Result** |
| --- | --- | --- | --- | --- | --- | --- |
| Schwartz-Mette et al, 2023^289^ | 362 | 6 | Cohort | Depression. | To investigate whether COVID-19-related loneliness and health anxiety (assessed in March 2020) predicted increased depressive symptoms, frequency of non-suicidal self-injury (NSSI), and suicide risk from pre-pandemic (late January/early February 2020) to June 2020. | They found that loneliness predicted higher depressive symptoms for all adolescents, non-suicidal self-injury frequency for adolescents with low pre-pandemic frequency (but less frequent non-suicidal self-injury for adolescents with high pre-pandemic frequency). |
| Shawon, Jahan et al, 2023^116^ | 30,013 | 14 | Cross-sectional | Psychological distress. | To investigate the association between psychological distress and unhealthy dietary behaviors among adolescents. | They found that psychological distress was associated with inadequate fruit intake (pooled OR = 1·20, 95 % CI 1·03, 1·40), inadequate vegetable intake (pooled OR = 1·17, 1·05, 1·31) and daily soft drink consumption (pooled OR = 1·14, 1·03, 1·26); but not with weekly fast-food consumption (pooled OR = 1·13, 0·96, 1·31). |
| Zhou et al, 2023^230^ | 393 | 15 | Cross-sectional | Depression;  Low school interaction. | To investigate examined loneliness and school belonging as predictors of suicide risk (viz., suicidal behavior and depression) in college students. | They found a significant loneliness × school belonging interaction as a predictor of both suicidal behavior and depression was found. |
| Yin et al,2022^231^ | 205 | 15 | Cross-sectional | Quality of life; Social support. | To investigate the relationship between loneliness and suicidal ideation of rural nursing home residents through the mediating effect of quality of life, and the moderating effect of social support, respectively. | They found that loneliness was negatively correlated with quality of life (r = −0·652) and that suicidal ideation was negatively correlated with quality of life (r = −0·638). Quality of life mediated the effect of loneliness on suicidal ideation (effect = 0·309, 95% CI 0·205–0·422), and social support moderated both the loneliness→quality of life (B = −0·113) and quality of life→suicidal ideation paths (B = 0·238). |
| Niu et al,2022^117^ | 400 | 11 | Cross-sectional | Authenticity; Experience avoidance. | To investigate the impact of authenticity on suicide idea and its mechanism. | They found that authenticity was negatively correlated with loneliness, suicidal ideation, and experiential avoidance (r = −0·463, −0·314, −0·537), while loneliness and experiential avoidance were positively correlated with suicidal ideation (r = 0·302, 0·434). Experiential avoidance fully mediated the effect of authenticity on suicidal ideation (95% CI −0·18 to −0·08), and loneliness together with experiential avoidance formed an additional mediating pathway (95% CI −0·09 to −0·03). |

Continue to the next page

**Continuing Table 2. Summary of studies included in the review of loneliness and suicidal behavior**

| **Citation** | **Sample size** | **Quality** | **Design** | **Associated factor** | **Study aim** | **Relevant Result** |
| --- | --- | --- | --- | --- | --- | --- |
| Raifman et al, 2022^118^ | 6,500 | 13 | Cross-sectional | Job less. | To investigate the relationship between stressors and suicidal ideation during the  start of the COVID-19 pandemic. | They found that suicidal ideation increased by 12·9 percentage points and was almost 4.8 times higher during the COVID-19 pandemic. Suicidal ideation was more prevalent among people facing difficulty paying rent (31·5%), job loss (24·1%), and loneliness (25·1%), with each stressor associated with suicidal ideation in bivariable models. |
| Aboagye et al, 2022^119^ | 19,119 | 14 | Cross-sectional | Low friend relationships. | To investigate the associations between loneliness, social support, and suicidal ideation  among adolescents. | They found that felt lonely (aOR = 1·88, 95% CI = 1·69, 2·09) were more likely to experience suicidal ideation. |
| Macalli et al, 2022^232^ | 1,913 | 15 | Cross-sectional | Mental disorder. | To investigate the contribution of perceived loneliness to suicidal ideation among college students during the COVID-19 pandemic, and to explore the role of depressive symptoms in this association. | They found that loneliness was associated with a fourfold higher risk of suicidal thoughts (aOR = 4·34; 95% CI: 3·17–5·95), with adjustment for age, gender, inclusion period, interactions by phone, interactions on social network, marital status, and history of psychiatric disorders. |
| Olié et al, 2022^290^ | 296 | 5 | Cohort | Anxiety;  Depression. | To investigate determine whether depressive, anxious symptomatology and suicidal ideation (i.e. mental health outcomes) were influenced by stay-at-home orders, and to identify the psychosocial dimensions that influenced these mental health outcomes in patients with pre-existing depression during/after COVID-19-related restrictions. | They found that loneliness and boredom were independent risk factors of anxiety and depression, and their changes dynamically affected the psychological state. Suicidal ideation was mostly driven by depressive symptomatology. |
| Nilsson et al, 2022^321^ | 14 | 21 | Qualitative | Low familial support. | To investigate the loss of a family member by suicide, based on the lived experience of suicide survivors. | They found that essence of losing a family member by suicide encompasses experiences of involuntary and existential loneliness, life suffering, and additional burdens in a life that is radically transformed, comprising prolonged and energy-intensive attempts to understand. |
| Mournet et al, 2022^291^ | 74 | 5 | Cohort | Perceived  Burdensomeness. | To investigate the contexts under which social support seeking may be related to greater, or lesser, suicidal ideation. | They found that participants felt burdensome and sought support, they had greater odds of reporting suicidal ideation (OR = 1·659, 95% CI = [1·420, 1·938]), compared with days they felt burdensome but did not seek support. |

Continue to the next page

**Continuing Table 2. Summary of studies included in the review of loneliness and suicidal behavior**

| **Citation** | **Sample size** | **Quality** | **Design** | **Associated factor** | **Study aim** | **Relevant Result** |
| --- | --- | --- | --- | --- | --- | --- |
| Wolf et al, 2022^315^ | 64 | 6 | Clinical trial | Depression | To investigate loneliness versus social network size as interpersonal risk factors for suicidal ideation and behavior in persistent depressive disorder and assess effects of cognitive behavioral analysis system of psychotherapy on this domain. | They found that suicidal ideation and behavior appears to be associated with interpersonal factors related to loneliness in persistent depressive disorder, but not in borderline personality disorder. |
| Marthoenis et al, 2022^233^ | 4,993 | 15 | Cross-sectional | Bulling;  Psychological distress;  Tabaco. | To investigate investigates the prevalence of loneliness and associated health-risk behaviors among Indonesian female adolescents. | They found that adolescents who have no close friends, have been bullied during the past month, experience sleep disturbance, have had suicide ideation and suicide plans, are always feeling hungry, are exposed to passive smoking, and are engaged in a sedentary lifestyle are at a higher likelihood of suffering from loneliness (p<0.05). |
| McClelland et al, 2022^324^ | 10 | 22 | Qualitative | Lack of Emotional Connectedness;  Lack of Feeling Understood. | To investigate the nature of loneliness experienced prior to suicide, and the role it has in association with other preceding factors, has not been fully explored. | They found a positive relationship with parents, knowing someone with similar experiences or having membership in more than one friendship group may reduce feelings of  loneliness and/or intentions to die. |
| Mao et al, 2022^234^ | 964 | 15 | Cross-sectional | Perceived discrimination. | To investigate the mediating effect of social support and loneliness in the relationships between perceived discrimination and suicidal ideation among impoverished Chinese college students. | They found that perceived discrimination, loneliness, and suicidal ideation were positively correlated with each other; social support was negatively correlated with perceived discrimination, loneliness, and suicidal ideation. |
| Lapane et al, 2022^336^ | - | 30 | Review | Depression. | To review the literature regarding the health impact of loneliness among older adults living in congregate living settings (i.e., assisted living, nursing homes). | They found that loneliness appeared common among older residents in congregate living situations. In most studies, loneliness was associated with depression (regardless of scale used), albeit all but one came from cross-sectional studies. |
| Badcock et al, 2022^292^ | 188 | 5 | Cohort | Losing belongingness. | To investigate whether high and low levels of loneliness are associated with different patterns of response to losses or gains of belongingness. | They found that the high loneliness group exhibited a larger magnitude of effect on desire to quit from gaining belongingness than for losing belongingness. In contrast, the low loneliness group showed a larger change in desire to quit from losing belongingness than gaining belongingness. |

Continue to the next page

**Continuing Table 2. Summary of studies included in the review of loneliness and suicidal behavior**

| **Citation** | **Sample size** | **Quality** | **Design** | **Associated factor** | **Study aim** | **Relevant Result** |
| --- | --- | --- | --- | --- | --- | --- |
| Wang et al, 2022^120^ | 8,452 | 14 | Cross-sectional | Childhood trauma. | To investigate the relationship between childhood trauma and suicidal ideation from the perspectives of indifference, identification with all humanity, and loneliness. | They found that suicidal ideation was positively correlated with indifference and loneliness, and negatively correlated with identification with all humanity (r = − 0·082~0·260, p < 0·001). |
| Psychogiou et al, 2022^293^ | 1,623 | 5 | Cohort | Depression. | To investigate whether adolescents’ loneliness and social withdrawal mediated the association between maternal depressive symptoms and  adolescent suicidality. | They found that loneliness explained 16% of the total effect of maternal depressive symptoms on adolescent suicidality (indirect effect OR = 1·02, 95% CI: 1·00–1·04). |
| Pengpid and Peltzer, 2022^121^ | 9,143 | 13 | Cross-sectional | Bulling. | To investigate the estimate associations of loneliness with poor mental health, social-environmental and health risk behavior indicators among adolescents. | They found that loneliness was significantly positively associated with bullying victimization, having been physically attacked, involvement in fighting, frequent experiences of hunger and passive smoking (among boys). |
| Beutel et al, 2022^273^ | 633 | 2 | Cohort | Anxiety | To investigate assessed the prevalence of loneliness and its impact on psychological symptoms over time in patients with cancer. | They found loneliness was still predictive of more severe  anxiety symptoms and suicidal ideation over two years later. |
| Chiu and Vargo, 2022^235^ | 5,290 | 15 | Cross-sectional | Alcohol abuse;  Anxiety. | To investigate the association between gender differences in bullying and suicidal behavior, as well as examine the association between gender differences in the selected risk factors associated with suicidal behavior among secondary school adolescents. | They found that strong predictors of suicidal behaviors were loneliness and getting in trouble due to alcohol consumption. |
| Kim et al, 2022^122^ | 3,000 | 14 | Cross-sectional | Permissiveness. | To investigate the relationship between attitudes toward suicide and suicidal behavior. | They found that “permissiveness,” “unjustified behavior,” and “loneliness” factors showed significant trends across the suicidal behavior continuum. |
| Bednarova et al, 2022^294^ | 121 | 5 | Cohort | Absence of social support. | To investigate the motives and factors connected to suicidal behavior in hospitalized patients with intentional self-harm. | They found that living alone and the absence of social support increased the likelihood of suicidal attempt. |

Continue to the next page

**Continuing Table 2. Summary of studies included in the review of loneliness and suicidal behavior**

| **Citation** | **Sample size** | **Quality** | **Design** | **Associated factor** | **Study aim** | **Relevant Result** |
| --- | --- | --- | --- | --- | --- | --- |
| Stanley et al, 2022^123^ | 4,069 | 14 | Cross-sectional | High levels of total  trauma burden. | To investigate associations between non-response to a question assessing lifetime self-  injurious thoughts and behaviors and proxy variables of suicide risk. | They found that veterans who declined to respond to a survey-based assessment of lifetime suicidal ideation and suicide attempts may, in fact, represent a covert subgroup who is at heightened risk for suicide, as indicated by several proxy measures of potential suicide risk. |
| Abio et al, 2022^124^ | 193,464 | 14 | Cross-sectional | Anxiety;  Substance abuse. | To investigate estimate the prevalence and identify individual-level and country-level factors which might explain the variability in suicidal behavior among students. | They found that strongest risk factors associated with suicidal behavior included anxiety, loneliness, no close friends, and the substance abuse. |
| Landa-Blanco et al, 2022^125^ | 1,696 | 14 | Cross-sectional | Depression. | To investigate the psychological factors associated with suicide risk in university students. | They found a significant inverse relation between suicide risk and hope, the presence and search for a meaning in life, and interpersonal support. However, a higher suicide risk is positively correlated with loneliness and depression. |
| Fernandez-Rodrigues et al, 2022^29^ | 777,412 | 27 | Review | Depression. | To review the evidence on the potential risk factors for suicide behavior development in depressive older adults, and to examine the effects of depression treatment to tackle suicide behavior in this population. | They found that the risk was related to depressive episode severity, psychiatric comorbidity (anxiety or substance use disorders), poorer health status, and loss of functionality. Reduced social support and loneliness were also associated with suicide behaviour in depressive older adults. |
| Nichter et al, 2022^277^ | 2,307 | 4 | Cohort | Trauma exposures | To investigate the risk factors associated with suicide attempts. | They found that strongest risk factors for suicide attempts were higher baseline levels of loneliness, lower baseline levels of adaptive psychosocial traits (e.g., dispositional gratitude), baseline thoughts of self-harm, and greater post-baseline trauma exposures (12·3%–41·3% of explained variance). |
| Smith et al, 2022^126^ | 34,129 | 14 | Cross-sectional | Visual impairment. | To investigate the associations of objectively  measured distance visual impairment with suicidal ideation and suicide attempts among adults aged ≥50 years. | They found that severe visual impairment was significantly associated with suicidal ideation (OR=9·50; 95% CI=2·47 to 36·52). |

Continue to the next page

**Continuing Table 2. Summary of studies included in the review of loneliness and suicidal behavior**

| **Citation** | **Sample size** | **Quality** | **Design** | **Associated factor** | **Study aim** | **Relevant Result** |
| --- | --- | --- | --- | --- | --- | --- |
| Sarfo et al, 2022^236^ | 1,877 | 15 | Cross-sectional | Bulling;  Tabaco. | To investigate the prevalence, risk and protective variables associated with suicidal behaviors. | They found that truancy, cigarette smoking, bullying victims (on/off school property), being cyberbullied, loneliness, and worry as risk factors for suicide ideation among adolescents. Risk for suicide plan was predicted by truancy, cigarette smoking, physical fight, bullying victims (on/off school property), being cyberbullied, loneliness, and worry. |
| Straus et al, 2022^127^ | 4,069 | 13 | Cross-sectional | Mental disorder. | To investigate the current prevalence of loneliness, and the relation between loneliness severity and mental and physical health conditions, suicidality, and functional measures in a predominantly older sample. | They found that loneliness severity was independently associated with a range of mental health (odds ratios [ORs] = 1·21−33·30), physical health (ORs = 1·21−6·80),  and functional difficulties (d’s = 0·09−0·59). Relative to hardly ever feeling lonely, feeling lonely often or sometimes was associated with a more than 12-and three-fold greater likelihood of current suicidal ideation |
| You et al,2021^237^ | 3,095 | 16 | Cross-sectional | Depression; Impulsivity. | To investigate the suicide risk in migrant workers and the relation of suicide risk with loneliness，depression and impulsivity. | They found that loneliness, depression, and impulsivity increased suicide risk among migrant workers, with loneliness (USL-6), depression (PHQ-9), and impulsivity (BIS-11) positively associated with suicide risk (OR = 1·28, 1·16, 1·09). Structural equation modeling confirmed that these three variables predicted suicide risk (β = 0·32, 0·17, 0·13), and bootstrap testing further showed that loneliness was positively associated with depression and impulsivity. |
| Luo et al,2021^238^ | 3,251 | 15 | Cross-sectional | Anxiety;  Depression. | To investigate the relationship between negative emotion and its factors and suicidal idea among college graduate students. | They found that females reported higher depression, anxiety, loneliness, and suicidal ideation scores than males, and graduate students scored lower on all four outcomes than undergraduates. Depression, anxiety, and loneliness were positively correlated (r = 0·536, 0·553, 0·499), and each significantly predicted suicidal ideation. |

Continue to the next page

**Continuing Table 2. Summary of studies included in the review of loneliness and suicidal behavior**

| **Citation** | **Sample size** | **Quality** | **Design** | **Associated factor** | **Study aim** | **Relevant Result** |
| --- | --- | --- | --- | --- | --- | --- |
| Li et al,2021^128^ | 2,626 | 13 | Cross-sectional | Economic disadvantage;  Higher hopelessness / sleep-related scores (not predictors);  Women,. | To investigate suicidal ideation and loneliness among economically disadvantaged university students and analyze their association. | They found that economically disadvantaged students had a higher prevalence of suicidal ideation (5·83% vs. 3·05%). Disadvantaged students scored higher on hopelessness, sleep problems, and suicidal ideation, with females showing higher hopelessness, sleep problems, and suicidal ideation than males. Loneliness significantly predicted suicidal ideation. |
| Jin et al,2021^129^ | 414 | 13 | Cross-sectional | Psychopathy; Social support. | To investigate the influence and mechanism of psychopathy on suicidal ideation. | They found that psychopathy was positively correlated with loneliness and suicidal ideation (r = 0·36, 0·56) and negatively correlated with perceived social support (r = −0·33). Loneliness significantly mediated the effect of psychopathy on suicidal ideation, accounting for 84·62% of the total effect, while perceived social support moderated both the psychopathy→loneliness (β = 0·08) and loneliness→suicidal ideation paths (β = −0·20). |
| Na et al,2021^337^ | 66,941 | 30 | Review | Female gender; Being Uneducated;  Non-married status;  Poor financial status;  Living alone;  Chronic disease;  Impaired ability of daily living;  Depression;  Loneliness;  Negative life events;  Unfilial children;  Family relations;  Social support. | To investigate the related factors of suicidal ideation in the elderly in China | They found that multiple sociodemographic, health, and psychosocial factors, including female gender (OR = 1·36), low education (OR = 1·36), non-married status (OR = 1·27), poor financial status (OR = 1·78), living alone (OR = 1·38), chronic disease (OR = 1·85), impaired daily living ability (OR = 1·52), depression (OR = 3·83), loneliness (OR = 1·37), negative life events (OR = 2·13), and unfilial children (OR = 1·74), were significantly associated with suicidal ideation, while good family relations (OR = 0·53) and higher social support (OR = 0·80) were protective. |

Continue to the next page

**Continuing Table 2. Summary of studies included in the review of loneliness and suicidal behavior**

| **Citation** | **Sample size** | **Quality** | **Design** | **Associated factor** | **Study aim** | **Relevant Result** |
| --- | --- | --- | --- | --- | --- | --- |
| Nishizuka, 2021^328^ | 8,009 | 11 | Review | Economic problems;  Health problems;  Family problems; Lifestyle problems. | To review the extent to which health and suicide risks can be expected among the elderly by refraining from going out and interacting with others. | They found that of the 8,009 persons aged 60 or older for whom a cause or motive could be identified, “health problems” accounted for 5,187 (64·7%), followed by “family problems” (1,229, 15·3%) and “economic and lifestyle problems” (948, 11·8%). The most common “health problems” included mental illness, physical illness, and physical disability. Suicide due to loneliness accounted for 224 (3·1%). |
| Tragantzopoulou et al, 2021^23^ | - | 11 | Review | Anxiety; Depression;  Psychotic disorder. | To review social isolation and loneliness while highlighting the serious repercussions on health and behavior. | They found high levels of mortality have been observed in both social isolation and loneliness and it is significantly possible that suicidal behavior can play a vital role for these high scores. Research investigating the links among social isolation, loneliness and suicidal behavior is scarce with existing findings indicating that adolescents and elderly are vulnerable to both loneliness and suicidal behavior. Lonely individuals are prone to perceiving life as stressful and unbearable, with chronic stress being also related with high levels of suicidal ideation. A meta- analysis found that having no partner, living alone, social isolated, feeling lonely, feeling alienated from others, and feeling not to belong are the main social constructs that can highly contribute to suicidal outcomes. In this study, subjective loneliness had the greatest impact on both suicidal ideations and suicidal attempts. This outcome was reinforced by an integrative meta-analysis showing that the function and the quality of social relationships are more predictive of suicidal behavior. In view of these findings, meaningful social relationships seemingly can constitute a protective factor against suicidal behavior. |
| Wang et al, 2021^130^ | 538 | 14 | Cross-sectional | Limited activities of daily living. | To investigate the association among limited activities of daily living, loneliness and suicidal ideation, and examined the multiple moderating effect of social support on the mediation model. | They found the multiple moderating role of social support in the effect of limited ADL and loneliness on suicidal ideation among nursing home residents. |
| Allan et al, 2021^131^ | 1,070 | 14 | Cross-sectional | Anxiety | To investigate the relations that loneliness, anxiety sensitivity, and intolerance of uncertainty shared with suicidal ideation during the early stages of the COVID-19 pandemic. | They found that loneliness and anxiety sensitivity were positively, uniquely associated with suicidal ideation across samples. |

Continue to the next page

**Continuing Table 2. Summary of studies included in the review of loneliness and suicidal behavior**

| **Citation** | **Sample size** | **Quality** | **Design** | **Associated factor** | **Study aim** | **Relevant Result** |
| --- | --- | --- | --- | --- | --- | --- |
| McClelland et al, 2021^239^ | 400 | 15 | Cross-sectional | Emotional Abuse. | To investigate the relationship between loneliness and self-injurious thoughts and behaviors. | They found that loneliness independently distinguished between participants with no history of self-injurious thoughts or behaviors, those with a history of self-injurious thoughts only, and those with a history of self-injurious behaviors. |
| Ernst et al, 2021^240^ | 2,450 | 15 | Cross-sectional | Younger men. | To investigate gender- and age-dependent associations of loneliness and suicidal  ideation. | They found that gender-specific analyses showed that the association of loneliness and suicidal ideation was especially strong among younger men (loneliness x age within the male sample: β=-.149; p=.014). |
| Yang et al, 2021^241^ | 538 | 15 | Cross-sectional | Depression. | To investigate the mediating role of depressive symptoms and the moderating effect of resilience on the risk factors of suicidal ideation to attenuate the adverse contribution among nursing home residents. | They found that mediating model (H1: B = 0·477, p < 0·001; H2: B = 0·325, p < 0·001; H3: B = 0·308, p < 0·001) and the moderating effect of resilience interacting loneliness (H4: B = −0·133, p < 0·001; H6: B = −0·109, p < 0·001) and depressive symptoms (H5: B = −0·077, p < 0·001) were statistically significant. |
| Antonelli-Salgado et al, 2021^278^ | 1,674 | 4 | Cohort | COVID-19. | To investigate whether subjective (such as loneliness) and objective (such as social distancing, living alone, and staying only indoors) measures of social relationships are risk factors for suicidal ideation in the COVID-19 pandemic considering a one-month follow-up. | They found that living alone (OR: 1·16; 95%CI = 1·03 – 1·30; p=0·015), number of days practicing social distancing (OR: 1·002; 95%CI = 1·000 – 1·004; p=0·027), and loneliness (OR: 1·49; 95%CI = 1·32 – 1·68; p<0·001) were associated with suicidal ideation in the cross-sectional analysis of W1. Only loneliness (OR= 2·12; 95%CI = 1·06 - 4.24; p = 0·033) remained significant as a risk factor to suicidal ideation in the longitudinal analysis between both waves. |
| Gomboc et al, 2021^242^ | 991 | 15 | Cross-sectional | Stress. | To investigate the prevalence of death ideation and suicidal ideation in the general population across four age groups and to determine the extent to which emotional and social loneliness are associated with suicidal ideation. | They found that several factors appeared as important predictors of suicidal ideation, with emotional loneliness being a significant factor. |

Continue to the next page

**Continuing Table 2. Summary of studies included in the review of loneliness and suicidal behavior**

| **Citation** | **Sample size** | **Quality** | **Design** | **Associated factor** | **Study aim** | **Relevant Result** |
| --- | --- | --- | --- | --- | --- | --- |
| Louie et al, 2021^78^ | 64 | 4 | Case-control | Depression. | To investigate patients with late-life depression and healthy controls in terms of suicidal ideation during the COVID-19 pandemic, and to determine predictors of suicidal ideation. | They found that older people with LLD had a higher level of suicidal ideation than healthy controls, after controlling for the level of depression and medical comorbidity (F (1, 59) = 5.72, p = 0·020). Regression analyses showed that coping efficacy and loneliness accounted for a significant portion of the variance in suicidal ideation, and loneliness significantly predicted the level of stress. |
| Shaw et al, 2021^295^ | 502,536 | 5 | Cohort | Men. | To investigate how living alone, loneliness and emotional support were related to suicide and self-harm in a longitudinal design. | They found for men, both living alone (Hazard Ratio (HR) 2·16, 95%CI 1·51–3·09) and living with non-partners (HR  1·80, 95%CI 1·08–3·00) were associated with death by suicide, independently of loneliness, which had a modest  relationship with suicide (HR 1·43, 95%CI 1·01–2·03). For women, there was no evidence that living arrangements, loneliness or emotional support were associated with death by suicide. |
| Ari and Mari, 2021^318^ | 2,644 | 13 | Qualitative | Mental disorder. | To investigate the reasons to consider suicide and to describe the kind of thoughts they have for and against suicide. | They found that suicides motives involved experiences of failure, loneliness and worthlessness, often combined with mental health problems and triggered by loss of employment or significant relationship. |
| Gijzen et al, 2021^243^ | 5,888 | 16 | Cross-sectional | Depression. | To investigate factors association with suicidal ideation. | They found that loneliness explained most variance of suicide ideation. |
| Zhang et al, 2021^244^ | 538 | 15 | Cross-sectional | Low resilience. | To investigate the mediating effect of resilience on the association between loneliness and suicidal ideation and whether this mediating effect was moderated by social support. | They found that overall social support moderated the resilience on suicidal ideation, indirectly impacting loneliness on suicidal ideation (moderating effect = 0·086 [95% CI =0·005–0·167]). |
| Lutzman et al, 2021^132^ | 198 | 14 | Cross-sectional | Pain. | To investigate the role of loneliness and social integration as potential mediators in the relationship between physical pain and suicidal ideation in the old adults. | They found that the association between physical pain and suicidal ideation was mediated by loneliness and social integration. |

Continue to the next page

**Continuing Table 2. Summary of studies included in the review of loneliness and suicidal behavior**

| **Citation** | **Sample size** | **Quality** | **Design** | **Associated factor** | **Study aim** | **Relevant Result** |
| --- | --- | --- | --- | --- | --- | --- |
| John et al, 2021^245^ | 12,975 | 15 | Cross-sectional | Unemployed;  Young;  Women. | To investigate assess the effects and change in effects of risk factors including loneliness and coping, as well as pre-existing mental health conditions on suicidal thoughts and self-harm during the COVID-19 pandemic. | They found that young persons (aged 18–24 years), females, students, those who were unemployed and individuals with pre-existing mental health conditions were more likely to report feeling lonely and not coping well. |
| Lirón et al, 2021^133^ | 4,217 | 10 | Cross-sectional | Middle-aged | To investigate the relation between suicidal ideation and social factors (loneliness, social support, trust, participation and cohabiting). | They found that in the middle-aged groups, loneliness is significantly associated with suicidal ideation in both women and man. |
| Oppong Asante et al, 2021^134^ | 2,798 | 14 | Cross-sectional | Alcohol abuse;  Anxiety;  Bulling;  Drugs abuse. | To investigate the 12-month prevalence of suicidal behaviors amongst school-going adolescents and describe some commonly reported psychosocial factors associated. | They found that personal level, being aged 18 or older, loneliness, and health risk behaviors (cannabis use, and leisure time sedentary behavior) were associated with increased odds of suicidal behaviors. |
| Smith et al, 2021^135^ | 162,994 | 14 | Cross-sectional | Alcohol abuse;  Drugs abuse;  Food insecurity;  Insomnia. | To investigate the association between putative physical, behavioral, and social correlates with multiple suicide attempts. | They found that among those who had attempted suicide at least once in the past 12 months, in the overall sample, food insecurity, smoking, alcohol consumption, cannabis use, amphetamine use, sedentary behavior, sexual intercourse, sleep problems, loneliness, no close friends, and bullying victimization were all independently associated with higher odds for multiple suicide attempts although some regional differences were observed. |
| Scheer et al, 2021^309^ | 1,047 | 7 | Cohort | Childhood sexual abuse. | To investigate the associations between childhood sexual abuse and suicidal thoughts and behaviors. | They found that among childhood sexual abuse exposed men were prospectively associated with passive suicidal ideation (adjusted odds ratio [aOR] = 1·38; 95% CI = 1·19; 1·61). |
| Farooq et al, 2021^41^ | 120,076 | 33 | Review | Insomnia. | To review studies that describing suicidal ideation, suicide attempts and suicide and associated risk factors during COVID-19 pandemic. | They found that main risk factors for suicidal ideations were: low social support, high physical and mental exhaustion and poorer self-reported physical health in frontline medical workers, sleep disturbances, quarantine and exhaustion, loneliness, and mental health difficulties. |

Continue on the next page

**Continuing Table 2. Summary of studies included in the review of loneliness and suicidal behavior**

| **Citation** | **Sample size** | **Quality** | **Design** | **Associated factor** | **Study aim** | **Relevant Result** |
| --- | --- | --- | --- | --- | --- | --- |
| López Steinmetz et al, 2021^296^ | 1,202 | 5 | Cohort | Mental disorder. | To investigate changes on suicidal risk levels, adjusting for impulsivity-related traits, quarantine duration, main demographic factors, mental disorder history, and loneliness, in college students with and without suicidal behavior history, during a quarantine of up to 103-day duration of the COVID-19 pandemic. | They found that suicidal risk diminished from the first measurement to the follow-up, having mental disorder history predicted higher suicidal risk, and negative urgency had the largest increasing effects on suicidal risk which persisted over time. |
| Hasan et al, 2021^246^ | 280,076 | 15 | Cross-sectional | Bulling. | To investigate the pathways explaining the association between bullying victimization and suicidal behaviors among school-based adolescents. | They found that nearly one-fourth (18·7%) of the total association between bullying and suicidal ideation was mediated by loneliness. |
| Harman et al, 2021^79^ | 11,369 | 6 | Case-control | Impulsivity. | To investigate the predictive models for suicidal ideation in a sample of children aged 9–10 using features previously implicated in risk among older adolescent and adult populations. | They found that feelings of loneliness, feelings of worthlessness, impulsivity, prodromal psychosis symptoms, and behavioral problems had been associated with suicidal behavior. |
| Na et al, 2021^297^ | 3,078 | 5 | Cohort | Financial stress. | To investigate examine longitudinal risk and protective factors of suicidal ideation during the COVID-19 pandemic in a nationally representative sample of veterans with pre-existing psychiatric conditions. | They found that older age, greater pre-pandemic psychiatric symptom severity, past-year suicidal ideation, lifetime suicide attempt, psychosocial difficulties, COVID-19 infection, and past-year increase in psychiatric symptom severity were linked to peri-pandemic suicidal ideation, while pre-pandemic higher income and purpose in life were protective. |
| Madsen and Harris, 2021^136^ | 713 | 13 | Cross-sectional | Financial stress. | To investigate the drivers of suicide through re-investigation of individuals’ reasons for dying as possible indicators of suicide risk. | They found strong association between the number of reported reasons for dying and suicidality. |
| Pengpid and Peltzer, 2021^137^ | 3,333 | 13 | Cross-sectional | Anxiety;  Bulling;  Women. | To investigate the frequency of suicide attempts as well as its associated factors among adolescents. | They found that female sex, loneliness, having no close friends, often bullied, frequently in a physical fight, frequently truant from school, ever amphetamine use, and past-month tobacco use were associated with ≥2 suicide attempts. |
| Li, You et al, 2021^138^ | 130,488 | 14 | Cross-sectional | Anxiety. | To investigate the prevalence of suicidal behaviors and their associated factors in young adolescents in low- and middle-income countries. | They found that factors associated with suicidal behaviors were being female, older age, loneliness, anxiety, a lack of close friends, and having family supportive (all *P* < 0·001). |

Continue on the next page

**Continuing Table 2. Summary of studies included in the review of loneliness and suicidal behavior**

| **Citation** | **Sample size** | **Quality** | **Design** | **Associated factor** | **Study aim** | **Relevant Result** |
| --- | --- | --- | --- | --- | --- | --- |
| Tabares and Peralta, 2021^267^ | 422 | 15 | Cross-sectional | Mental disorders. | To investigate associated factors with suicidal behavior in adolescents. | They found an association between loneliness and suicidal behavior. |
| Zhou et al, 2020^247^ | 3,553 | 15 | Cross-sectional | Alcohol;  Low self-esteem; Loneliness;  Poor emotional support;  Poor economic support. | To investigate the suicidal ideation among unmarried female migrant workers in Shanghai and Guangzhou and explore the association between suicidal ideation and some psychosocial problems, reproductive health factors. | They found that significant predictors of SI were alcohol use (OR = 1·42, 95% CI 1·04–1·93), low self-esteem (OR = 1·47, 95% CI 1·03–2·11), loneliness (OR = 3·30, 95% CI 2·42–4·51), poor emotional support (OR = 2·68, 95% CI 2·00–3·61), and poor economic support (OR = 3·79, 95% CI 2·86–5·04). |
| Park et al, 2020^338^ | - | 36 | Review | Anxiety;  Depression. | To review the comparative effects of loneliness on multiple distinct health outcomes. | They found the pooled correlation between loneliness and depression was -0·497, with a 95% confidence interval (CI) of -0·524 to -0·469. Similarly, for anxiety (2b), suicidality (2c), and general mental health (2d), the pooled correlations were -0·417 (95%CI: -0·469, -0·362), -0·516 (95%CI: -0·629, -0·381), and -0·489 (95%CI: -0·556, -0·415), respectively. |
| Papagavriel et al, 2020^139^ | 6,877 | 14 | Cross-sectional | Mental disorders. | To investigate the prevalence of loneliness and the association between loneliness and socio-demographic and clinical variables in people with borderline intellectual impairment and the general population. | They found that loneliness was associated with lower wellbeing and higher rates of common mental disorders, suicidal thoughts, and chronic physical disorders in both groups. Intellectual functioning moderated the relationship between loneliness and income (OR 1·82; 95%CI 1·06 to 3·11) and suicidal thoughts in the last week (OR 0·13; 95% CI 0·02 to 0·93). |
| Wang et al, 2020^248^ | 519 | 15 | Cross-sectional | Depression. | To investigate whether psychosocial health problems among patients with sexually transmitted infections were associated with suicidal ideation and to examine the syndemic effect of multiple psychosocial problems on suicidal ideation. | They found that depression (OR: 4·1; 95% CI: 2·3–7·2) and entrapment (OR:2·1; 95%CI: 1·1–4·1) each had a more significant relation with suicidal ideation than the other psychosocial problems examined. sexually transmitted infection patients who experienced two or more psychosocial health problems had approximately fourfold odds of suicide ideation (adjusted OR [aOR]: 4·2; 95%CI: 2·6–6·8) compared with those in the non-syndemic group, especially in the high-level (five or more psychosocial problems) group (aOR: 7·0; 95%CI: 3·9–12·5). |

Continue on the next page

**Continuing Table 2. Summary of studies included in the review of loneliness and suicidal behavior**

| **Citation** | **Sample size** | **Quality** | **Design** | **Associated factor** | **Study aim** | **Relevant Result** |
| --- | --- | --- | --- | --- | --- | --- |
| Pitman et al, 2020^140^ | 3,193 | 14 | Cross-sectional | Perceived stigma of bereavement. | To investigate the hypothesis that among people who experience sudden bereavement, loneliness is associated with post-bereavement SA and post-bereavement SI. | They found that loneliness was significantly associated with probability of post-bereavement suicide attempt (aOR 1·19; 95% CI 1·14–1·25) and of post-bereavement suicidal ideation (aOR 1·24; 95% CI 1·20–1·28). |
| McClelland et al, 2020^339^ | - | 36 | Review | Depression. | To review the extent to which loneliness predicts SI and/or behavior over time. | They found that loneliness was a significant predictor of both suicidal ideation and behavior and there was evidence that depression acted as a mediator. |
| Gratz et al, 2020^141^ | 500 | 13 | Cross-sectional | Thwarted belongingness. | To investigate the relations of two COVID-19 consequences (i.e., stay-at-home orders and job loss) to suicide risk through thwarted belongingness, perceived burdensomeness, and loneliness. | They found a significant indirect relation of stay-at-home order status to suicide risk through thwarted belongingness. |
| Niu et al, 2020^80^ | 484 | 6 | Case-control | Depression. | To investigate loneliness and other psychosocial factors in older adults’ suicide cases and explore their interaction effects. | They found that individuals who were unemployed [odds ratio (OR) = 2.344; 95% confidence interval (CI): 1·233–4·457], living alone (OR = 2·176; 95% CI: 1·113–4·254), had lower levels of subjective social support (OR = 2(185; 95% CI: 1·243–3·843), experienced depressive symptoms (OR = 6·700; 95% CI: 3·405–13·182), showed higher levels of hopelessness (OR = 7·253; 95% CI: 3·764–13·974) and felt higher levels of hopelessness × higher levels of loneliness (OR = 2·446; 95% CI: 1·089–5·492) were significantly associated with an elevated suicide risk in older people. |
| Klim et al, 2020^298^ | 63 | 6 | Cohort | Perceived rejection;  Thwarted Belongingness. | To investigate the potential for the Adult Social Relationship social support scales and their represented constructs to explain variation in SI. | They found that loneliness and perceived rejection were associated with SI at baseline. |
| Castelletti et al, 2020^90^ | 4,753 | 8 | Cross-sectional | Depression. | To investigate the association between the subjective experience of loneliness and SI. | They found that higher feelings of loneliness were significantly associated with greater odds for suicidal ideation (OR = 1·02; 95% CI = 1·01,1·02). After the exclusion of those individuals with depression, the association of loneliness with SI remained significant (OR = 1·01; 95% CI = 1·00,1·03; p < 0·001). |

Continue on the next page

**Continuing Table 2. Summary of studies included in the review of loneliness and suicidal behavior**

| **Citation** | **Sample size** | **Quality** | **Design** | **Associated factor** | **Study aim** | **Relevant Result** |
| --- | --- | --- | --- | --- | --- | --- |
| Santhanaraj, 2020^86^ | 64 | 3 | Cross-sectional | Unemployed; Unmarried. | To investigate the association of subjective sense of loneliness and SI among suicidal attempters and to correlate it with severity of SA. | They found that males of 31–45 years who were unemployed, unmarried to have more subjective sense of loneliness. Subjective sense of loneliness has positive correlation with severity of SA. |
| Shahedifar et al, 2020^268^ | 2,599 | 15 | Cross-sectional | Alcohol abuse;  Bulling. | To investigate the prevalence of and correlates for suicidal behaviors among school attending adolescents. | They found that attempters were more likely to have SI (OR=10·58; 95% CI 5·10, 21·97); have planned suicide (OR=9·82; 95% CI 4·60, 20·96); or sustained serious  injury (OR=4·01; 95% CI 2·03, 7·93). |
| Pengpid and Peltzer, 2020^269^ | 2,744 | 15 | Cross-sectional | Drugs use;  Fast-food. | To investigate the prevalence and correlates of single and multiple suicide attempts among adolescents. | They found that having no close friends, loneliness, having been frequently physically attacked, ever used amphetamine and fast-food intake were associated with multiple SA in the past 12 months. |
| Pandey et al, 2019^142^ | 6,531 | 13 | Cross-sectional | Anxiety;  Food insecurity;  Tabaco. | To investigate the prevalence of suicidal ideation and attempt among adolescent students and identify the factors associated with them. | They found that food insecurity (OR = 2·32, CI = 1·62–3·32), anxiety (OR = 2·54, CI = 1·49–4·30), loneliness (OR = 2·51, CI = 1·44–4·36) and gender (OR = 1·39, CI = 1·03–1·89) were identified as risk factors of suicidal ideation. Anxiety (OR = 3·02, CI = 1·18–7·74), loneliness (OR = 2·19, CI = 1·28–3·73) truancy (OR = 1·99, CI = 1·40–2·82), cigarette use (OR = 3·13, CI = 1·36–7·23) and gender (OR = 1·60, CI = 1·07–2·39) were identified as risk factors of suicidal attempt. Having 3 or more close friends was found to have protective effect (OR = 0·35, CI = 0·16– 0·75) against suicidal attempt. |
| Mo et al, 2019^81^ | 484 | 6 | Case-control | Depression. | To investigate the validity of proxy informants’ reports on Life Event Scale for the Elderly (LESE) by using psychological autopsy method. | They found that LESE was positively correlated with depression, loneliness and hopelessness, and negatively correlated with quality of life, family function and social support both in suicide cases and living controls. |
| Bračič et al, 2019^249^ | 1,547 | 15 | Cross-sectional | Depression. | To investigate the prevalence of suicidal ideation among adolescents and to examine the predictive value of gender, depression, loneliness, multiple health complaints, bullying and degree of family support for the presence of suicidal thoughts. | They found that depressive feelings and loneliness were the most important predictors of suicidal ideation. |

Continue on the next page

**Continuing Table 2. Summary of studies included in the review of loneliness and suicidal behavior**

| **Citation** | **Sample size** | **Quality** | **Design** | **Associated factor** | **Study aim** | **Relevant Result** |
| --- | --- | --- | --- | --- | --- | --- |
| Ali and Gibson, 2019^317^ | 210 | 2 | Qualitative | Mental disorders. | To investigate the reasons that young people provide for feeling suicidal in posts published on a suicide prevention forum, hosted on the social media platform Tumblr. | They found that feeling lonely and socially disconnected, experiencing identity stigma, failing to meet expectations, being helpless, feeling worthless, and experiences of mental ill-health. |
| Calati et al, 2019^332^ | - | 20 | Review | Unmarried. | To review the link between social isolation and suicidal thoughts and behaviors. | They found that main social constructs associated with suicidal outcomes were marital status (being single, separated, divorced, or widowed) and living alone, social isolation, loneliness, alienation, and belongingness. |
| Chang et al, 2019^143^ | 156 | 10 | Cross-sectional | Depression. | To investigate whether depression and loneliness represent important unique predictors of suicide risk, namely hopelessness and suicidal behaviors; and to examine if there is evidence for a significant depression–loneliness interaction effect in predicting suicide risk. | They found evidence for a significant depression–loneliness interaction effect in predicting suicide risk. |
| Bennardi et al, 2019^299^ | 2,392 | 5 | Cohort | Older adults. | To investigate whether positive and negative affect, social support, and loneliness are factors longitudinally related to suicide ideation in the general population in different age groups. | They found that feelings of loneliness were related to suicidal ideation in 60-year-and-older individuals. |
| Chang, Lee et al, 2019^144^ | 224 | 13 | Cross-sectional | Sexual assault victimization. | To investigate sexual assault victimization and loneliness as predictors of self-harm behaviors in a sample of female college students. | They found that loneliness, but not sexual assault victimization, was the only unique and significant predictor after controlling for self-harm behaviors. |
| Kabtni et al, 2019^87^ | 30 | 4 | Cross-sectional | Depression. | To investigate loneliness as a risk factor for suicidal ideation in depressed patients. | They found that frequent to severe loneliness was identified as a risk factor for suicidal ideation, only in the group moderately severe depression. |
| Dema et al, 2019^270^ | 5,809 | 15 | Cross-sectional | Alcohol/drugs use;  Bulling;  Food insecurity;  Physical attack;  Sexual violence;  Tabacco;  Women. | To investigate the prevalence and factors associated with self-reported suicidal behavior among school going adolescents. | They found that female sex, food insecurity, physical attack, sexual violence, bullying, feeling of loneliness, low parental engagement, reported worry about lack of sleep, urge to use drugs/alcohol, smokeless tobacco use, drug abuse and parental smoking were the factors associated with suicidal attempt. All these factors except smokeless tobacco use and parental smoking were associated with suicidal ideation. |

Continue on the next page

**Continuing Table 2. Summary of studies included in the review of loneliness and suicidal behavior**

| **Citation** | **Sample size** | **Quality** | **Design** | **Associated factor** | **Study aim** | **Relevant Result** |
| --- | --- | --- | --- | --- | --- | --- |
| Pengpid and Peltzer, 2019^271^ | 4,274 | 15 | Cross-sectional | Alcohol abuse. | To investigate the prevalence of past 12-month  SAs and associated factors among in-school adolescents. | They found that male sex and loneliness were associated with past 12-month suicide SA, and among boys, none of the variables, and among girls, loneliness and current alcohol use were associated with past 12-month SA. |
| Kivelä et al, 2019^306^ | 230 | 6 | Cohort | Insomnia. | To investigate the longitudinal course of SI, and to identify predictors of persistent SI. | They found that more severe insomnia and hopelessness were associated with increased odds of persistent SI, and hopelessness was a significant mediator of the relationship between insomnia and persistent SI. |
| Zygo et al, 2019^198^ | 5,685 | 14 | Cross-sectional | Drug use;  Running away from home;  Single-parent family;  Women. | To investigate the prevalence of suicidal thoughts, tendencies, and SAs in young people. | They found that suicidal behavior in adolescents correlates with the female gender, intake of psychoactive substances, running away from home, being raised in a single-parent family, addiction of family members to alcohol, and experiences of violence. |
| Levi-Belz et al, 2019^199^ | 990 | 14 | Cross-sectional | Distress. | To investigate psychosocial processes that could facilitate disclosure of SAs. | They found that compared with disclosures and controls, non-disclosing SAs were characterized by higher levels of SI, distress, and victimization. |
| Yu et al,2018^250^ | 1,580 | 18 | Cross-sectional | Drug abuse; Alcohol abuse; Depression; Generalized anxiety; Social support. | To investigate and understand the SI among migrant construction male workers in Pudong New Area, Shanghai, and analyze the  influence factors based on social cognitive theory. | They found that drug abuse, alcohol abuse, depression, generalized anxiety, and loneliness were associated with higher odds of SI, while perceived social support was protective. In multivariate regression, alcohol abuse (OR = 2·15), depression (OR = 2·47), generalized anxiety (OR = 2·24), loneliness (OR = 2·09), and lower perceived social support (OR = 1·84) remained significant predictors. |
| Wang et al, 2018^319^ | 15 | 14 | Qualitative | Context of the family;  Poor physical health. | To investigate common themes in completed suicides by older Asian people living in New Zealand and to understand the factors contributing to suicide in this group. | They found that among the 15 cases of late-life suicide among older Asian adults in New Zealand, the majority (93·3%) lived with family members, yet many experienced significant social and cultural isolation. Physical health problems, including terminal illnesses, chronic pain, and frailty, were identified as key contributing factors in approximately 46·7% of the cases. Despite the severity of their circumstances, only 20% had any prior contact with psychiatric services, and just 13·3% were receiving mental health care at the time of death. |

Continue on the next page

**Continuing Table 2. Summary of studies included in the review of loneliness and suicidal behavior**

| **Citation** | **Sample size** | **Quality** | **Design** | **Associated factor** | **Study aim** | **Relevant Result** |
| --- | --- | --- | --- | --- | --- | --- |
| Mérelle et al, 2018^251^ | 14,322 | 15 | Cross-sectional | Psychological distress. | To investigate identify characteristics that are associated with non-disclosure of suicidal ideation. | They found that non-disclosure was significantly associated with social loneliness (OR = 1·29). Inverse significant associations were found for age (35–49 years, OR = 0·53), poor health status (OR = 0·63), frequent suicidal ideation (OR = 0·48), and severe psychological distress (OR = 0·63). |
| Niu et al, 2018^82^ | 484 | 5 | Case-control | Depression. | To investigate the validity of proxy respondent reports on loneliness, and the reliability and validity of the University of California Los Angeles Loneliness Scale-6 (ULS-6) as used in psychological autopsy method with rural older adults. | They found that rural older adults that live alone, are left behind by children, not currently married, and have higher depression and poor quality of life, are more likely to suffer from loneliness. |
| Hedley et al, 2018^252^ | 185 | 15 | Cross-sectional | Autism Spectrum Disorder;  Depression. | To investigate examine loneliness and social support, in addition to the contribution of Autism Spectrum Disorder trait severity, as risk and protective factors associated with depression and suicidal ideation in Autism Spectrum Disorder. | They found that loneliness, satisfaction with social support, and Autism Spectrum Disorder traits predicted depression scores. Satisfaction with social support predicted suicidal ideation, however, it was no longer a significant predictor after the effects of depression were considered. |
| Murphy et al, 2018^279^ | 141 | 3 | Cohort | Depression. | To investigate the frequency and nature of suicide among nursing home residents in Australia. | They found that common major life stressors identified in suicide cases included the following: health deterioration (n = 112, 79·4%); isolation and loneliness (n = 60, 42·6%); and maladjustment to nursing home life (n = 42, 29·8%). |
| Teo et al, 2018^145^ | 301 | 14 | Cross-sectional | Depression. | To investigate what degree several facets of social connectedness (number of confidants, social support, interpersonal conflict, social norms, and loneliness) are correlated with depression-related outcomes. | They found that loneliness was associated with higher levels of depression and suicidal ideation, as well as lower patient activation and help-seeking intentions. |
| Synnott et al, 2018^320^ | 100 | 10 | Qualitative | Mental disorder. | To investigate suicide notes of those who died by suicide and of survivors of suicide attempts. | They found that suicide note writers who died by their attempt were more likely to combine a dislike of themselves and a concern for loved ones. |

Continue on the next page

**Continuing Table 2. Summary of studies included in the review of loneliness and suicidal behavior**

| **Citation** | **Sample size** | **Quality** | **Design** | **Associated factor** | **Study aim** | **Relevant Result** |
| --- | --- | --- | --- | --- | --- | --- |
| Chang et al, 2018^253^ | 457 | 15 | Cross-sectional | Pessimism. | To investigate loneliness and optimism as predictors of suicide risk, specifically, depressive symptoms and SI, in college students. | They found the importance of fostering optimism for potentially lowering suicide risk among lonely college students. |
| Shaw et al, 2018^280^ | 150,000 | 4 | Cohort | Unmarried. | To investigate the interrelationships between loneliness, living arrangements and emotional support in predicting suicidal thoughts and behaviors. | They found that not living with a partner had a stronger relationship with death by suicide for men (HR 2·08, 95% CI 1·36 to 3·18) than for women (HR 1·16, 95% CI 0·59 to 2·31). |
| Mou et al, 2018^91^ | 35 | 7 | Cross-sectional | Borderline personality disorder;  Depression. | To investigate whether those borderline personality disorder would experience a stronger relationship between negative affect and suicidal ideation than those without such a diagnosis. | They found association between negative affective states (e.g., abandonment, desperation, guilt, hopelessness, loneliness, rage, self-hatred, and upset), and severity of suicidal thinking was stronger among those with borderline personality disorder than among those without borderline personality disorder. |
| Monteith et al, 2018^254^ | 115 | 15 | Cross-sectional | Sexual trauma. | To investigate psychiatric and interpersonal correlates of suicidal ideation and suicide attempt among survivors of military sexual trauma. | They found that military sexual trauma survivors who reported more severe psychological distress (OR= 2·88), hazardous alcohol use (OR = 1·14), and perceived general disapproval from others (OR = 1·14) were significantly more likely to report experiencing SI in the past two weeks. |
| Liu et al, 2018^200^ | 146,460 | 14 | Cross-sectional | Alcohol abuse;  Anxiety,  Bulling;  Poverty;  Tobacco. | To investigate the prevalence, distribution, and associated factors of SAs among young adolescents. | They found that factors associated with SAs included poor socioeconomic status, history of bullying, loneliness and anxiety, tobacco and alcohol use, and weak family and social relationships. |
| Xia et al, 2017^333^ | 31,324 | 23 | Review | Bulling;  Internet overuse; Ostracism; Depression. | To investigate the main influencing factors of suicidal ideation among middle school student in China | They found that aloneness (OR = 2·84), internet overuse (OR = 1·85), ostracism/bullying (OR = 1·81), and depression (OR = 1·71) were significantly associated with higher odds of SI. |
| Goodman et al, 2017^255^ | 532 | 15 | Cross-sectional | Low collective self-esteem. | To investigate the association between subjective social status and SI in a sample of young men (age 18–34 years). | They found that lower collective self-esteem and more loneliness expressed lower hope and meaning in life, which also mediated pathways to SI. |

Continue on the next page

**Continuing Table 2. Summary of studies included in the review of loneliness and suicidal behavior**

| **Citation** | **Sample size** | **Quality** | **Design** | **Associated factor** | **Study aim** | **Relevant Result** |
| --- | --- | --- | --- | --- | --- | --- |
| Mereish et al, 2017^146^ | 503 | 13 | Cross-sectional | Psychological distress. | To investigate the effects of distal minority stressors and proximal stressors on psychological distress and suicidality among bisexual adults. | They found that loneliness mediated the effects of distal and proximal minority stressors on psychological distress and suicidality. |
| Chang, Wan et al, 2107^147^ | 228 | 13 | Cross-sectional | Depression. | To investigate loneliness and future orientation as predictors of suicidal risk, namely, depressive symptoms and SI, in college. | They found that high future orientation, compared to low future orientation, were found to report significantly lower levels of depressive symptoms and SI. |
| Zhang et al, 2017^148^ | 205 | 13 | Cross-sectional | Depression. | To investigate the association factors with the suicidal ideation in institutional older adults’ residents in rural. | They found that hopelessness and depression had impacts on SI, and self-esteem and loneliness can impact SI through, mediating of depression and hopelessness. |
| Beutel et al, 2017^310^ | 15,010 | 7 | Cohort | Anxiety;  Depression. | To investigate the prevalence and determinants of different degrees of, and to determine its associations to mental health, health behavior and health care utilization in community. | They found that loneliness was associated with depression (OR = 1·91), generalized anxiety (OR = 1·21)  and SI (OR = 1·35). |
| Pando et al, 2017^149^ | 500 | 14 | Cross-sectional | HIV;  Younger. | To investigate the acceptability of rapid HIV self-testing among men who have sex with men. | They found that factors associated with SI include lower age (p<0·001) and feeling of loneliness (p = 0·030). |
| Chang, Yan et al, 2017^150^ | 13,952 | 13 | Cross-sectional | Higher socioeconomic status. | To investigate the prevalence of suicide attempts and explore the shared and unique factors influencing suicide risk in left-behind children and non-left-behind children in rural area. | They found that children with optimal family socioeconomic status are more likely to report SAs (OR = 1) than are those in the general children population, OR 0·52 (95% CI: 0·39–0·70). Children with higher mother’s education level are subject to higher suicide rates in high school, OR 1·67 (95% CI: 1·13–2.46), and post-secondary education, OR 2·14 (95% CI: 1·37–3·37). |
| Huerta Ramírez, 2017^151^ | 4,583 | 13 | Cross-sectional | Anxiety;  Depression;  Unemployed;  Younger. | To investigate prevalence of suicide in general adult population and looking for sociodemographic and clinical associated factors. | They found that associated factors were younger age, worse physical health status, absence of labor activity, absence of a couple, more subjective loneliness and comorbidity with anxiety and depressive disorders. |
| Carrieri et al, 2017^152^ | 3,022 | 13 | Cross-sectional | HIV. | To investigate major correlates of suicide risk in persons living with HIV, to help target individuals who would benefit from suicide risk screening and psychiatric care. | They found number of discrimination-related social contexts reported (1·39 [1·19; 1·61]), homelessness (4·87 [1·82; 13·02]), and loneliness (4·62 [3·06; 6·97]) were major predictors of suicide risk. |

Continue on the next page

**Continuing Table 2. Summary of studies included in the review of loneliness and suicidal behavior.**

| **Citation** | **Sample size** | **Quality** | **Design** | **Associated factor** | **Study aim** | **Relevant Result** |
| --- | --- | --- | --- | --- | --- | --- |
| Oppong Asante et al, 2017^153^ | 1,984 | 14 | Cross-sectional | Anxiety;  Bulling;  Food insecure. | To investigate the prevalence and risk and protective factors associated with suicide among senior high school. | They found that anxiety increases the odds of suicidal behavior, even after controlling for other variables. Loneliness increases the odds of suicidal behavior but after adjusting for other factors the odds remained for only suicidal plan. Being bullied, physically attacked, involved in a physical fight and food insecurity remained risk factors for suicidal behavior. |
| Zhang et al, 2016^256^ | 506 | 15 | Cross-sectional | Stress life events. | To investigate relationship between stress life events, loneliness, and suicidal ideation among university students, provide reference for the prevention and intervention of college student suicide. | They found that loneliness differed significantly by gender and parental relationship quality, and regression analysis showed that loneliness and stressful life events were significant risk factors for suicidal ideation. |
| Gunzelmann et al, 2016^154^ | 795 | 13 | Cross-sectional | Older. | To investigate the extent of suicidal ideation in the elderly and how it is related to hopelessness and loneliness. | They found that suicidal ideation was more frequent in older persons aged 80 years or older who were living without a partner and experiencing a high degree of loneliness and hopelessness. Hopelessness proved to be the only significant predictor variable that significantly correlated with loneliness. |
| Spino et al, 2016^155^ | 44 | 14 | Cross-sectional | Depression. | To investigate text-messaging as a method of enhancing social support and reducing symptoms of depression and loneliness in survivors bereaved by suicide. | They found that symptoms of depression and loneliness were assessed in relation to reported social support avail-  able to participants. |
| Stickley and Koyanagi, 2016^156^ | 7,403 | 14 | Cross-sectional | Mental disorders. | To investigate whether loneliness is associated with suicidal behavior in the general adult population and whether this relation varies by the level of loneliness; and to examine the role of common mental disorders in this association. | They found that higher levels of loneliness were still significantly associated with suicidal ideation and suicide attempts with OR for those in the most severe loneliness category ranging from 3·45 (lifetime suicide attempt) to 17·37 (past 12-month suicide attempt). |
| Li, Cai et al, 2016^157^ | 547 | 13 | Cross-sectional | Anxiety;  Depression;  Impulsivity;  Involuntary subordination. | To investigate men who have sex with men with suicidal ideation and provide suggestions for future suicide intervention programs among men who have sex with men and others. | They found that depression, anxiety, loneliness, impulsivity, involuntary subordination, social support, and risky sex were associated with suicidal ideation. |

Continue on the next page

**Continuing Table 2. Summary of studies included in the review of loneliness and suicidal behavior**

| **Citation** | **Sample size** | **Quality** | **Design** | **Associated factor** | **Study aim** | **Relevant Result** |
| --- | --- | --- | --- | --- | --- | --- |
| Heisel et al, 2016^300^ | 109 | 5 | Cohort | Depression. | To investigate the roles of reasons for living and meaning in life in potentially promoting mental  health and well-being and protecting against suicide ideation among community-residing older adults. | They found that meaning in life explained significant unique variance in suicide ideation, controlling for these factors and reasons for living, and meaning in life significantly mediated the association between reasons for living and suicide ideation. |
| Li, Xu et al, 2016^158^ | 15,957 | 14 | Cross-sectional | Depression;  Older;  Urban residence. | To investigate the prevalence of suicidal thoughts and attempts reported by older adults;  and identified personal, interpersonal, and environmental factors related to the two suicidal behaviors. | They found that older adults’ religious affiliation, depression, loneliness, and urban residence were significantly related to suicidal attempts during the different time periods. |
| Hiramitsu, 2015^301^ | 94,189 | 6 | Cohort | Age;  Gender;  Presence or absence of cohabitants. | To investigate the association between suicide deaths attributed to loneliness and the presence or absence of cohabitants, stratified by gender and age. | They found that suicide mortality due to loneliness was consistently higher among individuals living alone compared to those living with others across all age groups and genders, with the risk increasing with age, particularly among elderly men. Among all suicide deaths, the proportion attributed to loneliness was higher in those living alone (men: 3·5%, women: 5·3%) than in those living with others (men: 1·0%, women: 1·4%). |
| Saffer er al, 2015^159^ | 413 | 13 | Cross-sectional | Mental disorders | To investigate the association between parental bonding and histories of suicide ideation and attempts. | They found that adolescents with a history of suicide attempts scored significantly lower on both maternal and paternal care scales than adolescents with a history of suicide ideation but no history of attempts, t(143) = 4·19 and t(130) = 2·26, respectively. |
| Muyan and Chang, 2015^257^ | 288 | 15 | Cross-sectional | Depression;  Perfectionism. | To investigate perfectionism and loneliness as predictors of suicidal risk (viz., depressive symptoms & suicide ideation). | They found that loneliness and several dimensions of perfectionism—namely concern over mistakes, doubts about actions, parental expectations, and parental criticism—were positively associated with both depressive symptoms and suicidal ideation. |
| Zhang et al, 2014^258^ | 1,101 | 15 | Cross-sectional | Self consistency; Self inconsistency; Self-stereotype; Self-flexibility. | To investigate the situation of suicide ideation among college students，and to explore possible relations among self-consistency loneliness and suicide ideation for suicide prevention and intervention. | They found that self-consistency, self-inconsistency, and self-stereotype were positively correlated with loneliness and suicidal ideation, whereas self-flexibility was negatively correlated with both. Self-related dimensions significantly predicted loneliness and suicidal ideation, and loneliness mediated the association between self-consistency and suicidal ideation (accounting for 30% of the total effect). |

Continue on the next page

**Continuing Table 2. Summary of studies included in the review of loneliness and suicidal behavior**

| **Citation** | **Sample size** | **Quality** | **Design** | **Associated factor** | **Study aim** | **Relevant Result** |
| --- | --- | --- | --- | --- | --- | --- |
| He et al, 2014^259^ | 839 | 18 | Cross-sectional | Depressive symptoms. | To investigate the association between loneliness and suicidal ideation among rural  elderly. | They found that after controlling for demographics and life events, both loneliness (OR = 1·09, 95% CI 1·01–1·17) and depressive symptoms (OR = 1·19, 95% CI 1·12–1·26) were independently associated with suicidal ideation. |
| Pješčić et al, 2014^160^ | 212 | 14 | Cross-sectional | Depression;  Schizophrenia. | To investigate the influence of certain psychosocial factors – insight, psychoeducation, family and social support, loneliness, and social isolation – on the appearance of depression and suicidal risk in schizophrenia. | They found that group of patients with depression and suicidal risk, compared with the Control group, there was significantly higher frequency of insight in the mental status (χ^2^=31·736, p<0·001), number of patients without psycho-education (χ^2^=10·039, p=0·002), deficit of family support (χ^2^=13·359, p=0·001), deficit of social support (χ^2^=6·103, p=0·047), loneliness (χ^2^=6·239, p=0·012), and social isolation (χ^2^=47·218, p<0·001). |
| Shelef et al, 2014^260^ | 103 | 15 | Cross-sectional | Burdensomeness. | To investigate the interpersonal-psychological and cognitive-deficit models in relation to suicidal ideation, and to develop an alternative approach for assessing these thoughts and the distress connected to suicide risk. | They found that prior suicide attempt, loneliness and burdensomeness together explain 65% (P < 0·001) of the variance in suicidal ideation. |
| Lamis et al, 2014^161^ | 207 | 13 | Cross-sectional | Drug use. | To investigate the importance of drug use as a possible pathway for explaining, in part, the association between loneliness and suicidal ideation. | They found a significant indirect (mediated) effect of loneliness on suicidal ideation via drug use (*ab* = 0·09, 95% CI: 0·02–0·18), suggesting that loneliness may contribute to suicidal ideation through increased drug use among college students. |
| Sueki et al, 2014^311^ | 5,495 | 7 | Cohort | Anxiety;  Depression. | To investigate the impacts of suicide-related or mental health consultation-related internet use. | They found that internet users who had employed the internet for suicide-related or mental health consultation-related reasons at T0 (n = 2813), compared with those who had not (n = 2682), showed a significant increase in suicidal ideation (b = 0·38, 95%CI: 0·20–0·55) and depression/anxiety (b = 0·37, 95%CI: 0·12–0·61) from T1 to T2. Those who disclosed their own suicidal ideation and browsed for information about suicide methods on the web showed increased suicidal ideation (b = 0·55,  95%CI: 0·23–0·88; b = 0·45, 95% CI: 0·26–0·63, respectively). Although mental health consultation with an anonymous other online did not increase suicidal ideation, increased depression/anxiety was observed (b = 0·34, 95%CI: 20·03–0·71). |

Continue on the next page

**Continuing Table 2. Summary of studies included in the review of loneliness and suicidal behavior**

| **Citation** | **Sample size** | **Quality** | **Design** | **Associated factor** | **Study aim** | **Relevant Result** |
| --- | --- | --- | --- | --- | --- | --- |
| Mushtaq et al, 2014^24^ | - | 11 | Review | Cognitive decline. | To review associated factors with loneliness. | They found that research on suicide has revealed that there is a strong association between suicide ideation, parasuicide and loneliness. The prevalence of suicide ideation and parasuicide rises with the degree of loneliness. Further the peak season for loneliness has been reported to be winter and spring, the same season for which peak incidence of suicide has been reported. However, there is minimal differences in suicide between men and women related to loneliness. It was attributes loneliness as an important factor in etiology of suicide and parasuicide. It also considers loneliness as a disease and wants its place in classification of psychiatric disorders. |
| Bonnewyn et al, 2014^322^ | 8 | 20 | Qualitative | Older. | To investigate the reflections of older adults on the process preceding their suicide attempt. | They found the self as disrupted after experiencing a loss, loneliness, loss of control, and unwillingness to continue living the current life. |
| Randall et al, 2014^162^ | 2,690 | 14 | Cross-sectional | Anxiety;  Bulling;  Alcohol abuse;  Drug use. | To investigate the prevalence of suicide attempts and investigate the association between demographic, psycho-social and socio-environmental risk factors, and suicide attempts among school youth. | They found that anxiety, loneliness, being bullied, alcohol misuse, illicit drug use, and lack of parental support were independently related to the ideation outcomes, suicidal ideation without planning and suicidal ideation with planning. |
| Gallagher et al, 2014^302^ | 144 | 5 | Cohort | Social anxiety. | To investigate social anxiety as a unique predictor of adolescent suicidal ideation. | They found that loneliness may be particularly implicated in the relationship between social anxiety and suicidality in teens. |
| Bromberg et al, 2014^163^ | 281 | 11 | Cross-sectional | Chronic pain. | To investigate rates and identify predictors of suicidal ideation in adolescents with and without chronic pain. | They found that higher functional disability (*B* = 0·06) and loneliness (*B* = 0·10) and lower self-worth (*B* = -0·90, p’s <0·05) predicted the presence of suicidal ideation in chronic pain patients. |
| Kim, 2014^323^ | 35 | 20 | Qualitative | Chronic pain;  Depression. | To investigate life experiences following suicide attempts from the perspective of older adults. | They found that to prevent suicide in older adults, it's important to help them find meaning through crisis management, family support, and appropriate medication. Addressing depression, chronic pain, and loneliness, along with regular health assessments, can reduce repeated attempts. |

Continue on the next page

**Continuing Table 2. Summary of studies included in the review of loneliness and suicidal behavior**

| **Citation** | **Sample size** | **Quality** | **Design** | **Associated factor** | **Study aim** | **Relevant Result** |
| --- | --- | --- | --- | --- | --- | --- |
| Narishige et al, 2014^261^ | 193 | 15 | Cross-sectional | Mental disorders. | To investigate gender differences of precipitating factors for suicide attempts. | They found that rate of subjects diagnosed with “major depressive disorder, bipolar disorder” was significantly higher in males while that of subjects diagnosed with “personality disorders” or “dysthymic disorder” was significantly higher in females. Subjects with “health problems”, “financial problems”, “work problems”, “debts (others)” or “unwanted transfer” were significantly more numerous among males; subjects with “family problems”, “parent–child relations” or “loneliness” were significantly more frequently found among females. |
| Miret et al, 2014^262^ | 4,583 | 15 | Cross-sectional | Alcohol abuse;  Mental disorders. | To investigate current prevalence of suicidal  ideation and attempts in the general population in Spain, to compare it with the prevalence found before the economic crisis, and to analyze the factors associated with suicidality in different age groups. | They found that mental disorders presented the highest significant effects on lifetime suicidal ideation. Marital status, heavy alcohol consumption, and occupational status were associated with lifetime suicidal ideation in people aged 18–49, whereas loneliness was associated with the 50–64 group, and financial problems with the 65+ group. A younger age, poor health status and the presence of depression were all associated with lifetime suicide attempts. |
| Altangerel et al, 2014^164^ | 5,148 | 14 | Cross-sectional | Sleep problem. | To investigate the prevalence and predictors of suicidal behavior among high school students. | They found that students who had no close friends were 2·45 times as likely (OR = 2·45; 95 % CI 1·86–3·23) to think about suicide as those who have 1 or more close friends. Students who felt lonely were 2·3 times as likely (OR = 2·30; 95 % CI 1·94–2·73) to think about suicide as those who did not feel lonely over the past 30 days. Students who had sleeping problems were 2·2 times as likely (OR = 2·28; 95 % CI 1·96–2·65) to think about suicide as those who slept well. |
| Liu J et al,2013^263^ | 1,815 | 16 | Cross-sectional | Anxiety; Depression;  Introversion;  Lack of energy during daytime; Neuroticism; Psychoticism;  Sleep problem; | To investigate the personal risk factors on suicidal ideation and their affective path in junior high students． | They found the personal risk factors of suicidal ideation in junior high students included depression, anxiety, neuroticism, psychoticism, introversion, attitude towards suicide, attitude towards victim of suicide and their relatives, emotional and social loneliness, difficulty to fall sleep，lack of energy during daytime, etc. The influences of these risk factors on suicidal ideation might be direct or indirect． |

Continue on the next page

**Continuing Table 2. Summary of studies included in the review of loneliness and suicidal behavior**

| **Citation** | **Sample size** | **Quality** | **Design** | **Associated factor** | **Study aim** | **Relevant Result** |
| --- | --- | --- | --- | --- | --- | --- |
| Liu C et al,2013^264^ | 830 | 16 | Cross-sectional | Specialty;  Sibling status (associated factors) | To investigate the status of suicide idea and loneliness of medical students and their relationship． | They found that suicidal ideation differed significantly across specialties and was more common among students with siblings, and students with suicidal ideation had higher loneliness scores. |
| Saito, 2013^329^ | 229 | 11 | Review | Lack of social ties | To review issues associated with social isolation among older adults and discuss future directions for interventions. | They found a lack of social ties: In the Finnish study, among suicide victims:  - 42·5% had no friends sharing hobbies.  - 37·9% reported feelings of loneliness.  - 25·3% had no close opposite-sex partner. |
| Schinka et al, 2013^303^ | 832 | 5 | Cohort | Depression. | To investigate how experiences of loneliness emerge in distinct developmental patterns over the course of middle childhood and adolescence. | They found ethnicity, income, age 7 social skills, age 7 depression, and age 7 aggression were associated with loneliness trajectory. |
| Salvo and Castro, 2013^165^ | 763 | 13 | Cross-sectional | Alcohol abuse;  Impulsivity. | To investigate the predictive value of loneliness, impulsivity, and alcohol use on suicidal behavior in adolescents. | They found that loneliness, impulsivity, and alcohol use were directly related to suicidal behavior. |
| Levi-Belz et al, 2013^166^ | 102 | 13 | Cross-sectional | Mental pain. | To investigate whether both anxious and avoidant attachment patterns would be associated with a greater likelihood to engage in more lethal suicidal behavior with more serious intent. | They found that the psychological mechanisms of medically serious suicide behavior involve high levels of mental pain amplified by insecure attachment patterns and interpersonal difficulties. |
| Yang et al, 2012^265^ | 1,122 | 15 | Cross-  sectional | Depression levels. | To investigate high school students’ attitudes toward suicide and explore the correlations between suicide attitudes, loneliness, and depressive emotions, to provide an evidence base for suicide prevention and mental health interventions. | They found that correlation analysis revealed that greater loneliness and depression were linked to more tolerant attitudes toward suicide and euthanasia, while depression was associated with more negative views of suicide victims’ families. Regression results indicated that social loneliness predicted tolerance toward suicide behavior, emotional loneliness predicted tolerance toward euthanasia, and depression predicted negative attitudes toward victims’ families. |

Continue on the next page

**Continuing Table 2. Summary of studies included in the review of loneliness and suicidal behavior**

| **Citation** | **Sample size** | **Quality** | **Design** | **Associated factor** | **Study aim** | **Relevant Result** |
| --- | --- | --- | --- | --- | --- | --- |
| Li H et al, 2012^167^ | 449 | 13 | Cross-  sectional | Depression. | To investigate the relationship between loneliness and suicidal ideation among Chinese college students, and to test whether loneliness independently predicts suicidal ideation after controlling for depression. | The study found that loneliness was significantly and positively correlated with both depression (r = 0·29, p < 0·01) and suicidal ideation (r = 0·27, p < 0·01). Even after controlling for depression, loneliness remained a significant independent predictor of suicidal ideation (β = 0·20, p < 0·001), indicating its unique contribution to suicidal risk. Furthermore, participants with high loneliness scores demonstrated a significantly stronger implicit association between “self” and “death” (F = 7·58, p < 0·01) compared with those with low loneliness. The consistency between implicit and explicit measures suggests that loneliness serves as an independent and sensitive indicator of suicidal ideation beyond the effect of depression. |
| Hirsch et al, 2012^168^ | 385 | 13 | Cross-  sectional | Stress. | To investigate the combined moderating effects of life stress and loneliness on the association between social problem-solving ability and suicidal behaviors. | They found that loneliness increases the strength of the association between poor problem-solving and suicidal behaviors. |
| Chen, Fettich, Tierney et al, 2012^169^ | 334 | 14 | Cross-  sectional | Depression;  Hopelessness;  Lack of college education;  Psychological distress;  Sexual abuse. | To investigate the factors associated with current suicide ideation in severely obese bariatric surgery-seeking individuals. | They found that lack of college education, a history of suicide ideation and/or behavior, psychological distress, hopelessness, loneliness, history of physical and/or sexual abuse, and lifetime major depression were associated with current suicide ideation. |
| Chen, Fettich and McCloskey, 2012^170^ | 396 | 13 | Cross-  sectional | Weight-related stigma. | To investigate whether weight-related stigma increases the likelihood of suicidal behavior or the degree of loneliness. | They found that for severely obese surgery-seeking women, weight-related stigma was associated with suicidal behavior, though this was not mediated by loneliness. |
| Schinka et al, 2012^304^ | 937 | 5 | Cohort | Adolescent. | To investigate how loneliness in middle childhood and adolescence is related to suicidal thoughts and suicidal behaviors cross-sectionally and longitudinally. | They found that loneliness at age 15 years was concurrently associated with both suicidal ideation and suicidal behavior at age 15 years. Loneliness in middle childhood was longitudinally associated with suicidal behaviors at age 15 years, but not with suicidal ideation at age 15 years. |

Continue on the next page

**Continuing Table 2. Summary of studies included in the review of loneliness and suicidal behavior**

| **Citation** | **Sample size** | **Quality** | **Design** | **Associated factor** | **Study aim** | **Relevant Result** |
| --- | --- | --- | --- | --- | --- | --- |
| Creemers et al, 2012^171^ | 95 | 14 | Cross-  sectional | Depression. | To investigate whether explicit and implicit self-esteem, the interaction between these two constructs, and their discrepancy are associated with depressive symptoms, suicidal ideation, and loneliness. | They found that explicit but not implicit self-esteem was negatively associated with depressive symptoms, suicidal ideation, and loneliness. |
| Wilson et al, 2012^172^ | 1,427 | 13 | Cross-  sectional | Depression;  Tabaco. | To investigate correlates for suicidal expression among adolescents. | They found that those reporting suicidal ideation were younger (relative risk ratio RRR= 0·81; CI = 0·68–0·96), indicated signs of depression (RRR= 1·69; CI = 1·05–2·72) and loneliness (RRR=3·36; CI =1·93–5·84). Tobacco use (RRR = 2·34; CI = 1·32–4·12) and not having close friends (RRR = 3·32; CI = 1·54–7·15) were significantly associated with suicidal ideation. Those with suicidal ideation/planning were more likely to be female (RRR = 0·47; 0·30–0·74), anxious (RRR= 3·04; CI = 1·89–4·88) and lonely (RRR= 1·74; CI= 1·07–2·84). |
| Swahn et al, 2012^266^ | 457 | 15 | Cross-  sectional | Alcohol abuse;  Food insecure;  Parental problems;  Trading sex. | To investigate the prevalence and correlates of suicidal behavior. | They found that reporting both parents deceased aOR = 2·36; 95% CI: 1·23–4·52), parental neglect due to alcohol use (aOR = 2·09; 95% CI: 1·16–3·77), trading sex for food, shelter or money (aOR = 1·95; 95% CI: 1·09–3·51), sadness (aOR = 2·42; 95% CI: 1·20–4·89), loneliness (aOR = 2·67; 95% CI: 1·12-6·40) and expectations of dying prior to age 30 (aOR = 2·54; 95% CI: 1·53–4·23) were significantly associated with suicide ideation in multivariate analyses. |
| Jones et al, 2011^305^ | 889 | 5 | Cohort | Depression. | To investigate whether changes in loneliness during middle childhood as well as from middle childhood into adolescence were associated with adolescent self-harm behaviors and suicidal thoughts. | They found that increases in loneliness indirectly affect self-harm behaviors and suicidal thoughts through depression and externalizing behavior problems. |
| Cui et al, 2011^173^ | 8,778 | 13 | Cross-  sectional | Bulling. | To investigate the association between peer relationships and suicide ideation and attempts among adolescents; and whether such associations were moderated or mediated by feeling of loneliness. | They found that specific problems in peer relationships, such as lack of peer association and being victimized by bullying, were significantly related to suicide ideation and attempts. |

Continue on the next page

**Continuing Table 2. Summary of studies included in the review of loneliness and suicidal behavior**

| **Citation** | **Sample size** | **Quality** | **Design** | **Associated factor** | **Study aim** | **Relevant Result** |
| --- | --- | --- | --- | --- | --- | --- |
| Lasgaard et al, 2011^281^ | 541 | 4 | Cohort | Depression. | To investigate the relationship between loneliness, depressive symptoms, and suicide ideation in adolescence. | They found that loneliness was a correlate of depressive symptoms at the cross-sectional level, independent of gender, other demographic factors, multiple psychosocial variables, and social desirability. Loneliness did not predict suicide ideation over time or at the cross-sectional level, when controlling for depressive symptoms. |
| Carvajal and Caro, 2011^174^ | 482 | 14 | Cross-  sectional | Depression;  Low self-esteem. | To investigate the relationship between hopelessness, loneliness, and the degree of family health in school-aged adolescents, with and without suicidal ideation. | They found that variables that best explain the presence of suicidal ideation are: history of suicide attempt, low self-esteem, depression, and belonging to an unhealthy family. |
| Chang et al, 2010^175^ | 160 | 13 | Cross-  sectional | Hopelessness. | To investigate loneliness and negative life events as predictors of suicide risk (viz., hopelessness and suicidal behaviors). | They found loneliness and negative life events to be positively associated with both hopelessness and suicidal behaviors. |
| Su et al, 2009^176^ | 10,235 | 13 | Cross-  sectional | Gender; Depression;  High study pressure; Insomnia;  Feeling unsafe to/from school. | To investigate the prevalence and associated factors of suicidal ideation among adolescents in Fujian Province, and to provide evidence for preventive interventions. | They found that multivariate logistic regression confirmed depression, loneliness, stress, insomnia, and perceived insecurity as significant predictors.  Recommendations: strengthen psychological education, reduce study pressure, enhance family, and school support. |
| van Bergen et al, 2008^177^ | 249 | 13 | Cross-  sectional | Low self-esteem. | To investigate the prevalence and explored the vulnerability to suicidal ideation across several ethnic minority versus ethnic majority adolescents. | They found that lack of self-pride and the idea of not becoming successful in life, appeared to be important, as well as feelings of loneliness. |
| Kidd and Shahar, 2008^178^ | 208 | 13 | Cross-  sectional | Low self-esteem. | To investigate the protective role of self-esteem, social involvement, and secure attachment among homeless youths. | They found that self-esteem as a protective factor, predicting levels of loneliness, feeling trapped, and suicide ideation. |
| Rudatsikira et al, 2007^179^ | 1,506 | 14 | Cross-  sectional | Alcohol abuse;  Bulling;  Tabaco. | To investigate the prevalence and associated factors of suicidal ideation among school going adolescents. | They found that loneliness, worry were positively associated with suicide ideation after adjusting for age, gender, smoking, drinking, and experience of having been bullied (OR = 1·59; 95% CI [1·12, 2·26] and OR = 1·19; 95% CI [1·12, 2·25]) respectively. |

Continue on the next page

**Continuing Table 2. Summary of studies included in the review of loneliness and suicidal behavior**

| **Citation** | **Sample size** | **Quality** | **Design** | **Associated factor** | **Study aim** | **Relevant Result** |
| --- | --- | --- | --- | --- | --- | --- |
| Hendin et al, 2007^180^ | 36 | 13 | Cross-  sectional | Depression. | To investigate the extent to which intense affective states in depressed patients may signal a suicide crisis, i.e., an acute risk for suicide. | They found that suicide patients had a significantly greater number of intense affects than did the comparison patients. |
| Conner et al, 2007^282^ | 73 | 3 | Cohort | Drug use. | To investigate the role of three theoretically important interpersonal variables in attempted suicide and unintentional overdose using a diverse methadone patients. | They found the relevance of a sense of belonging to vulnerability to suicidal behavior, and lend further support to the notion that suicide attempts and unintentional overdose have dissimilar correlates. |
| Xu et al,2006^181^ | 912 | 14 | Cross-sectional | Negative life events;  Neighbor relationships; Depressive symptoms; Hopelessness. | To investigate the prevalence of suicidal ideation and identify major psychosocial and demographic risk factors among rural elderly in Yuanjiang, China. | Multivariate logistic regression analysis identified several significant risk factors, including negative life events (OR≈2·3), poor neighbor relationships (OR≈2·1), depressive tendency (OR≈1·9), hopelessness (OR≈2·6), and loneliness (OR≈1·8). The study concluded that suicidal ideation is relatively high among rural elderly individuals, and effective interventions should focus on strengthening social support networks, improving emotional well-being, and providing timely psychological care and follow-up after major life events. |
| Otsuka et al, 2006^182^ | 561 | 13 | Cross-sectional | Economic problems;  Family problems;  Health problems;  Work-related problems. | To investigate the living conditions of suicide victims, awareness of depression and suicide, and the actual situation of suicide attempters in psychiatric emergency care in the Kuji region of Iwate Prefecture, with a particular focus on elderly suicide. | They found that despite most elderly suicide attempters living with others, more than half had not consulted anyone regarding their psychological problems before attempting suicide. Family members accounted for most consultation sources. Furthermore, a large proportion (57%) of elderly attempters were diagnosed with mood disorders. |
| Grøholt et al, 2005^183^ | 455 | 13 | Cross-  sectional | Depression;  Low self-esteem. | To investigate differences between risk factors among suicidal adolescents and nonsuicidal adolescents by focusing on self-esteem. | They found that variance in self-esteem was explained by depression and loneliness, and among nonsuicidal adolescents also by self-concept stability, support, and competencies. Loneliness and self-concept stability related differently to self-esteem in suicidal and nonsuicidal adolescents. |
| Chou et al, 2005^184^ | 154 | 13 | Cross-  sectional | Depression;  Hopelessness. | To investigate the psychometric properties of the Chinese version of the Geriatric Suicide Ideation Scale. | They found convergent relation between depression, loneliness, hopelessness, and suicidal behavior. |

Continue on the next page

**Continuing Table 2. Summary of studies included in the review of loneliness and suicidal behavior**

| **Citation** | **Sample size** | **Quality** | **Design** | **Associated factor** | **Study aim** | **Relevant Result** |
| --- | --- | --- | --- | --- | --- | --- |
| Durak Batigün, 2005^185^ | 683 | 13 | Cross-  sectional | Hopelessness;  Younger. | To investigate the reasons that hold people clinging to life; their suicide probability and to describe the relationship of these variables with other variables such as hopelessness and loneliness, taking age, education, and other socioeconomic variables into consideration. | They found that the group aged between 15-25 years reported fewer reasons for living, higher suicide probability, more that the group aged between 15-25 years reported fewer reasons for living, higher suicide probability, more hopelessness and loneliness, compared to older ages. and loneliness, compared to older ages. |
| Fujioka et al, 2004^186^ | 420 | 14 | Cross-sectional | Age;  Economic problems;  Family problems;  Gender;  Health problems;  History of previous suicide attempts;  Work-related problems. | To investigate the actual situation of suicide and to discuss future directions for suicide prevention. | They found that male suicides (292 cases) were more than twice as common as female suicides (128 cases). Male suicides were most frequent among middle-aged and older adults (30s–70s), while female suicides were concentrated in the elderly (50s–80s). Suicides occurred significantly more often in spring than in other seasons. About 70% experienced some form of psychosocial stress before death, and 23% had a history of suicide attempts. Behavioral changes before suicide included depression (14%), deviant behavior (11%), complaints of physical illness (5%), and social isolation (4%). |
| Lunsky, 2004^187^ | 98 | 13 | Cross-  sectional | Anxiety;  Depression. | To investigate suicidal ideation in a combined sample of adults with mental retardation from community and clinical settings, according to self-report, informant ratings, and clinical charts when feasible. | They found that individuals who reporting suicidal ideation endorsed more loneliness, stress, anxiety, and depression, along with less social support than other individuals, consistent with reports of suicidal individuals in the general population. |
| Langlo et al, 2003^188^ | 92 | 13 | Cross-  sectional | Depression;  Low self-esteem. | To investigate association of suicidal behavior in younger. | They found association with lower self-esteem, more loneliness, and more frequent depressive feelings (p < 0·01). |
| Aydemir et al, 2002^83^ | 60 | 5 | Case-control | Depression. | To investigate the cognitive and emotional structures of patients who attempted suicide with those diagnosed with major depression. | They found suicide group had high depressive symptomatology. |
| Dieserud et al, 2001^189^ | 123 | 13 | Cross-  sectional | Depression;  Low self-esteem;  Unmarried. | To investigate association between depression, low self-esteem, hopelessness, interpersonal problems, and loneliness with suicidal behavior. | They found that low self-esteem, loneliness, and separation or divorce, which advanced to depression, and  was further mediated by hopelessness and suicidal ideation which led to suicide attempt. |

Continue on the next page

**Continuing Table 2. Summary of studies included in the review of loneliness and suicidal behavior**

| **Citation** | **Sample size** | **Quality** | **Design** | **Associated factor** | **Study aim** | **Relevant Result** |
| --- | --- | --- | --- | --- | --- | --- |
| Stravynski and Boyer, 2001^190^ | 19,724 | 13 | Cross-  sectional | Men. | To investigate the association between loneliness and suicidal behavior. | They found strong associations among suicide ideation, parasuicide and different ways of being lonely and alone, defined either subjectively (i.e., the feeling), or objectively (i.e., living alone or being without friends). |
| Guertin et al, 2001^84^ | 95 | 5 | Case-control | Depression;  Dysthymia;  Hopelessness;  Oppositional defiant disorder. | To investigate the cognitive/affective and behavioral symptoms of adolescent suicide attempters with self-mutilative behavior. | They found that self-mutilative behavior group was significantly more likely to be diagnosed with oppositional defiant disorder, major depression, and dysthymia and had higher scores on measures of hopelessness, loneliness, anger, risk taking, reckless behavior, and alcohol use than did the non- self-mutilative behavior group. |
| Grøholt et al, 2000^191^ | 1,968 | 13 | Cross-  sectional | Alcohol abuse;  Depression;  Disruptive disorders;  Low socioeconomic status;  Low self-worth. | To investigate risk factors for self-harm in 2 groups: hospitalized adolescents who had attempted suicide and adolescents reporting suicide attempts. | They found for suicide attempts in hospitalized adolescents were depression (odds ratio [OR] =4·7), disruptive disorders (OR =9·4), low self-worth (OR =1·3), infrequent support from parents (OR =3·3) or peers (OR =3·3), parents' excessive drinking (OR =4·3), and low socioeconomic status (OR =2·4). For adolescents who self-reported self-harm, depression (OR =3·1) and loneliness (OR =1·13) were significant adjusted. risk factors (p < 0·001). |
| Magne-Ingvar and Ojehagen, 1999^192^ | 81 | 14 | Cross-  sectional | Mental disorder. | To investigate whether information from a significant other would be helpful in the psychiatric assessment of the patient, and in assessing the well-being of significant others and their need for support. | They found association between loneliness and suicidal behavior. |
| Abe et al, 1998^193^ | 83 | 13 | Cross-sectional | Economic problems;  Health problems;  Mental disorders | To investigate the behavioral patterns, mental states, family environments, and welfare service utilization of elderly individuals who died by suicide, and to consider how welfare services can be structured to contribute to the prevention of elderly suicide. | They found that among the 83 elderly individuals who died by suicide, 62 (74·7%) exhibited physical symptoms such as loss of appetite and sleep disturbances, and 63 (75·9%) were in a state of loneliness or social isolation, characterized by staying indoors and having minimal contact with others. Only 3 individuals (3·6%) were living alone. |

Continue on the next page

**Continuing Table 2. Summary of studies included in the review of loneliness and suicidal behavior**

| **Citation** | **Sample size** | **Quality** | **Design** | **Associated factor** | **Study aim** | **Relevant Result** |
| --- | --- | --- | --- | --- | --- | --- |
| Roberts et al, 1998^194^ | 5,423 | 13 | Cross-  sectional | Depression. | To investigate the risk of suicidal plans and ideation, depression, and other factors (low self-esteem, loneliness, fatalism, pessimism) among adolescents with a lifetime history of attempted suicide. | They found associated with current suicidal thinking were history of attempts (odds ratio [OR] = 3·50), depression (OR = 5·34), and recent life stress (OR = 2·64). |
| Joiner and Rudd, 1996^195^ | 234 | 13 | Cross-  sectional | Hopelessness. | To investigate the interrelations between hopelessness, loneliness, and suicidal ideation, by comparing two conceptually driven models of their relationships. | They found that hopelessness as a source variable that is predictive of both loneliness and suicidality. |
| Hattori et al, 1995^312^ | 628 | 7 | Cohort | Death or severe disease of family member;  Distress by severe illness;  Divorce or loss of love;  Females losing a spouse;  Intrafamilial conflicts;  Males living alone;  Psychiatric diagnosis. | To investigate demographic and diagnostic characteristics of psychiatric patients with suicidal behavior in a general hospital setting; psychosocial factors associated with suicidal behavior; the role of consultation-liaison psychiatry in emergency medicine. | They found that suicide rate among psychiatric patients was 82·6/100,000/year, about 3–4 times higher than general population rates. Male patients were more likely to complete suicide, females more likely to attempt without completing. Schizoaffective disorder and depressive disorders were significantly associated with completed suicide; schizophrenia, depressive disorders, and adjustment disorders with attempted suicide. Among psychiatric patients who attempted suicide, 15% experienced loneliness from living alone, 24% faced intrafamilial conflicts, and 16% had lost a loved one through divorce or separation. Among referred patients, 16% experienced loneliness, 26% reported loss of love or divorce, and 16% had intrafamilial conflicts. |
| Garnefski et al 1992^85^ | 570 | 5 | Case-control | Depression;  Drugs use;  Low self-esteem. | To investigate differences between attempters and nonattempters is the matched control design. | They found that the experience of sexual abuse, feelings of loneliness, depressed mood, low self-esteem, and the use of drugs were particularly strongly related to suicidal thoughts and behavior. |
| Bonner and Rich, 1987^196^ | 158 | 13 | Cross-  sectional | Depression;  Hopelessness. | To investigate self-report measures of life stress, faulty cognitions, loneliness, depression, hopelessness, family cohesiveness, adaptive reasons for living, and suicidal ideation and behavior. | They found association between loneliness and suicidal behavior. |
| Wenz, 1977^197^ | 110 | 11 | Cross-  sectional | Seasonality. | To investigate the relation of two components of loneliness, present and future loneliness, to seasonality of suicide attempts. | They found that mean scale scores for present and future loneliness were greatest for spring and winter, the peak seasons for the timing of suicide attempts. |
