# Supplementary material for: Loneliness as an Interface Between Alzheimer's Disease and Suicidal Behaviour: A Systematic Review, Meta‐Analysis and Meta‐Analytic Factor Analysis
Source: Psychogeriatrics. 2026 Apr 7;26(3):e70165. doi: 10.1111/psyg.70165 (PMC13055150; doi:10.1111/psyg.70165)
Supplement: Supplementary file 3 — Data S1: Supplementary statistics loneliness and AD‐SB. [file PSYG-26-0-s003.docx]

**Supplementary Statistics loneliness and AD-SB**

**Graphic 1. Accumulative risk of developing AD due to loneliness.**


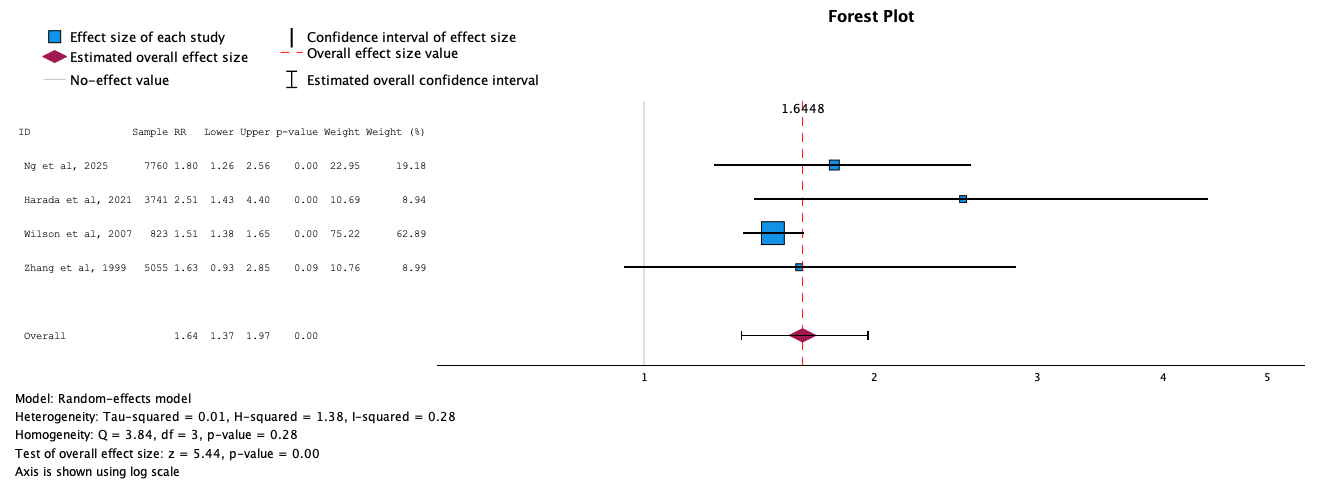

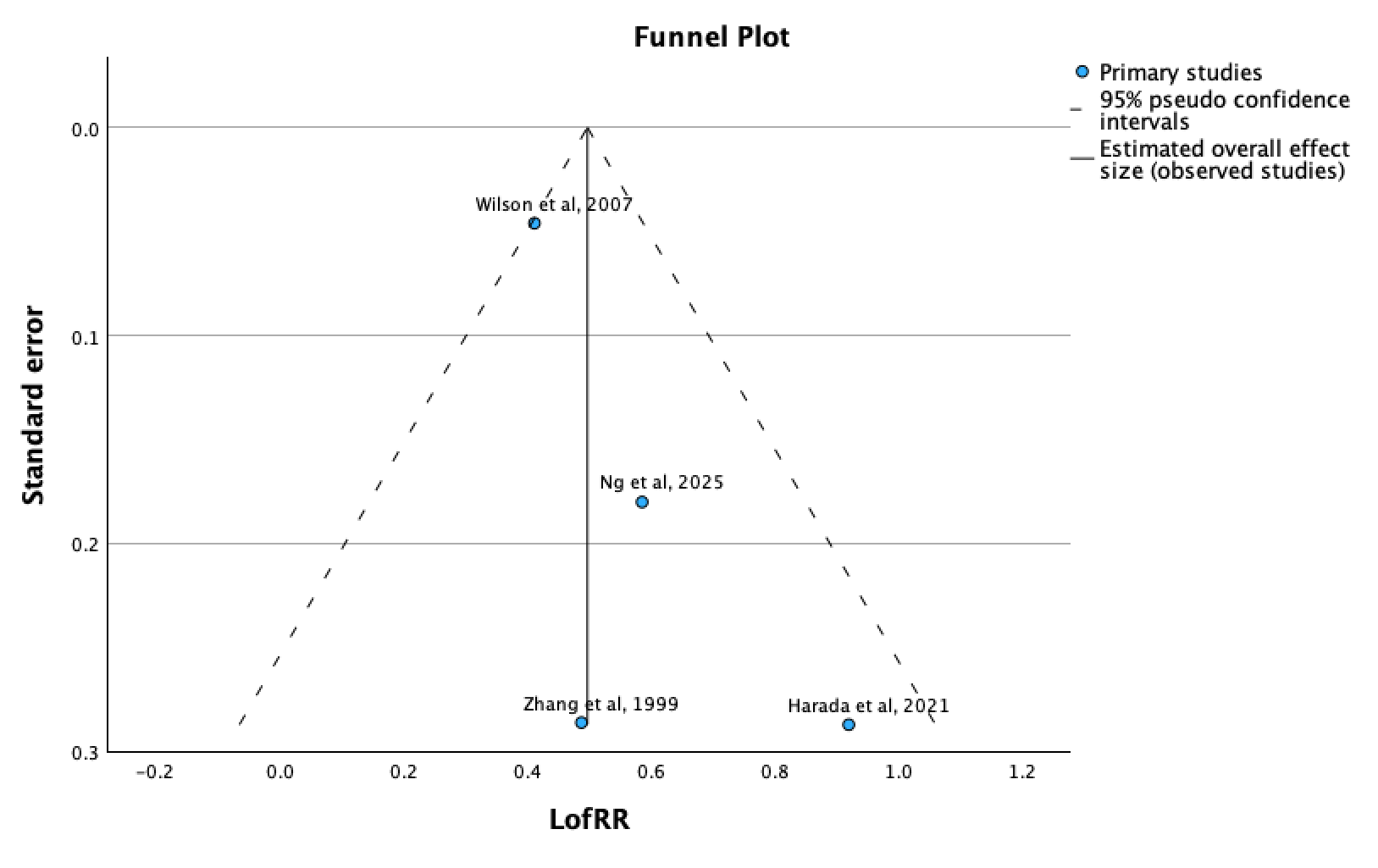

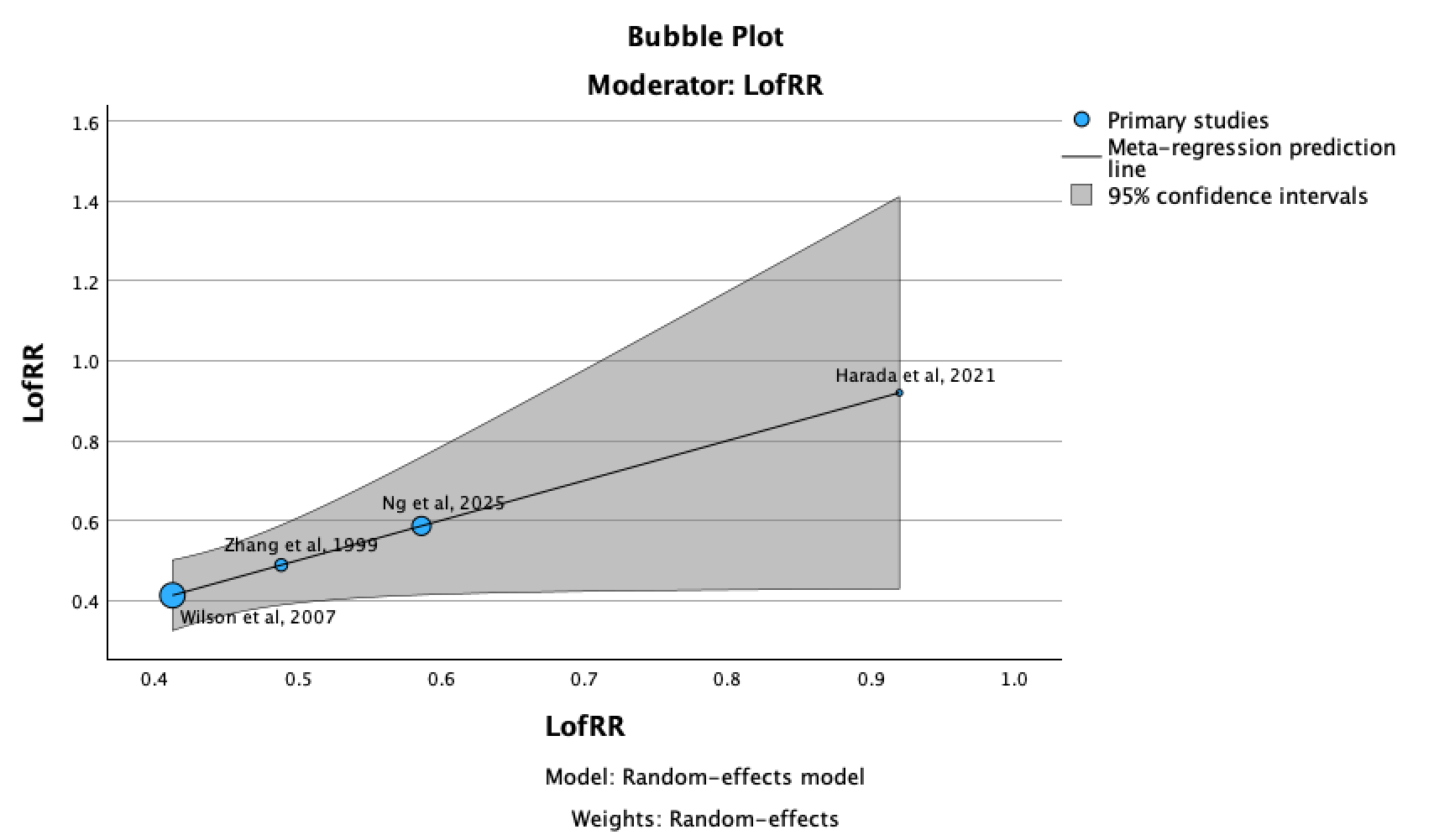


| **Egger's Regression-Based Test^a^** | | | | | | |
| --- | --- | --- | --- | --- | --- | --- |
| Parameter | Coefficient | Std. Error | t | Sig. (2-tailed) | 95% Confidence Interval | |
|  |  |  |  |  | Lower | Upper |
| (Intercept) | ·355 | ·0654 | 5·437 | ·032 | ·074 | ·637 |
| SE^b^ | 1·236 | ·7508 | 1·646 | ·241 | -1·994 | 4·466 |
| a. Random-effects meta-regression | | | | | | |
| b. Standard error of effect size | | | | | | |

| **Effect Size Estimates for Trim-and-Fill Analysis** | | | | | | | |
| --- | --- | --- | --- | --- | --- | --- | --- |
|  | Number | Effect Size | Std. Error | Z | Sig. (2-tailed) | 95% Confidence Interval | |
|  |  |  |  |  |  | Lower | Upper |
| Observed | 4 | ·498 | ·0914 | 5·442 | <·001 | ·318 | ·677 |
| Observed + Imputed^a^ | 6 | ·414 | ·0419 | 9·880 | <·001 | ·332 | ·496 |
| 1. Number of imputed studies: 2 | | | | | | | |

Egger’s Test demonstrates risk of bias [CI: 0·74 to 0·637]. The Trim-and-Fill analysis found a difference in effect size between the observed and the observed plus imputed groups in a single additional hypothetical study and identified a risk of bias. No study demonstrated funnel plot asymmetry. The bubble plot showed significant dispersion in one study, which was removed for reanalysis.^62^ After adjustment, the pooling data demonstrated low heterogeneity, with a RR of 1·53 [CI: 1·40-1·67; p<0·01; *I*^2^ = 0%; z = 9·63] (Supplementary Graphic 1a).

**Graphic 1a. Accumulative risk of developing AD due to loneliness after adjusting for bias.**


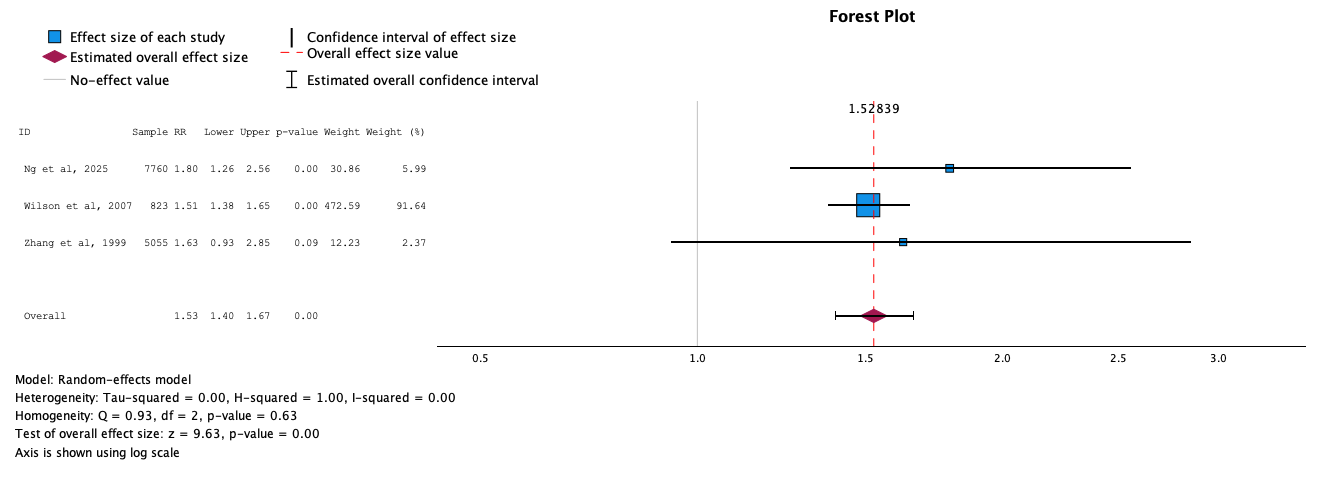

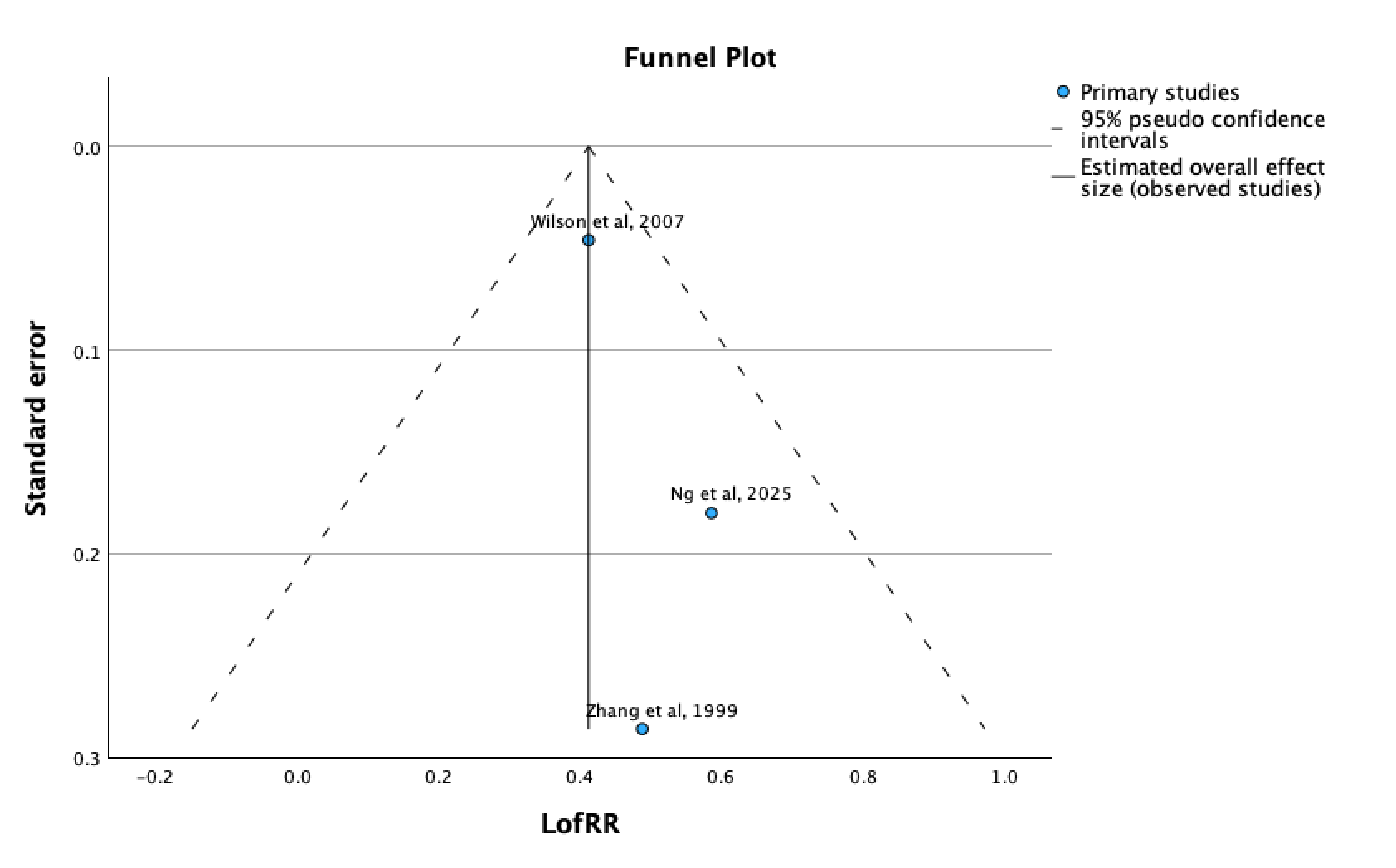


| **Egger's Regression-Based Test^a^** | | | | | | |
| --- | --- | --- | --- | --- | --- | --- |
| Parameter | Coefficient | Std. Error | t | Sig. (2-tailed) | 95% Confidence Interval | |
|  |  |  |  |  | Lower | Upper |
| (Intercept) | ·380 | ·0708 | 5·372 | ·117 | -·519 | 1·279 |
| SE^b^ | ·737 | ·9279 | ·795 | ·573 | -11·053 | 12·527 |
| a. Random-effects meta-regression | | | | | | |
| b. Standard error of effect size | | | | | | |

Egger’s Test did not demonstrate risk of bias [CI: -0·519 to 1·279]. No study demonstrated funnel plot asymmetry.

**Graphic 2. Immediate risk of developing AD due to loneliness.**


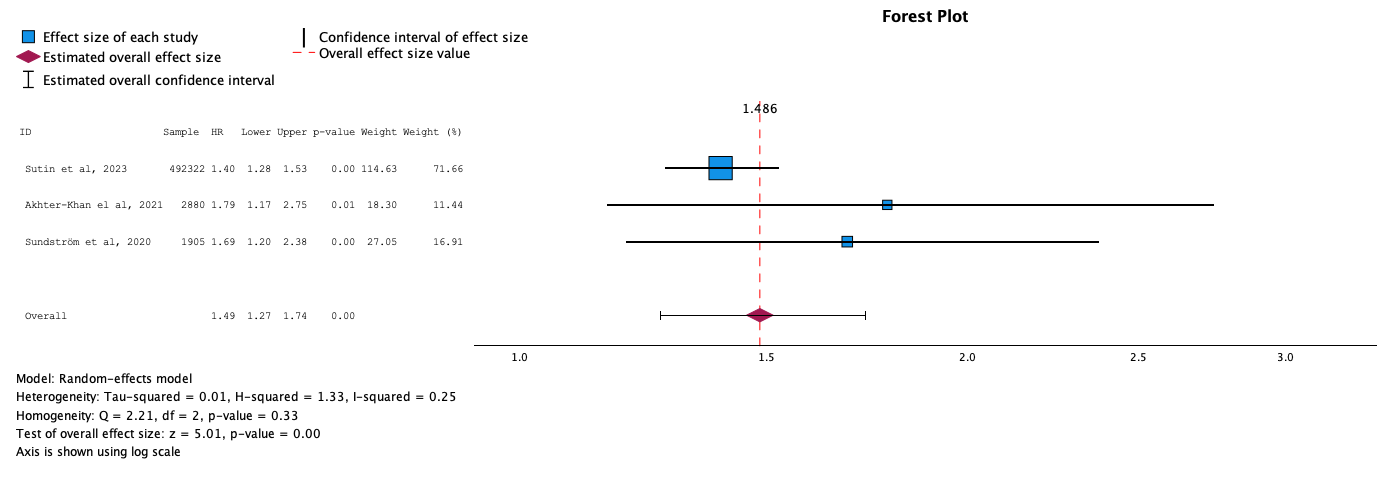

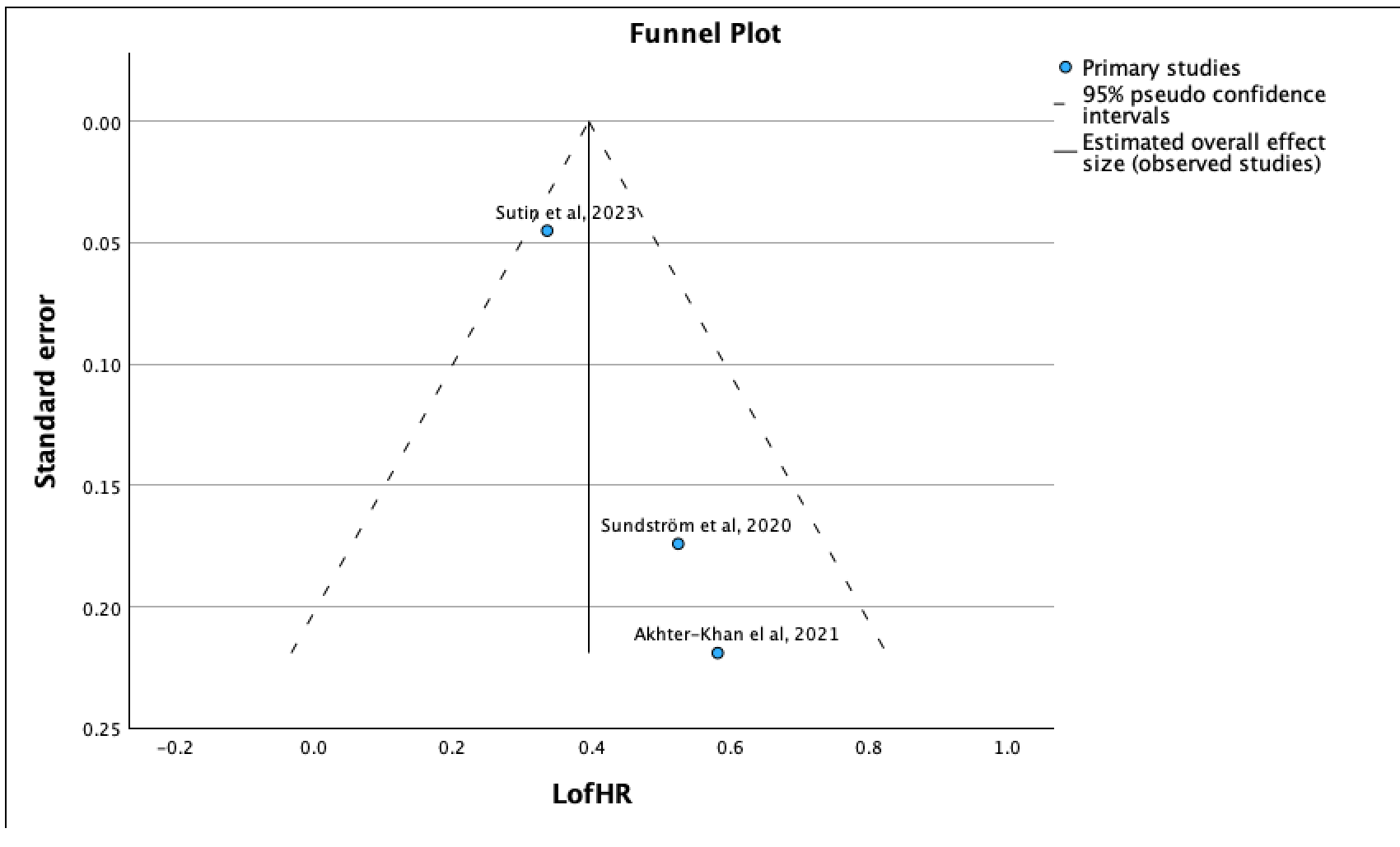


| **Egger's Regression-Based Test^a^** | | | | | | |
| --- | --- | --- | --- | --- | --- | --- |
| Parameter | Coefficient | Std. Error | t | Sig. (2-tailed) | 95% Confidence Interval | |
|  |  |  |  |  | Lower | Upper |
| (Intercept) | ·271 | ·0716 | 3·788 | ·164 | -·639 | 1·182 |
| SE^b^ | 1·437 | ·9680 | 1·485 | ·377 | -10·862 | 13·736 |
| a. Random-effects meta-regression | | | | | | |
| b. Standard error of effect size | | | | | | |

Egger’s Test did not demonstrate risk of bias [CI: -0·639 to 1·182]. No study demonstrated funnel plot asymmetry.

**Graphic 2a. Association between AD and loneliness.**


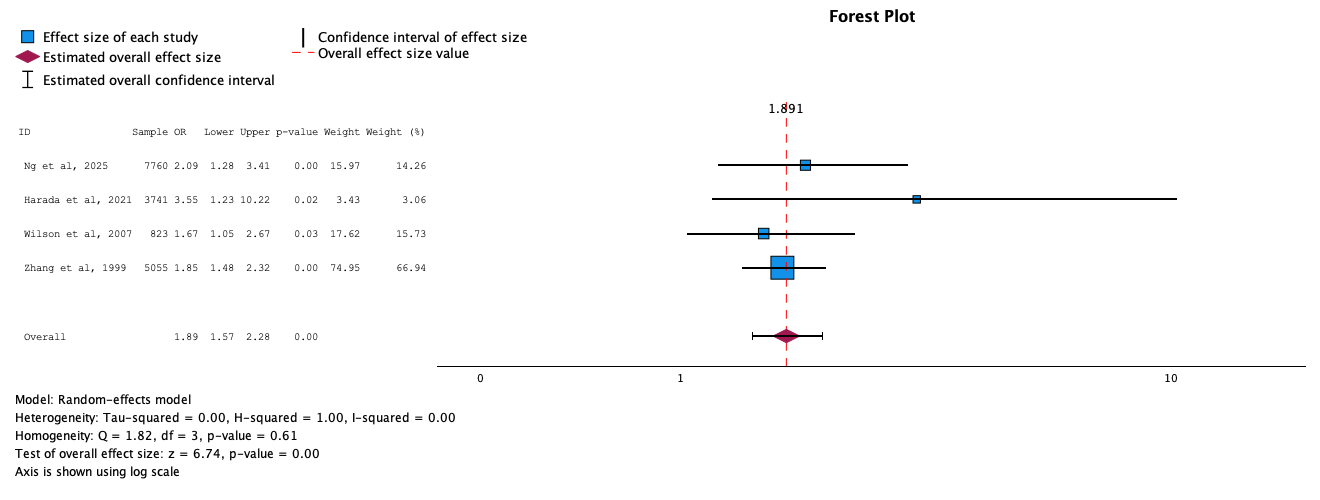

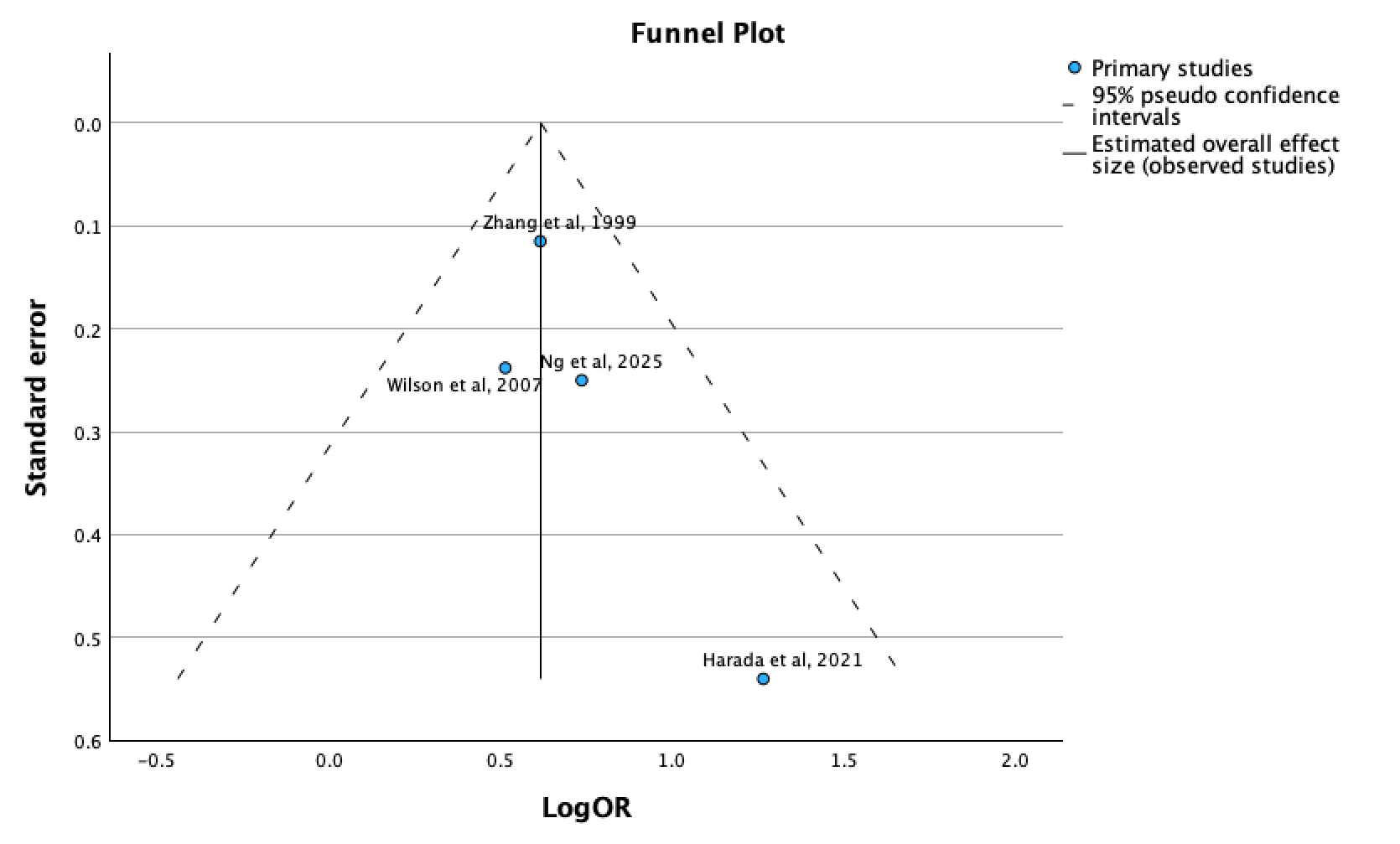


| **Egger's Regression-Based Test^a^** | | | | | | |
| --- | --- | --- | --- | --- | --- | --- |
| Parameter | Coefficient | Std. Error | t | Sig. (2-tailed) | 95% Confidence Interval | |
|  |  |  |  |  | Lower | Upper |
| (Intercept) | ·473 | ·2007 | 2·358 | ·142 | -·390 | 1·337 |
| SE^b^ | ·983 | 1·0655 | ·923 | ·454 | -3·601 | 5·568 |
| a. Random-effects meta-regression | | | | | | |
| b. Standard error of effect size | | | | | | |

**Graphic 3. Association between loneliness and suicidal ideation**


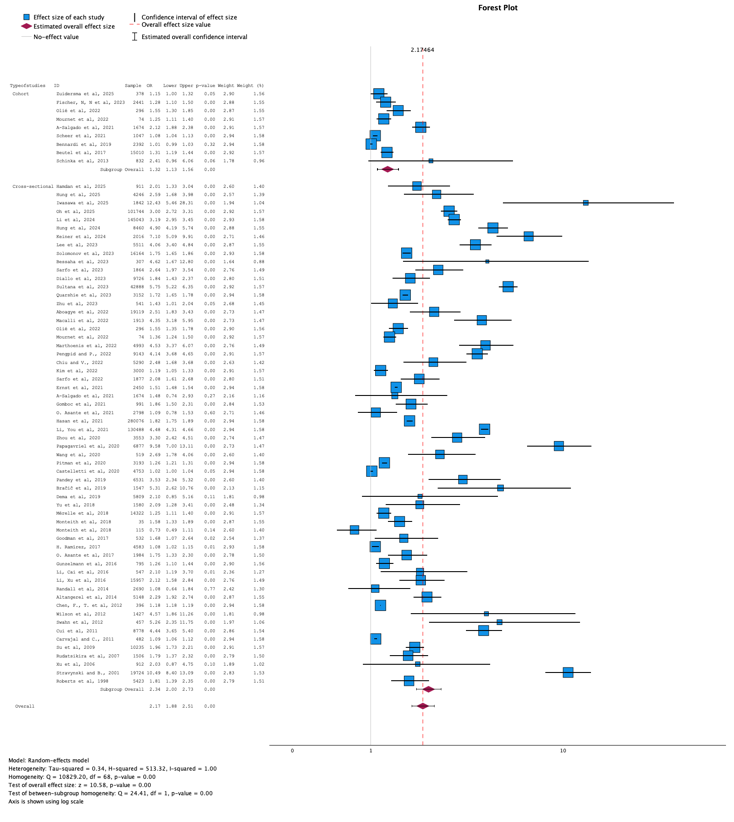

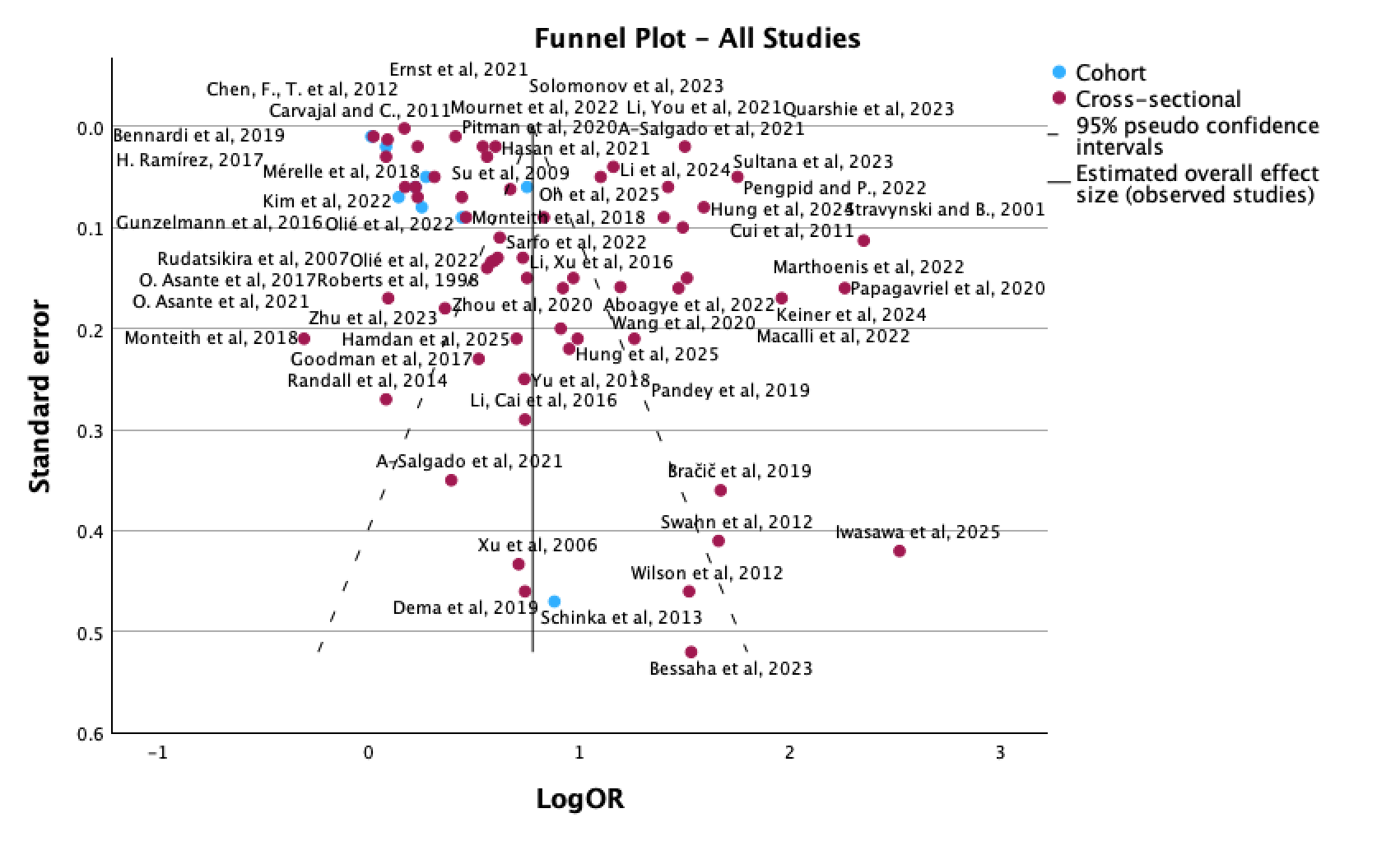


**Continuing graphic 3.**

| **Egger's Regression-Based Test^a^** | | | | | | | |
| --- | --- | --- | --- | --- | --- | --- | --- |
|  | Parameter | Coefficient | Std. Error | t | Sig. (2-tailed) | 95% Confidence Interval | |
|  |  |  |  |  |  | Lower | Upper |
| Cohort | (Intercept) | ·155 | ·1033 | 1·500 | ·177 | -·089 | ·399 |
|  | SE^b^ | 1·960 | 1·1430 | 1·714 | ·130 | -·743 | 4·662 |
| Cross-sectional | (Intercept) | ·639 | ·1215 | 5·258 | <·001 | ·396 | ·882 |
|  | SE^b^ | 1·492 | ·6654 | 2·243 | ·029 | ·160 | 2·825 |
| Overall | (Intercept) | ·546 | ·1060 | 5·149 | <·001 | ·334 | ·758 |
|  | SE^b^ | 1·731 | ·6018 | 2·876 | ·005 | ·530 | 2·932 |
| a. Random-effects meta-regression | | | | | | | |
| b. Standard error of effect size | | | | | | | |

| **Effect Size Estimates for Trim-and-Fill Analysis** | | | | | | | |
| --- | --- | --- | --- | --- | --- | --- | --- |
|  | Number | Effect Size | Std. Error | Z | Sig. (2-tailed) | 95% Confidence Interval | |
|  |  |  |  |  |  | Lower | Upper |
| Observed | 69 | ·777 | ·0734 | 10·583 | <·001 | ·633 | ·921 |
| Observed + Imputed^a^ | 69 | ·777 | ·0734 | 10·583 | <·001 | ·633 | ·921 |
| a. Number of imputed studies: 0 | | | | | | | |

Egger’s Test demonstrates risk of bias [CI: 0·334 to 0·758]. The Trim-and-Fill analysis did not find a difference in effect size between the observed and the observed plus imputed groups and identified a low risk of bias. More than half of the studies demonstrated asymmetry in the funnel plot. The data were recalculated in fixed-model and pooling data demonstrated a high heterogeneity with a OR of 1·22 [CI: 1·21-1·22; p<0·01; *I*^2^ = 100%; z = 107·99]. (Supplementary Graphic 3a).

**Graphic 3a. Association between loneliness and suicidal ideation in fixed-model**


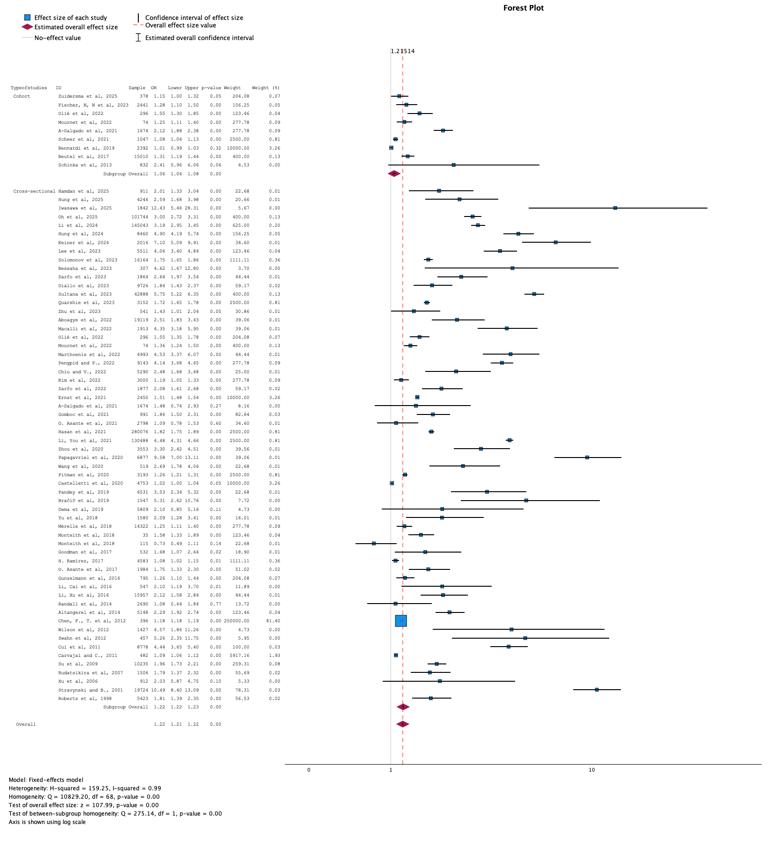

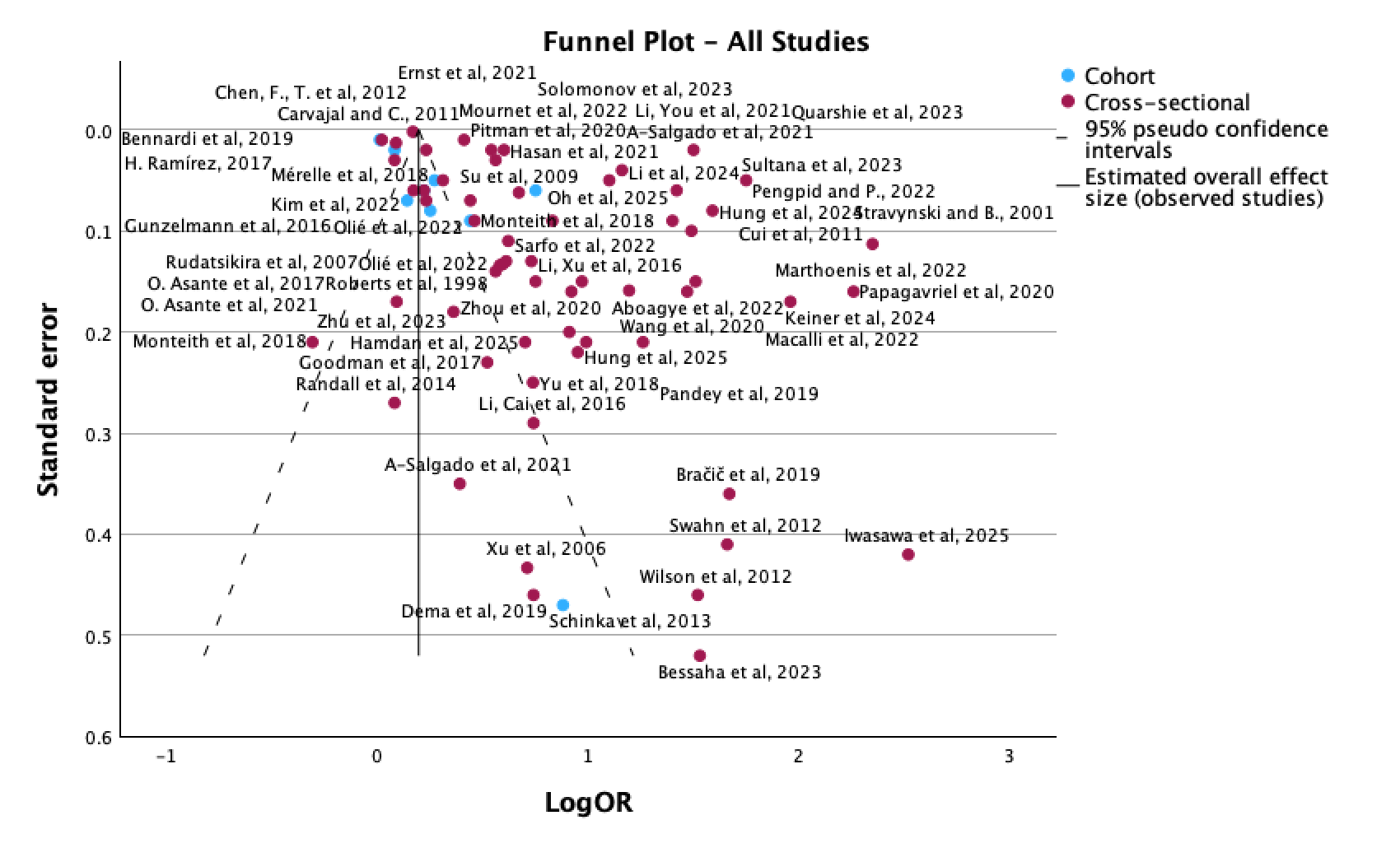


**Continuing graphic 3a.**

| **Egger's Regression-Based Test^a^** | | | | | | | |
| --- | --- | --- | --- | --- | --- | --- | --- |
|  | Parameter | Coefficient | Std. Error | t | Sig. (2-tailed) | 95% Confidence Interval | |
|  |  |  |  |  |  | Lower | Upper |
| Cohort | (Intercept) | -·032 | ·0117 | -2·755 | ·028 | -·060 | -·005 |
|  | SE^b^ | 5·141 | ·4587 | 11·207 | <·001 | 4·056 | 6·225 |
| Cross-sectional | (Intercept) | ·165 | ·0020 | 83·626 | <·001 | ·161 | ·169 |
|  | SE^b^ | 7·356 | ·1377 | 53·416 | <·001 | 7·080 | 7·632 |
| Overall | (Intercept) | ·159 | ·0019 | 81·696 | <·001 | ·155 | ·163 |
|  | SE^b^ | 6·523 | ·1296 | 50·335 | <·001 | 6·264 | 6·781 |
| a. Fixed-effects meta-regression | | | | | | | |
| b. Standard error of effect size | | | | | | | |

| **Effect Size Estimates for Trim-and-Fill Analysis** | | | | | | | |
| --- | --- | --- | --- | --- | --- | --- | --- |
|  | Number | Effect Size | Std. Error | Z | Sig. (2-tailed) | 95% Confidence Interval | |
|  |  |  |  |  |  | Lower | Upper |
| Observed | 69 | ·195 | ·0018 | 107·987 | <·001 | ·191 | ·198 |
| Observed + Imputed^a^ | 69 | ·195 | ·0018 | 107·987 | <·001 | ·191 | ·198 |
| a. Number of imputed studies: 0 | | | | | | | |

Egger’s Test demonstrates risk of bias [CI: 0·155 to 0·163]. The Trim-and-Fill analysis did not find a difference in effect size between the observed and the observed plus imputed groups and identified a low risk of bias. More than half of the studies demonstrated asymmetry in the funnel plot. Statistical analyses began to be carried out by groups of study types.

**Graphic 3b. Association between loneliness and suicidal ideation in longitudinal studies**


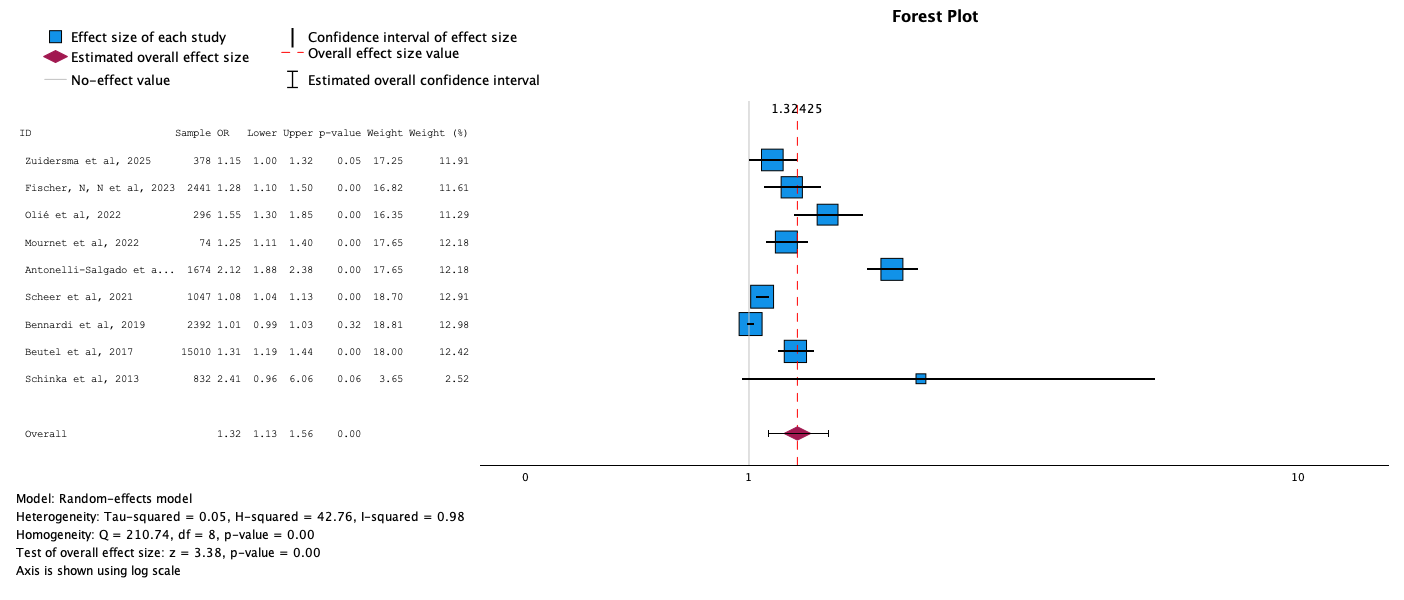

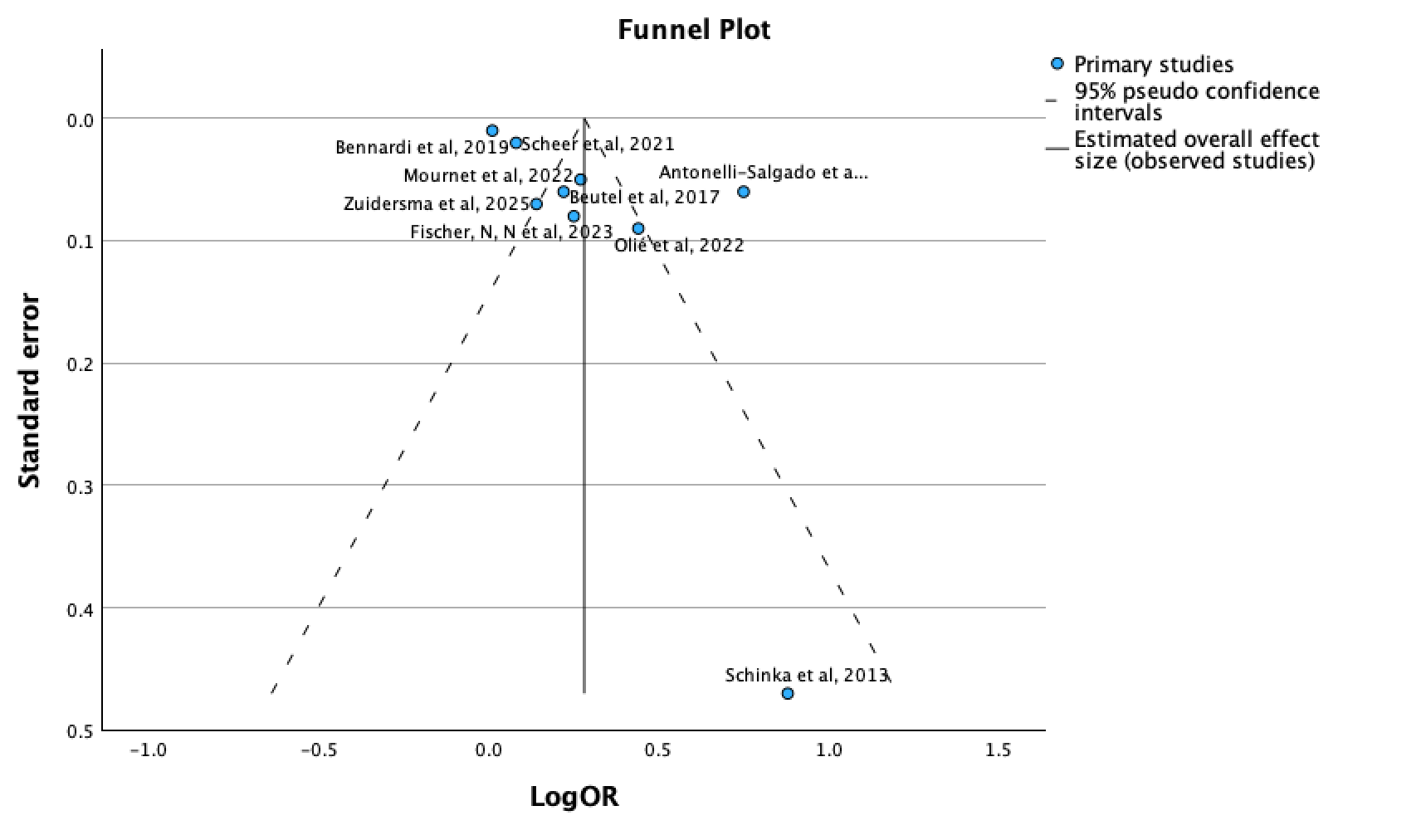


| **Egger's Regression-Based Test^a^** | | | | | | |
| --- | --- | --- | --- | --- | --- | --- |
| Parameter | Coefficient | Std. Error | t | Sig. (2-tailed) | 95% Confidence Interval | |
|  |  |  |  |  | Lower | Upper |
| (Intercept) | ·155 | ·1033 | 1·500 | ·177 | -·089 | ·399 |
| SE^b^ | 1·960 | 1·1430 | 1·714 | ·130 | -·743 | 4·662 |
| a. Random-effects meta-regression | | | | | | |
| b. Standard error of effect size | | | | | | |

Egger’s Test did not demonstrate risk of bias [CI: -0·089 to 0·399]. Three studies demonstrated asymmetry in the funnel plot. The data were recalculated in fixed-model and pooling data demonstrated a high heterogeneity with an OR of 1·06 [CI: 1·04-1·08; p<0·01; *I*^2^ = 96%; z = 6·80]. (Supplementary Graphic 3c).

**Graphic 3c. Association between loneliness and suicidal ideation in longitudinal studies in fixed-model**


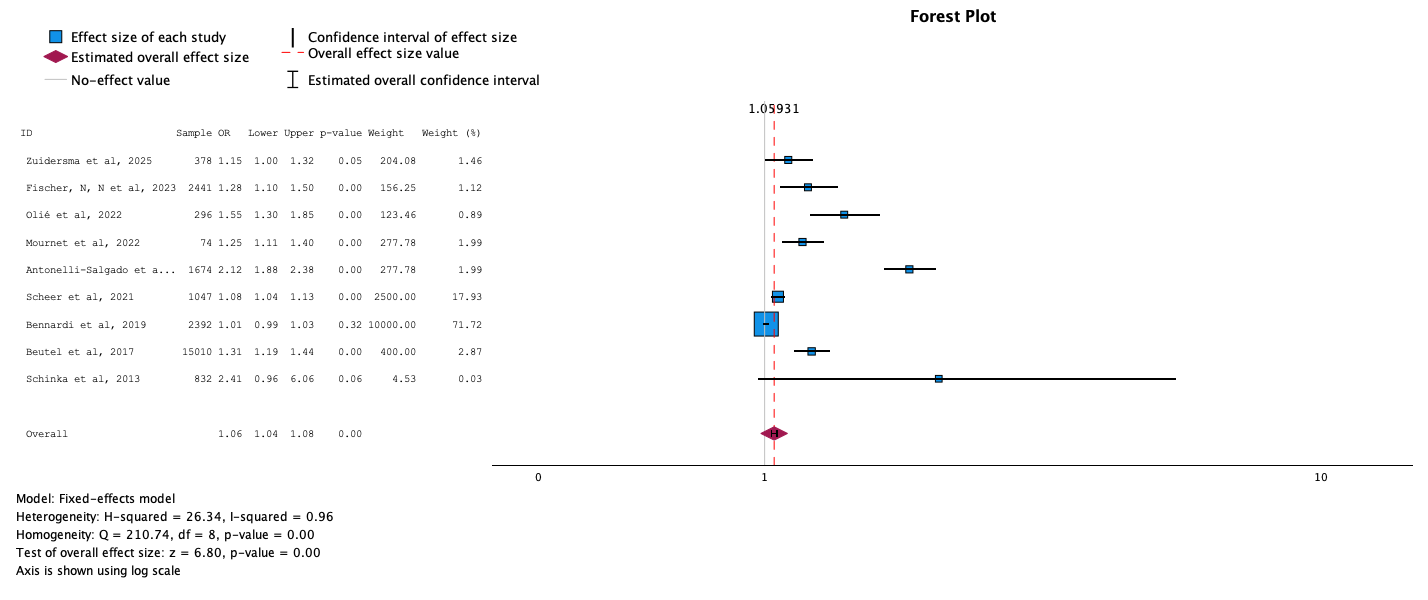

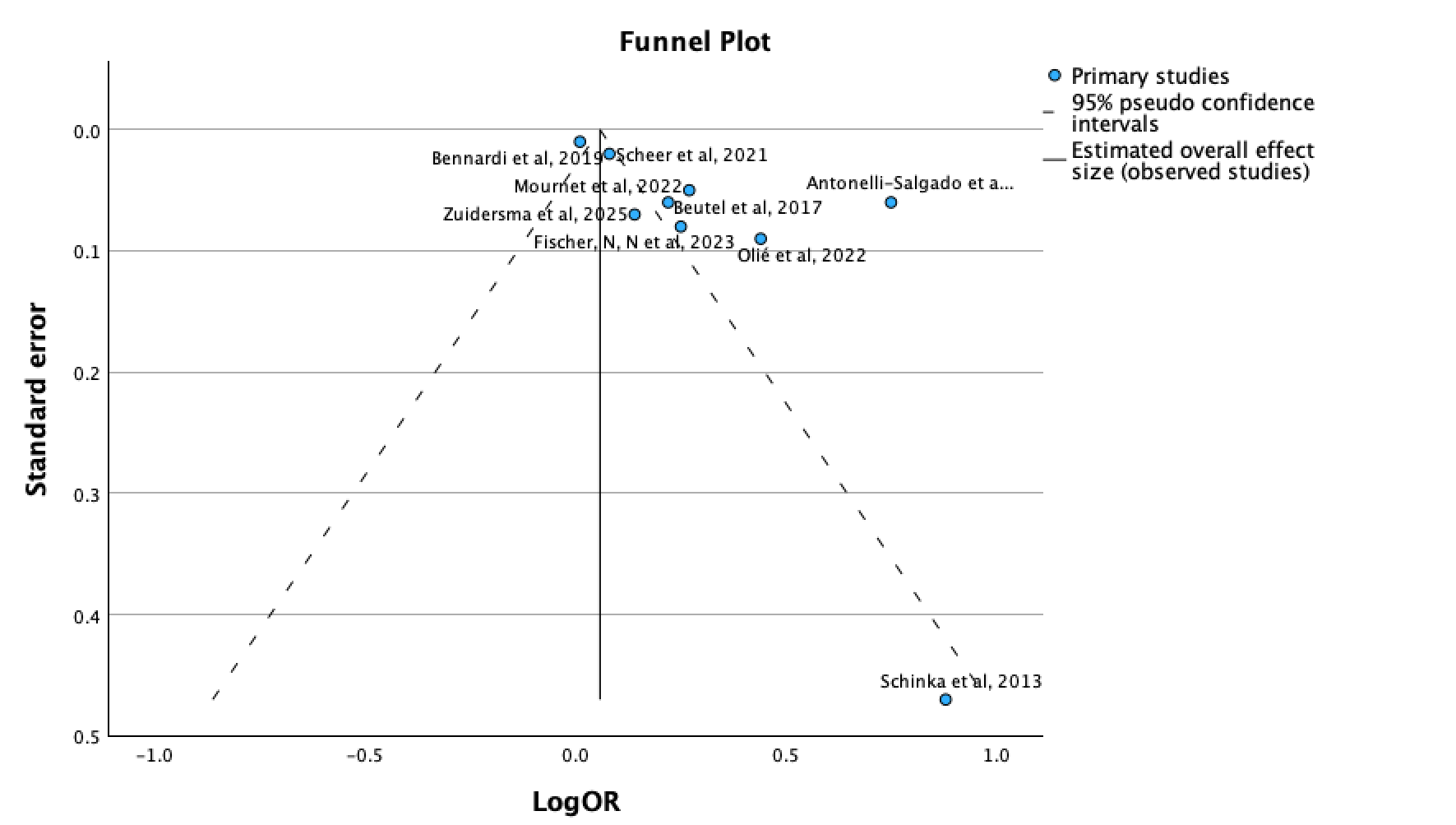

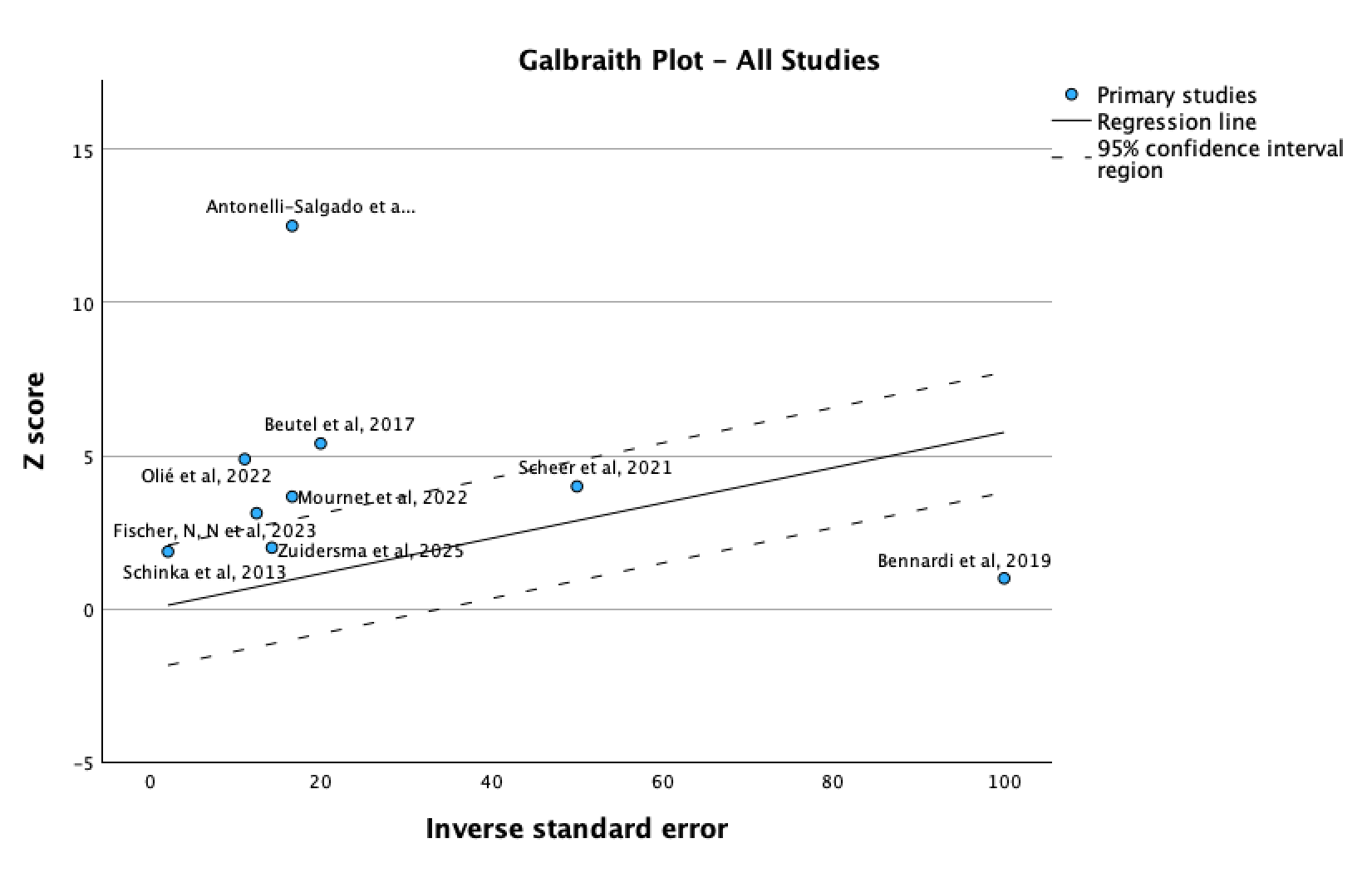


**Continuing graphic 3c.**

| **Egger's Regression-Based Test^a^** | | | | | | |
| --- | --- | --- | --- | --- | --- | --- |
| Parameter | Coefficient | Std. Error | t | Sig. (2-tailed) | 95% Confidence Interval | |
|  |  |  |  |  | Lower | Upper |
| (Intercept) | -·032 | ·0117 | -2·755 | ·028 | -·060 | -·005 |
| SE^b^ | 5·141 | ·4587 | 11·207 | <·001 | 4·056 | 6·225 |
| a. Fixed-effects meta-regression | | | | | | |
| b. Standard error of effect size | | | | | | |

| **Effect Size Estimates for Trim-and-Fill Analysis** | | | | | | | |
| --- | --- | --- | --- | --- | --- | --- | --- |
|  | Number | Effect Size | Std. Error | Z | Sig. (2-tailed) | 95% Confidence Interval | |
|  |  |  |  |  |  | Lower | Upper |
| Observed | 9 | ·058 | ·0085 | 6·803 | <·001 | ·041 | ·074 |
| Observed + Imputed^a^ | 9 | ·058 | ·0085 | 6·803 | <·001 | ·041 | ·074 |
| a. Number of imputed studies: 0 | | | | | | | |

Egger’s Test demonstrates risk of bias [CI: -0·060 to -0·005]. The Trim-and-Fill analysis did not find a difference in effect size between the observed and the observed plus imputed groups and identified a low risk of bias. More than half of the studies demonstrated asymmetry in the funnel plot. It was used the Galbraith Plot to help found asymmetry studies. Two studies demonstrated high asymmetry ^278,299^. After adjusted for asymmetry the pooling date demonstrated moderate risk of heterogeneity with an OR of 1·14 [CI: 1·04-1·26; p=0·01; *I*^2^ = 57%; z = 2·76]. (Supplementary Graphics 3d, e). Egger’s Test did not demonstrate risk of bias [CI: -0·387 to 0·463].

**Graphic 3d. Association between loneliness and suicidal ideation in longitudinal studies after asymmetry adjusted**


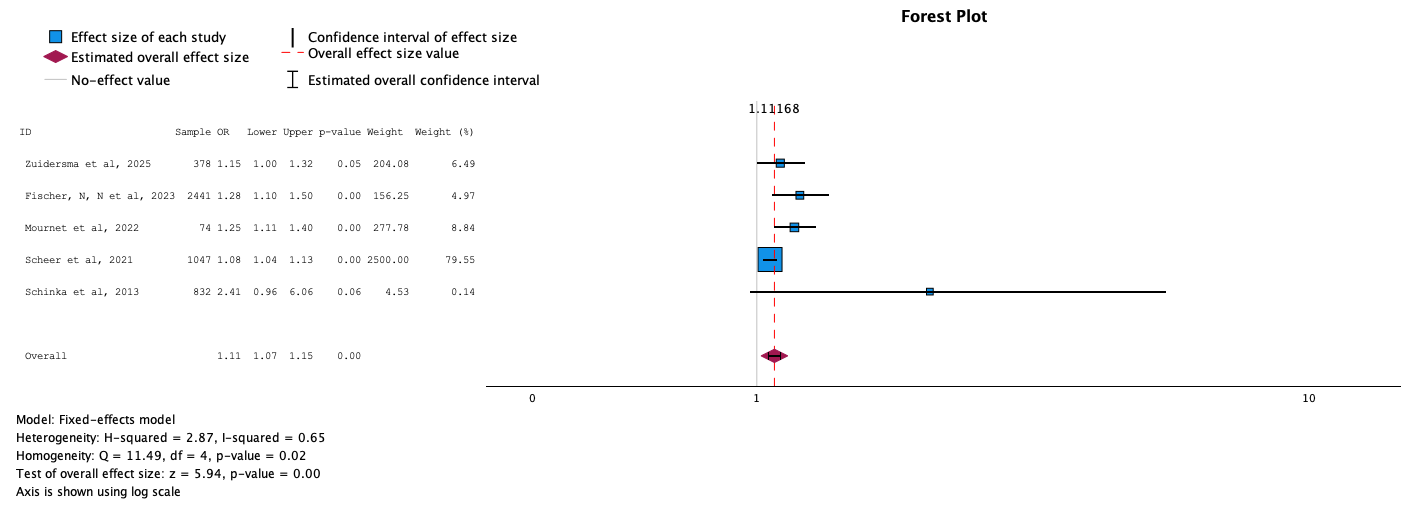

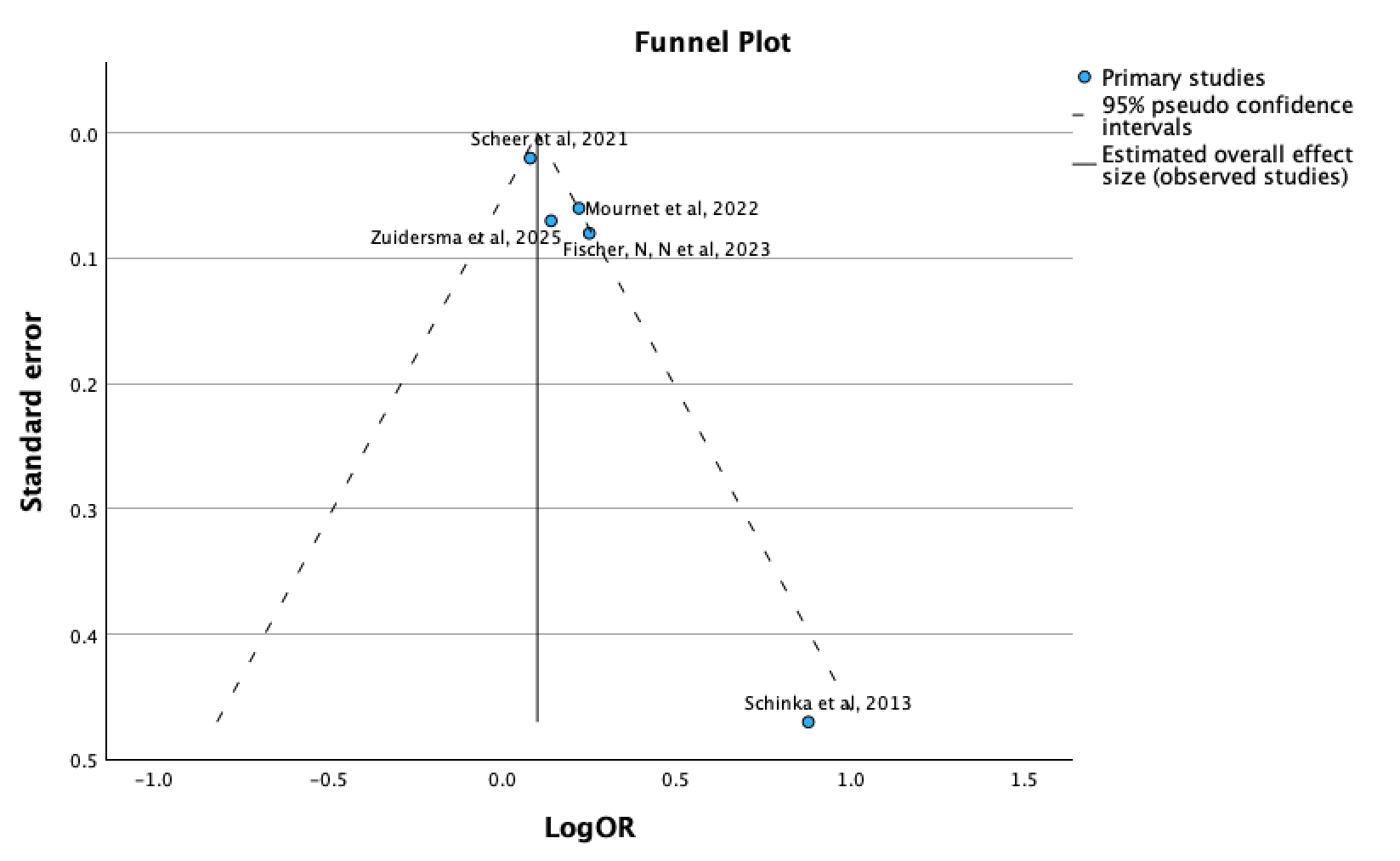


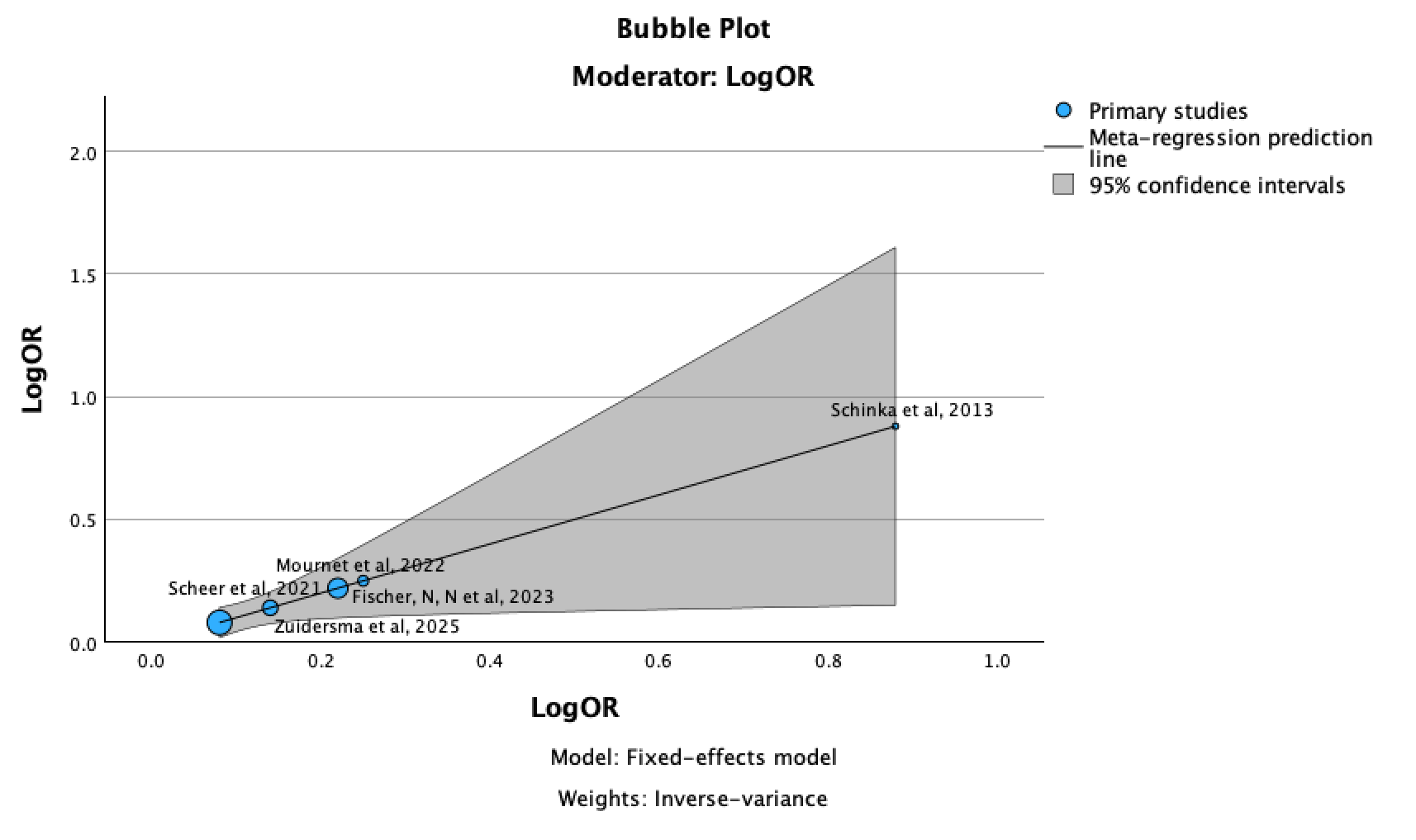


**Continuing graphic 3d.**

| **Egger's Regression-Based Test^a^** | | | | | | |
| --- | --- | --- | --- | --- | --- | --- |
| Parameter | Coefficient | Std. Error | t | Sig. (2-tailed) | 95% Confidence Interval | |
|  |  |  |  |  | Lower | Upper |
| (Intercept) | ·040 | ·0276 | 1·445 | ·244 | -·048 | ·128 |
| SE^b^ | 2·171 | ·6912 | 3·141 | ·052 | -·029 | 4·371 |
| a. Fixed-effects meta-regression | | | | | | |
| b. Standard error of effect size | | | | | | |

| **Effect Size Estimates for Trim-and-Fill Analysis** | | | | | | | |
| --- | --- | --- | --- | --- | --- | --- | --- |
|  | Number | Effect Size | Std. Error | Z | Sig. (2-tailed) | 95% Confidence Interval | |
|  |  |  |  |  |  | Lower | Upper |
| Observed | 5 | ·106 | ·0178 | 5·935 | <·001 | ·071 | ·141 |
| Observed + Imputed^a^ | 7 | ·101 | ·0174 | 5·781 | <·001 | ·066 | ·135 |
| a. Number of imputed studies: 2 | | | | | | | |

**Graphic 3e. Association between loneliness and suicidal ideation in longitudinal studies after asymmetry adjusted 2**


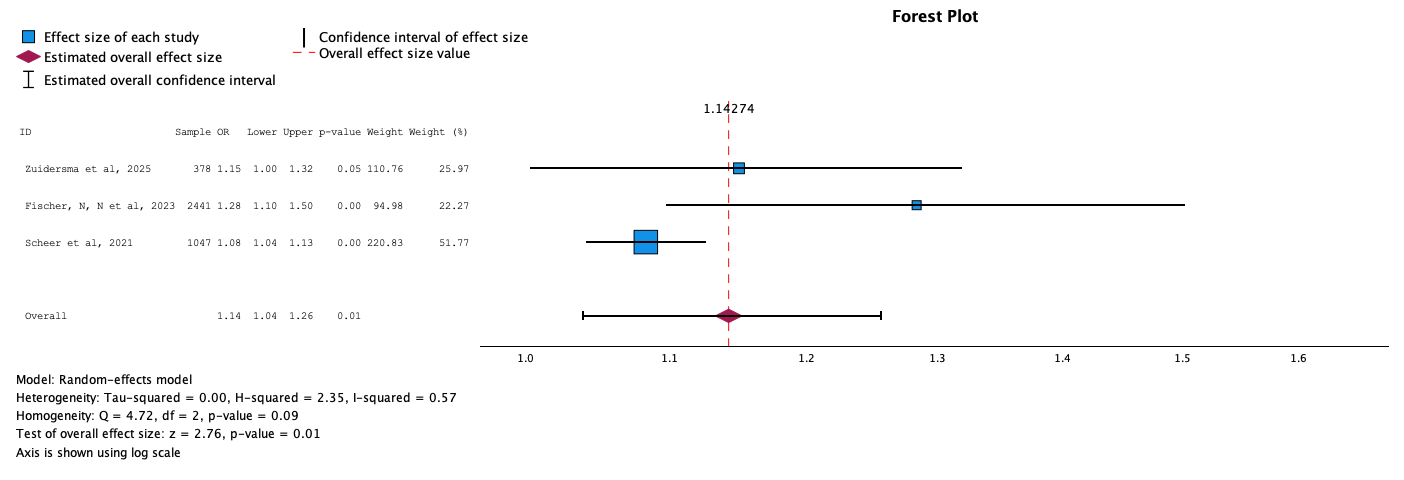

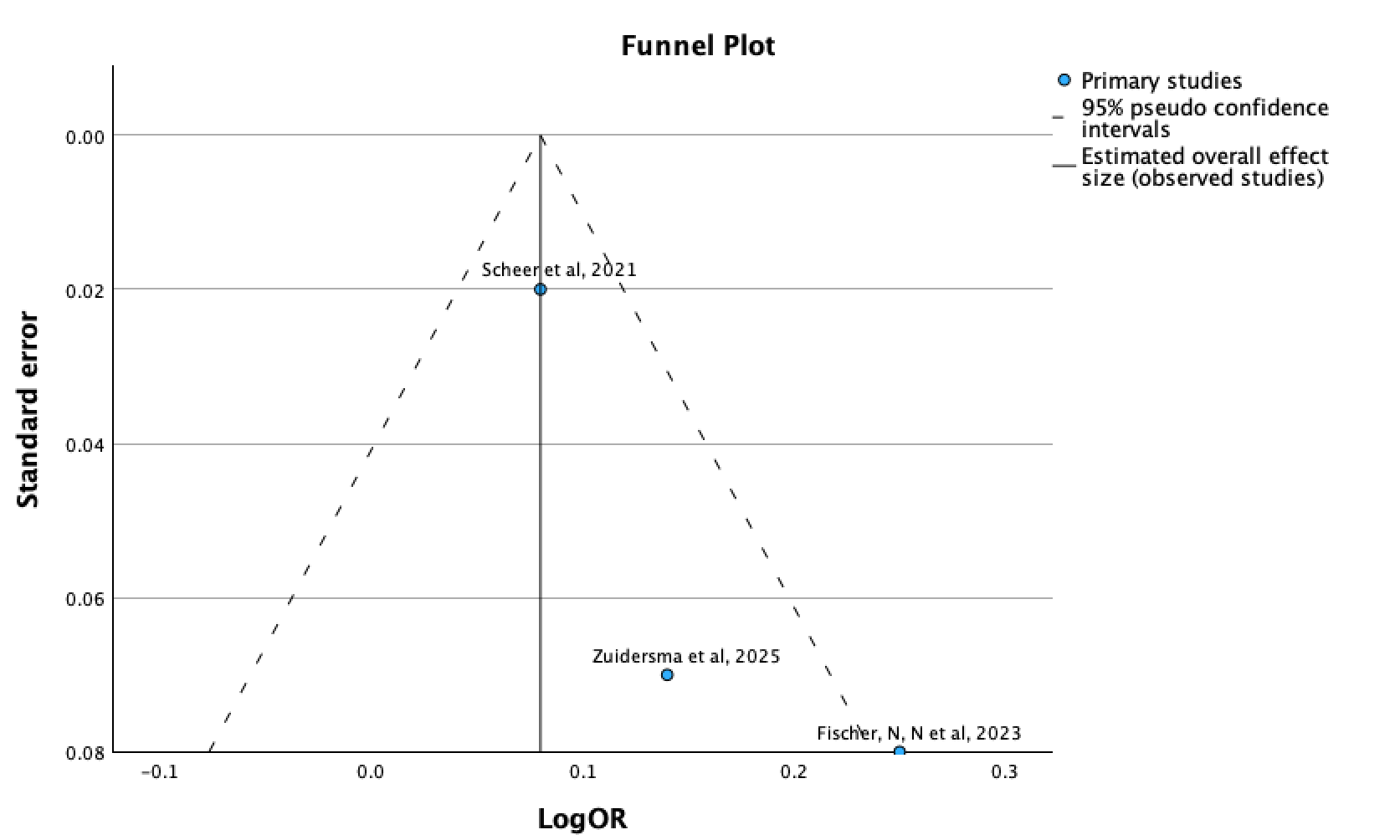


| **Egger's Regression-Based Test^a^** | | | | | | |
| --- | --- | --- | --- | --- | --- | --- |
| Parameter | Coefficient | Std. Error | t | Sig. (2-tailed) | 95% Confidence Interval | |
|  |  |  |  |  | Lower | Upper |
| (Intercept) | ·038 | ·0334 | 1·139 | ·459 | -·387 | ·463 |
| SE^b^ | 2·067 | 1·0321 | 2·003 | ·295 | -11·048 | 15·182 |
| a. Random-effects meta-regression | | | | | | |
| b. Standard error of effect size | | | | | | |

**Graphic 3f. Association between loneliness and suicidal ideation in cross-sectional studies**


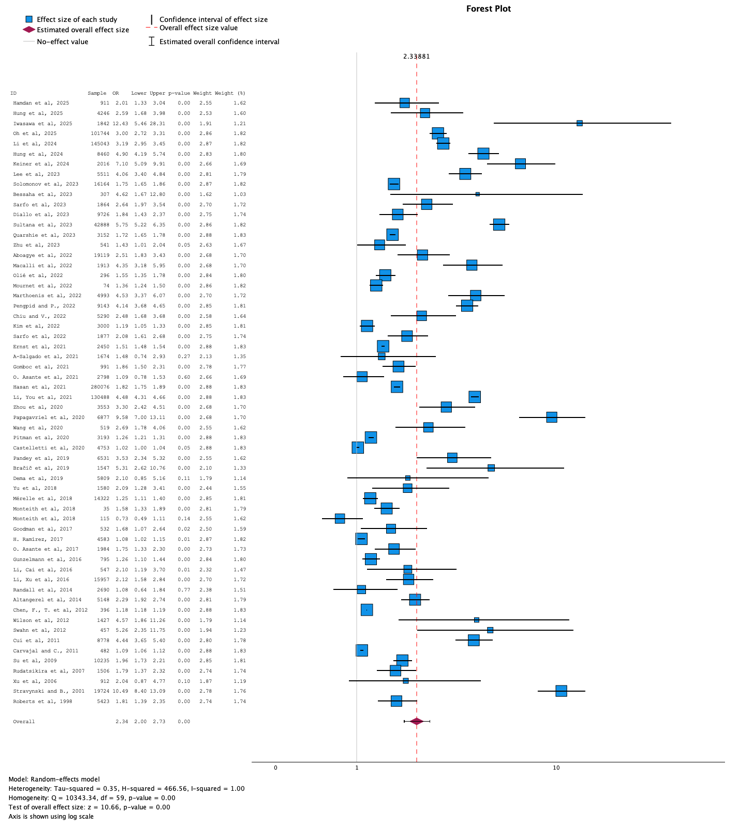

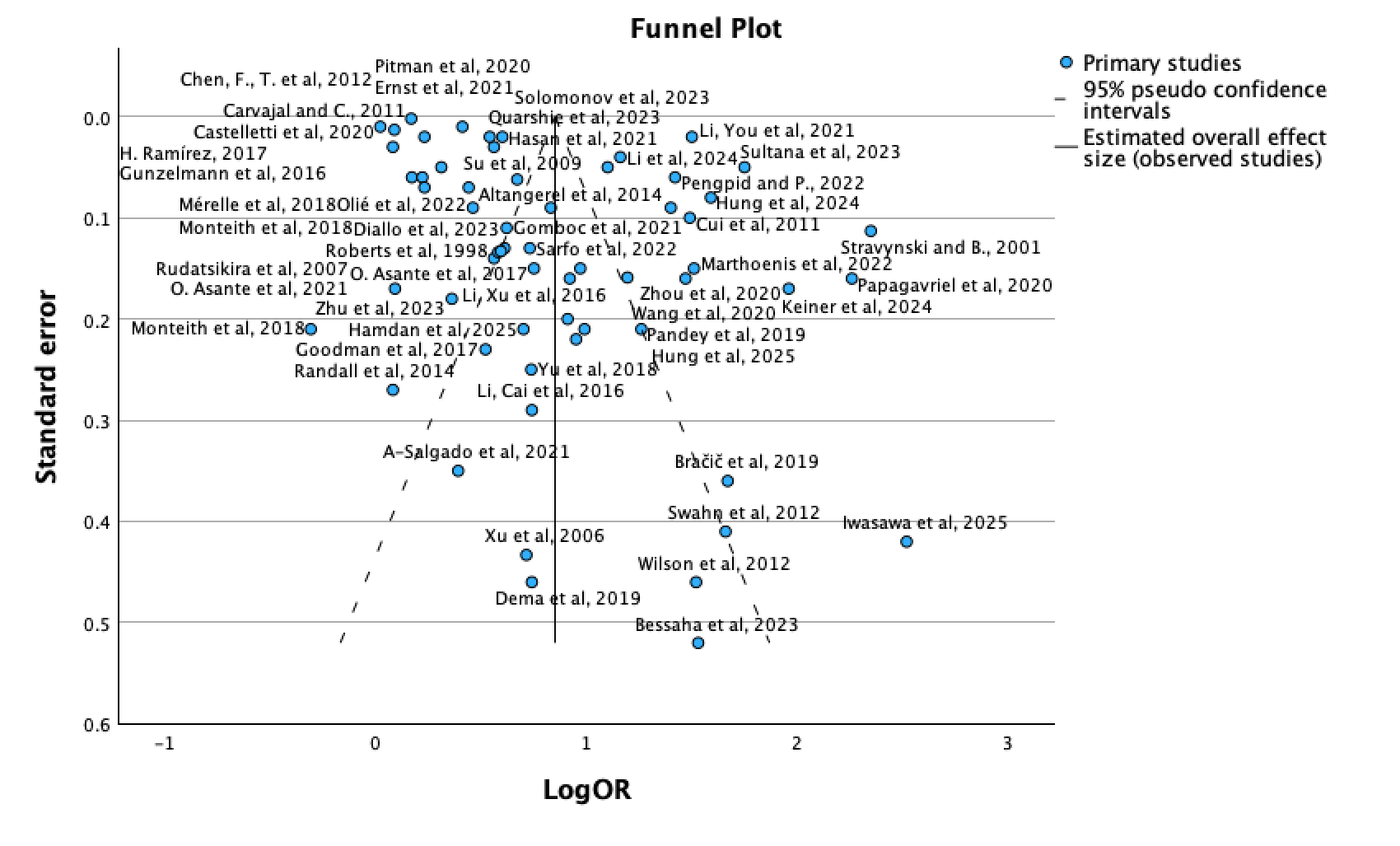


**Continuing graphic 3f.**

| **Egger's Regression-Based Test^a^** | | | | | | |
| --- | --- | --- | --- | --- | --- | --- |
| Parameter | Coefficient | Std. Error | t | Sig. (2-tailed) | 95% Confidence Interval | |
|  |  |  |  |  | Lower | Upper |
| (Intercept) | ·639 | ·1215 | 5·258 | <·001 | ·395 | ·882 |
| SE^b^ | 1·493 | ·6654 | 2·244 | ·029 | ·162 | 2·825 |
| a. Random-effects meta-regression | | | | | | |
| b. Standard error of effect size | | | | | | |

| **Effect Size Estimates for Trim-and-Fill Analysis** | | | | | | | |
| --- | --- | --- | --- | --- | --- | --- | --- |
|  | Number | Effect Size | Std. Error | Z | Sig. (2-tailed) | 95% Confidence Interval | |
|  |  |  |  |  |  | Lower | Upper |
| Observed | 60 | ·850 | ·0797 | 10·662 | <·001 | ·693 | 1.006 |
| Observed + Imputed^a^ | 60 | ·850 | ·0797 | 10·662 | <·001 | ·693 | 1·006 |
| a. Number of imputed studies: 0 | | | | | | | |

Egger’s Test demonstrates risk of bias [CI: 0·395 to 0·882]. The Trim-and-Fill analysis did not find a difference in effect size between the observed and the observed plus imputed groups and identified a low risk of bias. More than half of the studies demonstrated asymmetry in the funnel plot. The data were recalculated in fixed-model and pooling data demonstrated a high heterogeneity with an OR of 1·22 [CI: 1·22-1·23; p<0·01; *I*^2^ = 100%; z = 109·04]. (Supplementary Graphic 3g).

**Graphic 3g. Association between loneliness and suicidal ideation in cross-sectional studies in fixed-model**
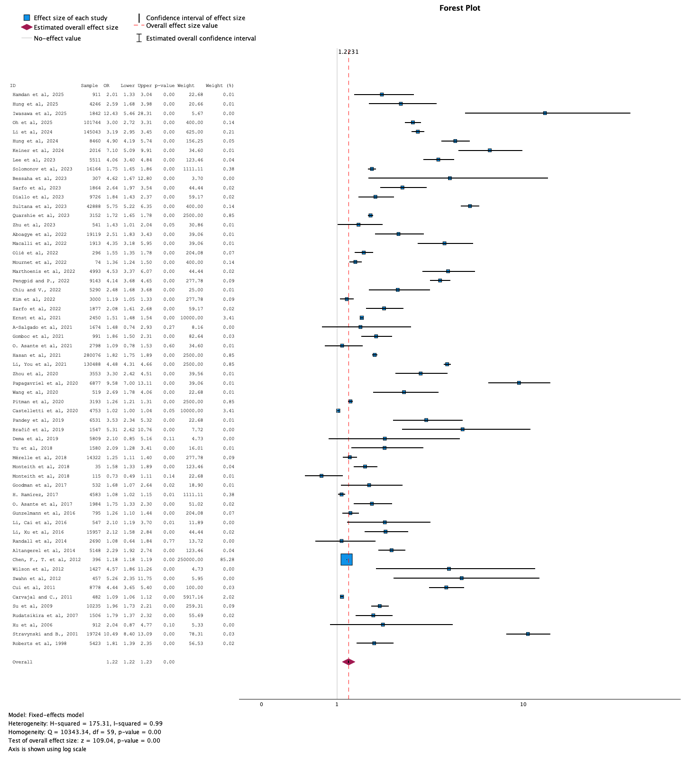

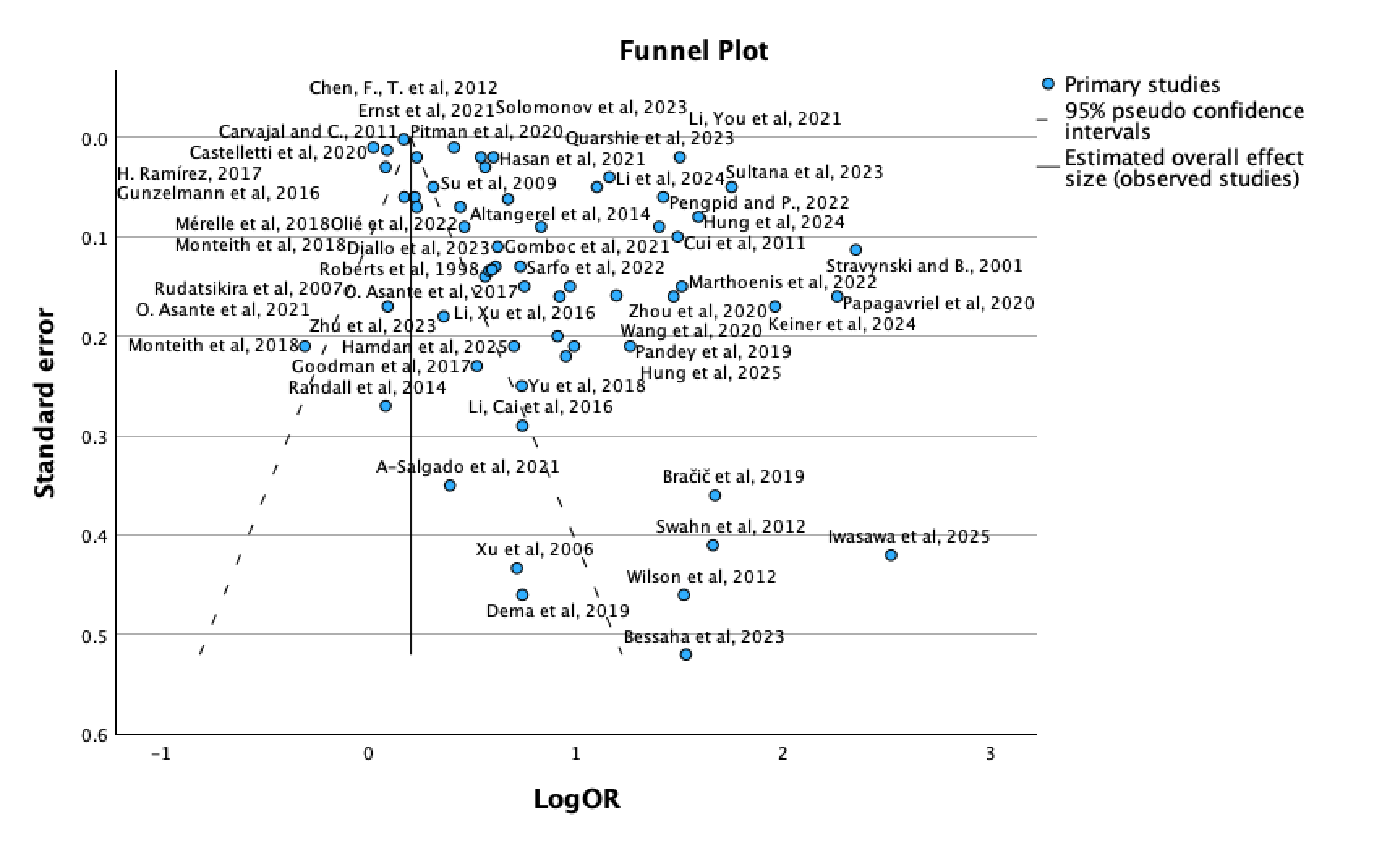


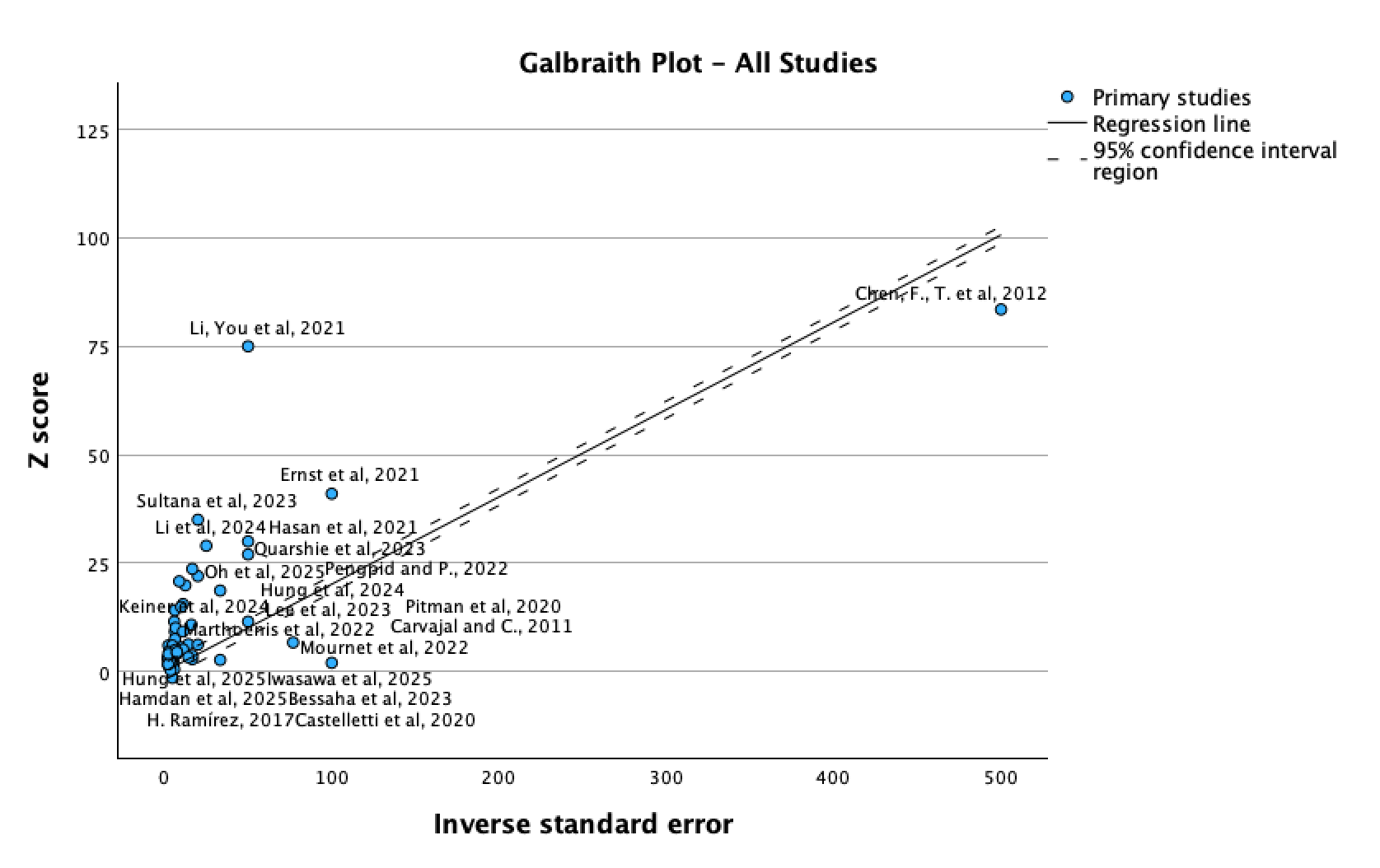

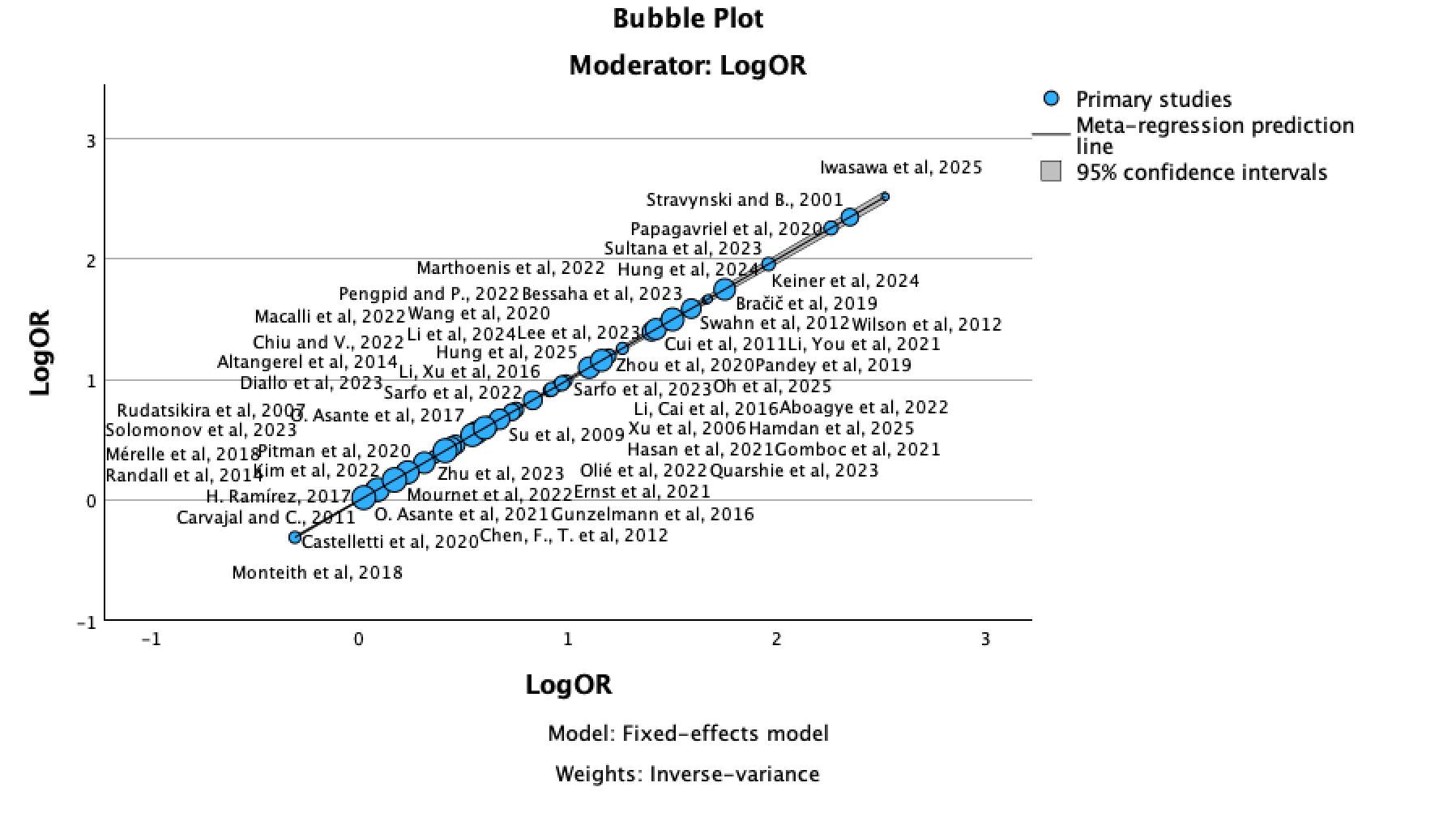


**Continuing graphic 3g.**

| **Egger's Regression-Based Test^a^** | | | | | | |
| --- | --- | --- | --- | --- | --- | --- |
| Parameter | Coefficient | Std. Error | t | Sig. (2-tailed) | 95% Confidence Interval | |
|  |  |  |  |  | Lower | Upper |
| (Intercept) | ·165 | ·0020 | 83·625 | <·001 | ·161 | ·169 |
| SE^b^ | 7·356 | ·1377 | 53·417 | <·001 | 7·081 | 7·632 |
| a. Fixed-effects meta-regression | | | | | | |
| b. Standard error of effect size | | | | | | |

| **Effect Size Estimates for Trim-and-Fill Analysis** | | | | | | | |
| --- | --- | --- | --- | --- | --- | --- | --- |
|  | Number | Effect Size | Std. Error | Z | Sig. (2-tailed) | 95% Confidence Interval | |
|  |  |  |  |  |  | Lower | Upper |
| Observed | 60 | ·201 | ·0018 | 109·041 | <·001 | ·198 | ·205 |
| Observed + Imputed^a^ | 60 | ·201 | ·0018 | 109·041 | <·001 | ·198 | ·205 |
| a. Number of imputed studies: 0 | | | | | | | |

Egger’s Test demonstrates risk of bias [CI: 0·161 to 0·169]. The Trim-and-Fill analysis did not find a difference in effect size between the observed and the observed plus imputed groups and identified a low risk of bias. More than half of the studies demonstrated asymmetry in the funnel plot. It was used the Galbraith Plot to help found asymmetry studies. Two studies demonstrated high asymmetry ^138,169^. After adjusted for asymmetry the pooling date demonstrated low risk of heterogeneity with an OR of 1·75 [CI: 1·69-1·82; p<0·01; *I*^2^ = 25%; z = 30·59]. (Supplementary Graphics 3h, i). Egger’s Test demonstrates risk of bias [CI: 0·504 to 0·627]. The Trim-and-Fill analysis did not find a difference in effect size between the observed and the observed plus imputed groups and identified a low risk of bias.

**Graphic 3h. Association between loneliness and suicidal ideation in cross-sectional studies after asymmetry adjusted**


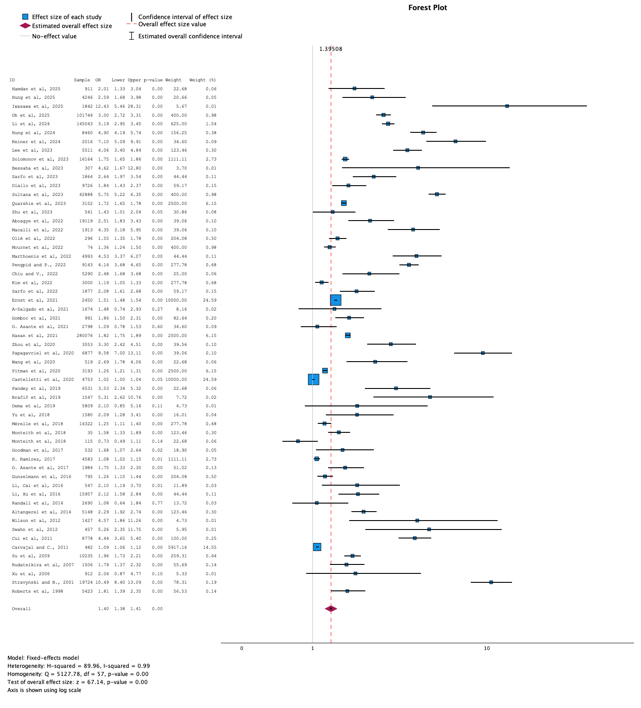

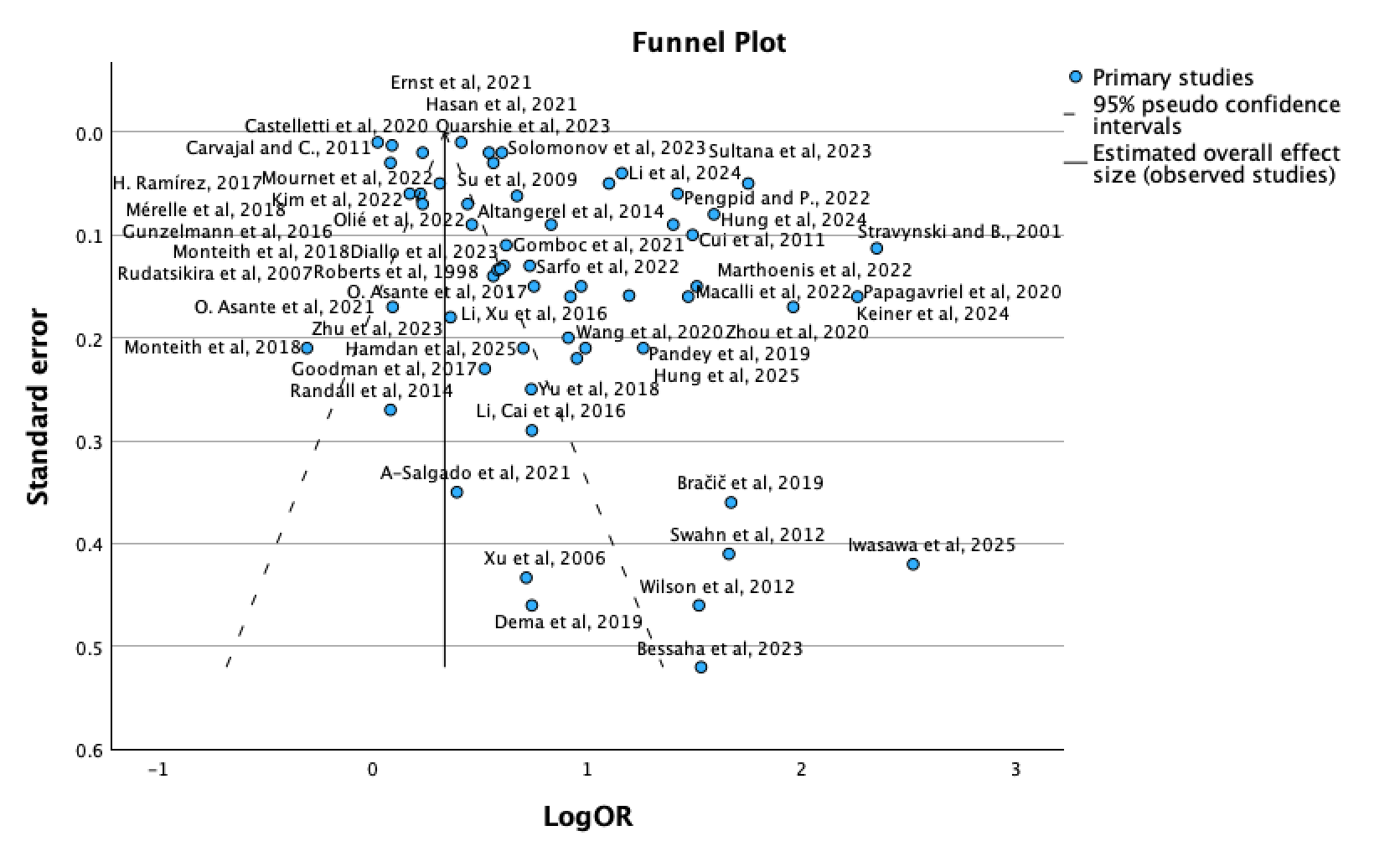


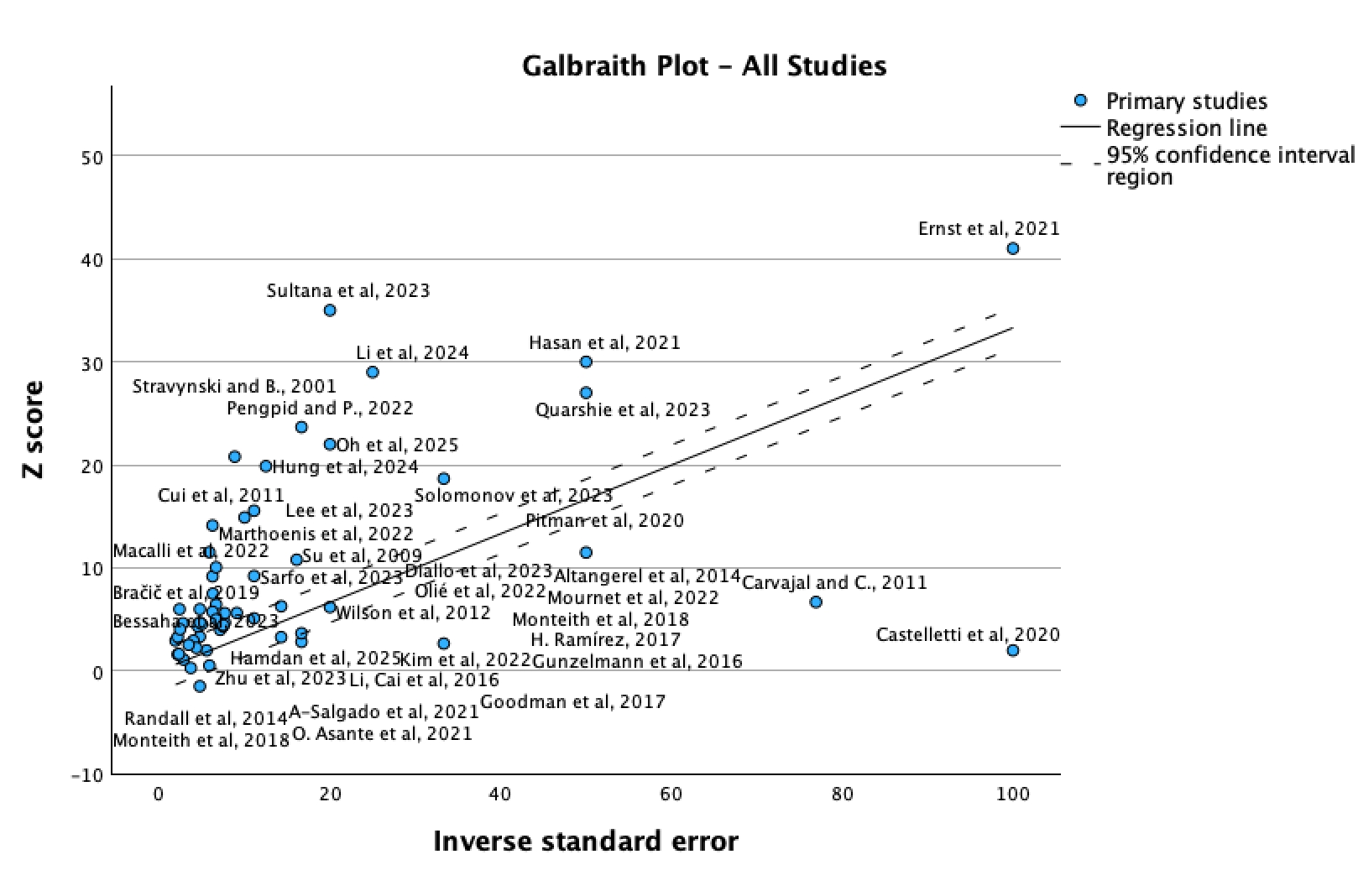


**Continuing graphic 3h.**

| **Egger's Regression-Based Test^a^** | | | | | | |
| --- | --- | --- | --- | --- | --- | --- |
| Parameter | Coefficient | Std. Error | t | Sig. (2-tailed) | 95% Confidence Interval | |
|  |  |  |  |  | Lower | Upper |
| (Intercept) | ·202 | ·0062 | 32·797 | <·001 | ·190 | ·214 |
| SE^b^ | 5·857 | ·1630 | 35·929 | <·001 | 5·530 | 6·183 |
| a. Fixed-effects meta-regression | | | | | | |
| b. Standard error of effect size | | | | | | |

| **Effect Size Estimates for Trim-and-Fill Analysis** | | | | | | | |
| --- | --- | --- | --- | --- | --- | --- | --- |
|  | Number | Effect Size | Std. Error | Z | Sig. (2-tailed) | 95% Confidence Interval | |
|  |  |  |  |  |  | Lower | Upper |
| Observed | 58 | ·333 | ·0050 | 67·144 | <·001 | ·323 | ·343 |
| Observed + Imputed^a^ | 58 | ·333 | ·0050 | 67·144 | <·001 | ·323 | ·343 |
| a. Number of imputed studies: 0 | | | | | | | |

**Graphic 3i. Association between loneliness and suicidal ideation in cross-sectional studies after asymmetry adjusted 2**


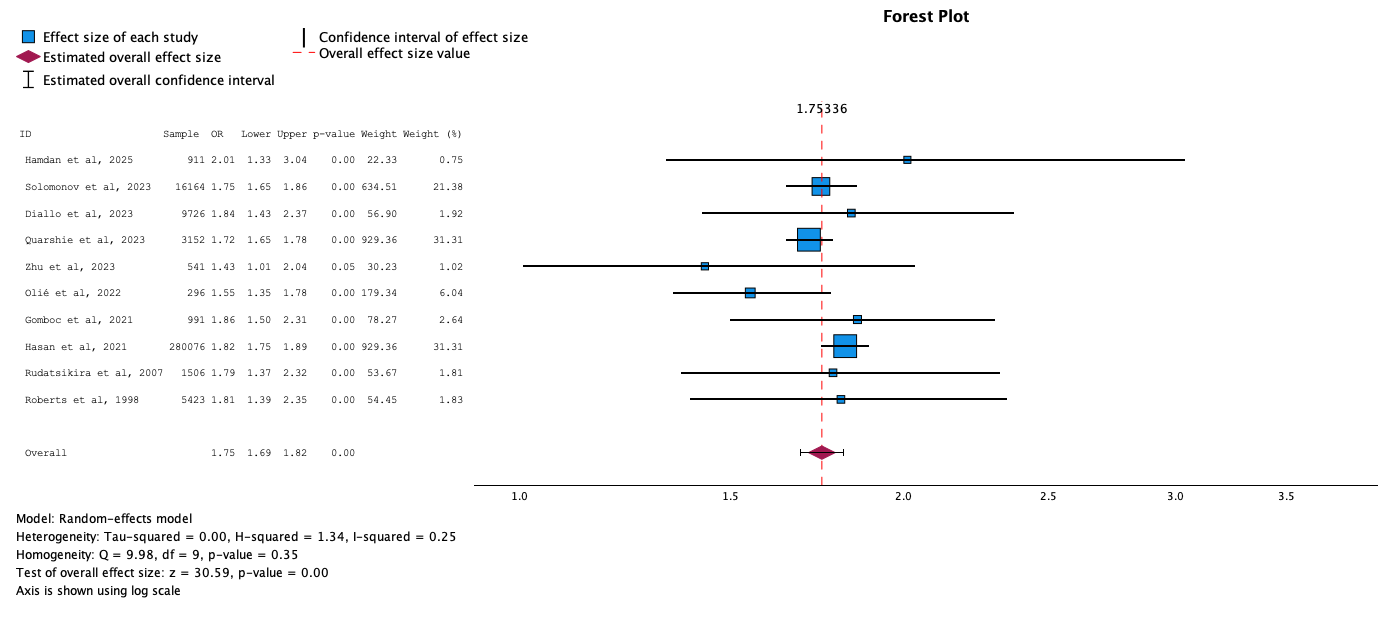

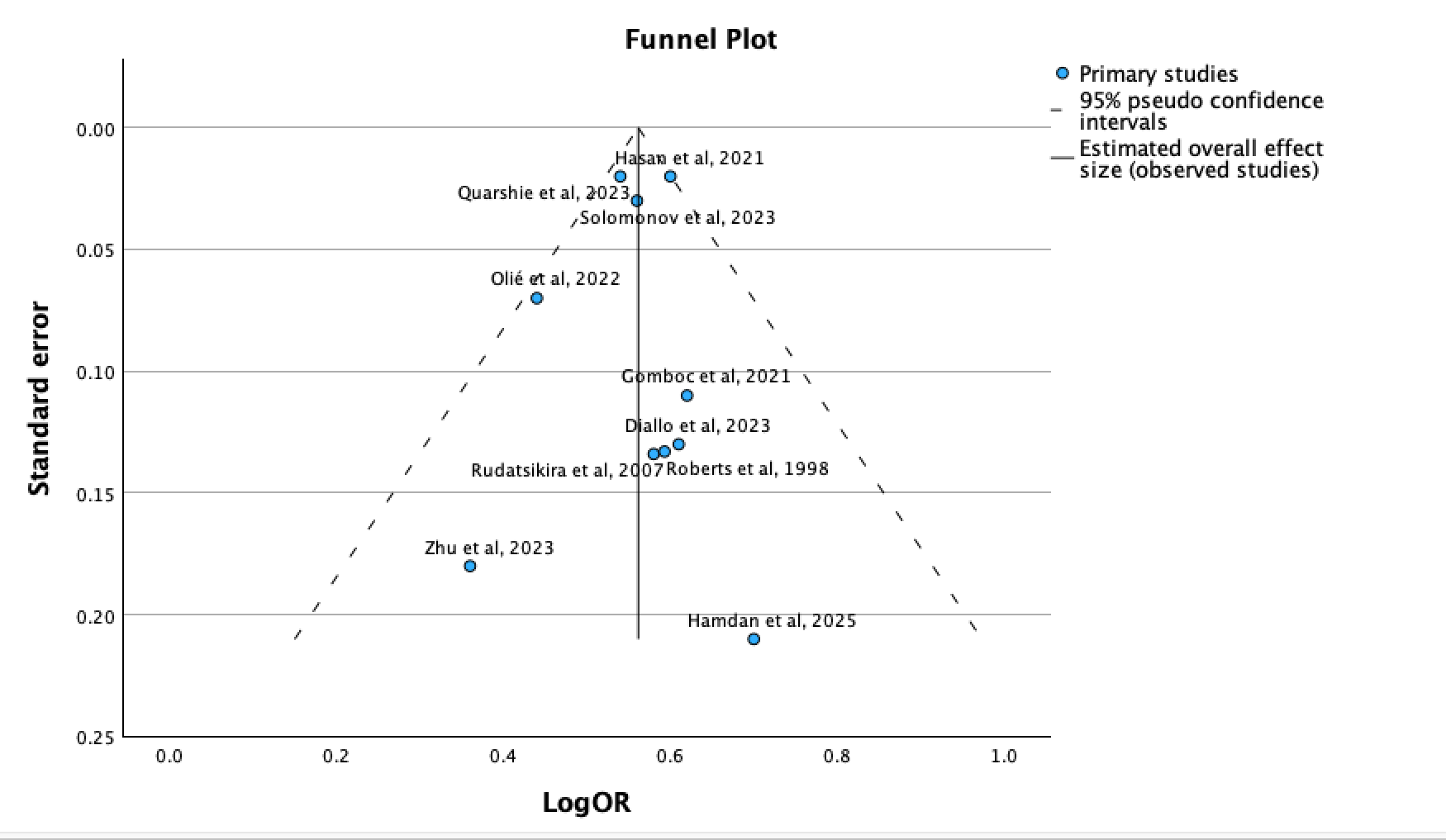


**Continuing graphic 3i.**

| **Egger's Regression-Based Test^a^** | | | | | | |
| --- | --- | --- | --- | --- | --- | --- |
| Parameter | Coefficient | Std. Error | t | Sig. (2-tailed) | 95% Confidence Interval | |
|  |  |  |  |  | Lower | Upper |
| (Intercept) | ·566 | ·0267 | 21·193 | <·001 | ·504 | ·627 |
| SE^b^ | -·113 | ·5051 | -·224 | ·828 | -1·278 | 1·052 |
| a. Random-effects meta-regression | | | | | | |
| b. Standard error of effect size | | | | | | |

| **Effect Size Estimates for Trim-and-Fill Analysis** | | | | | | | |
| --- | --- | --- | --- | --- | --- | --- | --- |
|  | Number | Effect Size | Std. Error | Z | Sig. (2-tailed) | 95% Confidence Interval | |
|  |  |  |  |  |  | Lower | Upper |
| Observed | 10 | ·562 | ·0184 | 30·594 | <·001 | ·526 | ·598 |
| Observed + Imputed^a^ | 10 | ·562 | ·0184 | 30·594 | <·001 | ·526 | ·598 |
| a. Number of imputed studies: 0 | | | | | | | |

**Graphic 4. Association between loneliness and suicidal planning.**


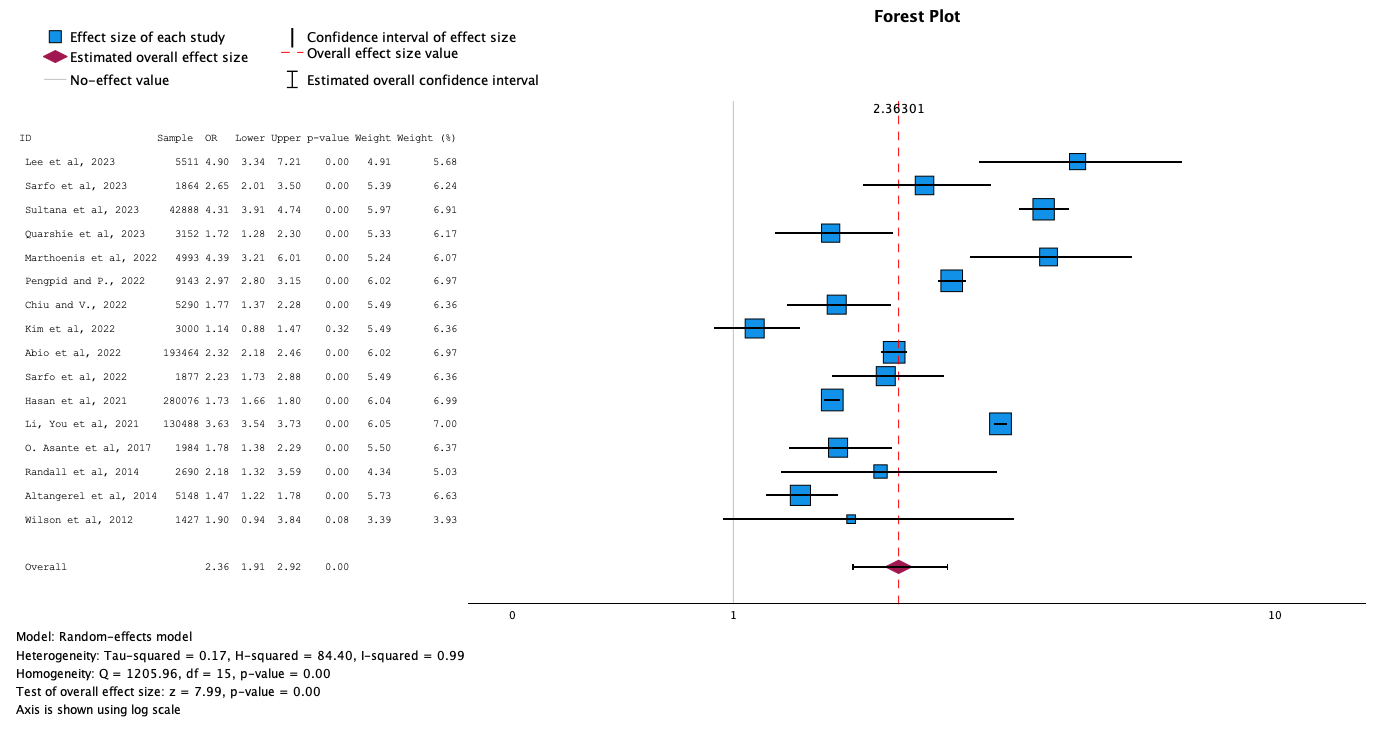

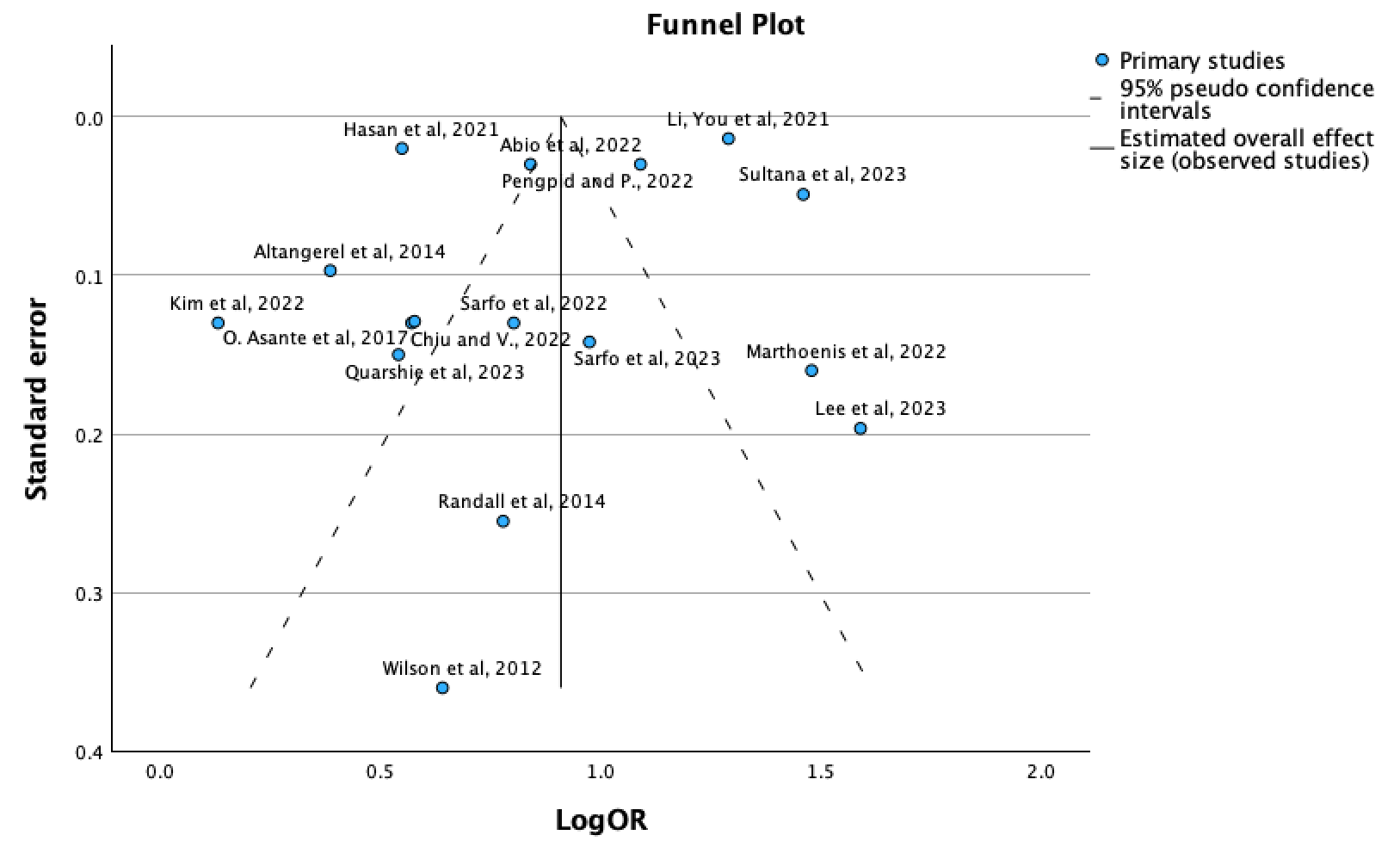


**Continuing graphic 4.**

| **Egger's Regression-Based Test^a^** | | | | | | |
| --- | --- | --- | --- | --- | --- | --- |
| Parameter | Coefficient | Std. Error | t | Sig. (2-tailed) | 95% Confidence Interval | |
|  |  |  |  |  | Lower | Upper |
| (Intercept) | ·925 | ·1903 | 4·860 | <·001 | ·517 | 1·333 |
| SE^b^ | -·563 | 1·3417 | -·420 | ·681 | -3·441 | 2·314 |
| a. Random-effects meta-regression | | | | | | |
| b. Standard error of effect size | | | | | | |

| **Effect Size Estimates for Trim-and-Fill Analysis** | | | | | | | |
| --- | --- | --- | --- | --- | --- | --- | --- |
|  | Number | Effect Size | Std. Error | Z | Sig. (2-tailed) | 95% Confidence Interval | |
|  |  |  |  |  |  | Lower | Upper |
| Observed | 16 | ·860 | ·1076 | 7·993 | <·001 | ·649 | 1·071 |
| Observed + Imputed^a^ | 17 | ·909 | ·1123 | 8·094 | <·001 | ·689 | 1·129 |
| a. Number of imputed studies: 1 | | | | | | | |

Egger’s Test demonstrates risk of bias [CI: 0·517 to 1·333]. The Trim-and-Fill analysis found a difference in effect size between the observed and the observed plus imputed groups, but included only one hypothetical study and identified a low risk of bias. More than half of the studies demonstrated asymmetry in the funnel plot. The data were recalculated in fixed-model and pooling data demonstrated a high heterogeneity with a OR of 2·80 [CI: 2·75-2·86; p<0·01; *I*^2^ = 100%; z = 34·00]. (Supplementary Graphic 4a).

**Graphic 4a. Association between loneliness and suicidal planning in fixed-model.**


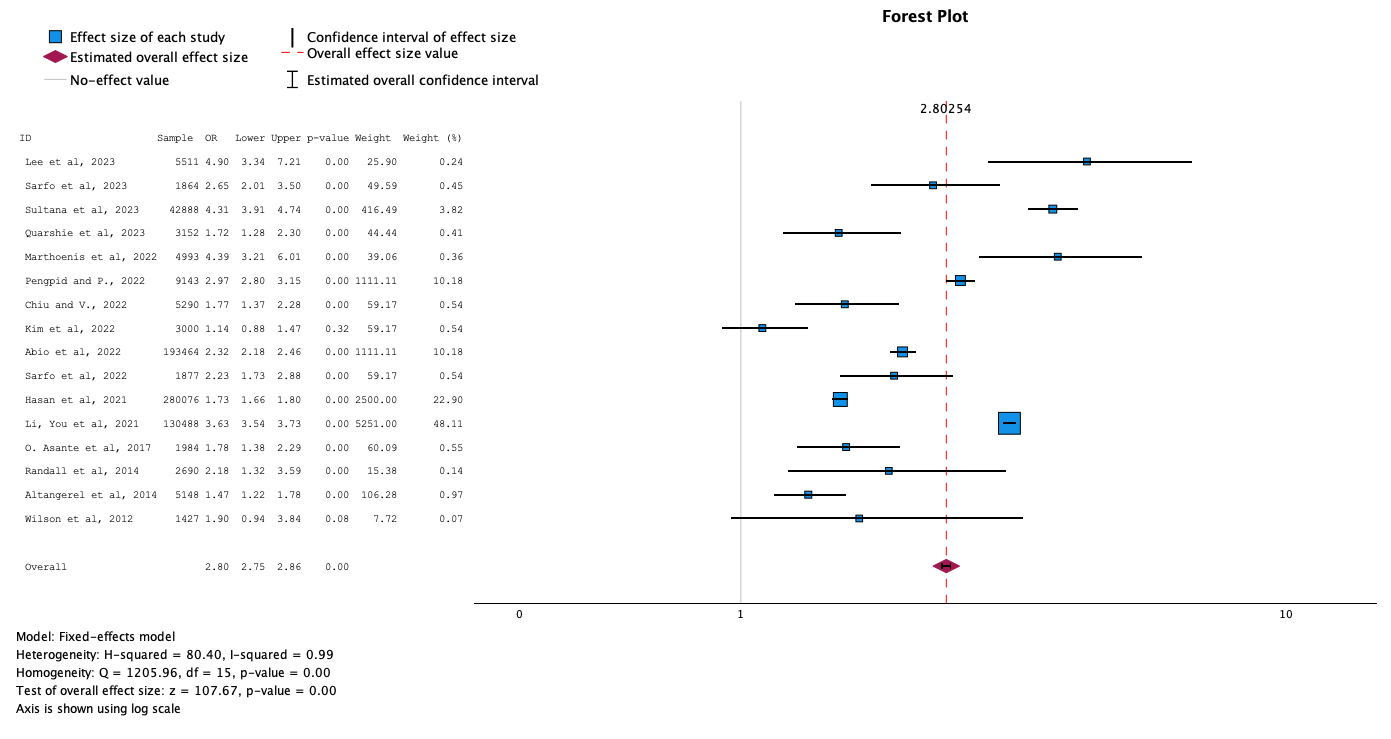

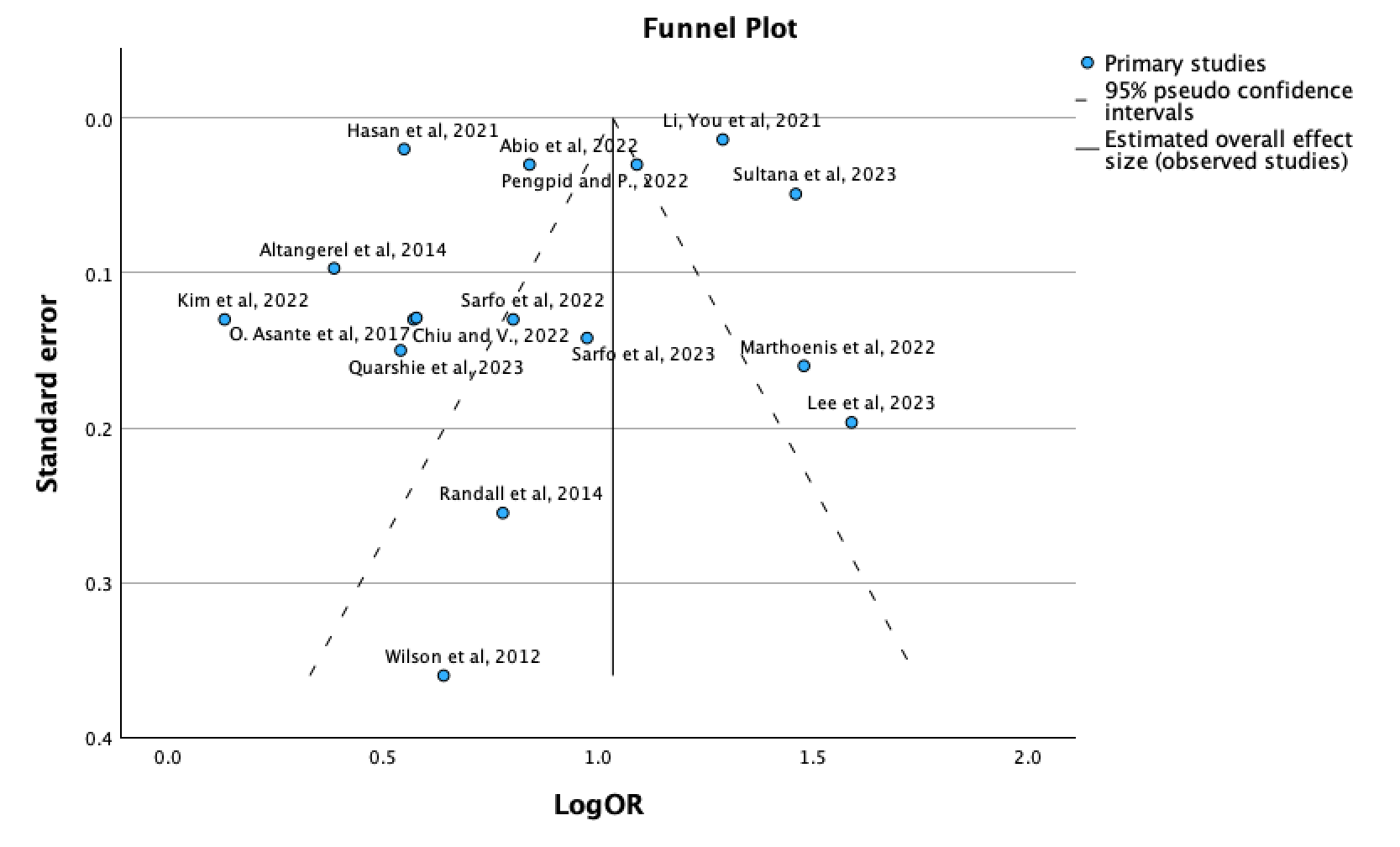


**
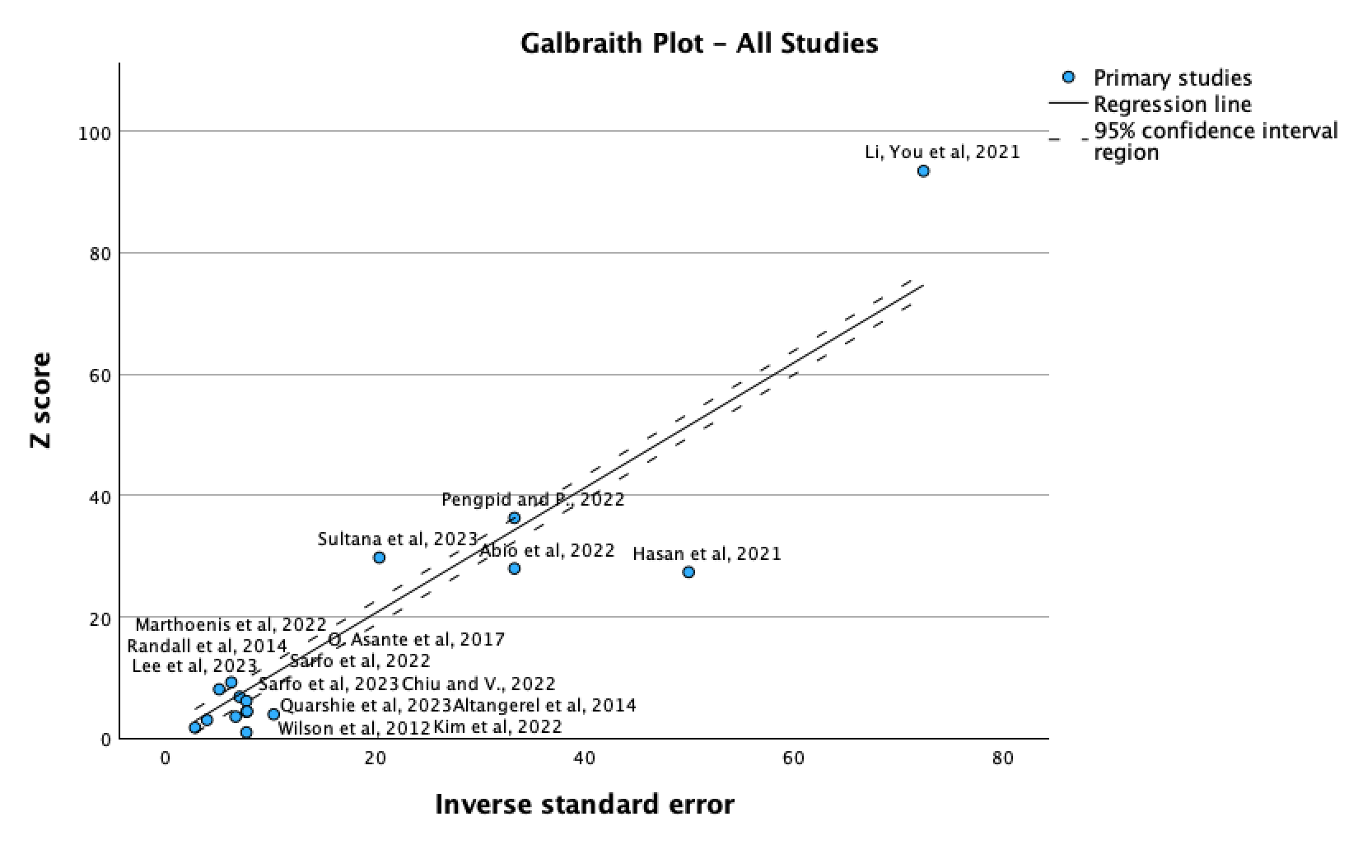
**

**Continuing graphic 4a.**

| **Egger's Regression-Based Test^a^** | | | | | | |
| --- | --- | --- | --- | --- | --- | --- |
| Parameter | Coefficient | Std. Error | t | Sig. (2-tailed) | 95% Confidence Interval | |
|  |  |  |  |  | Lower | Upper |
| (Intercept) | 1·100 | ·0130 | 84·729 | <·001 | 1·072 | 1·128 |
| SE^b^ | -2·694 | ·3392 | -7·944 | <·001 | -3·422 | -1·967 |
| a. Fixed-effects meta-regression | | | | | | |
| b. Standard error of effect size | | | | | | |

| **Effect Size Estimates for Trim-and-Fill Analysis** | | | | | | | |
| --- | --- | --- | --- | --- | --- | --- | --- |
|  | Number | Effect Size | Std. Error | Z | Sig. (2-tailed) | 95% Confidence Interval | |
|  |  |  |  |  |  | Lower | Upper |
| Observed | 16 | 1·031 | ·0096 | 107·668 | <·001 | 1·012 | 1·049 |
| Observed + Imputed^a^ | 17 | 1·034 | ·0095 | 108·332 | <·001 | 1·015 | 1·053 |
| a. Number of imputed studies: 1 | | | | | | | |

Egger’s Test demonstrates risk of bias [CI: 1·072 to 1·128]. The Trim-and-Fill analysis found a difference in effect size between the observed and the observed plus imputed groups, but included only one hypothetical study and identified a low risk of bias. More than half of the studies demonstrated asymmetry in the funnel plot. It was used the Galbraith Plot to help found asymmetry studies. Two studies demonstrated high asymmetry ^138,246^. After adjusted for asymmetry the pooling date demonstrated moderate risk of heterogeneity with an OR of 2·06 [CI: 1·69-2·51; p<0·01; *I*^2^ = 53%; z = 7·20]. (Supplementary Graphics 4b, c). Egger’s Test did not demonstrate risk of bias [CI: -7·719 to 9·628].

**Graphic 4b. Association between loneliness and suicidal planning after asymmetry adjusted**


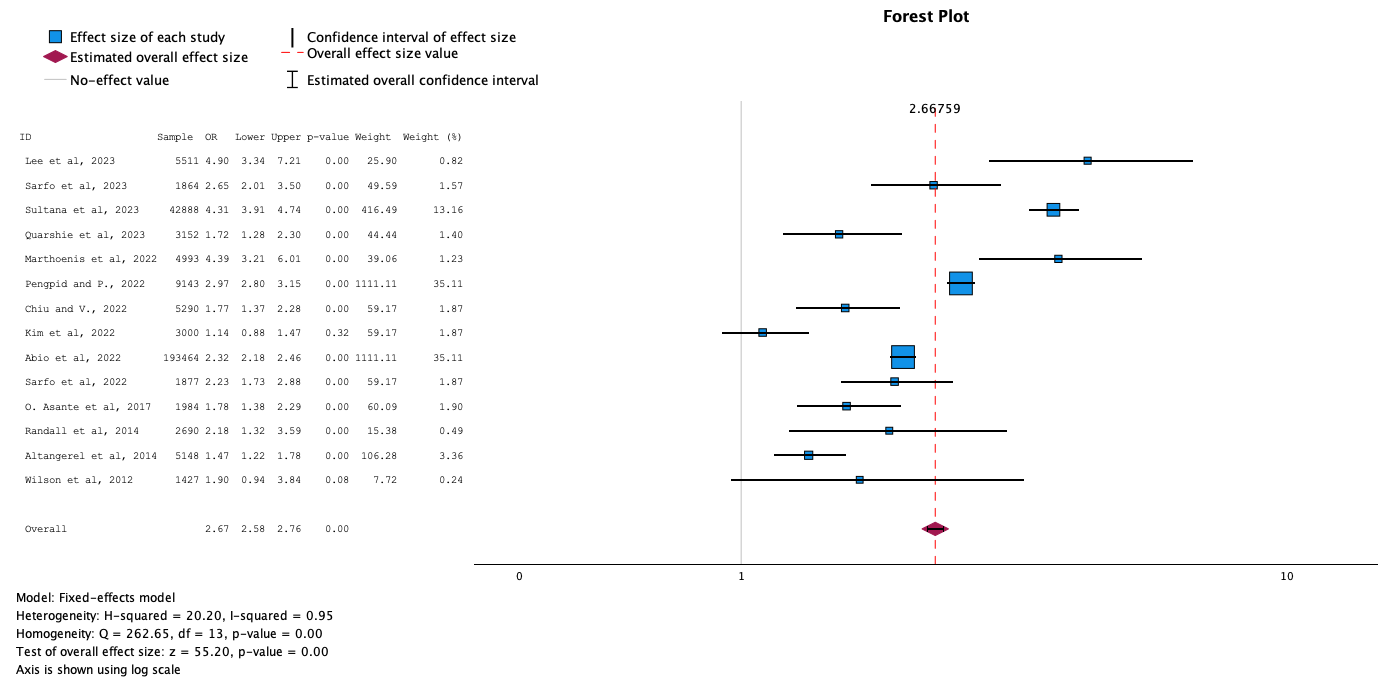

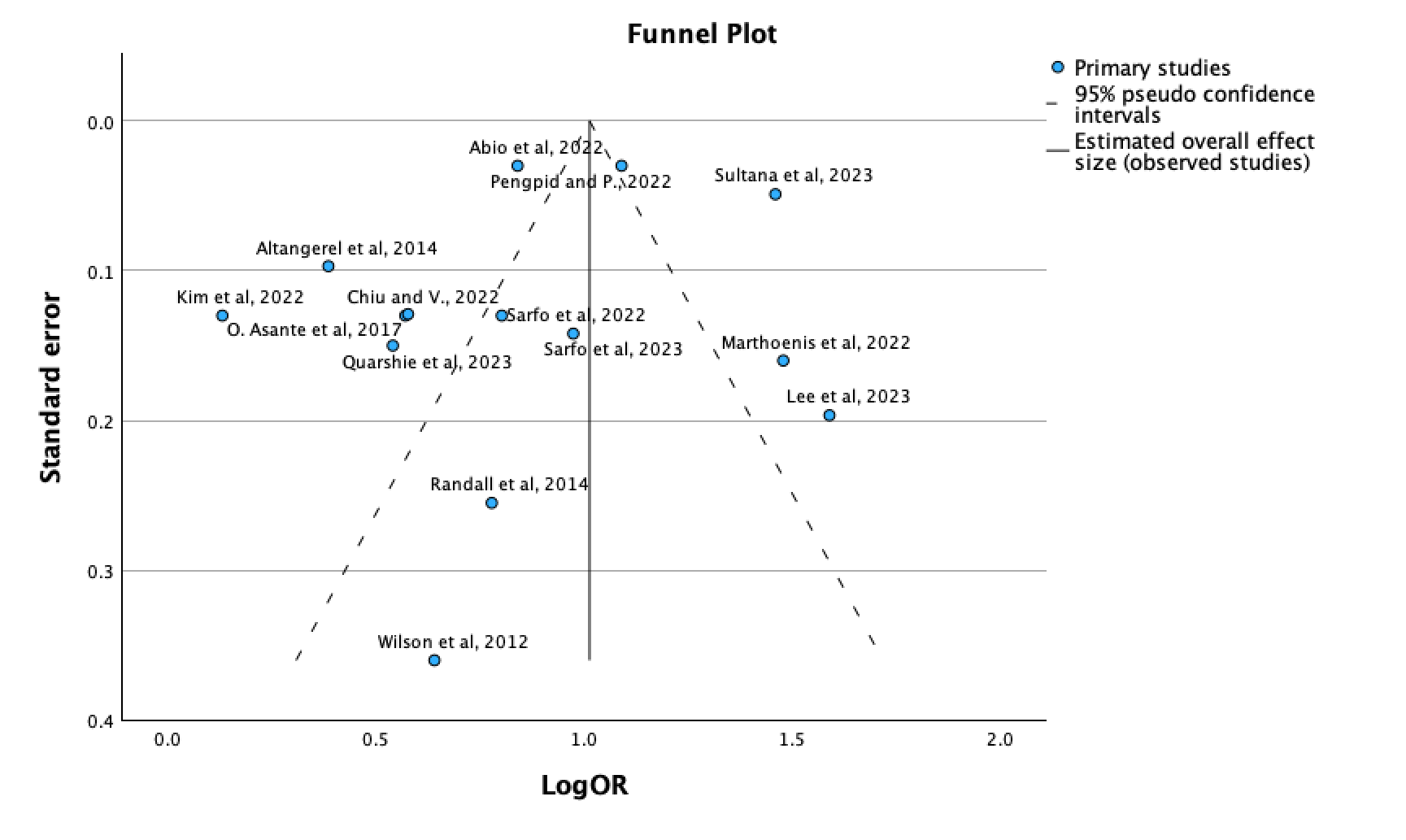


**
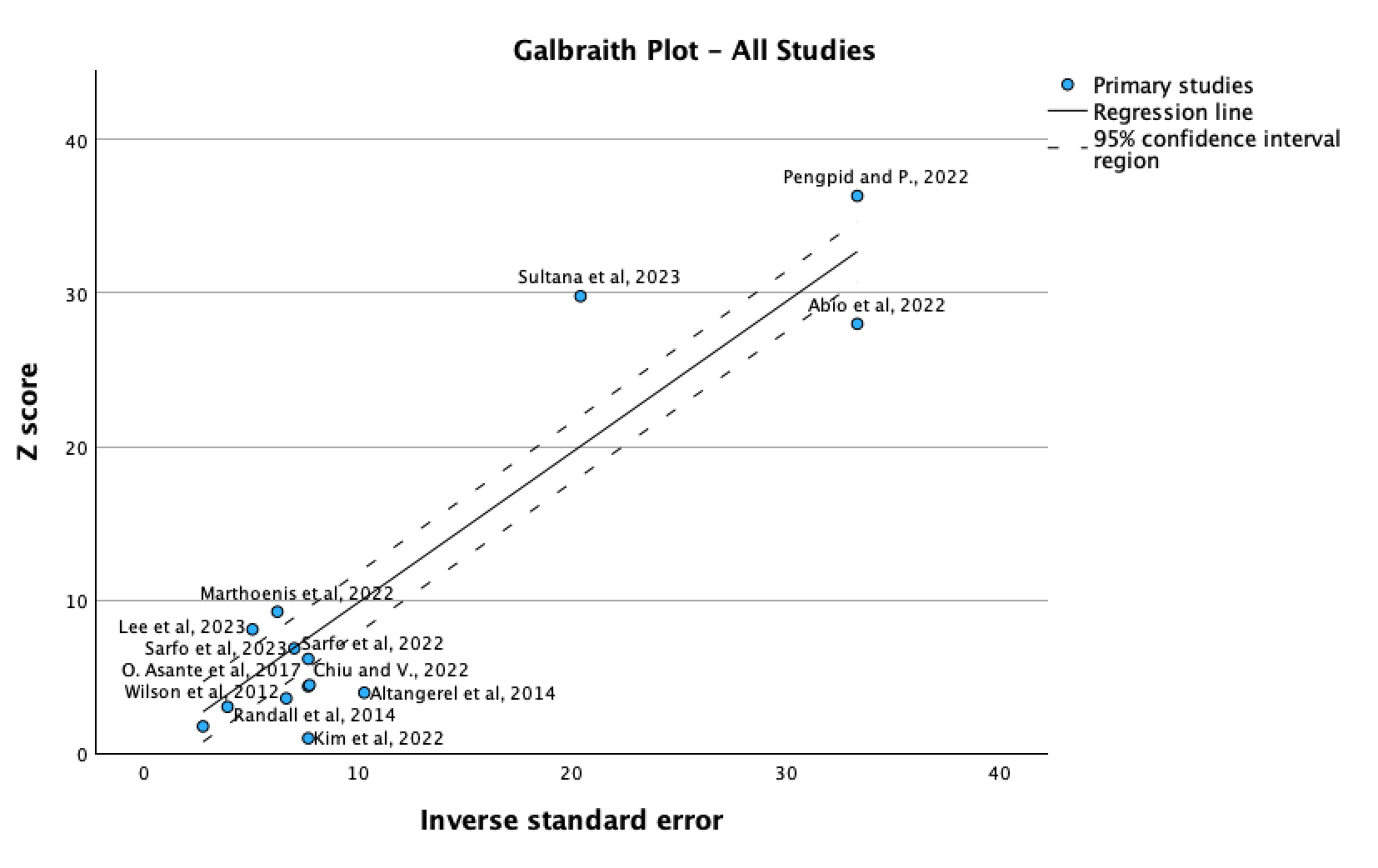

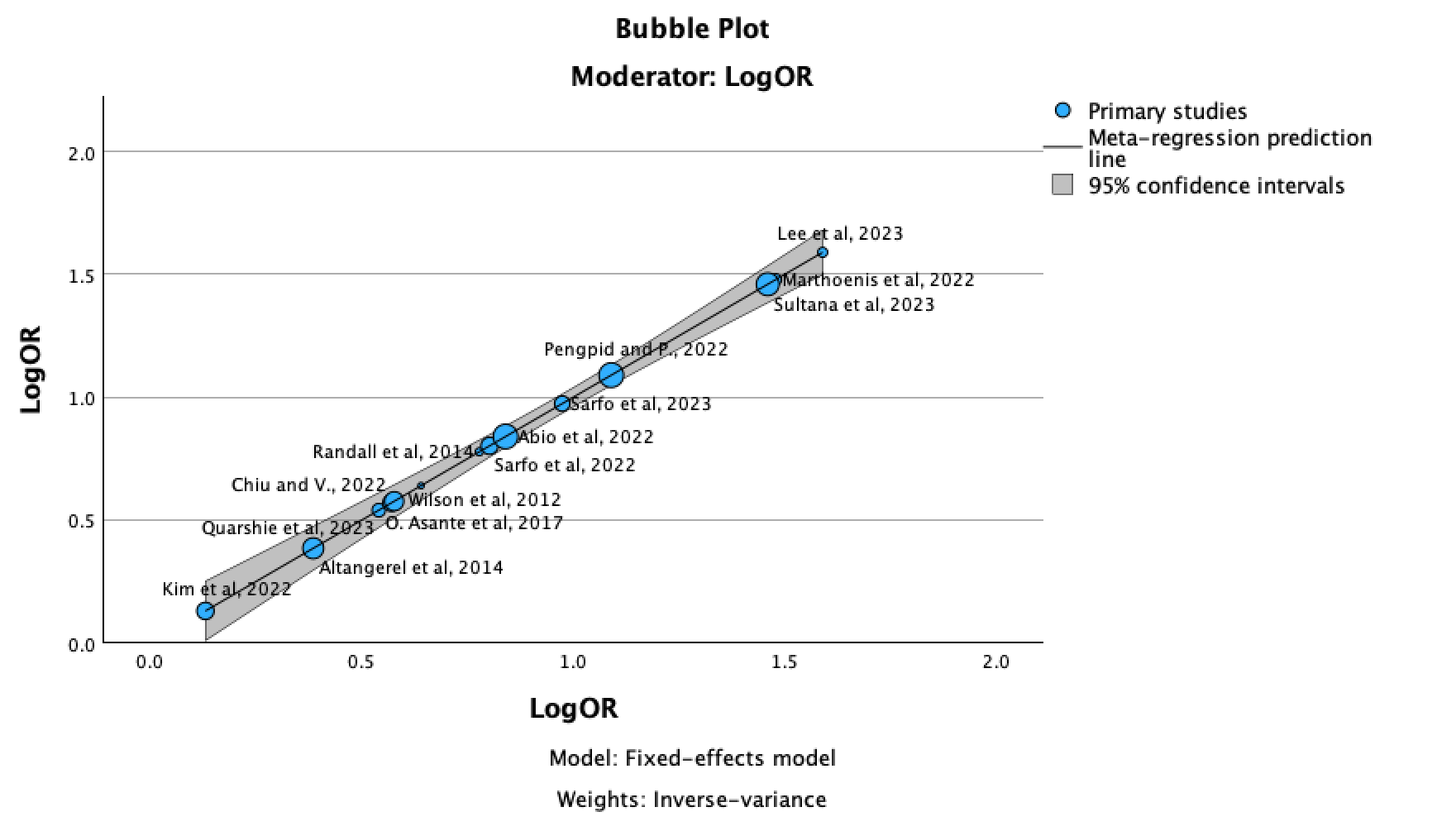
**

**Continuing graphic 4b.**

| **Egger's Regression-Based Test^a^** | | | | | | |
| --- | --- | --- | --- | --- | --- | --- |
| Parameter | Coefficient | Std. Error | t | Sig. (2-tailed) | 95% Confidence Interval | |
|  |  |  |  |  | Lower | Upper |
| (Intercept) | 1·068 | ·0273 | 39·066 | <·001 | 1·009 | 1·128 |
| SE^b^ | -1·725 | ·4112 | -4·196 | ·001 | -2·621 | -·829 |
| a. Fixed-effects meta-regression | | | | | | |
| b. Standard error of effect size | | | | | | |

| **Effect Size Estimates for Trim-and-Fill Analysis** | | | | | | | |
| --- | --- | --- | --- | --- | --- | --- | --- |
|  | Number | Effect Size | Std. Error | Z | Sig. (2-tailed) | 95% Confidence Interval | |
|  |  |  |  |  |  | Lower | Upper |
| Observed | 14 | ·981 | ·0178 | 55·197 | <·001 | ·946 | 1·016 |
| Observed + Imputed^a^ | 16 | 1·013 | ·0173 | 58·456 | <·001 | ·979 | 1·047 |
| a. Number of imputed studies: 2 | | | | | | | |

**Graphic 4c. Association between loneliness and suicidal planning after asymmetry adjusted 2**


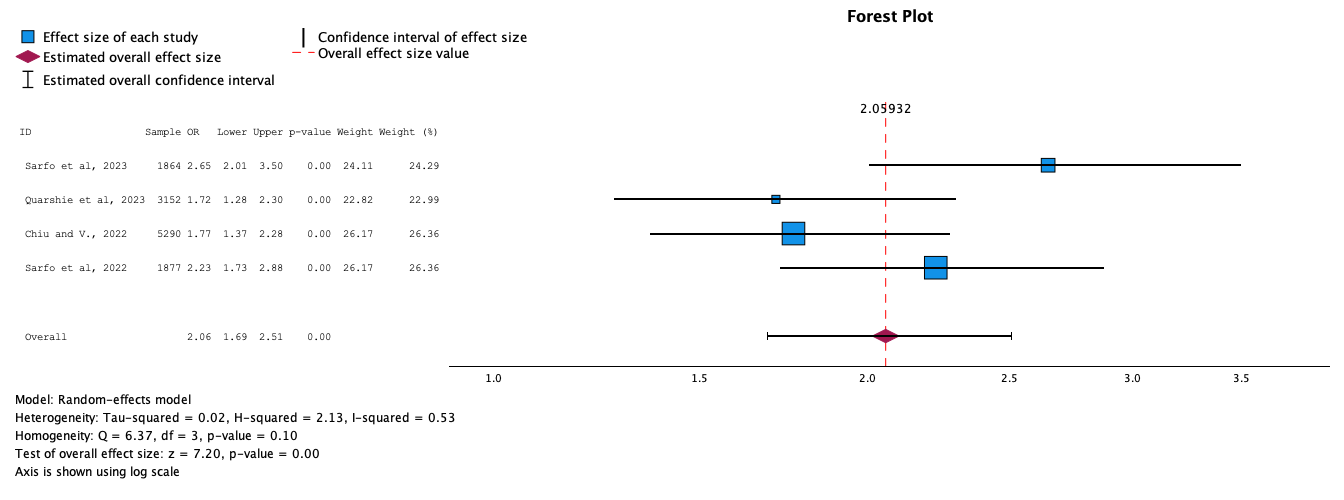

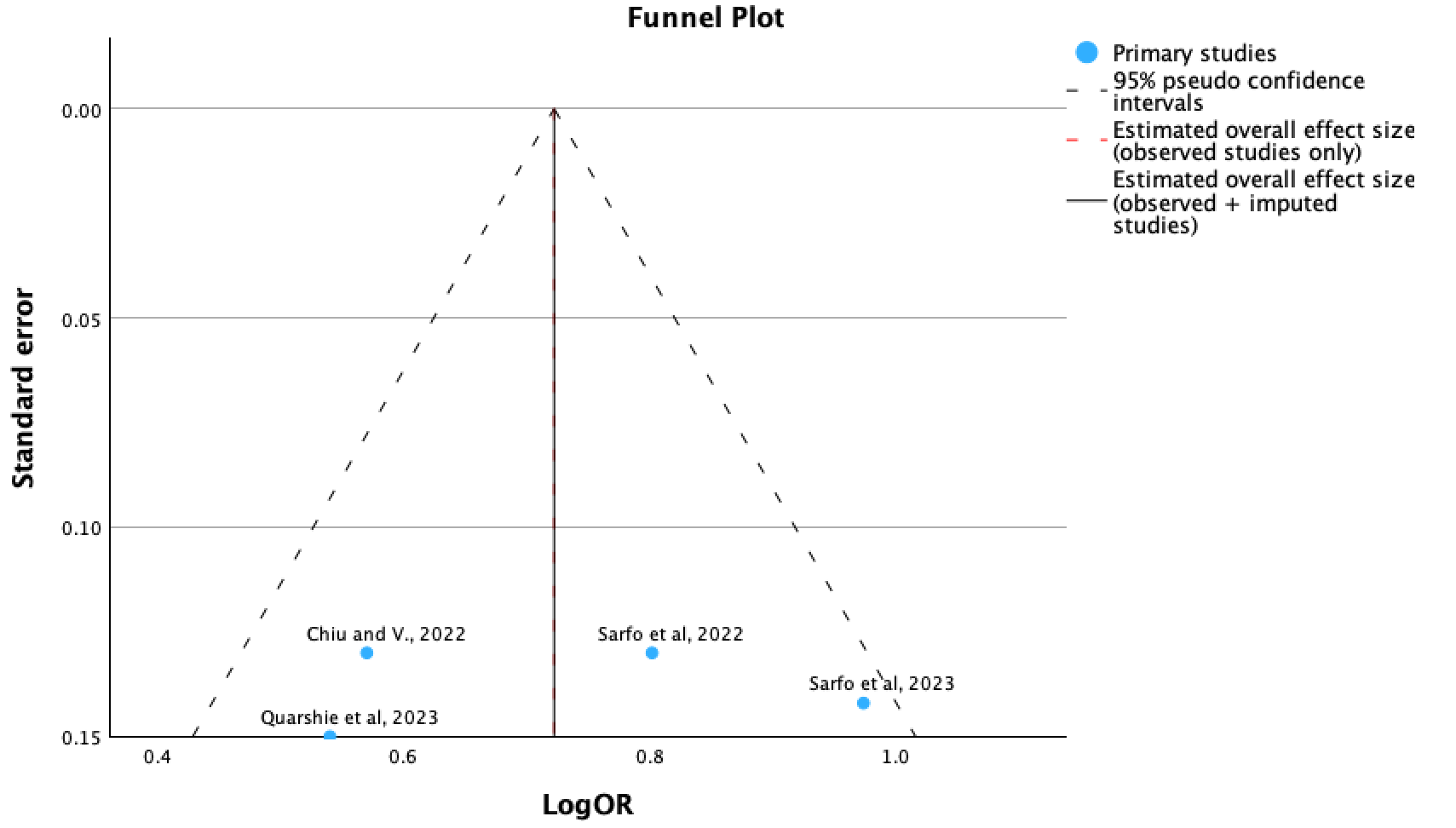


| **Egger's Regression-Based Test^a^** | | | | | | |
| --- | --- | --- | --- | --- | --- | --- |
| Parameter | Coefficient | Std. Error | t | Sig. (2-tailed) | 95% Confidence Interval | |
|  |  |  |  |  | Lower | Upper |
| (Intercept) | ·954 | 2·0159 | ·473 | ·683 | -7·719 | 9·628 |
| SE^b^ | -1·687 | 14·6150 | -·115 | ·919 | -64·570 | 61·197 |
| a. Random-effects meta-regression | | | | | | |
| b. Standard error of effect size | | | | | | |

**Graphic 5. Association between loneliness and suicide attempt.**


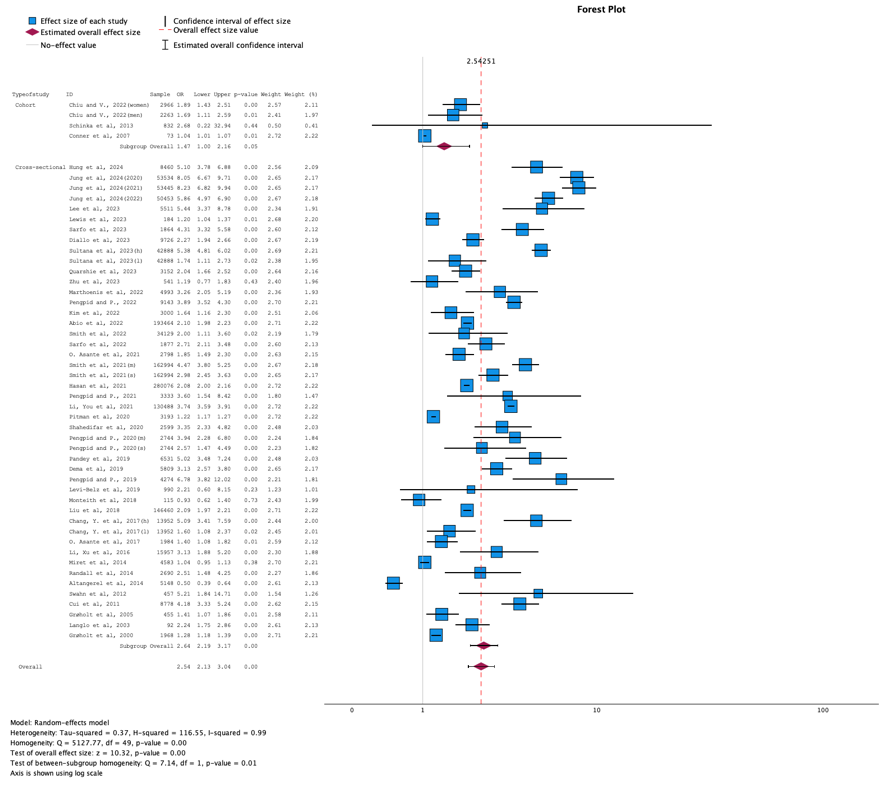

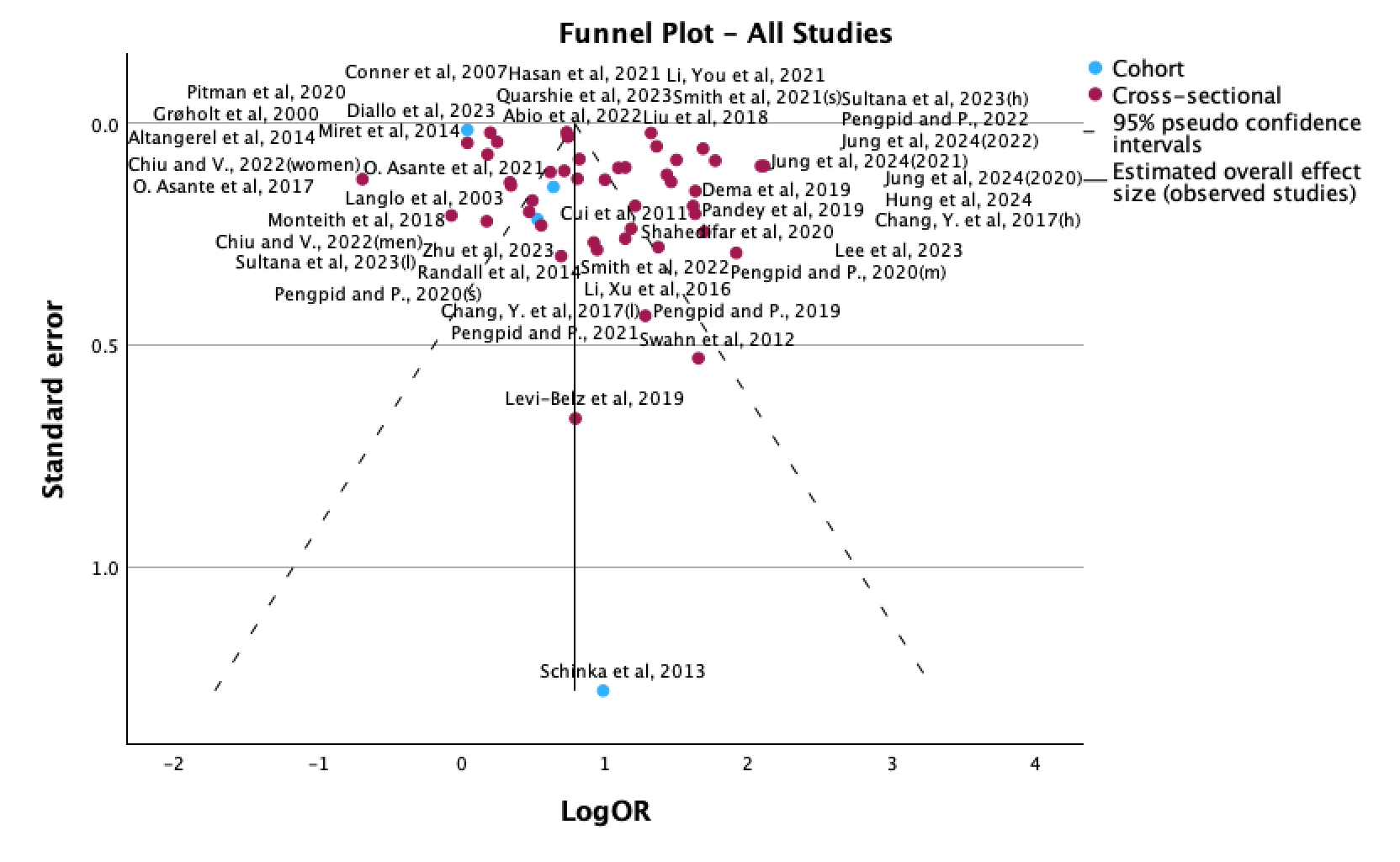


**Continuing graphic 5.**

| **Egger's Regression-Based Test^a^** | | | | | | | |
| --- | --- | --- | --- | --- | --- | --- | --- |
|  | Parameter | Coefficient | Std. Error | t | Sig. (2-tailed) | 95% Confidence Interval | |
|  |  |  |  |  |  | Lower | Upper |
| Cohort | (Intercept) | ·221 | ·1992 | 1·109 | ·383 | -·636 | 1·078 |
|  | SE^b^ | 1·143 | ·9664 | 1·183 | ·358 | -3·015 | 5·301 |
| Cross-sectional | (Intercept) | ·860 | ·1575 | 5·456 | <·001 | ·542 | 1·177 |
|  | SE^b^ | ·725 | ·8331 | ·870 | ·389 | -·954 | 2·404 |
| Overall | (Intercept) | ·835 | ·1385 | 6·031 | <·001 | ·557 | 1·114 |
|  | SE^b^ | ·634 | ·6816 | ·930 | ·357 | -·737 | 2·004 |
| a. Random-effects meta-regression | | | | | | | |
| b. Standard error of effect size | | | | | | | |

| **Effect Size Estimates for Trim-and-Fill Analysis** | | | | | | | |
| --- | --- | --- | --- | --- | --- | --- | --- |
|  | Number | Effect Size | Std. Error | Z | Sig. (2-tailed) | 95% Confidence Interval | |
|  |  |  |  |  |  | Lower | Upper |
| Observed | 50 | ·933 | ·0905 | 10·315 | <·001 | ·756 | 1·110 |
| Observed + Imputed^a^ | 57 | ·787 | ·0957 | 8·223 | <·001 | ·600 | ·975 |
| a. Number of imputed studies: 7 | | | | | | | |

The Trim-and-Fill analysis found a difference in effect size between the observed and the observed plus imputed groups and included seven hypothetical study, demonstrated a high risk of bias. More than half of the studies demonstrated asymmetry in the funnel plot. The data were recalculated in fixed-model and pooling data demonstrated high heterogeneity, with a OR of 1·79 [CI: 1·76-1·82; p<0·01; *I*^2^ = 99%; z = 77·45] (Supplementary Graphic 5a).

**Graphic 5a. Association between loneliness and suicide attempt in fixed-model.**


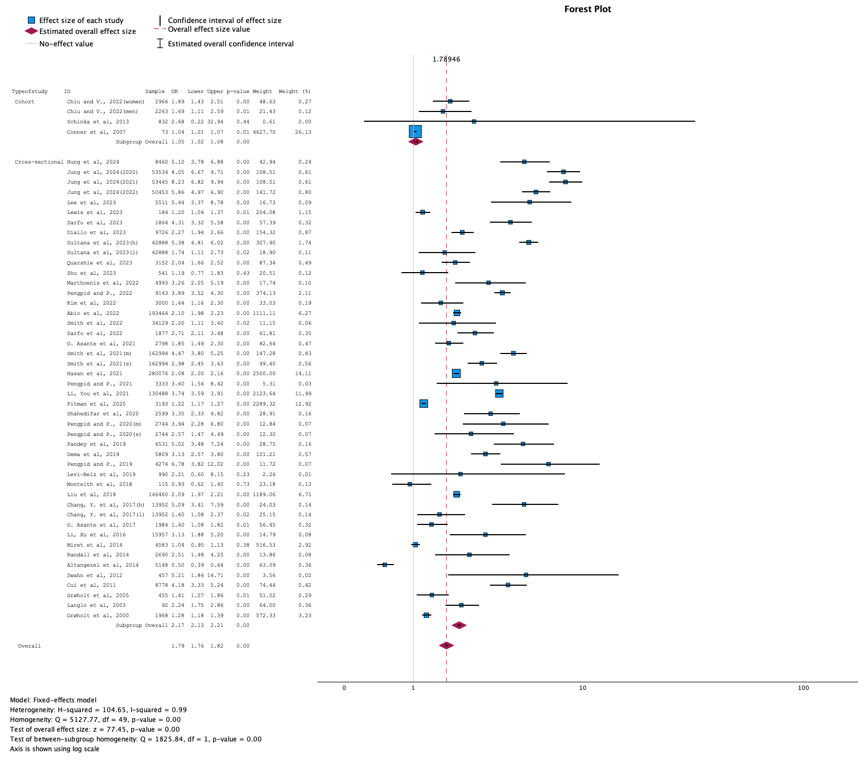

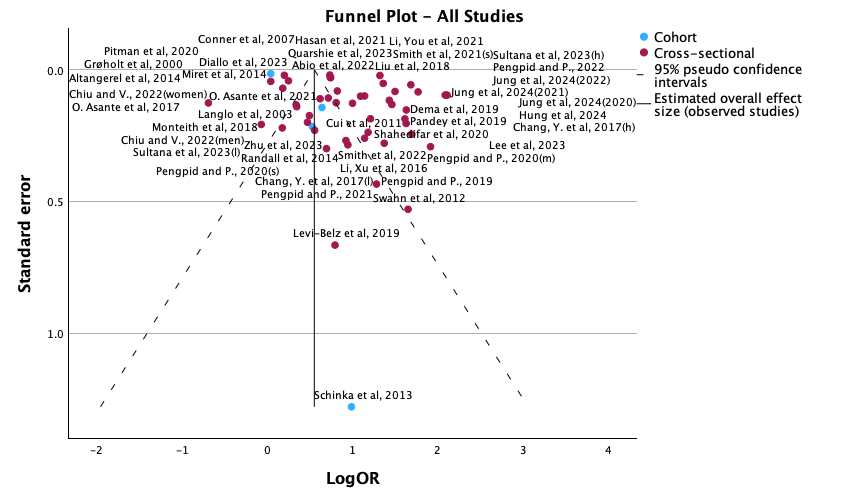


**Continuing graphic 5a.**

| **Egger's Regression-Based Test^a^** | | | | | | | |
| --- | --- | --- | --- | --- | --- | --- | --- |
|  | Parameter | Coefficient | Std. Error | t | Sig. (2-tailed) | 95% Confidence Interval | |
|  |  |  |  |  |  | Lower | Upper |
| Cohort | (Intercept) | ·005 | ·0180 | ·288 | ·800 | -·072 | ·083 |
|  | SE^b^ | 2·476 | ·6174 | 4·010 | ·057 | -·180 | 5·132 |
| Cross-sectional | (Intercept) | ·680 | ·0123 | 55·395 | <·001 | ·656 | ·705 |
|  | SE^b^ | 2·265 | ·2066 | 10·963 | <·001 | 1·849 | 2·682 |
| Overall | (Intercept) | ·431 | ·0100 | 42·976 | <·001 | ·410 | ·451 |
|  | SE^b^ | 4·308 | ·1886 | 22·845 | <·001 | 3·929 | 4·687 |
| a. Fixed-effects meta-regression | | | | | | | |
| b. Standard error of effect size | | | | | | | |

| **Effect Size Estimates for Trim-and-Fill Analysis** | | | | | | | |
| --- | --- | --- | --- | --- | --- | --- | --- |
|  | Number | Effect Size | Std. Error | Z | Sig. (2-tailed) | 95% Confidence Interval | |
|  |  |  |  |  |  | Lower | Upper |
| Observed | 50 | ·582 | ·0075 | 77·448 | <·001 | ·567 | ·597 |
| Observed + Imputed^a^ | 57 | ·550 | ·0074 | 74·602 | <·001 | ·535 | ·564 |
| a. Number of imputed studies: 7 | | | | | | | |

Egger’s Test demonstrates risk of bias [CI: 0·410 to 0·451]. The Trim-and-Fill analysis found a difference in effect size between the observed and the observed plus imputed groups and included seven hypothetical study, demonstrated a high risk of bias. More than half of the studies demonstrated asymmetry in the funnel plot. Statistical analyses began to be carried out by groups of study types.

**Graphic 5b. Association between loneliness and suicide attempt in longitudinal studies.**


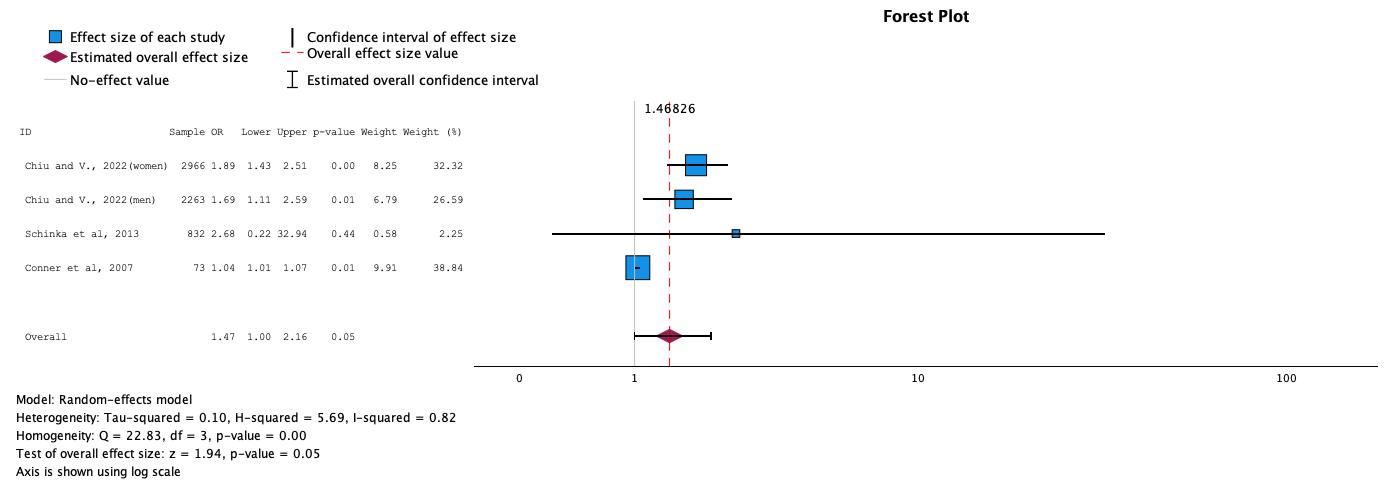

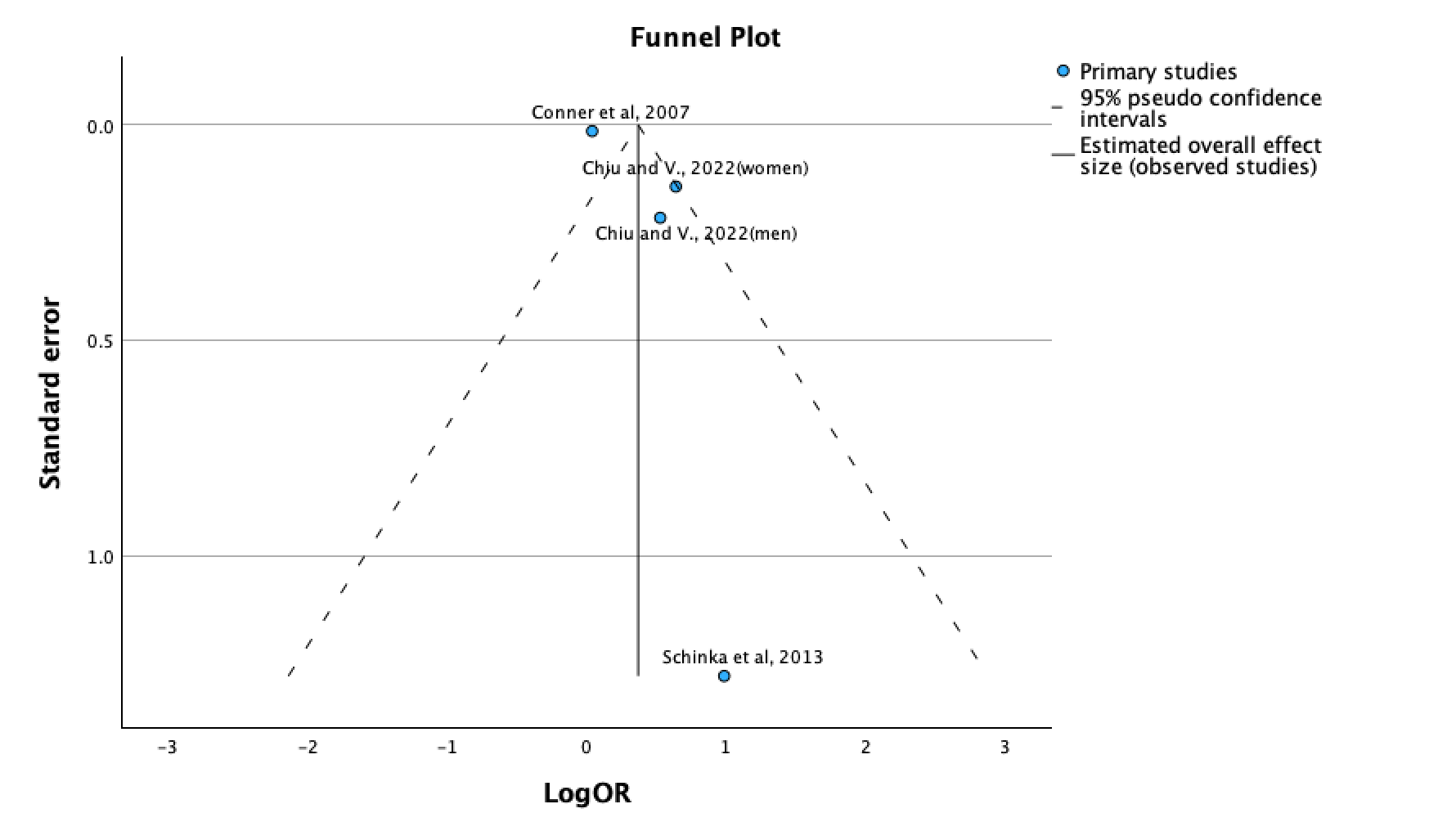


| **Egger's Regression-Based Test^a^** | | | | | | |
| --- | --- | --- | --- | --- | --- | --- |
| Parameter | Coefficient | Std. Error | t | Sig. (2-tailed) | 95% Confidence Interval | |
|  |  |  |  |  | Lower | Upper |
| (Intercept) | ·221 | ·1992 | 1·109 | ·383 | -·636 | 1·078 |
| SE^b^ | 1·143 | ·9664 | 1·183 | ·358 | -3·015 | 5·301 |
| a. Random-effects meta-regression | | | | | | |
| b. Standard error of effect size | | | | | | |

Z score demonstrated a low effect. Egger’s Test did not demonstrate risk of bias [CI: -0·636 to 1·078]. One study demonstrated asymmetry in the funnel plot. The data were recalculated in fixed-model and pooling data demonstrated a high heterogeneity with an OR of 1·05 [CI: 1·02-1·08; p<0·01; *I*^2^ = 87%; z = 3·26]. (Supplementary Graphic 5c).

**Graphic 5c. Association between loneliness and suicide attempt in longitudinal studies in fixed-model.**


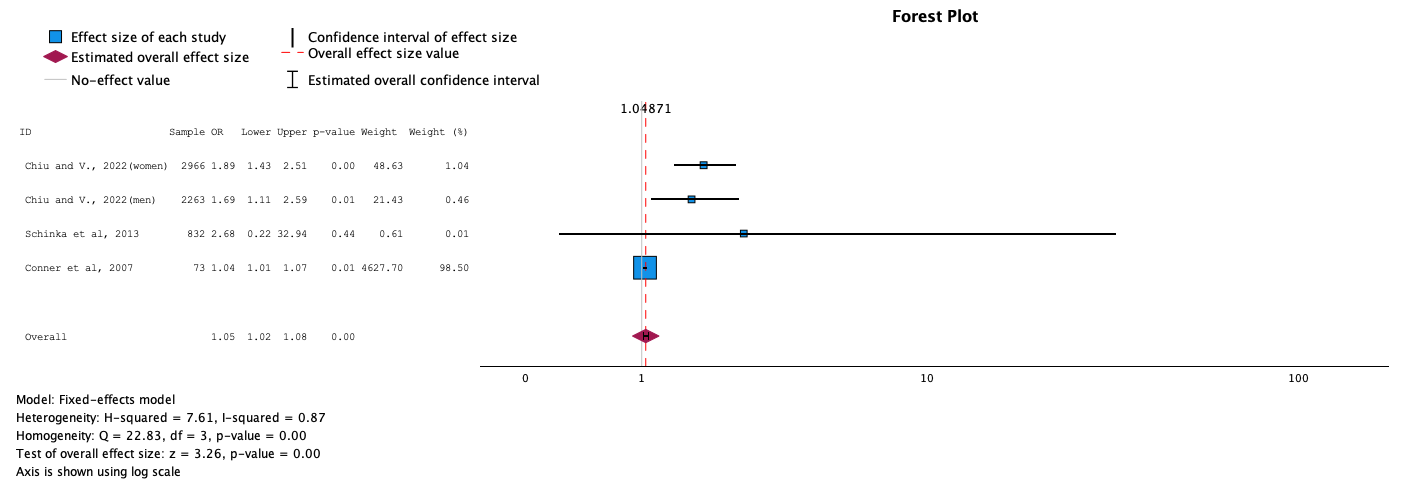

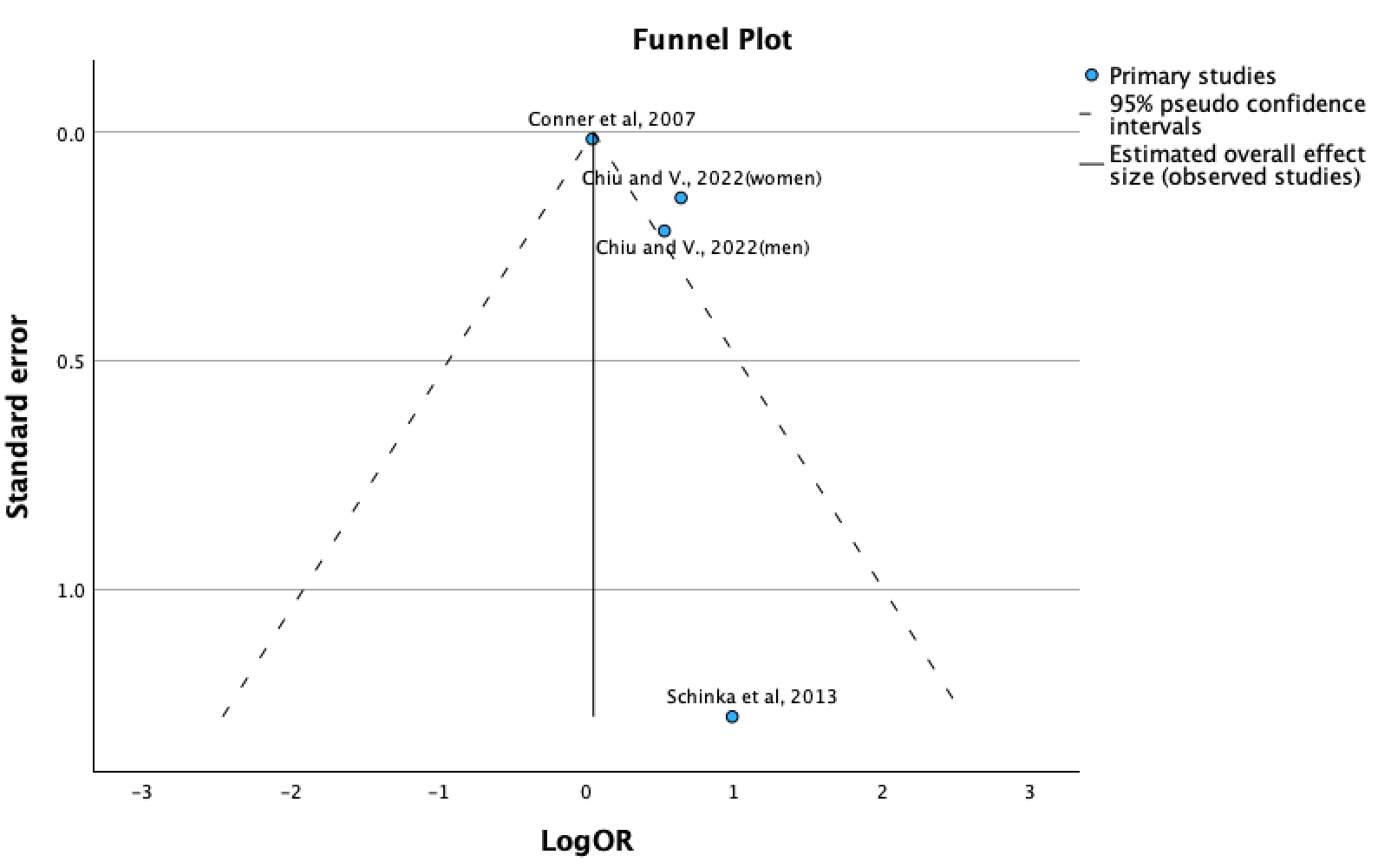


| **Egger's Regression-Based Test^a^** | | | | | | |
| --- | --- | --- | --- | --- | --- | --- |
| Parameter | Coefficient | Std. Error | t | Sig. (2-tailed) | 95% Confidence Interval | |
|  |  |  |  |  | Lower | Upper |
| (Intercept) | ·005 | ·0180 | ·288 | ·800 | -·072 | ·083 |
| SE^b^ | 2·476 | ·6174 | 4·010 | ·057 | -·180 | 5·132 |
| a. Fixed-effects meta-regression | | | | | | |
| b. Standard error of effect size | | | | | | |

Egger’s Test did not demonstrate risk of bias [CI: -0·072 to 0·083]. One study, that was made sex analyzed, demonstrated women asymmetry in the funnel plot ^235^. After adjusted for asymmetry the pooling date demonstrated moderate heterogeneity with an OR of 1·04 [CI: 1·01-1·07; p<0·01; *I*^2^ = 64%; z = 2·82]. (Supplementary Graphic 5d). Egger’s Test did not demonstrate risk of bias [CI: -0·221 to 0·225].

**Graphic 5d. Association between loneliness and suicide attempt in longitudinal studies after asymmetry adjusted.**


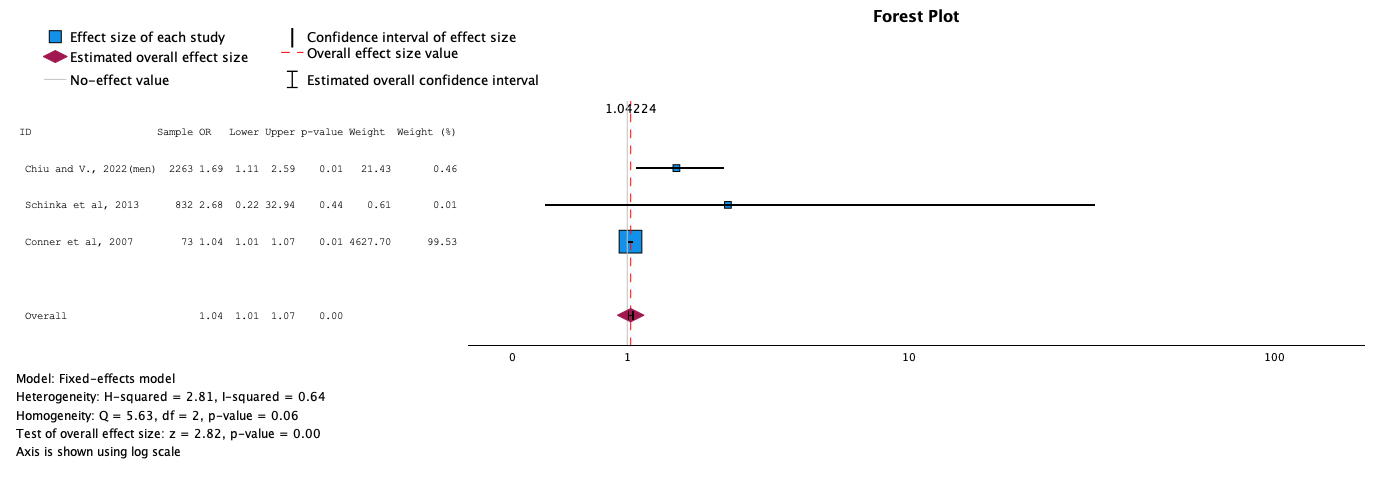

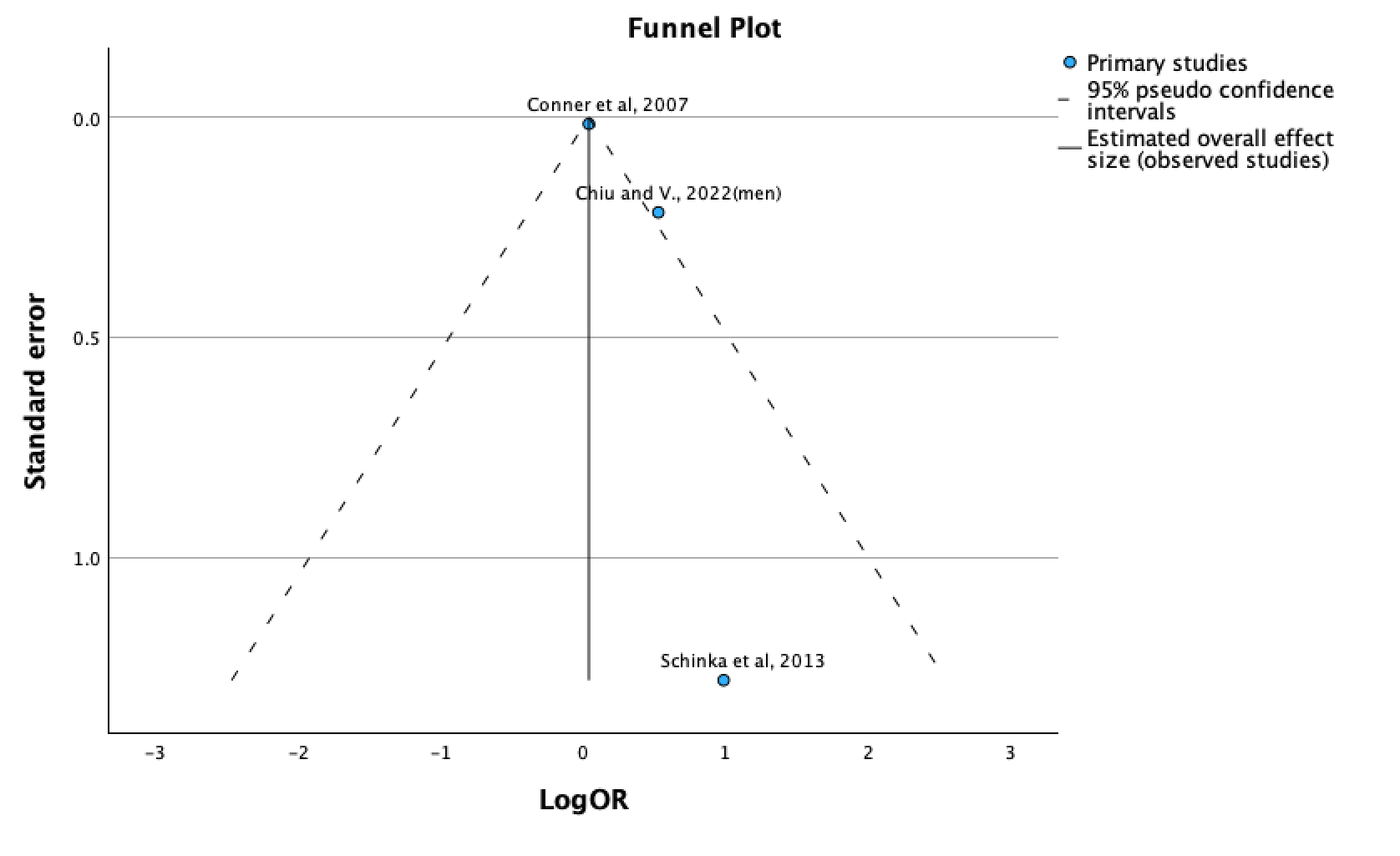


| **Egger's Regression-Based Test^a^** | | | | | | |
| --- | --- | --- | --- | --- | --- | --- |
| Parameter | Coefficient | Std. Error | t | Sig. (2-tailed) | 95% Confidence Interval | |
|  |  |  |  |  | Lower | Upper |
| (Intercept) | ·017 | ·0187 | ·915 | ·528 | -·221 | ·255 |
| SE^b^ | 1·535 | ·7372 | 2·082 | ·285 | -7·832 | 10·902 |
| a. Fixed-effects meta-regression | | | | | | |
| b. Standard error of effect size | | | | | | |

**Graphic 5e. Association between loneliness and suicide attempt in cross-sectional studies.**


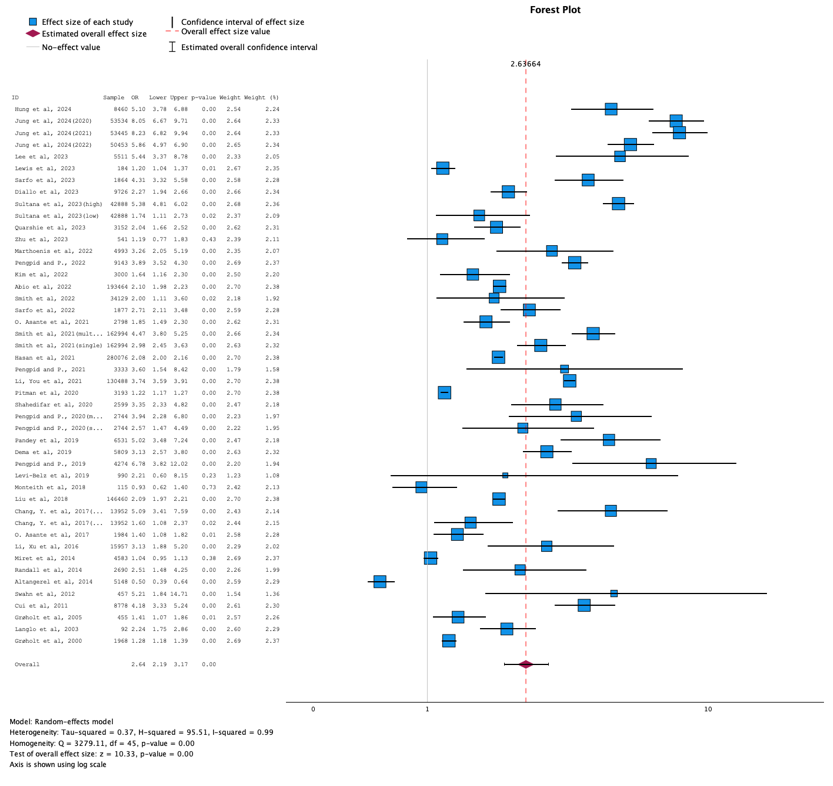

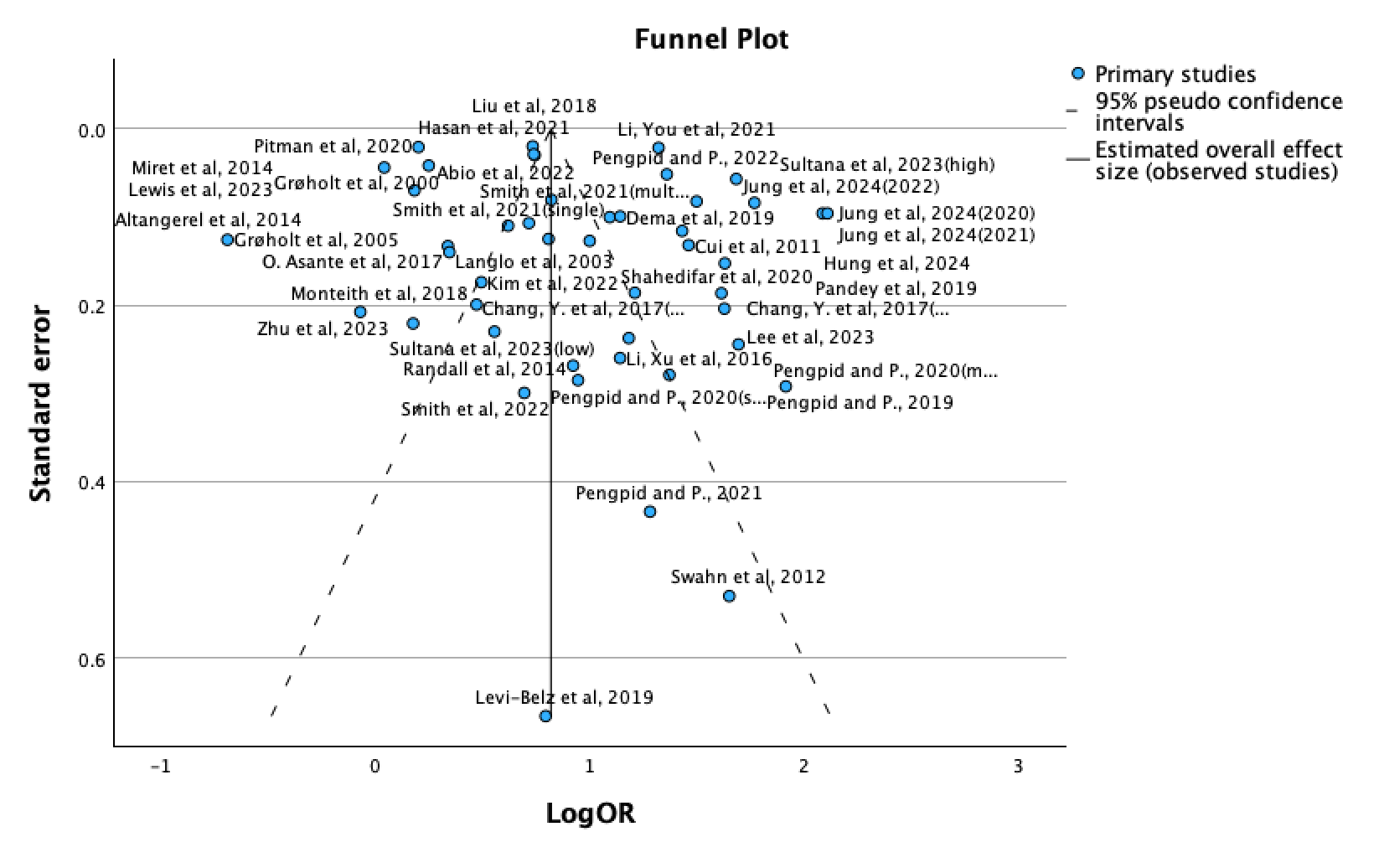


**Continuing graphic 5e.**

| **Egger's Regression-Based Test^a^** | | | | | | |
| --- | --- | --- | --- | --- | --- | --- |
| Parameter | Coefficient | Std. Error | t | Sig. (2-tailed) | 95% Confidence Interval | |
|  |  |  |  |  | Lower | Upper |
| (Intercept) | ·860 | ·1575 | 5·456 | <·001 | ·542 | 1·177 |
| SE^b^ | ·725 | ·8331 | ·870 | ·389 | -·954 | 2·404 |
| a. Random-effects meta-regression | | | | | | |
| b. Standard error of effect size | | | | | | |

| **Effect Size Estimates for Trim-and-Fill Analysis** | | | | | | | |
| --- | --- | --- | --- | --- | --- | --- | --- |
|  | Number | Effect Size | Std. Error | Z | Sig. (2-tailed) | 95% Confidence Interval | |
|  |  |  |  |  |  | Lower | Upper |
| Observed | 46 | ·970 | ·0939 | 10·326 | <·001 | ·785 | 1·154 |
| Observed + Imputed^a^ | 53 | ·817 | ·0988 | 8·268 | <·001 | ·624 | 1·011 |
| a. Number of imputed studies: 7 | | | | | | | |

Egger’s Test demonstrates risk of bias [CI: 0·542 to 1·177]. The Trim-and-Fill analysis found a difference in effect size between the observed and the observed plus imputed groups and included seven hypothetical study, demonstrated a high risk of bias. More than half of the studies demonstrated asymmetry in the funnel plot. The data were recalculated in fixed-model and pooling data demonstrated a high heterogeneity with an OR of 2·17 [CI: 2·13-2·21; p<0·01; *I*^2^ = 99%; z = 88·39]. (Supplementary Graphic 5f).

**Graphic 5f. Association between loneliness and suicide attempt in cross-sectional studies in fixed-model.**


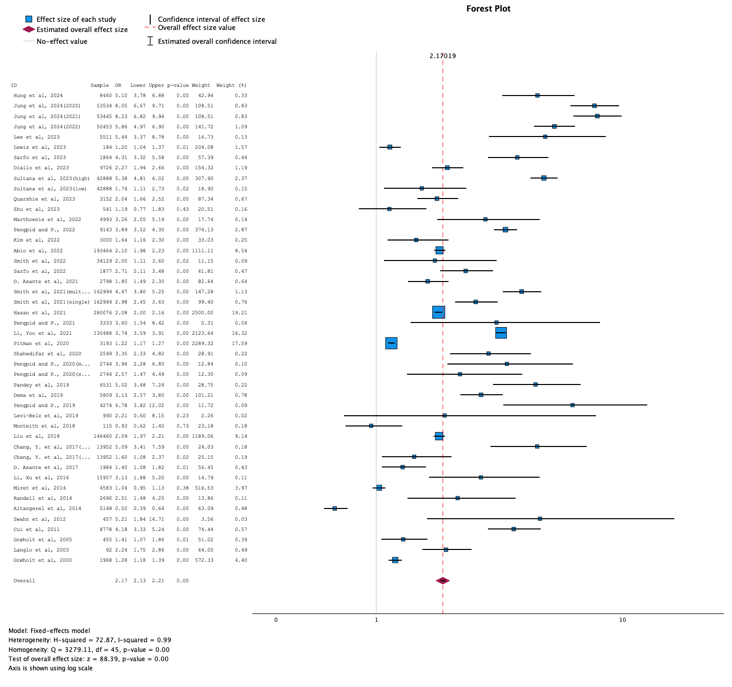

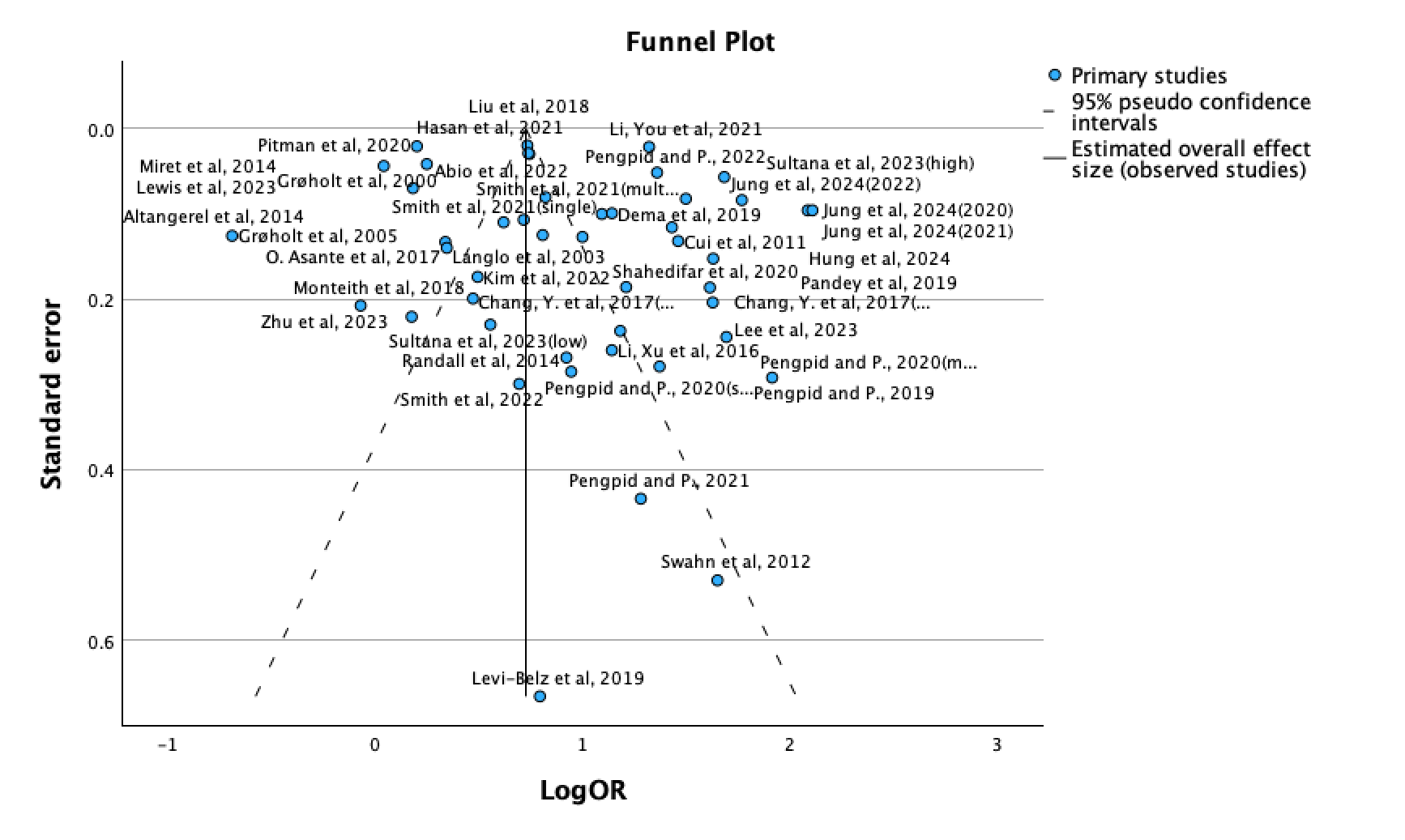


**
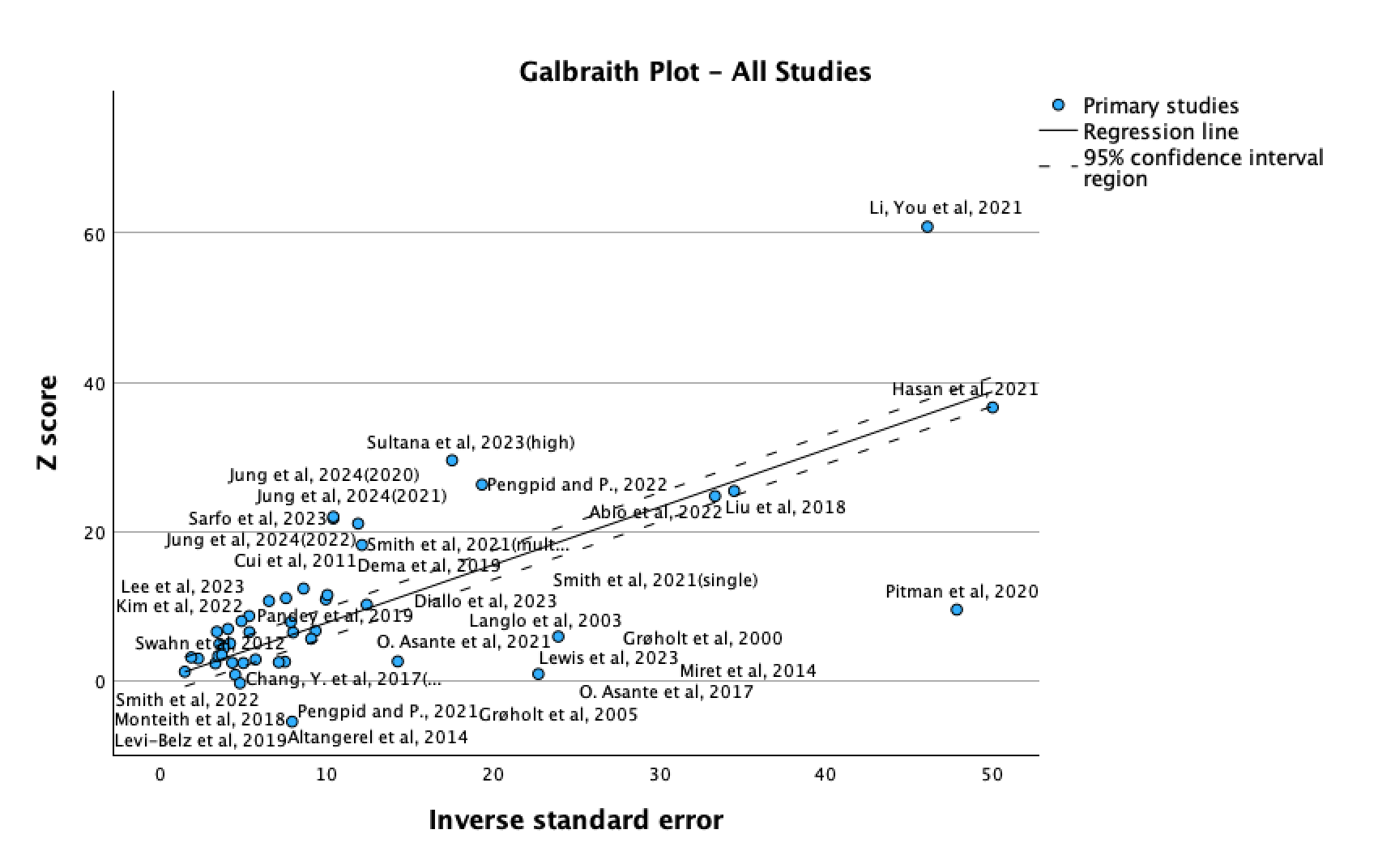

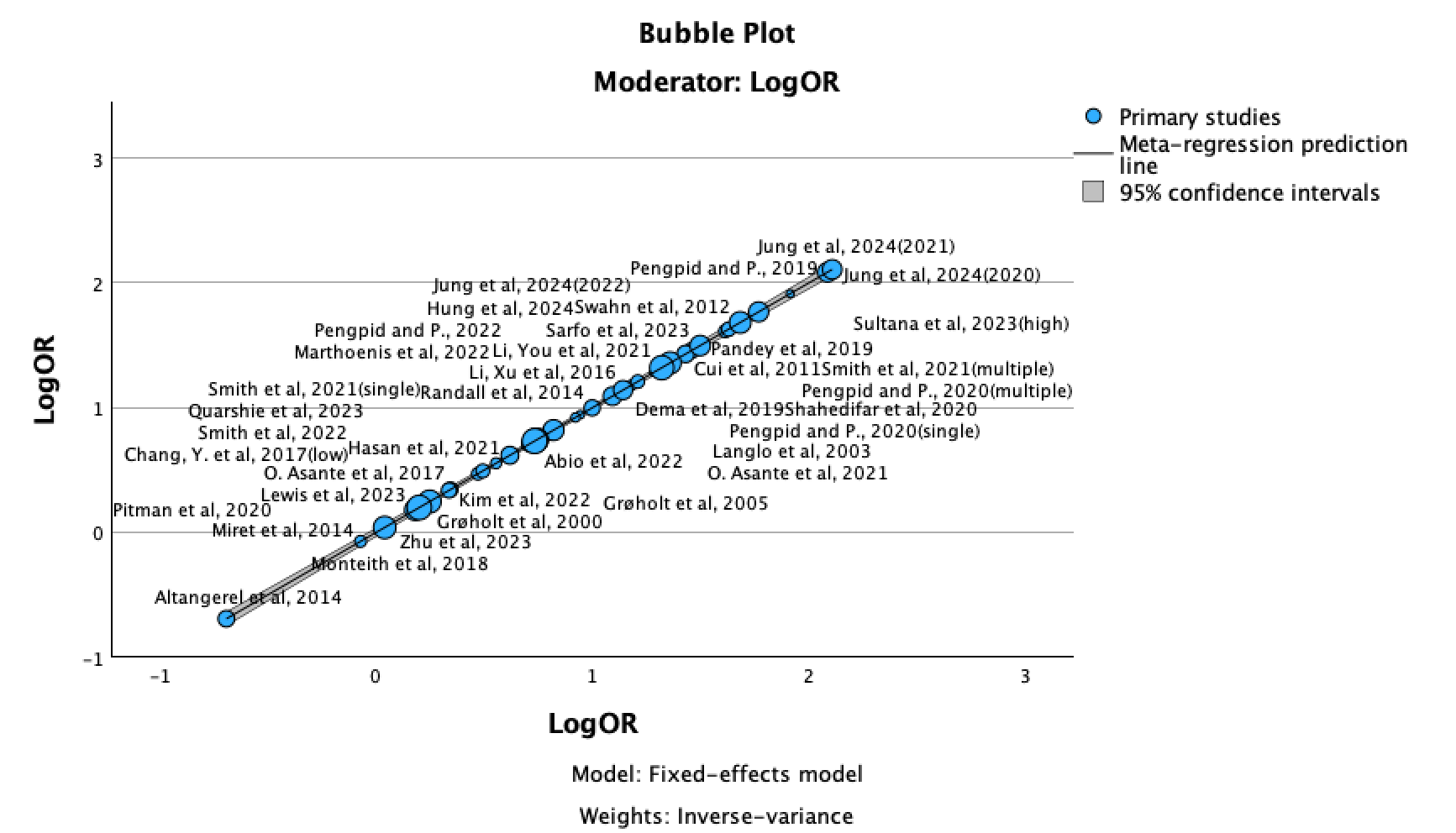
**

**Continuing graphic 5f.**

| **Egger's Regression-Based Test^a^** | | | | | | |
| --- | --- | --- | --- | --- | --- | --- |
| Parameter | Coefficient | Std. Error | t | Sig. (2-tailed) | 95% Confidence Interval | |
|  |  |  |  |  | Lower | Upper |
| (Intercept) | ·680 | ·0123 | 55·395 | <·001 | ·656 | ·705 |
| SE^b^ | 2·265 | ·2066 | 10·963 | <·001 | 1·849 | 2·682 |
| a. Fixed-effects meta-regression | | | | | | |
| b. Standard error of effect size | | | | | | |

| **Effect Size Estimates for Trim-and-Fill Analysis** | | | | | | | |
| --- | --- | --- | --- | --- | --- | --- | --- |
|  | Number | Effect Size | Std. Error | Z | Sig. (2-tailed) | 95% Confidence Interval | |
|  |  |  |  |  |  | Lower | Upper |
| Observed | 46 | ·775 | ·0088 | 88·393 | <·001 | ·758 | ·792 |
| Observed + Imputed^a^ | 53 | ·725 | ·0085 | 84·901 | <·001 | ·708 | ·742 |
| a. Number of imputed studies: 7 | | | | | | | |

Egger’s Test demonstrates risk of bias [CI: 0·656 to 0·705]. The Trim-and-Fill analysis found a difference in effect size between the observed and the observed plus imputed groups and included seven hypothetical study, demonstrated a high risk of bias. More than half of the studies demonstrated asymmetry in the funnel plot. It was used the Galbraith Plot to help found asymmetry studies. Two studies demonstrated high asymmetry.^138,140^ After adjusted for asymmetry the pooling date demonstrated low risk of heterogeneity with an OR of 3·55 [CI: 3·09-4·08; p<0·01; *I*^2^ = 40% z = 17·78]. (Supplementary Graphic 5g, h). Egger’s Test demonstrates risk of bias [CI: 0·860 to 1·581]. The Trim-and-Fill analysis found a difference in effect size between the observed and the observed plus imputed groups, but included only one hypothetical study and identified a low risk of bias.

**Graphic 5g. Association between loneliness and suicide attempt in cross-sectional studies after asymmetry adjusted.**


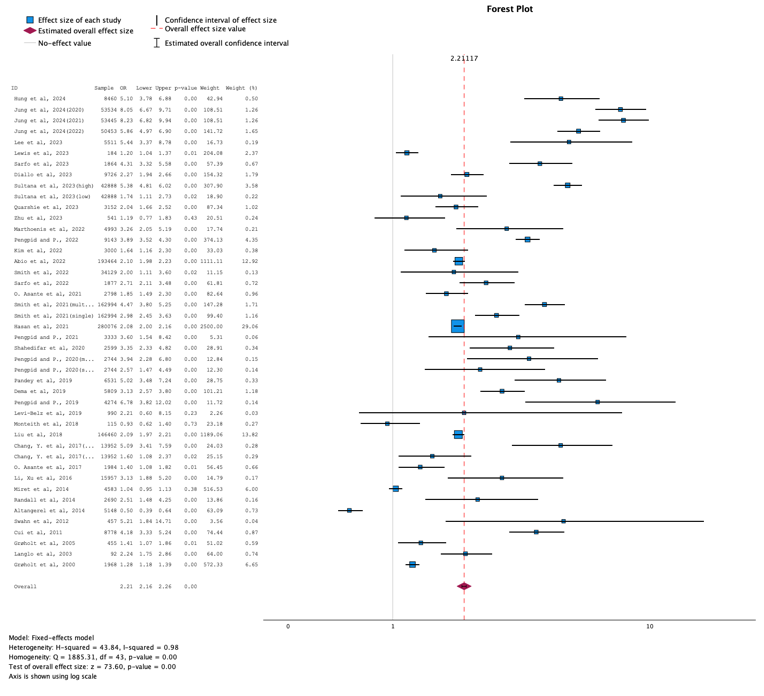

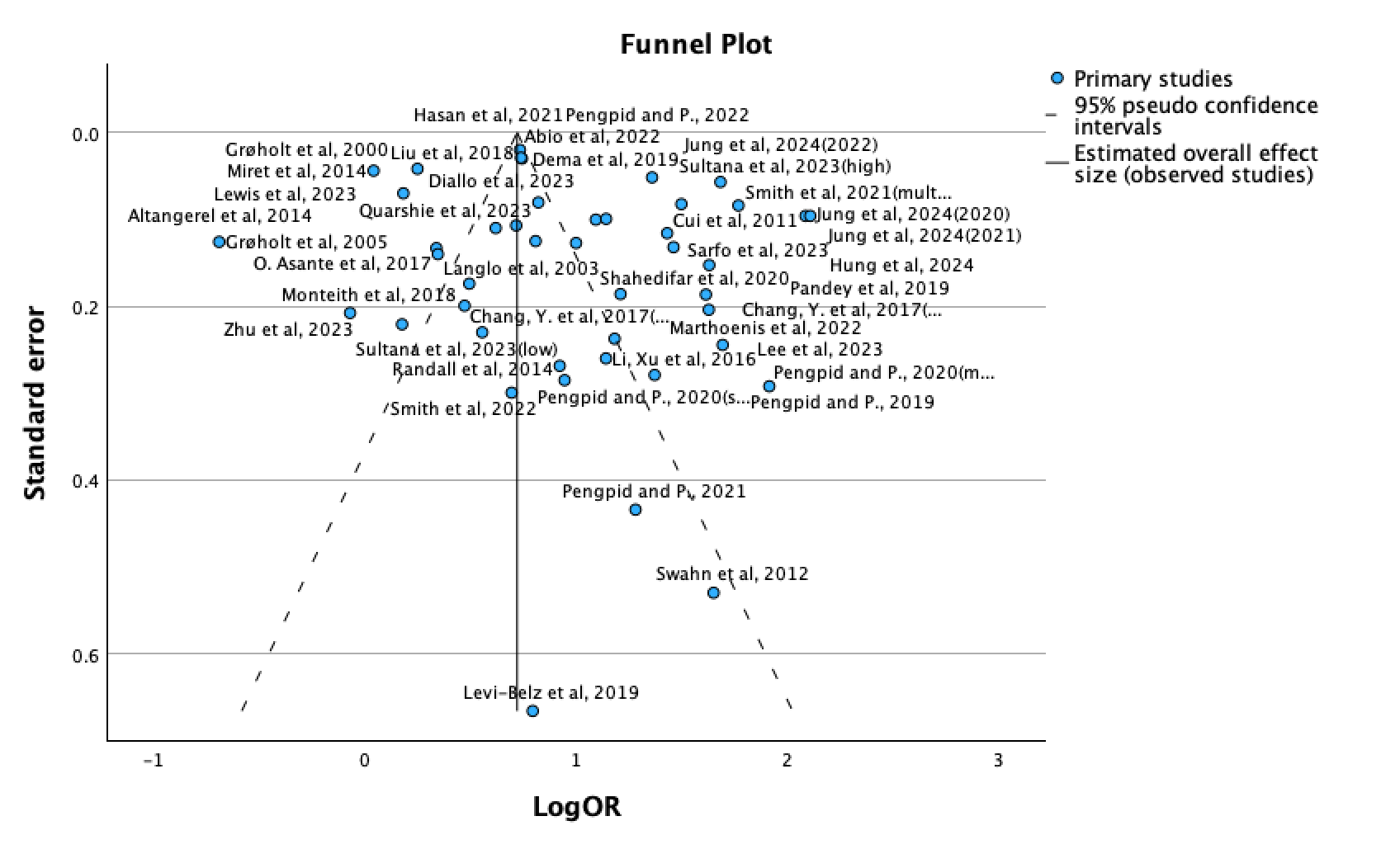


**
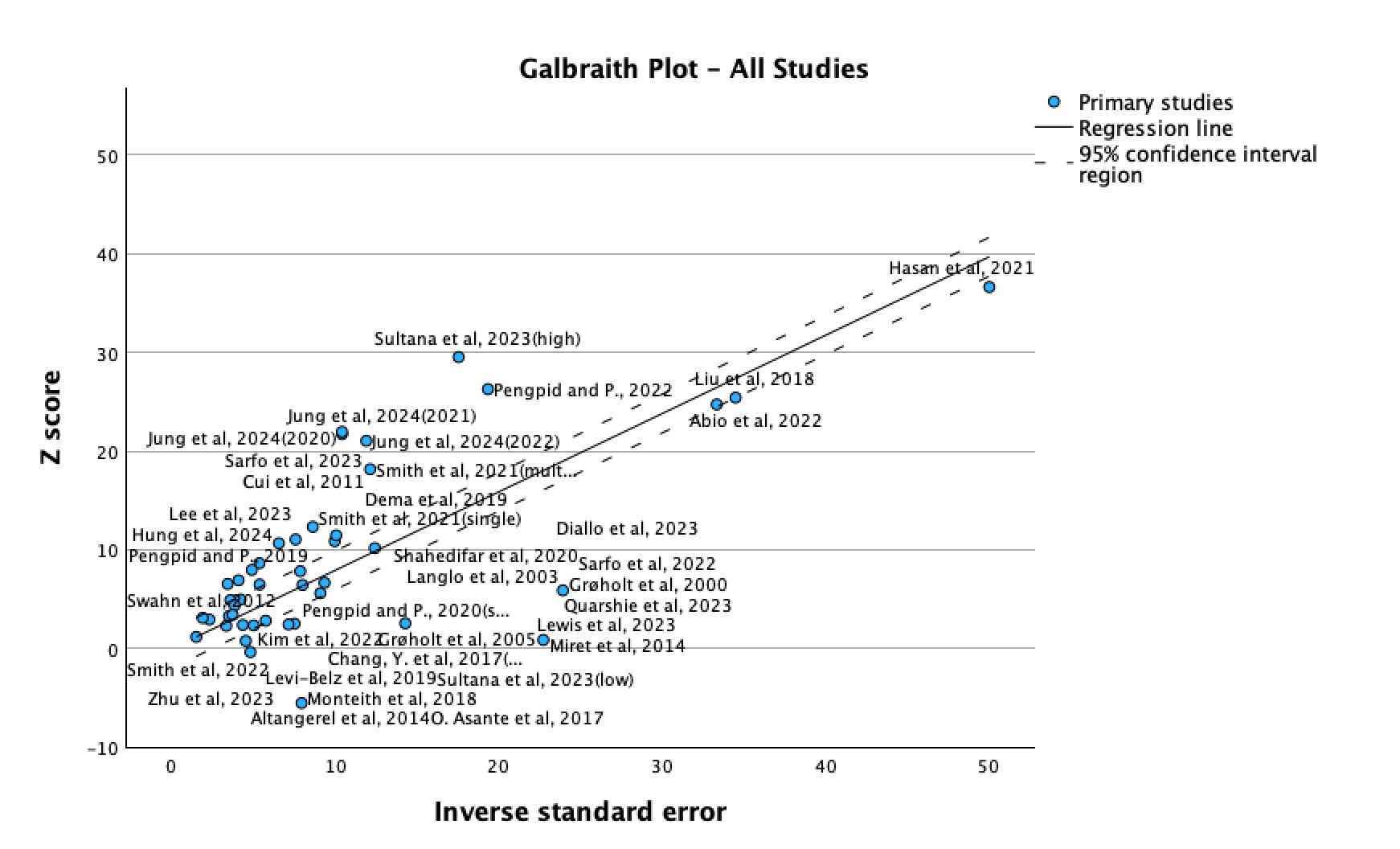

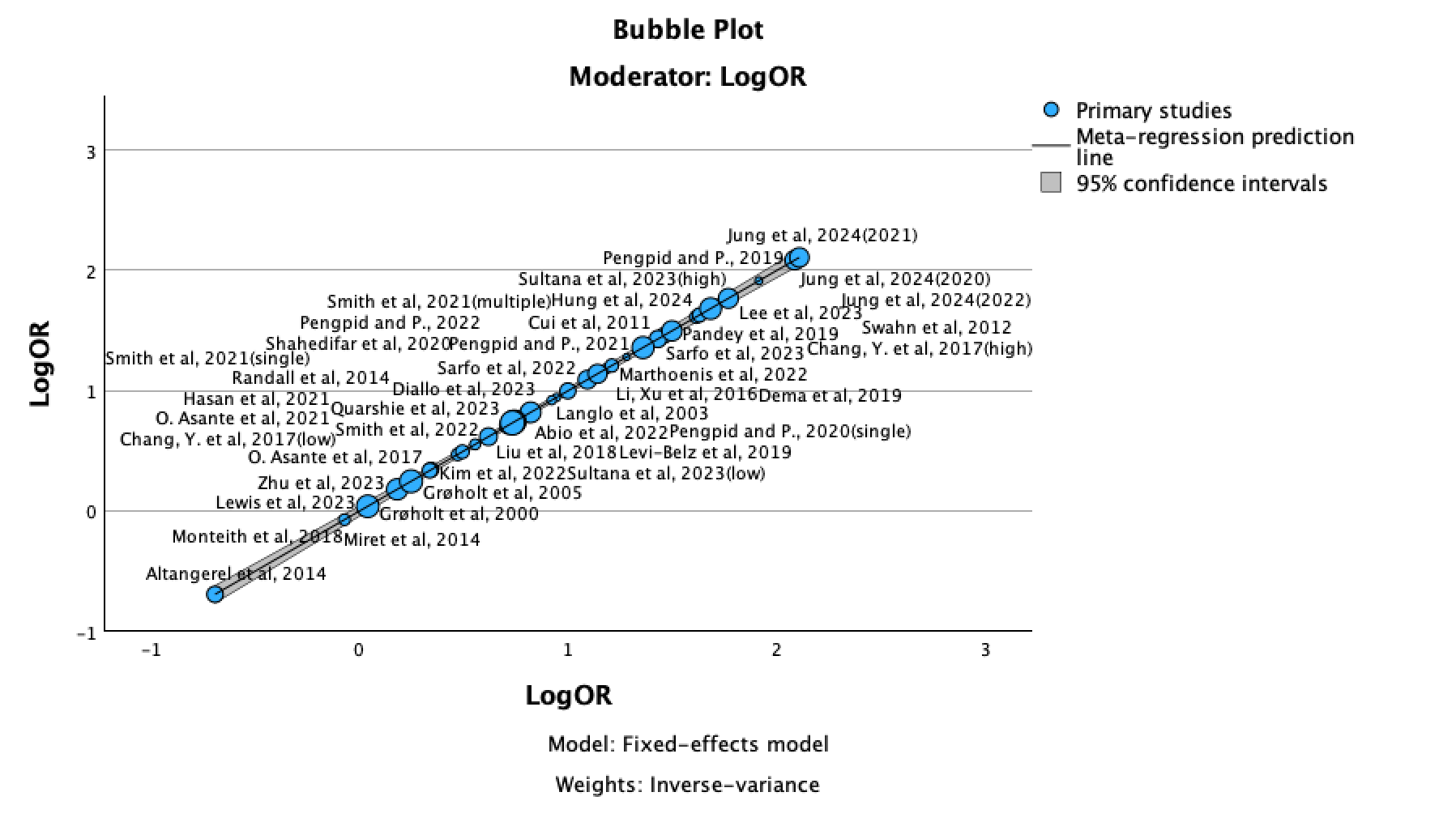
**

**Continuing graphic 5g.**

| **Egger's Regression-Based Test^a^** | | | | | | |
| --- | --- | --- | --- | --- | --- | --- |
| Parameter | Coefficient | Std. Error | t | Sig. (2-tailed) | 95% Confidence Interval | |
|  |  |  |  |  | Lower | Upper |
| (Intercept) | ·675 | ·0157 | 42·871 | <·001 | ·643 | ·706 |
| SE^b^ | 2·281 | ·2200 | 10·367 | <·001 | 1·837 | 2·725 |
| a. Fixed-effects meta-regression | | | | | | |
| b. Standard error of effect size | | | | | | |

| **Effect Size Estimates for Trim-and-Fill Analysis** | | | | | | | |
| --- | --- | --- | --- | --- | --- | --- | --- |
|  | Number | Effect Size | Std. Error | Z | Sig. (2-tailed) | 95% Confidence Interval | |
|  |  |  |  |  |  | Lower | Upper |
| Observed | 44 | ·794 | ·0108 | 73·597 | <·001 | ·772 | ·815 |
| Observed + Imputed^a^ | 51 | ·719 | ·0104 | 69·355 | <·001 | ·699 | ·739 |
| a. Number of imputed studies: 7 | | | | | | | |

**Graphic 5h. Association between loneliness and suicide attempt in cross-sectional studies after asymmetry adjusted 2.**


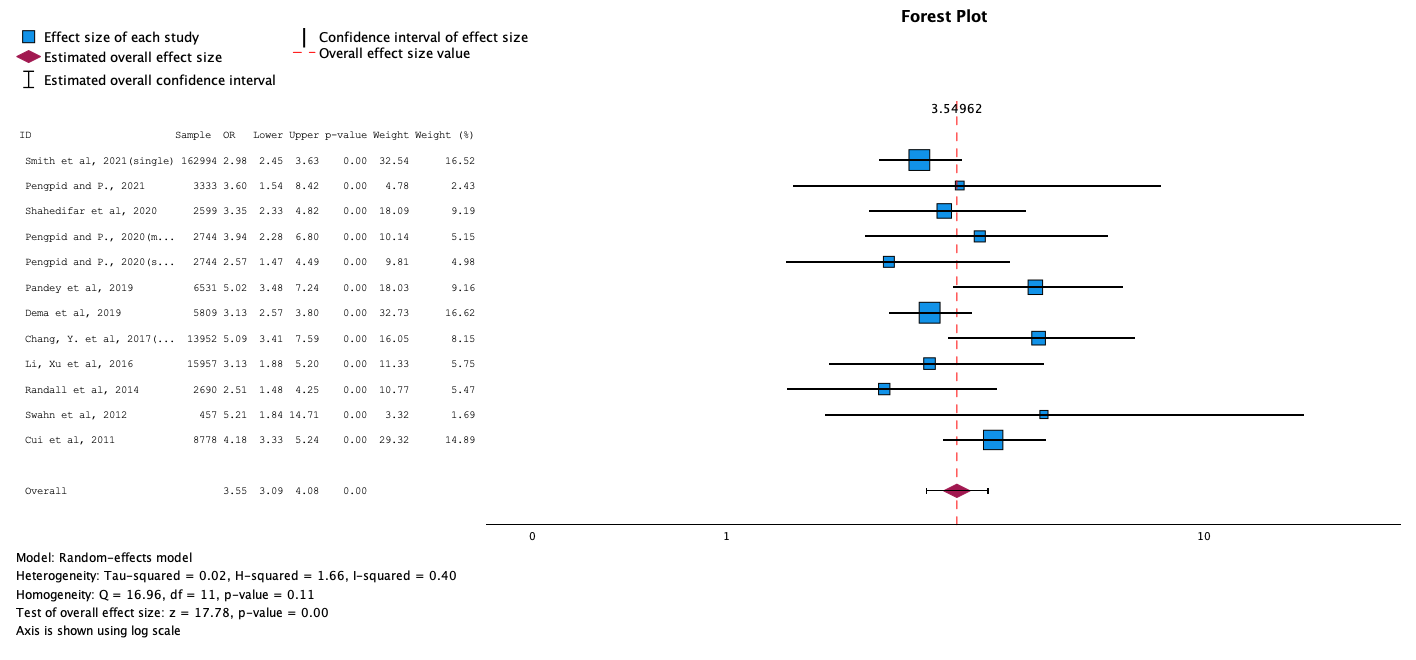

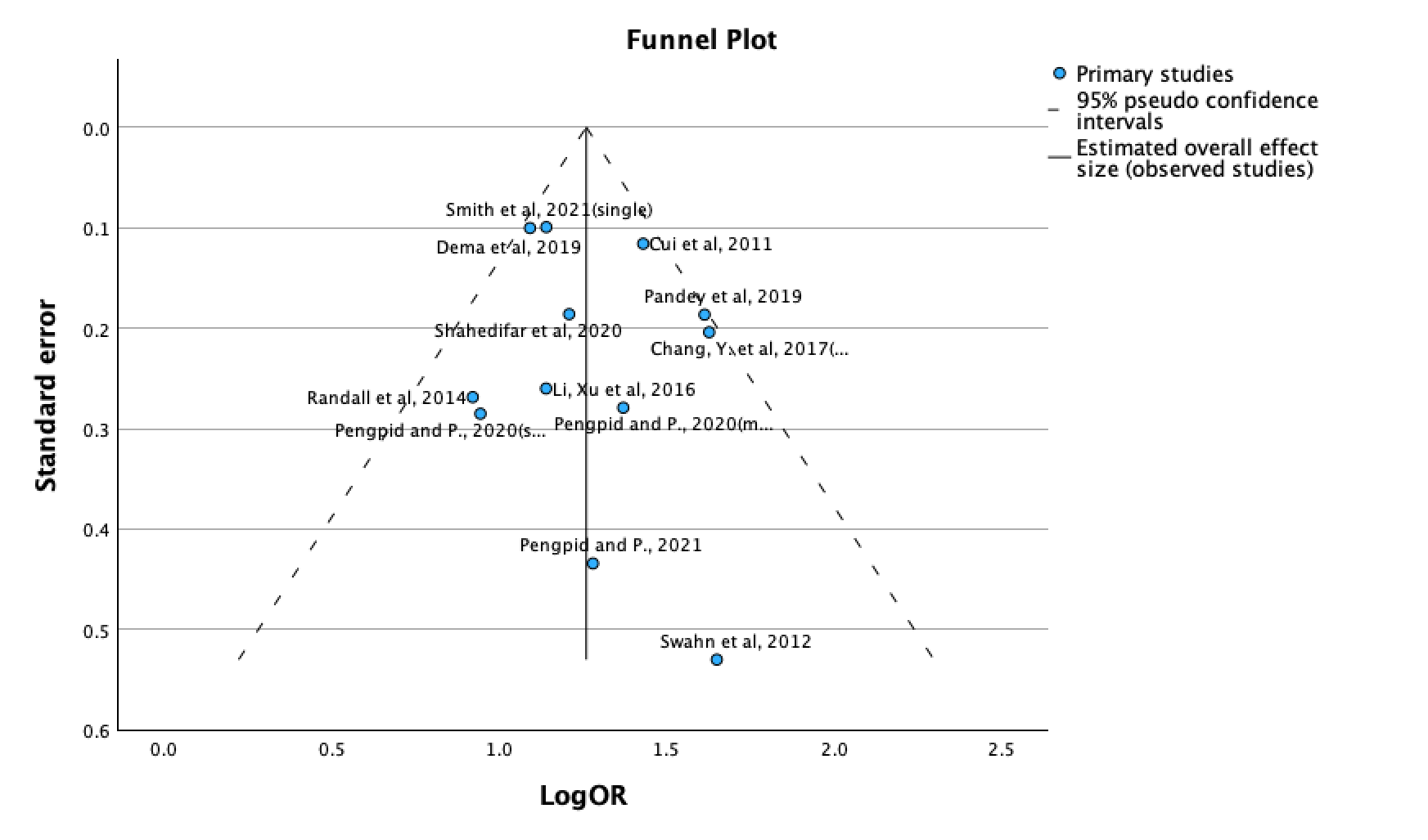


**Continuing graphic 5h.**

| **Egger's Regression-Based Test^a^** | | | | | | |
| --- | --- | --- | --- | --- | --- | --- |
| Parameter | Coefficient | Std. Error | t | Sig. (2-tailed) | 95% Confidence Interval | |
|  |  |  |  |  | Lower | Upper |
| (Intercept) | 1·221 | ·1618 | 7·543 | <·001 | ·860 | 1·581 |
| SE^b^ | ·259 | ·7978 | ·324 | ·752 | -1·519 | 2·036 |
| a. Random-effects meta-regression | | | | | | |
| b. Standard error of effect size | | | | | | |

| **Effect Size Estimates for Trim-and-Fill Analysis** | | | | | | | |
| --- | --- | --- | --- | --- | --- | --- | --- |
|  | Number | Effect Size | Std. Error | Z | Sig. (2-tailed) | 95% Confidence Interval | |
|  |  |  |  |  |  | Lower | Upper |
| Observed | 12 | 1·267 | ·0713 | 17·777 | <·001 | 1·127 | 1·407 |
| Observed + Imputed^a^ | 13 | 1·260 | ·0702 | 17·963 | <·001 | 1·123 | 1·398 |
| a. Number of imputed studies: 1 | | | | | | | |

**Graphic 6. Association between loneliness and suicidal behavior**


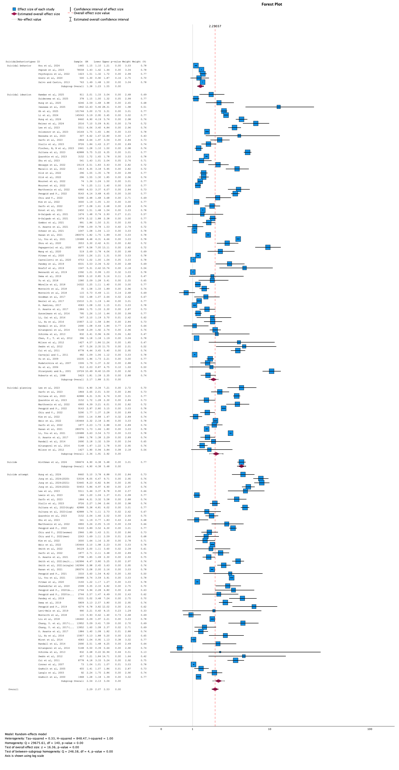

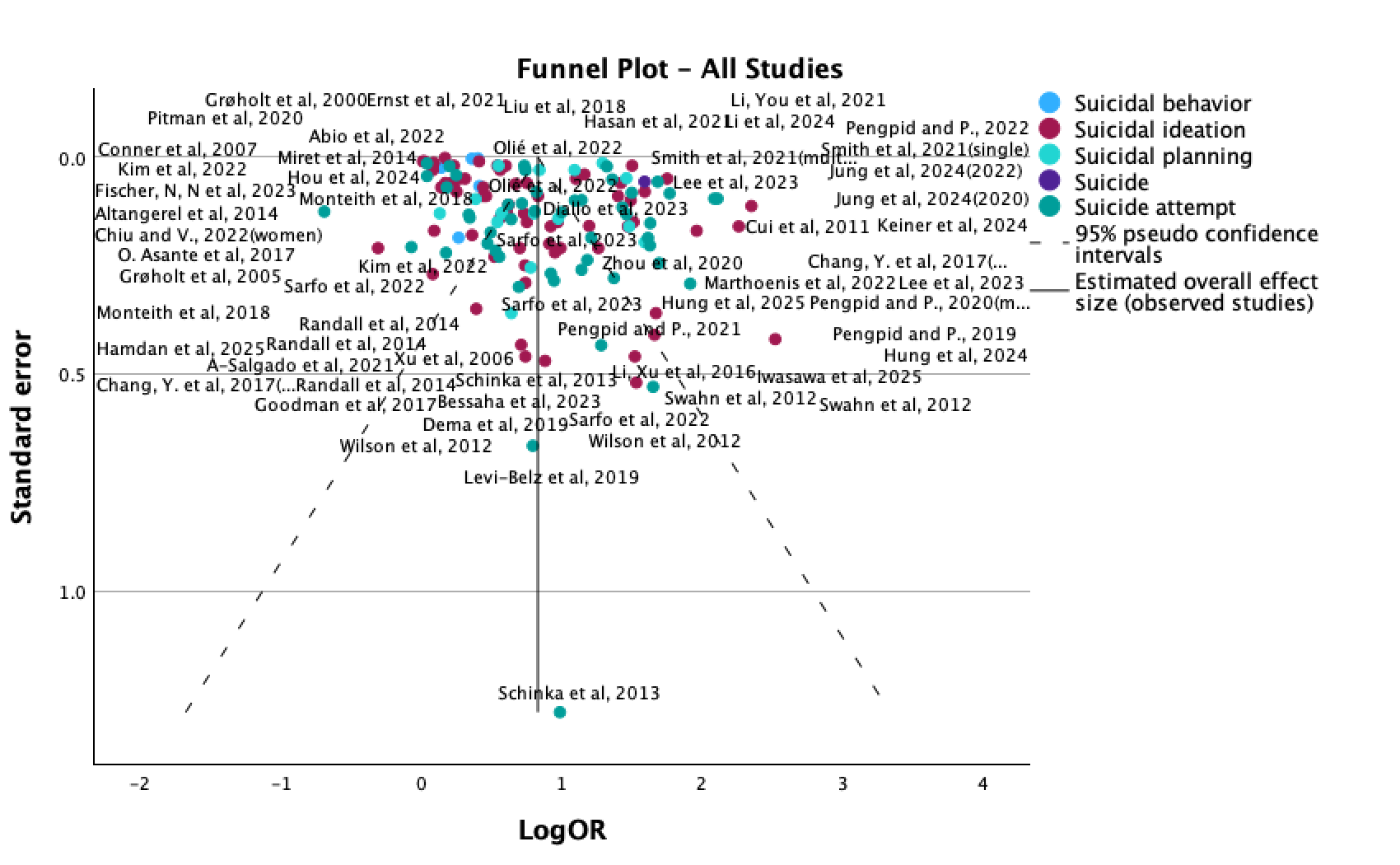


**MAFA – Loneliness as an interface between Alzheimer’s disease and suicidal behavior**

morbosus anxiety depression entrapment hopelessness

morbosus 1.000 0.354 0.398 0.466 0.628

anxiety 0.354 1.000 0.259 0.354 0.397

depression 0.398 0.259 1.000 0.316 0.474

entrapment 0.466 0.354 0.316 1.000 0.466

hopelessness 0.628 0.397 0.474 0.466 1.000

insomnia 0.519 0.467 0.251 0.630 0.475

perceived burdensomeness 0.317 0.625 0.355 0.518 0.249

stress 0.474 0.523 0.398 0.316 0.353

insomnia perceived burdensomeness stress

morbosus 0.519 0.317 0.474

anxiety 0.467 0.625 0.523

depression 0.251 0.355 0.398

entrapment 0.630 0.518 0.316

hopelessness 0.475 0.249 0.353

insomnia 1.000 0.396 0.466

perceived burdensomeness 0.396 1.000 0.626

stress 0.466 0.626 1.000

Q statistic on the homogeneity of effect sizes: 3541.616

Degrees of freedom of the Q statistic: 197

P value of the Q statistic: 0

Heterogeneity indices (based on the estimated Tau2):

Estimate

Intercept1: I2 (Q statistic) 0.9665

Intercept2: I2 (Q statistic) 0.9499

Intercept3: I2 (Q statistic) 0.9763

Intercept4: I2 (Q statistic) 0.0000

Intercept5: I2 (Q statistic) 0.9148

Intercept6: I2 (Q statistic) 0.9196

Intercept7: I2 (Q statistic) 0.0000

Intercept8: I2 (Q statistic) 0.9935

Intercept9: I2 (Q statistic) 0.9665

Intercept10: I2 (Q statistic) 0.9487

Intercept11: I2 (Q statistic) 0.9760

Intercept12: I2 (Q statistic) 0.0000

Intercept13: I2 (Q statistic) 0.8950

Intercept14: I2 (Q statistic) 0.9205

Intercept15: I2 (Q statistic) 0.0000

Intercept16: I2 (Q statistic) 0.9940

Intercept17: I2 (Q statistic) 0.9667

Intercept18: I2 (Q statistic) 0.9486

Intercept19: I2 (Q statistic) 0.9760

Intercept20: I2 (Q statistic) 0.0000

Intercept21: I2 (Q statistic) 0.8942

Intercept22: I2 (Q statistic) 0.9201

Intercept23: I2 (Q statistic) 0.0000

Intercept24: I2 (Q statistic) 0.9939

Intercept25: I2 (Q statistic) 0.9667

Intercept26: I2 (Q statistic) 0.9477

Intercept27: I2 (Q statistic) 0.9764

Intercept28: I2 (Q statistic) 0.0000

Number of studies (or clusters): 26

Number of observed statistics: 225

Number of estimated parameters: 56

Degrees of freedom: 169

-2 log likelihood: -321.8217

OpenMx status1: 6 ("0" or "1": The optimization is considered fine.

Other values may indicate problems.)
